# Cascade reactions of nitrogen-substituted isocyanates: a new tool in heterocyclic chemistry†
†Electronic supplementary information (ESI) available: Complete experimental procedures, characterization data, and NMR spectra. CCDC 1420522 and 1420523. For ESI and crystallographic data in CIF or other electronic format see DOI: 10.1039/c5sc03197d


**DOI:** 10.1039/c5sc03197d

**Published:** 2015-09-23

**Authors:** Jean-François Vincent-Rocan, Ryan A. Ivanovich, Christian Clavette, Kyle Leckett, Julien Bejjani, André M. Beauchemin

**Affiliations:** a Centre for Catalysis Research and Innovation , Department of Chemistry and Biomolecular Sciences , University of Ottawa , 10 Marie-Curie , Ottawa , ON K1N 6N5 , Canada . Email: andre.beauchemin@uottawa.ca

## Abstract

In contrast to normal *C*-substituted isocyanates, nitrogen-substituted isocyanates (*N*-isocyanates) are rare. Their high reactivity and amphoteric/ambident nature has prevented the scientific community from exploiting their synthetic potential. Recently, we have developed an *in situ* formation approach using a reversible equilibrium, which allows controlled generation and reactivity of *N*-isocyanates and prevents the dimerization that is typically observed with these intermediates. This blocked (masked) *N*-isocyanate approach enables the use of various *N*-isocyanate precursors to assemble heterocycles possessing the N–N–C=O motif, which is often found in agrochemicals and pharmaceuticals. Cascade reactions for the rapid assembly of several valuable 5- and 6-membered heterocycles are reported, including amino-hydantoins, acyl-pyrazoles, acyl-phthalazinones and azauracils. Over 100 different compounds were synthesized using amino-, imino- and amido-substituted *N*-isocyanates, demonstrating their potential as powerful intermediates in heterocyclic synthesis. Their reactivity also enables access to unprecedented bicyclic derivatives and to substitution patterns of azauracils that are difficult to access using known methods, illustrating that controlled reactivity of *N*-isocyanates provides new disconnections, and a new tool to assemble complex N–N–C=O containing motifs.

## Abstract 2

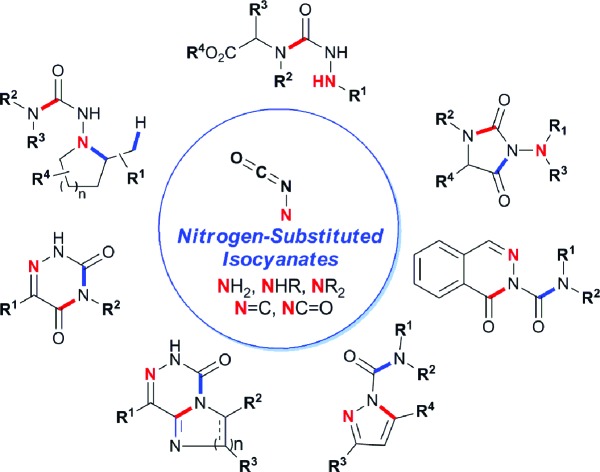
Rare and amphoteric intermediates, *in situ* generation: controlled reactivity in diverse cascade reactions, over 100 examples.

## Introduction

Since their discovery in 1848 by Wurtz, isocyanates have received significant attention from the synthetic community.^1^ Isocyanates are important bulk and fine chemicals and are used industrially in coatings, paints, foams, adhesives, elastomers, and as building blocks for pharmaceuticals and agrochemicals. Over 4000 isocyanates are also commercially available. Perhaps most notably, isocyanates are used to form polyurethanes. In 2011, 14 million tons of polyurethanes were produced, corresponding to *ca.* 5% of the global polymer market.1*d* Consequently, it is difficult to downplay the industrial importance of isocyanates.1*d*

The high reactivity of isocyanates is essential to many industrial processes but their promiscuous nature can be problematic. Therefore, for a variety of applications the use of *isocyanate precursors* is enabling. Classical rearrangement reactions such as the Curtius, Schmidt, Lossen and Hoffman rearrangements are commonly used to form isocyanates *in situ*. In contrast, blocked (masked) isocyanates are simple precursors releasing isocyanates through a chemical equilibrium. Such blocked isocyanates—typically generated from an isocyanate and a blocking group (*e.g.* phenol, *t*-BuOH, caprolactam, 3,5-dimethylpyrazole and methyl ethyl ketoxime)^2^—allow the release of isocyanates upon heating or using catalysis, which can be fine-tuned for use in a selected application. Blocked-isocyanates allow process engineering, and have thus been extensively studied^2^ for two main reasons: (1) the slow release of isocyanates at different temperatures is a useful way of controlling isocyanate concentration and reactivity, which can ultimately minimize side reactions and lead to products with different properties; (2) isocyanates are known to have acute toxicity, which can lead to worker sensitization upon exposure. Therefore, blocking groups have been carefully investigated and various oxygen, nitrogen and even carbon-based blocking groups have emerged with reactivity suited to their intended uses or their mode of activation (*e.g.* thermolysis, base, acid, and metal catalyzed formation).^2^

Isocyanates attached to heteroatoms are also known, but are less developed and used than normal *C*-substituted isocyanates.^4^ Nitrogen-substituted isocyanates (*N*-isocyanates) are a class of heterocumulene possessing comparable synthetic potential to *C*-substituted isocyanates. However, despite the early discovery of *N*-isocyanates,4a,b the synthetic potential of these reactive intermediates remains virtually untapped. Their scarcity in the literature is likely a consequence of their amphoteric nature,^3^ as both a nucleophilic nitrogen atom and an electrophilic isocyanate are present on the same molecule. They share the same connectivity as α-amino aldehydes, and these amphoteric molecules are also notoriously difficult to handle.^3^ Importantly, the amphotericity of *N*-isocyanates results in difficult syntheses and a propensity for these intermediates to homodimerize or oligomerize, even at temperatures as low as –40 °C.4*ba*

Much of the early work studied the formation of *N*-isocyanates, and only explored their reactivity.^4^ The formation of these intermediates was performed through a Curtius rearrangement of carbamoyl azides,4c,d,e,x ring opening induced formation4h,z,ab,ac,ag,al,am,ap,ar or by thermolysis of hydrazide derivatives.4a,b,h,ak,aq,aw,ax Most studies only described their solvolysis but more complex cycloaddition reactions4ac,ad,ag,al,an and intramolecular cyclizations4c,d,x,ar were also reported. Recently, Maier4*aq* and Wentrup4*bd* have observed and studied the reactivity of *N*-isocyanates under UV photolysis conditions. Despite this pioneering work, the reaction conditions required to form these amphoteric intermediates, and their propensity to dimerize, severely limited their synthetic applications. To date, only a few reactions are reported using *N*-isocyanates or their blocked derivatives. After an extensive literature search, we only found 57 publications either forming, studying, using or suggesting the formation of *N*-isocyanates and *N*-isothiocyanates.^4^ This number is probably underestimated as some reported reactivity could likely be attributed to *N*-isocyanate intermediates. In contrast, there are over 100 000 publications and patents on *C*-isocyanates, and over 7000 on blocked *C*-isocyanates. The difference between the synthetic uses of *C*-isocyanates *vs. N*-isocyanates is inherently proportional, and highlights the need for a convenient procedure to form *N*-isocyanates and control their reactivity. Recently we have developed several blocked *N*-isocyanate precursors as part of our efforts on intra- and intermolecular alkene aminocarbonylation reactions (eqn (1)). These reactions involve *N*-isocyanates as key intermediates in a reaction sequence allowing the transformation of alkenes into valuable β-aminocarbonyl motifs. This work required the development of practical reagents: the use of hydrazide and hydrazone derivatives as *N*-isocyanate precursors emerged as a practical and general approach for these reactions. Most importantly, it allowed the desired concerted [3 + 2] alkene cycloaddition to occur in high yield, with little *N*-isocyanate dimerization or decomposition (especially in the presence of excess alkene).^5^ In addition, the reactivity observed is well aligned with the blocked *C*-isocyanate literature: (1) they appear to follow similar deblocking temperature trends; (2) base catalysis is also possible; (3) observations support a reversible equilibrium favoring the hydrazide and hydrazone starting materials.
1

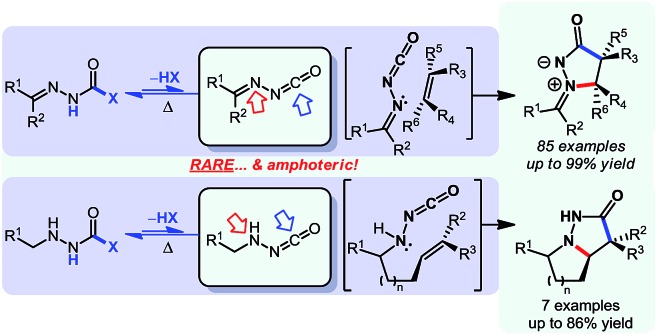




**Scheme 1:**
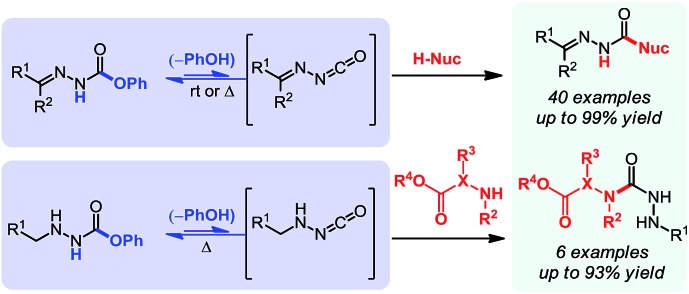
Synthetic applications of *N*-isocyanate intermediates.

**Fig. 1:**
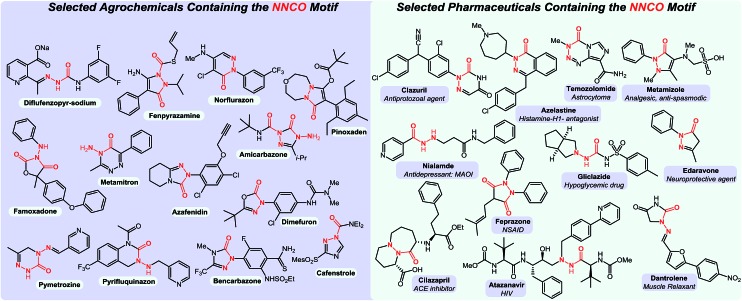
Prevalence of N–N–C=O motif in the agrochemical (left) and pharmaceutical industries (right).

In parallel to this aminocarbonylation work, we noticed the paucity of simpler reactions of *N*-isocyanates, for example with alcohols, amines, and thiols as nucleophiles. This encouraged us to investigate the generation and reactivity of *N*-isocyanate precursors with simple nucleophiles. Gratifyingly, the substitution reactions proceeded efficiently under *stoichiometric* conditions using both hydrazones^6^ and hydrazides^7^ as blocked *N*-isocyanates (Scheme 1). A comparison of the conditions below establishes that imino-isocyanates react more readily than amino-isocyanates. This reactivity trend is in line with the observed increased reactivity of aromatic *C*-isocyanates relative to aliphatic *C*-isocyanates in related reactions.2*a* Exploring this fundamental reactivity turned our attention to the prevalence of the N–N–C=O motif in complex bioactive molecules, including several marketed agrochemicals and pharmaceuticals (Fig. 1). There are various N–N–C=O containing substructures: fully or partially incorporated within a heterocycle, or in acyclic molecules.

Therefore, the development of strategies for the incorporation of the N–N–C=O motif in complex molecular scaffolds is highly desirable. However, it is a considerable challenge due to the diversity of motifs present and due to intrinsic chemoselectivity issues associated with hydrazine derivatives: the presence of two nitrogen atoms that can react and lead to different products.^9^ Considering the underdevelopment of *N*-isocyanate chemistry and the diversity of N–N–C=O motifs present, it was hypothesized that *N*-isocyanates could provide the missing link for a unified approach to N–N–C=O incorporation in heterocyclic chemistry; provided that their reactivity could be controlled enough to allow for new cascade reactions. This article constitutes a detailed account of our work toward this goal.

Herein, we discuss the first cascade reactions developed using amino-, imino- and amido-substituted *N*-isocyanates for the synthesis of heterocyclic molecules. In addition to previously communicated work toward saturated 5- and 6-membered azacycles and nitrogen-substituted hydantoins,^7^,^8^ we report new cascade reactions furnishing important heteroaromatic cores incorporating the N–N–C=O motif in several different orientations. *N*-Isocyanates were used to assemble 5- and 6-membered aromatic heterocycles including acyl-pyrazoles, acyl-phthalazinones and azauracils. This novel synthetic approach gives rise to substitution patterns that have otherwise been difficult or impossible to access, and allows the formation of new bicyclic heterocycles. With over *100 new compounds spanning 6 heterocyclic classes* assembled using cascade reactions of amphoteric *N*-isocyanate intermediates, this article highlights that highly controlled reactivity is possible through the use of blocked (masked) *N*-isocyanate precursors.

## Results and discussion

To acquire proof of concept results to validate that the controlled reactivity of *N*-isocyanate precursors could lead to efficient cascade reactions, we first targeted a reaction sequence in which generation of the *N*-isocyanate and reaction at its electrophilic carbon would occur first, followed by cyclization. It was expected that the substitution of nitrogen nucleophiles (*e.g.* amines) on *N*-isocyanates would be essentially irreversible. Building on our expertise in metal-free hydroamination reactions of hydrazine derivatives,^10^ we developed an *N*-isocyanate addition/Cope-type hydroamination cascade for the formation of saturated nitrogen heterocycles (eqn (2)), illustrated below using blocked *N*-isocyanate **1a**.^7^
2

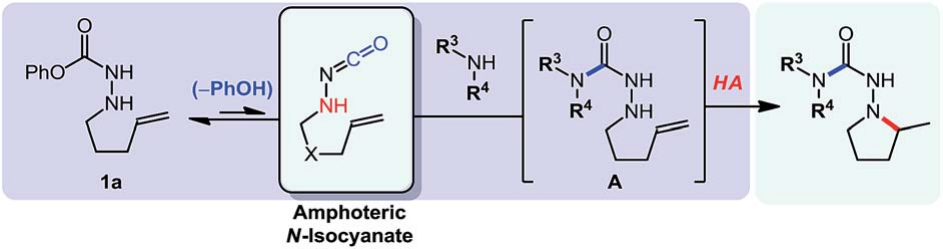




Gratifyingly, *N*-isocyanate precursors allowed the formation of several 5- and 6-membered nitrogen heterocycles incorporating one nitrogen atom (βN) of the amino-isocyanate in the desired heterocycle (Table 1). This reaction sequence involves nucleophilic attack of the amine on the *in situ* generated *N*-isocyanate to form the corresponding semi-carbazide (**A**), which then undergoes a Cope-type hydroamination to form the nitrogen heterocycle. Since isocyanate generation/addition occurred rapidly (*ca.* <10 minutes at 80 °C), the hydroamination reaction was rate limiting and the build-up of the unsaturated semi-carbazide **A** was observed when monitoring these reactions. However, upon heating at temperatures allowing hydroamination to occur, this cascade allowed the synthesis of semi-carbazide-based pyrrolidines (**2a**, **d**, **f–h**), piperidines (**2b**, **e**) and piperazine (**2c**) using pyrrolidine as the nucleophilic amine. As expected, substitution was well tolerated on the alkenyl chain, and incorporation of a Thorpe–Ingold bias was beneficial to achieve cyclization at a lower temperature (**2d**) or to reduce the time required for reaction completion (**2e**). Unfortunately, the incorporation of a small chiral centre on the alkenyl chain didn't result in any diastereoselecitivty (**2f**, d.r: 1 : 1). The cascade reaction also allowed cyclization *via* the more challenging hydroamination of an internal alkene (**2h**). A protected alcohol on the alkene chain was also tolerated (**2g**) and could allow further functionalization of the desired product. In addition to providing a cascade for the rapid assembly of molecular complexity, this data showed that semi-carbazide formation is essentially irreversible at temperatures up to 175 °C, a useful finding for the development of other cascade reactions.

**Table 1:** Scope of the *N*-isocyanate addition/hydroamination cascade^*a*^

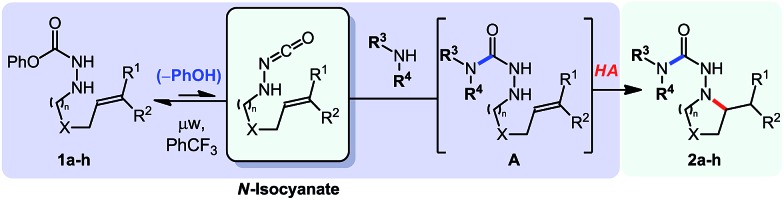
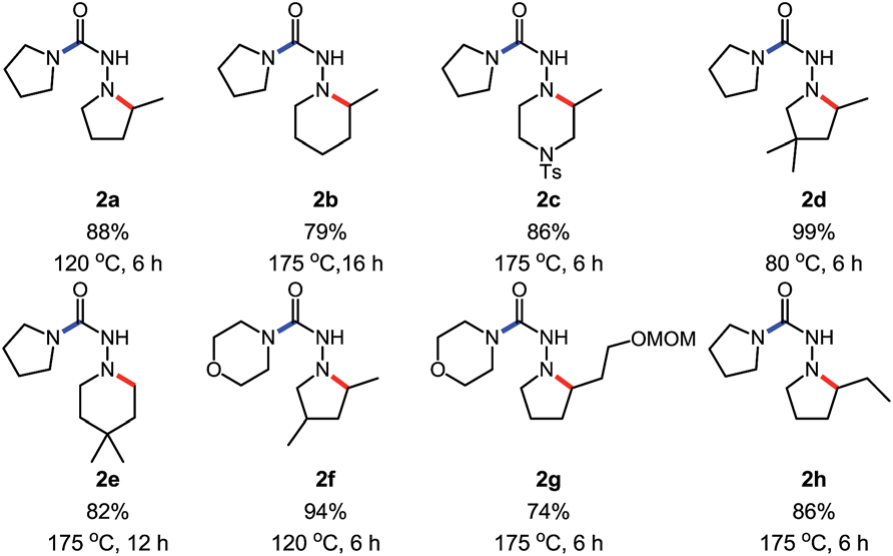

^*a*^Conditions: carbazate (1 equiv.), amine (1.1 equiv.) in PhCF_3_ (0.3 M) heated in a sealed vial (microwave reactor).

Before developing other cascade reactions, we decided to address an important limitation of intramolecular hydroamination reactions.^11^ Indeed, these reactions are generally not well suited for the rapid generation of molecular complexity. The key limitation is that each substrate is typically prepared in several steps, and can only provide a single hydroamination product. In contrast, the use of *N*-isocyanate precursors in cascade reactions allows the formation of multiple hydroamination substrates from a common precursor, followed by intramolecular hydroamination events. This approach led to a diversity-oriented synthesis of pyrrolidines shown in Table 2.

**Table 2:** Cascade synthesis of multiple hydroamination products from a single *N*-isocyanate precursor^*a*^

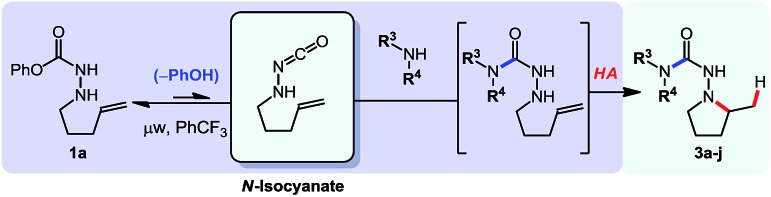
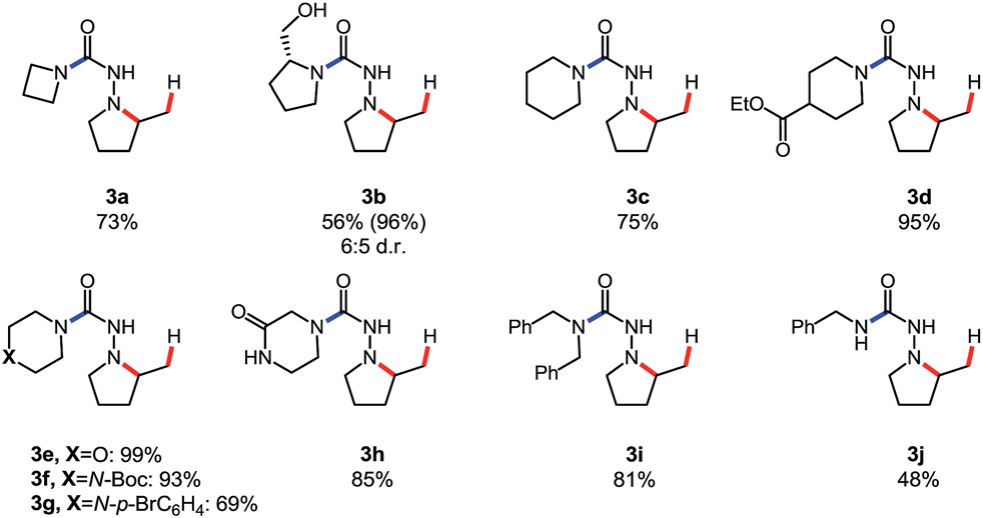

^*a*^Conditions: carbazate (1 equiv.), amine (1.1 equiv.) in PhCF_3_ (0.3 M) heated in sealed vial (microwave reactor, 120 °C, 6 h).

Using this *N*-isocyanate addition/Cope-type hydroamination cascade ten different semi-carbazide-based pyrrolidines were synthesized from the same carbazate precursor (**1a**, Table 2). Cyclic and acyclic amine nucleophiles were tolerated in this cascade reaction. Azetidine (**3a**), and piperidine derivatives (**3c–g**) yielded the desired products in good to excellent yields. (*S*)-Prolinol was also a competent nucleophile but only showed modest diastereoselectivity. Product **3g** bearing a bromine atom was also formed to highlight the potential of this metal-free method. The medicinally relevant 2-oxopiperazine demonstrated chemoselectivity for the most nucleophilic nitrogen, providing the desired heterocycle **3h** in 85% yield. While both acyclic and cyclic secondary amines proved competent reaction partners, the result with primary amine **3j** led to a modest, but preparatively useful yield. We attribute this difference of reactivity to a more challenging hydroamination step, due to the relative population of the *E*- and *Z*-conformers of the semi-carbazide.

**Scheme 2:**
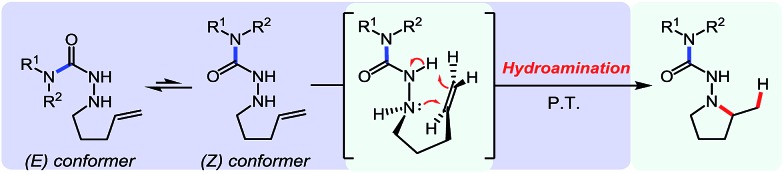
Impact of the hydrazide conformation on hydroamination reactivity.

The semi-carbazide formed from benzylamine has increased conformational flexibility, and its *E* hydrazide conformer is thermodynamically favoured.9*b* In contrast, a destabilizing A(1,3) allylic strain interaction is present in the adducts of secondary amines (*i.e.* destabilizing interaction between R^2^ and βN in the *E*-conformer). Thus, the *Z*-conformer of these intermediates is thermodynamically favoured. Previous DFT studies suggest that the *Z*-conformer is the reactive conformer in Cope-type hydrohydrazidations (Scheme 2).5*a*

**Scheme 3:**
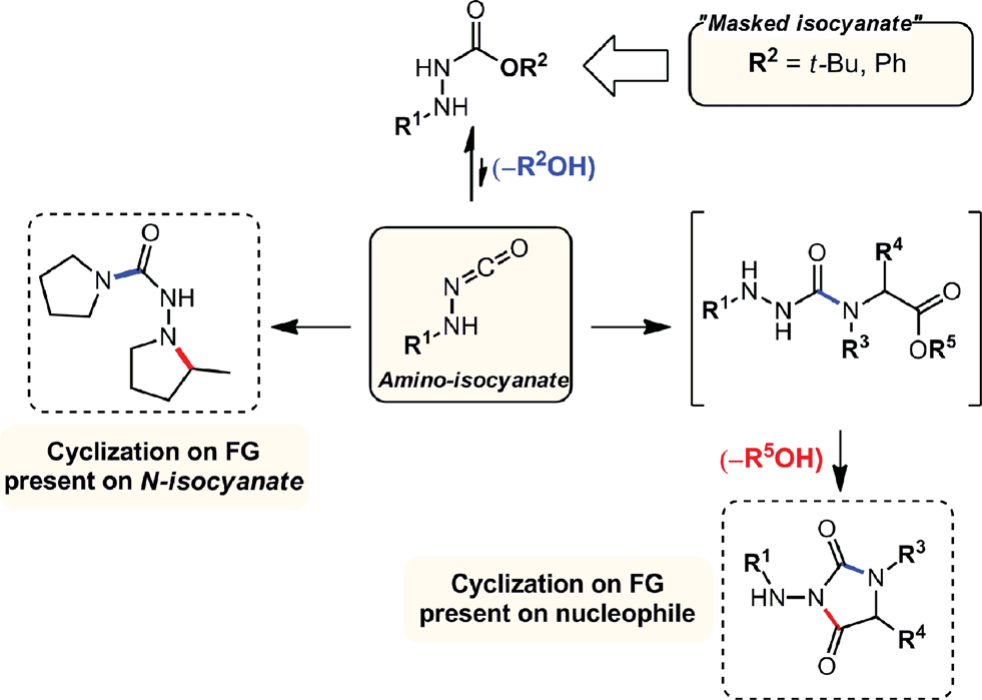
Comparison of hydantoin synthesis *versus* hydroamination cascade.

Overall, this study was the first example of a cascade reaction using amphoteric amino-isocyanates generated *in situ* from carbazates. Strategically, this methodology used an external nucleophile to generate a derivative in which the βN subsequently participated in the cyclization event (hydroamination), with an alkene present on the *N*-isocyanate substrate. To further develop cascade reactions of *N*-isocyanates, we were drawn to different cascade reactions in which cyclization would occur on a functional group (FG) present on the incoming nucleophile. Recently, we reported such a cascade reaction using α-amino esters to rapidly assemble *N*-substituted hydantoins (Scheme 3).^8^ Since encouraging results were obtained for substitution reactions using amino-esters on blocked *N*-isocyanates (Scheme 3), we rationalized that we could develop a new substitution/cyclization reaction cascade. This cascade was first investigated with amino-isocyanates since two different products could be formed depending on which nitrogen would cyclize.

Indeed, cyclization using the proximal nitrogen (αN) would yield the 5-membered amino-hydantoin, while cyclization using the distal nitrogen (βN) would yield the 6-membered aza-diketopiperazine.^13^ We tested the reaction with a proline ester, and were pleased to observe complete selectivity for amino-hydantoin formation (eqn (3)).^12^ After this initial result, we decided to further explore this reactivity using *N*-benzyl carbazate and several amino-esters (Table 3).
3

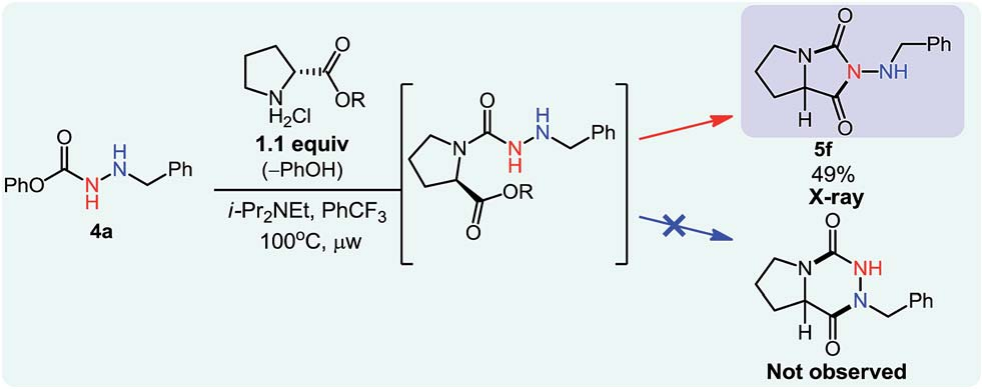




**Table 3:** Scope of the cascade synthesis of amino-hydantoins using carbazates^*a*^

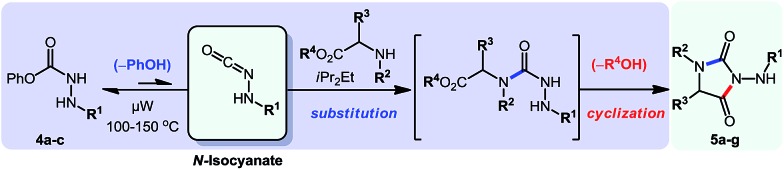
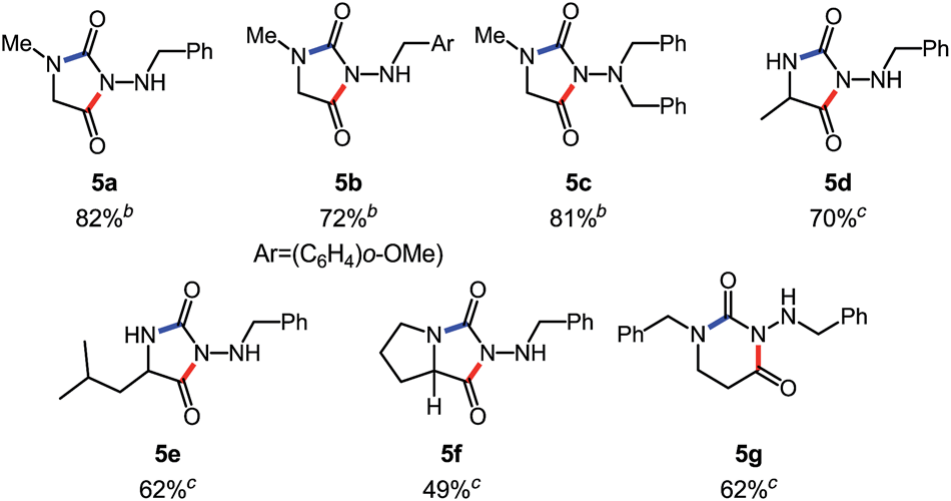

^*a*^Conditions: carbazate (1 equiv.), i-Pr_2_NEt (1.2 equiv.), aminoester hydrochloride (1.1 equiv.) in PhCF_3_ (0.3 M) heated in a sealed vial (microwave reactor, 100 °C or 150 °C, 6 h).

^*b*^Reaction at 100 °C.

^*c*^Reaction at 150 °C.

As shown in Table 3, the cascade reaction proved efficient for several *N*-substituted glycine esters (**5a**, **5b** and **5c**). Alanine (**5d**), leucine (**5e**) and proline (**5f**) esters also cyclized in moderate to good yields. However, racemization occurred under these reaction conditions. In addition, we were pleased to observe the formation of a dihydrouracil ring (**5g**) in moderate yield using a *N*-benzyl β-aminoester as the reaction partner. Since the reaction was completely selective for the cyclization on the proximal nitrogen (αN), we decided to expand this study to include other *N*-isocyanate precursors. As indicated in the introduction, differences are expected and observed for the formation and reactivity of diverse *N*-isocyanates. In the context of this cascade reaction we wondered if blocked precursors of other amino-isocyanates (βNsp^3^, carbazate precursors), imino-isocyanates (βNsp^2^, carbazone precursors), and amido-isocyanates would be suitable reaction partners. The results using various *N*-isocyanate precursors are presented in Table 4. Gratifyingly, a variety of carbazones proved competent reaction partners with *N*-alkyl glycine esters (Table 4). It should be highlighted that carbazones are typically excellent *N*-isocyanate precursors: easier to synthesize, stable, often crystalline yet in general more prone than amino-isocyanates to react with nucleophilic amines.^6^

Aliphatic carbazones afforded the desired hydantoins in excellent yields (**7a**, **7b**). We were also pleased that electron-rich and electron-poor aromatic carbazones both led to efficient product formation (**7c–e**). We then surveyed the reactivity of bulky keto-carbazones: acetophenone, fluorenone and diisopropyl ketone-derived reagents afforded the cyclized products in good yields (**7f–h**). A heteroaromatic carbazone also produced the desired heterocycle in good yield (**7i**). We also investigated the use of several *N*-glycine esters using the aldcarbazone derived from 4-methoxybenzaldehyde as a test substrate. The somewhat hindered *N*-isopropyl glycine ester afforded the desired hydantoin **7j** in moderate yield. Functional groups such as nitriles (**7k**) and esters (**7l**) were tolerated on the nitrogen substituent. Even *N*-aryl glycine esters provided the *N*-aryl substituted amino-hydantoins. This indicates that electron-rich (**7n**), electron-neutral (**7m**) and even electron-poor (**7o**) anilines are competent nucleophiles under the reaction conditions. We then used this late-stage functionalization strategy to synthesize 5 azumolene analogues (**7s–w**), without the use of chromatography (*i.e.* purified by filtration). Finally, we performed exploratory attempts toward three related cascades. These proved rewarding as we showed that: (1) imidazolidinone (**7p**) formation was possible if ring closure was achieved *via* 1,4-addition (rather than 1,2-addition), using an α,β-unsaturated amino-ester as reagent; (2) an *N*-isothiocyanate precursor also engaged in a related cascade^14^ to form an amino-thiohydantoin (**7q**); (3) amide-substituted hydantoin (**7r**) could be synthesized using an amido-isocyanate precursor. Collectively, this data suggested that a variety of *N*-isocyanate precursors could engage in cascade reactions and display similar reactivity.

**Table 4:** Cascade synthesis of *N*-substituted-hydantoins using carbazones and other *N*-isocyanate precursors^*a*^

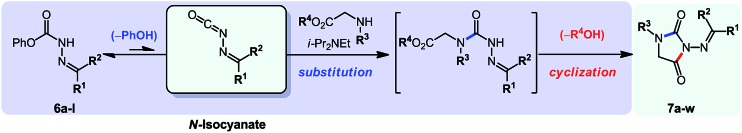
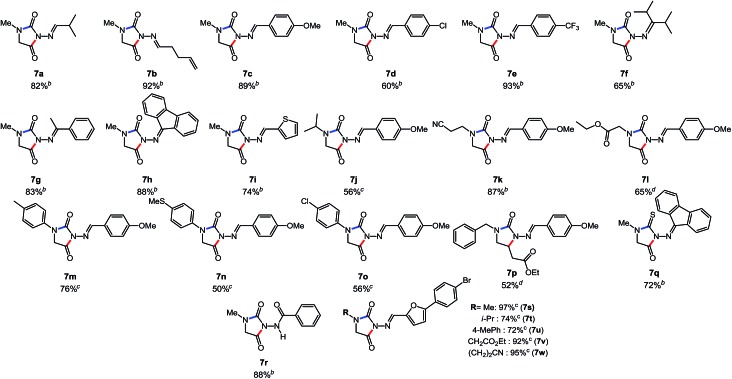

^*a*^Conditions: carbazone (1 equiv.), i-Pr_2_NEt (1.2 equiv.), aminoester hydrochloride (1.1 equiv.) in PhCF_3_ (0.3 M) heated in a sealed vial (microwave reactor, 100–150 °C, 3–6 h).

^*b*^Reaction at 100 °C.

^*c*^Reaction at 150 °C.

^*d*^Reaction at 120 °C.

Considering the encouraging results obtained with the two reaction sequences presented above, we felt confident that we could expand this chemistry to different synthetic targets. The diversity of *N*-isocyanates that could be used to form *N*-substituted hydantoins suggested that *O*-phenyl carbazate itself could be a building block for the incorporation of the N–N–C=O motif: indeed it could serve as the precursor to the simplest possible *N*-isocyanate, NH_2_–NCO (eqn (4)).4*aq*
4

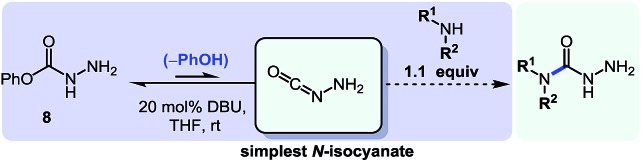




Initially we wondered if the lack of steric shielding on the distal nitrogen atom (*i.e.* NH_2_*vs.* NHR, previously) would result in a greater propensity to dimerize. We thus became interested in achieving even milder reactivity through the use of base catalysis. Previous studies conducted in the context of our alkene aminocarbonylation work showed that bases (*e.g.* Et_3_N) led to imino-isocyanate formation under milder conditions.5*d* Related literature on blocked *C*-isocyanates^2^ also suggested that base catalysis could have broad applicability for other *N*-isocyanate precursors, which could prove an asset for the development of other cascade reactions. Gratifyingly using 20 mol% of DBU with *O*-phenyl carbazate proved to be a convenient way of generating NH_2_–NCO at room temperature. This was performed in the presence of a nucleophilic amine (1.1 equiv.) and afforded the desired semicarbazide products (Table 5).

**Table 5:** Base catalyzed synthesis of unprotected semi-carbazides^*a*^

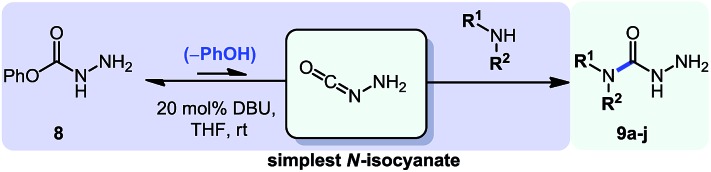			
Entry	Nucleophile	Product	Yield (%)
1 (**9a**)	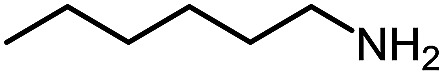	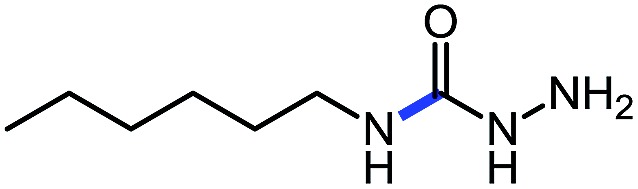	83
2 (**9b**)	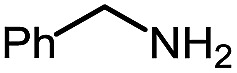	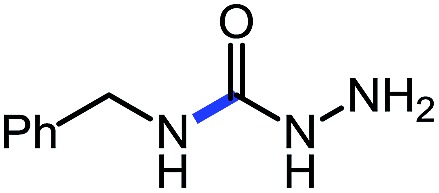	77
3 (**9c**)	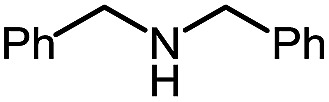	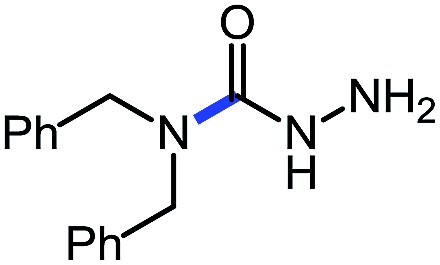	94
4 (**9d**)	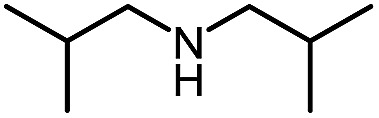	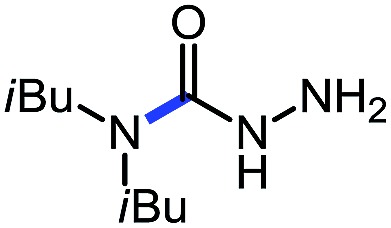	76
5 (**9e**)	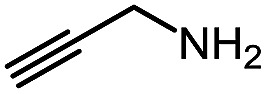	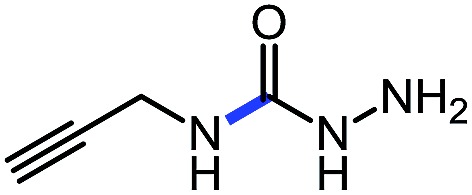	53
6 (**9f**)	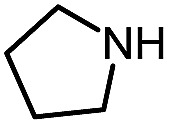	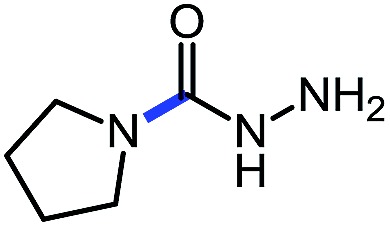	73
7 (**9g**)	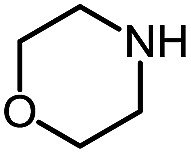	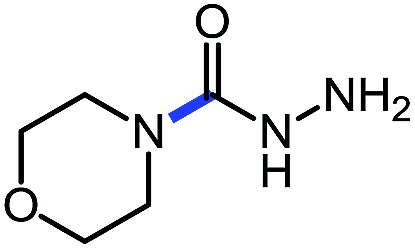	83
8 (**9h**)	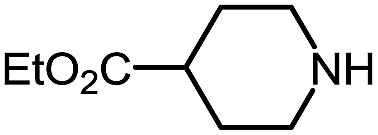	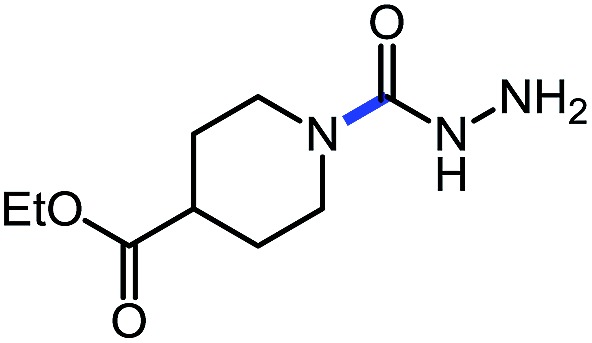	85
9 (**9i**)^*b*^	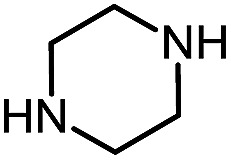	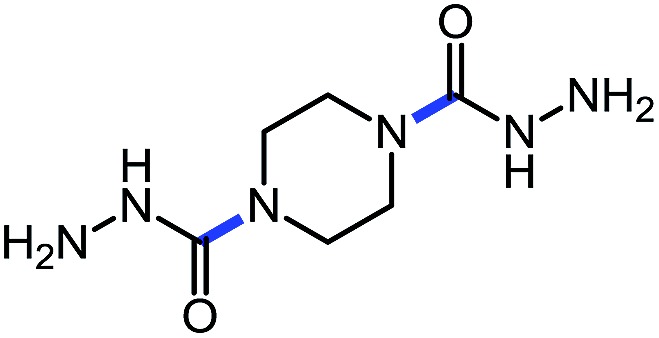	91

^*a*^Conditions: *O*-phenyl carbazate (1 equiv.), amine (1.1 equiv.), DBU (20 mol%) in THF (0.3 M) stirred at room temperature for 16 h.

^*b*^Reaction with 0.5 equiv. amine.

Using this base catalysis procedure, we studied the reaction of different amines with *O*-phenyl carbazate: the results are shown in Table 5.^15^ Several semi-carbazides could be readily formed at room temperature by combining amines and *O*-phenyl carbazate using base catalysis. The use of hexylamine led to an 83% yield of the corresponding semi-carbazide (entry 1, **9a**). Both primary (entry 2, **9b**) and secondary (entry 3, **9c**) benzylic amines proved competent reactants yielding the desired semi-carbazide in good to excellent yield. Propargylamine underwent the substitution reaction providing the propargylic semi-carbazide in modest yield (entry 5, **9e**). In general, both acyclic and cyclic secondary amines were tolerated (entries 4, 6 and 7). An ester functionality was also tolerated to yield the substituted piperidine based semi-carbazide (entry 8, **9h**). Finally, we were pleased to observe that double substitution could also be achieved using piperazine (entry 9, **9i**). Overall, the data shown in Table 5 showed that simple reactions of *N*-isocyanates could also benefit from base catalysis, with no detectable dimerization or oligomerization occurring under the reaction conditions. This new reactivity also provided a new route to semi-carbazides that could serve as building blocks for more complex derivatives.^16^

A natural extension was to explore if *O*-phenyl carbazate could also engage in established cascade reactions. The advantage of this strategy is the ability to provide a free NH_2_ group for further derivatization reactions. We were quite pleased to see that the reaction with *N*-methyl glycine ethyl ester provided the NH_2_-substituted hydantoin in 91% yield on gram scale (eqn (5)). We then used the NH_2_ group to form pyrrole-substituted hydantoin **10b** in 90% yield (eqn (6)). We were also able to synthesize an imidazolidinone derivative through a *N*-isocyanate cascade exploiting addition/cyclization by 1,4-addition (eqn (7)).
5

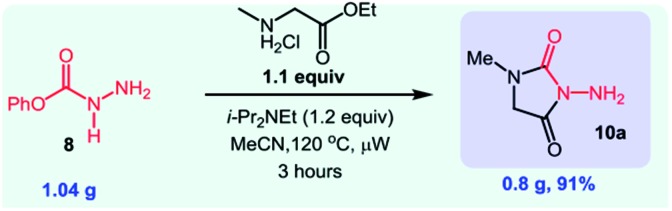



6

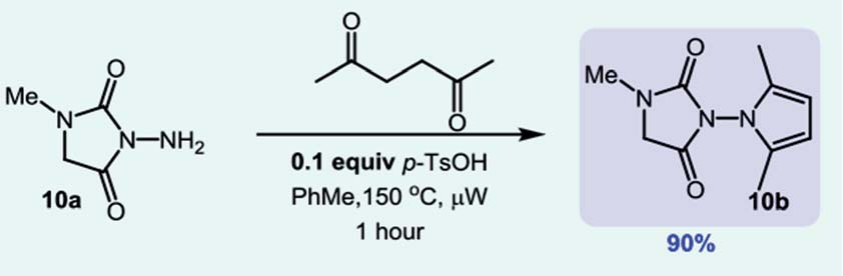



7

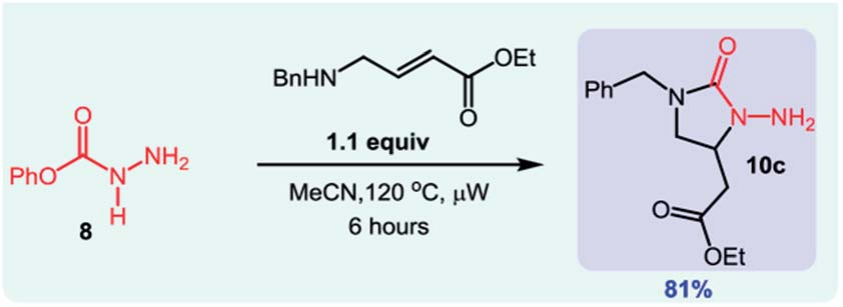




Having established the potential of *N*-isocyanates to form saturated heterocycles, amino-hydantoins and imidazolidinones, we sought to develop reaction cascades forming aromatic heterocyclic compounds possessing the N–N–C=O motif. One could expect that the aromaticity of the product should prove advantageous by either facilitating the cyclization event or simply by forming stable products that do not interfere with the cascade reaction. However, this strategy also inherently implied the use of precursors at a higher oxidation state, with the unsaturations required for aromatization being present in their structure.

**Scheme 4:**
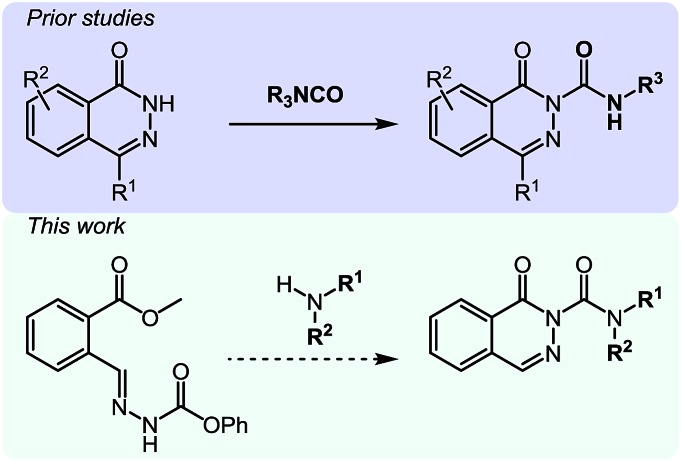
Comparison of existing method *versus N*-isocyanate synthesis of substituted phthalazinones.

For the first heteroaromatic synthesis, we wanted to build on our work on amino-esters, and explore the formation of carbamoyl-substituted phthalazinones using suitable ester-containing starting materials (Scheme 4). Only few syntheses of this biologically-active core^17^ have been reported. Indeed, a common method17*d* to synthesize functionalized phthalazinones involves the carbamoylation of the core using isocyanates. In contrast, our envisioned approach involves the formation of the phthalazinone core induced by the addition of amines onto a suitably protected *N*-isocyanate precursor (Scheme 4). The results for this strategy are presented in Table 6.

**Table 6:** Amine scope for the phthalazinones^*a*^

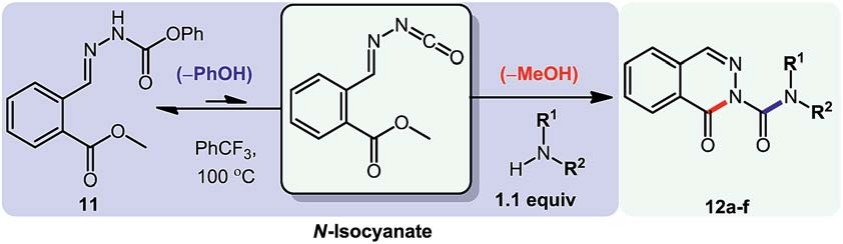			
Entry	Nucleophile	Product	Yield (%)
1 (**12a**)	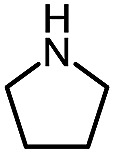	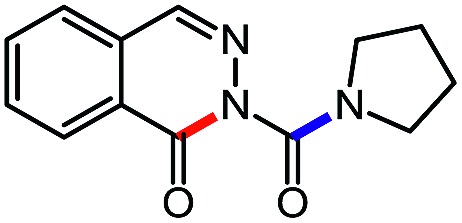	79
2 (**12b**)	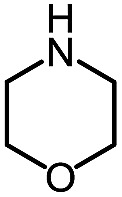	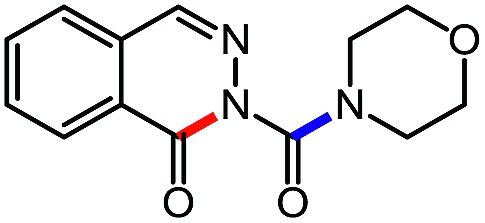	87
3 (**12c**)	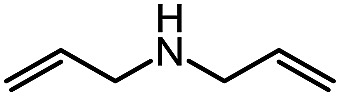	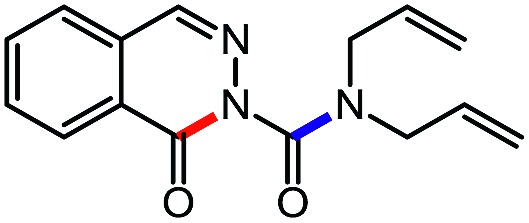	97
4 (**12d**)	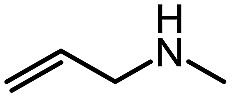	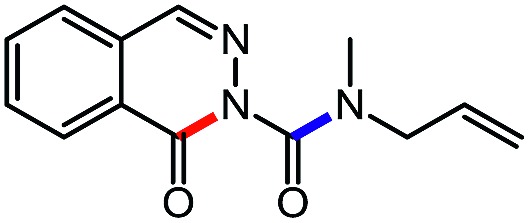	99
5 (**12e**)	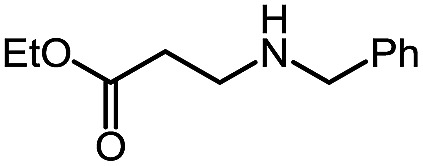	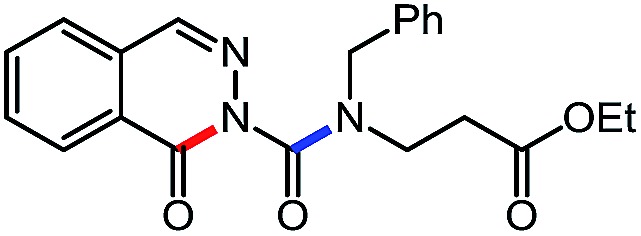	90
6 (**12f**)	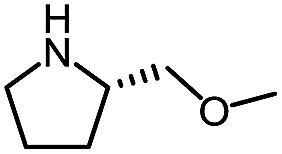	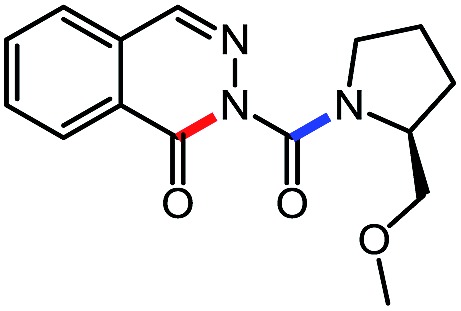	65

^*a*^Conditions: carbazone ester (1 equiv.), amine (1.1 equiv.) in PhCF_3_ (0.3 M) heated in a sealed vial (oil bath, 100 °C, 18 h for cyclic amine or 48 h for acyclic amine).

Pleasingly, optimization provided the desired cascade reaction, and carbamoyl-substituted phthalazinones were formed with several secondary amines at 100 °C. The reaction was tolerant of secondary cyclic amines such as pyrrolidine (entry 1, **12a**), morpholine (entry 2, **12b**) and an ether-containing proline derivative (entry 6, **12f**) yielding the desired heterocycle efficiently after heating for 18 h. Cascade reactions of secondary acyclic amines required prolonged heating (48 h) but yielded the corresponding phthalazinones in almost quantitative yields (entries 3–5, **12c–e**). Symmetrical and unsymmetrical amines afforded the desired products, but mixtures of semi-carbazide rotamers were observed by ^1^H NMR for the adducts of unsymmetrical amines. Unfortunately, all attempts to use primary amine nucleophiles resulted in the free N–H phthalazinone. This observation strongly suggested that the desired product was formed, but then acted as a blocked *C*-isocyanate precursor by forming the isocyanate upon thermal extrusion of N–H phthalazinone. Nevertheless, despite being limited to secondary amines this cascade provided us with the first cascade reaction forming a heteroaromatic core using *N*-isocyanate intermediates. The hydantoin and phthalazinone work showcased the cyclization potential of carbazone-derived *N*-isocyanates on esters.

**Scheme 5:**
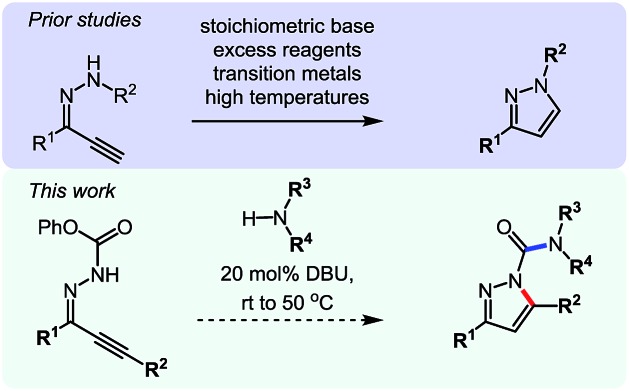
Comparison of existing method *versus N*-isocyanate synthesis of substituted pyrazoles.

To continue our studies, we wanted to expand the reactivity of *N*-isocyanates to encompass other types of cyclization reactions. With a variety of cyclization protocols, an array of heteroaromatic cores could easily be accessed. The pyrazole core came as an obvious synthetic target due to its presence in several pharmaceuticals and agrochemicals.^18^ Considerable effort has been dedicated to the synthesis of pyrazoles and several efficient strategies exist. For example, intramolecular cyclizations furnishing the pyrazole core from alkynylcarbazones^19^ are often carried out in the presence of stoichiometric base. These basic conditions are likely necessary to access a facile 5-*endo*-dig anionic cyclization pathway. It was envisioned that we could use milder conditions and assemble a library of carbamoyl-substituted pyrazoles using a cascade reaction. Such acyl pyrazoles are somewhat scarce in the literature, but have shown to be both bioactive compounds,^20^ for example in the core of the agrochemical Dimetilan, and useful building blocks^21^ (Scheme 5). The conditions and scope of this cascade reaction are presented in Table 7.

**Table 7:** Scope of a cascade reaction for the formation of acyl-pyrazoles^*a*^

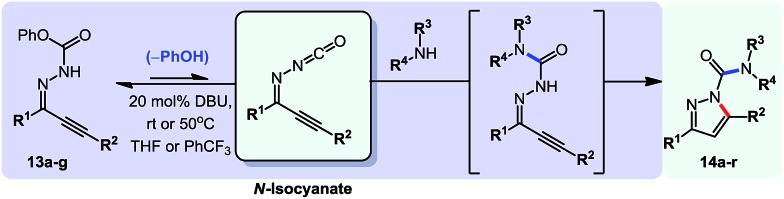
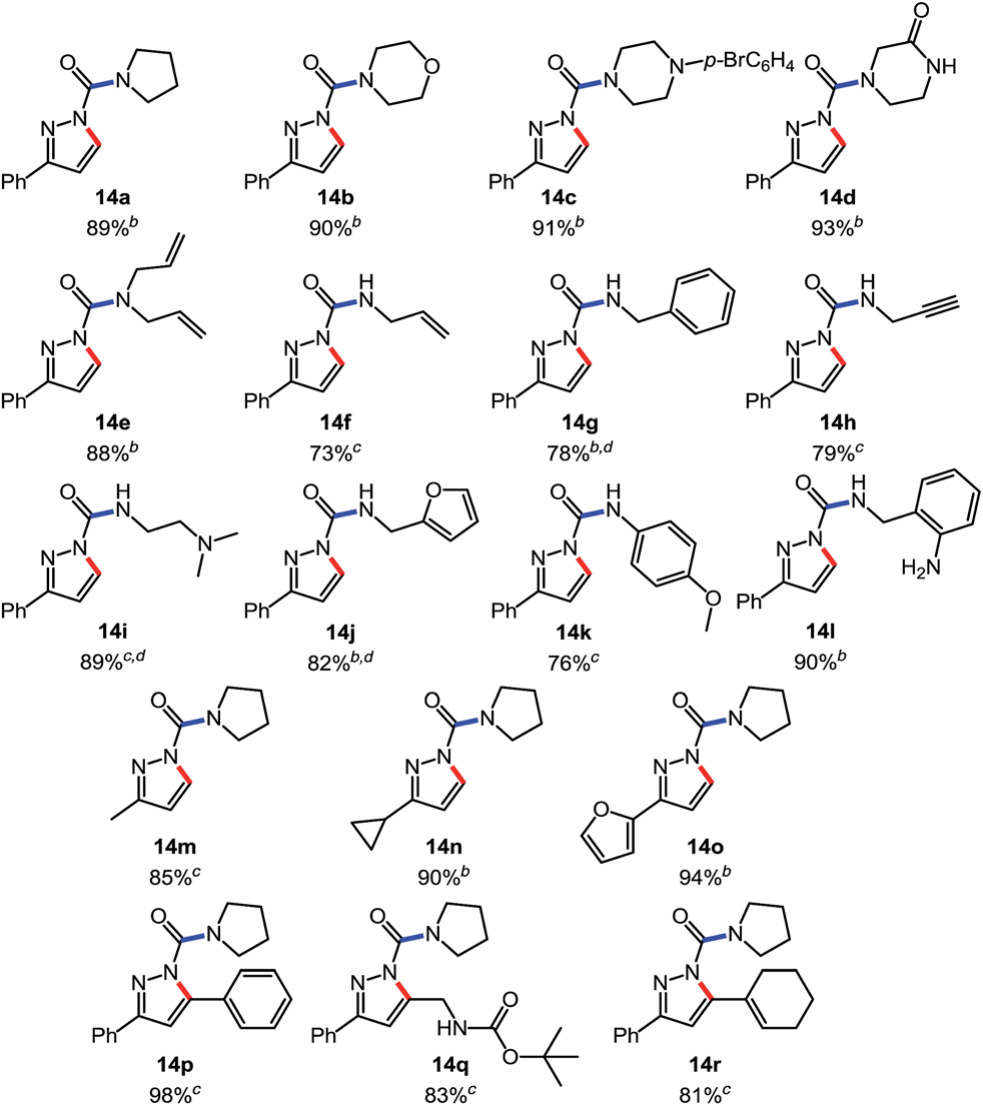

^*a*^Conditions: alkynyl carbazone (1 equiv.), amine (1.1 equiv.), DBU (20 mol%) in THF at room temperature or 50 °C for 16 h.

^*b*^Reaction at room temperature.

^*c*^Reaction at 50 °C.

^*d*^Reaction conducted in PhCF_3_.

Gratifyingly, the *N*-isocyanate addition/alkyne annulation cascade allowed the formation of a variety of pyrazoles using primary and secondary amines (Table 7).^22^ A wide variety of amines proved to be competent partners in this reaction (Table 7, top). Cyclic amines including pyrrolidine (**14a**), morpholine (**14b**) and piperazine derivatives (**14c** and **d**) all cyclized in high yield. A halogen substituted aromatic group was tolerated in the reaction (**14c**), which highlights the possibility of further functionalization. Acyclic amines were also good reaction partners for the synthesis of acyl pyrazoles (**14e–l**). Secondary amines yield the desired heteroaromatic core in high yield at room temperature. In contrast, primary amines (**14f**, **h**, **i**) required gentle heating at 50 °C but also provided the desired pyrazoles in good yield. Interestingly, benzylamine (**14g**) and furfurylamine (**14j**) did not require higher temperature to form the corresponding pyrazoles. Anilines could also be used as nucleophiles (**14k**). Given the low nucleophilicity of anilines, their use in this cascade reaction at 50 °C again supports the formation of a reactive *N*-isocyanate intermediate as the participating electrophile. The reaction could also be highly chemoselective for the most nucleophilic amine when using diamines, as demonstrated by the selective formation of adduct **14l**. In parallel to efforts using different amines the cascade reaction was also performed with several carbazones, using pyrrolidine as a representative nucleophile (Table 7, bottom). The impact of varying the carbazone (R^1^) substituent on the outcome of the cascade reaction proved minimal. Products containing both small (**14m**, R^1^ = Me) and large (**14o**, R^1^ = furyl) substituents were formed in high yield. The result obtained with the methyl-substituted carbazone (84% yield, 50 °C, 24 h) was especially noteworthy. *Indeed*, *both E and Z isomers of the carbazone starting material were present*, *favoring* (*ca.* 9 : 1 by ^1^H NMR) the *E* isomer which was not the appropriate configuration to cyclize. Thus the high yield supports that carbazone or imino-isocyanate isomerization occurred under the reaction conditions to form the *Z*-isomer required for cyclization on the alkyne. Alkyne substitution (R^2^) was also well tolerated and allows the formation of 1,3,5-trisubsituted pyrazoles under similar conditions (**14p–r**). Finally, it should be noted that product formation for these 1,3,5-trisubtituted entries was observed at room temperature but that yields were typically higher at 50 °C, and that this cascade reaction is also scalable (eqn (8)).
8

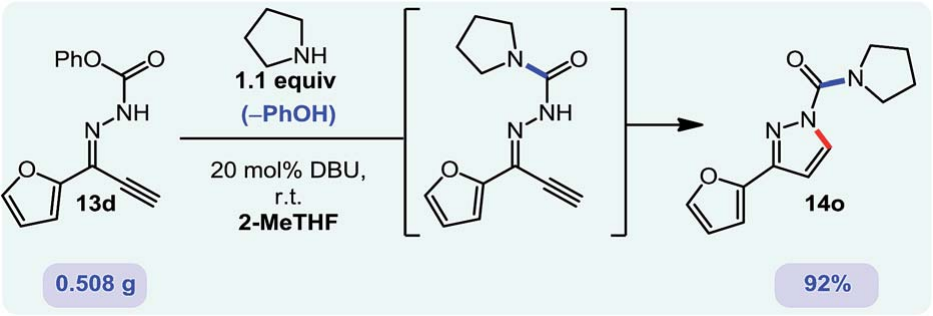




It was imperative to use basic conditions for the formation of these acyl pyrazoles due to the labile nature of products formed with primary amines. Indeed, attempts to form acyl pyrazoles upon heating in the absence of base led to formation of free N–H pyrazoles, since the acyl-pyrazoles products **14** (R^4^ = H) are known to be blocked (masked) *C*-isocyanate precursors.^2^ Fortuitously, using the DBU-catalyzed procedure the products formed would not subsequently decompose. To ensure stability during product isolation, Et_3_N-treated silica gel was also needed, suggesting that mildly acidic conditions could promote isocyanate formation. Overall, this data illustrated the usefulness of milder conditions for the development of new reaction cascades.

After showing that *N*-isocyanates could engage in cascade reactions forming 5-membered and 6-membered aromatic heterocycles, we sought to develop a cascade in which the amine nucleophile would be incorporated within the aromatic heterocycle formed. Strategically, this represented the most difficult cascade reaction targeted with *N*-isocyanates. After surveying potential scaffolds that could be obtained using this approach, the 6-azauracil ring system stood out as an excellent synthetic target due to reported biological activities^23^ as well as a lack of efficient syntheses for several substitution patterns. Interestingly, several 6-azauracil derivatives have been used for decades as pharmaceuticals and agrochemicals, and reports document their use as anticoccidials,23i–l,n,o thyroid hormone receptor agonists,23b–k CTSK inhibitors,23*d* GNRHR antagonists,23*c* P2X_7_ receptor antagonists,23*f* and 5-HT_1A_ receptor agonists.23a–j Despite the importance of this motif, we could not find cascade reactions allowing the facile generation of libraries of complex 6-azauracil compounds. Instead, most syntheses relied on the functionalization of the commercially available core structure, resulting in limitations in the substituents that could be included on the ring system (such as at the 3 position for example). To build on the reactivity previously described and exploit the ability of *N*-isocyanates to readily form semi-carbazones, we envisioned the use of carbazones derived from α-keto-esters.

As illustrated in eqn (9), using this approach would provide the ability to incorporate the primary amine reagent at the 3 position of the azauracil compounds, upon cyclization of the incoming-nitrogen atom on the ester group of the parent carbazone.
9

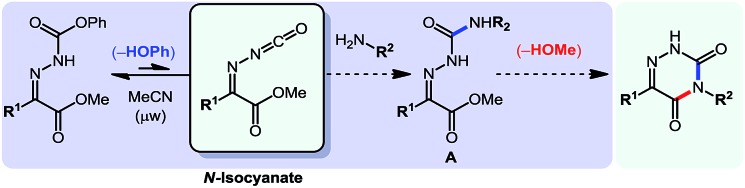




Initially, we were confident about the ability to access semi-carbazone **A** under mild conditions. We believed that both *E* and *Z* isomers of **A** would be in equilibrium thus allowing for complete conversion to the stable aromatic product. However, we expected a strong conformational preference for this intermediate that would make the cyclization step difficult, noting that related cyclizations (R^2^ = H) typically only proceed at high temperatures.^24^ Indeed, during reaction optimization only *N*-isocyanate addition products (semi-carbazone **A**) were observed at temperatures below 150 °C. However cyclization was typically observed around 150 °C, and further optimization showed that the desired azauracils formed in good yields upon heating at 175 °C. With these conditions in hand, we explored the scope of this cascade reaction: the results are displayed in Table 8.

**Table 8:** Cascade synthesis of 6-azauracil derivatives^*a*^

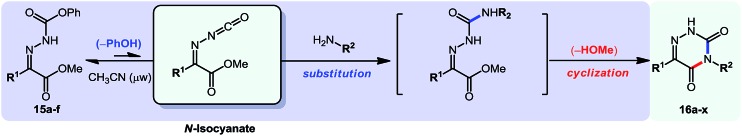
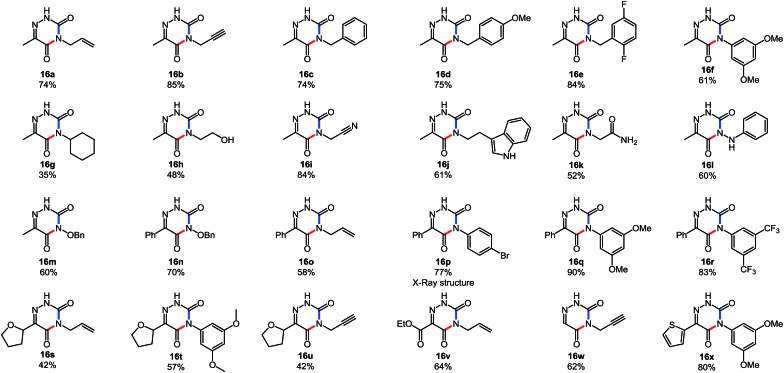

^*a*^Conditions: carbazone ester (1 equiv.), amine (1.1 equiv.) in MeCN (0.3 M) heated in a sealed vial microwave reactor, 175 °C, 6 h.

Fortunately, the cascade reaction forms a variety of substituted 6-azauracils effectively (Table 8). First, the scope of the amine partner was surveyed using pyruvate-derived carbazone **15a** (R^1^ = Me). We were pleased that a variety of primary amines, a hydrazine and a hydroxylamine could form azauracil products in moderate to high yield (35–85%). The reaction tolerated the use of hindered amines such as cyclohexylamine (**16g**, slower cyclization, 35% yield), of less nucleophilic amines such as anilines (**16f**), and of primary amines with a proximal electron-withdrawing group (**16i**, **16k**). While conducting the reaction with 4-bromoaniline, a crystalline product (**16p**) was obtained in 77% yield, and X-ray analysis secured the structural assignment (see ESI† for details). The use of anilines was also encouraging since such products inherently face chemoselectivity issues in alternative syntheses relying on the arylation of the azauracil core.^25^ Next, we investigated the incorporation of heteroatom substituents at the 3 position. Gratifyingly the reaction with phenylhydrazine resulted in 60% of the cyclized product, along with 30% of the uncyclized adduct. The reaction with *O*-benzyl hydroxylamine allowed the formation of oxygen-substituted products **16m** and **16n**; surprisingly such derivatives had not been reported in the azauracil literature. We also tested the ability of the cascade reaction to proceed with different substituents at the R^1^ position. This substitution was well tolerated, as shown by the formation of azauracil products possessing a simple hydrogen (**16w**), an ester (**16v**) and a tetrahydrofuryl (**16t**, **u**) group at the R^1^ position. These substitution patterns have medicinal relevance, for example product **16u** is a *C*-linked nucleoside analogue. Finally, this cascade tolerated a diverse set of functional groups, including a free hydroxyl (**16h**), allyl (**16o**) and propargyl (**16b**) groups, a nitrile (**16i**), ethers (**16t** and **16u**), an ester (**16v**), heteroaromatic rings such as thiophene (**16x**) and N–H indole (**16j**), a free amide (**16k**), and aromatic bromides (**16p**) and fluorides (**16e**). Collectively, these results highlight that this cascade reaction has broad applicability to rapidly assemble 6-azauracil compounds.

**Scheme 6:**
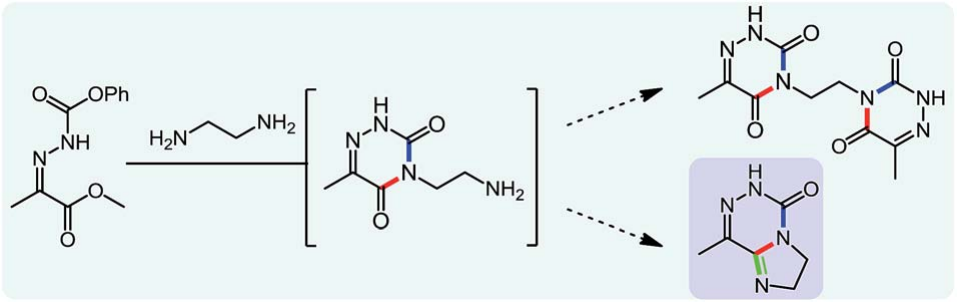
Cascade reactions forming 6-azauracils: possible divergent reactivity of diamines.

While studying the scope of azauracil formation, we wondered what would occur if a diamine was used as a nucleophile. We hypothesized that the second nitrogen atom could participate in the formation of a second heterocycle *via* an intramolecular condensation, rather than form a bis-azauracil through cyclization of each nitrogen atom (Scheme 6). To test this hypothesis, we used 2-aminoaniline and were quite pleased to observe formation of tricyclic product **17a** in 67% yield.^26^ The structure of **17a** was secured using X-ray analysis (see ESI† for details). Following this encouraging lead result, we explored the scope of this reaction with selected substrates and diamines (Table 9).

**Table 9:** Assembly of bicyclic heterocycles using an *N*-isocyanate reaction cascade^*a*^

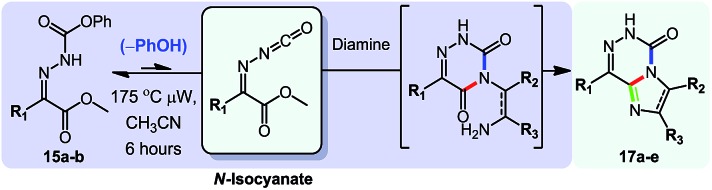			
Entry	Nucleophile	Product	Yield (%)
1 (**17a**)	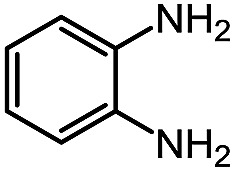	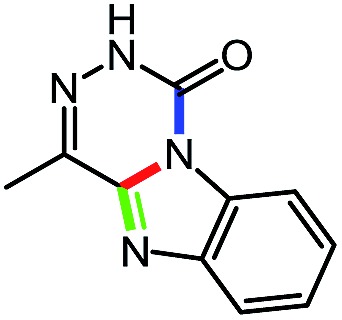	67
2 (**17b**)	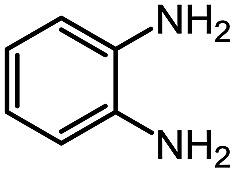	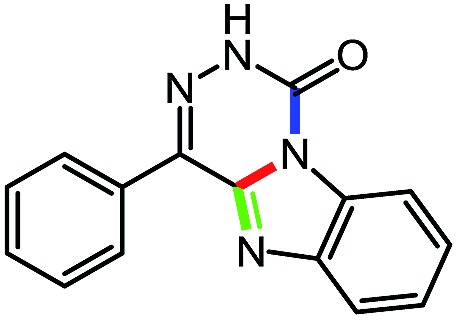	76
3 (**17c**)	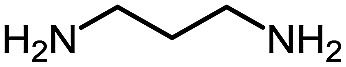	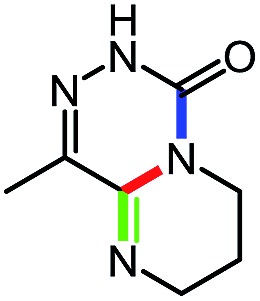	64
4 (**17d**)	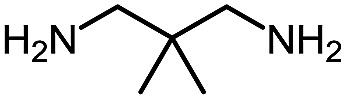	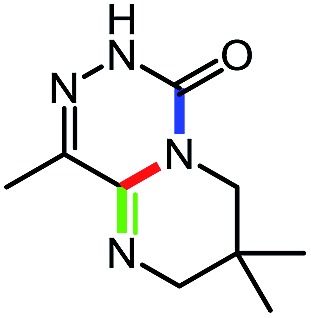	72
5 (**17e**)	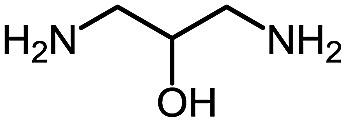	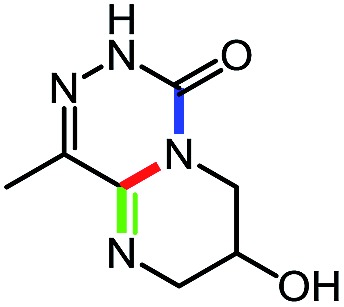	55

^*a*^Conditions: carbazone ester (1 equiv.), diamine (1.1 equiv.) in MeCN (0.3 M) heated in a sealed vial (microwave reactor, 175 °C, 6 h).

Encouragingly, several diamines engaged in a cascade reaction forming bicyclic or tricyclic systems. Relatively high yields (55–76%) were obtained considering that product formation involves *N*-isocyanate formation, addition on the *N*-isocyanate, cyclization to form the azauracil ring, and a second cyclization to form the bi- or tri-cyclic ring system. In practice, the reaction was experimentally simple since most products (entries 1, 2 and 5) precipitated out of the reaction upon heating in acetonitrile, and cooling at the end of the reaction. In addition to encouraging results to form tricyclic systems using 1,2-aminoaniline (entries 1 and 2), we were pleased that 1,3-diaminopropanes yielded the corresponding 6,6-bicyclic compounds in good yields (entries 3–5). Surprisingly, this ring system had not been described in the literature, despite decades of work on the synthesis of purine analogues,^26^^*c*^ further highlighting that cascade reactions of *N*-isocyanates can provide access to new heterocycles through simple reaction sequences.

## Conclusions

In summary, we have demonstrated that despite their amphoteric nature and reported propensity to dimerize, *N*-isocyanates are powerful intermediates in heterocyclic chemistry. Our data shows that the use of *N*-isocyanates provides a versatile strategy to assemble heterocyclic compounds possessing N–N–C=O motifs, which are common in agrochemicals and pharmaceuticals. Various heterocycles could be assembled by taking advantage of the controlled reactivity provided by the use of blocked (masked) precursors that reversibly form the desired *N*-isocyanates upon heating or in the presence of catalytic bases such as DBU. This reactivity also demonstrated the ability of different *N*-isocyanates—amino-, imino-, and amido-isocyanates—to engage in cascade reactions and allowed a comparison of their reactivity. We also demonstrated the use of *O*-phenyl carbazate as a precursor for the simplest *N*-isocyanate, NH_2_–NCO. Over *100* new heterocyclic products were formed using new reaction cascades, including new heterocycles and heterocyclic products with substitution patterns that are either difficult to prepare or that have not been reported in the literature. Beyond providing a new tool in heterocyclic chemistry, this work addresses an important void in the isocyanate literature: the lack of reactions exploiting the reactivity of *N*-isocyanates. This scarcity is surprising considering that the applications of *C*-substituted isocyanates are extremely well developed. We hope that this first thorough study on the synthetic uses of *N*-isocyanates will encourage others to develop reactions of *N*-isocyanates, also taking advantage of the blocked *N*-isocyanate approach to overcome dimerization. Efforts along these lines are ongoing in our laboratories and will be reported in due course.

## Supplementary Material

Supplementary information

Crystal structure data
