# Supplementary material for: Cascade reactions of nitrogen-substituted isocyanates: a new tool in heterocyclic chemistry
Source: Chem Sci. 2015 Sep 23;7(1):315–28. doi: 10.1039/c5sc03197d (PMC5952554; doi:10.1039/c5sc03197d)
Supplement: Supplementary file 1 [file SC-007-C5SC03197D-s001.pdf]

Electronic Supplementary Material (ESI) for Chemical Science.
This journal is © The Royal Society of Chemistry 2015

Cascade Reactions of Nitrogen-Substituted Isocyanates: A New Tool in Heterocyclic Chemistry

Jean-François Vincent-Rocan, Ryan A. Ivanovich, Christian Clavette, Kyle Leckett, Julien Bejjani, André M. Beauchemin*

Centre for Catalysis Research and Innovation, Department of Chemistry and Biomolecular Sciences, University of Ottawa. 10 Marie-Curie, Ottawa, ON, K1N 6N5, Canada.

Electronic Supplementary information

Table of Contents

General Information **2**

Materials **2**

Substitution-Hydroamination Cascade (Tables 1-2) **2**

Cascade Synthesis of *N*-Substituted Hydantoins (Tables 3-4) **3**

Phenyl Carbazate Substitution (Table 5) **3**

Procedures for Equations 5-7 **7**

Phthalazinones (Table 6) **8**

Pyrazoles (Tables 7) **11**

Other Attempts to form Pyrazoles (Not Shown in Table 7) **23**

Azauracils (Tables 8-9) **25**

References **40**

Spectra **41**

General Information

Purification of reaction products was carried out by flash column chromatography using Silicycle silica gel (40-63 μm), unless otherwise noted. Analytical thin layer chromatography (TLC) was performed on aluminum, cut to size. Visualization was accomplished with UV light followed by staining with a potassium permanganate solution and heating.

^{1}H NMR and ^{13}C NMR spectra were recorded on Bruker AVANCE 300 MHz and 400 MHz spectrometers at ambient temperature, unless otherwise indicated. Spectral data was reported in ppm using solvent as the reference ($CDCl_3$ at 7.26 ppm, C_6D_6 at 7.15 ppm, or DMSO-d_6 at 2.50 ppm for ^{1}H NMR and $CDCl_3$ at 77.0 ppm or DMSO-d_6 at 39.43 for ^{13}C NMR). ^{1}H NMR data was reported as: multiplicity (br = broad, s = singlet, d = doublet, t = triplet, q = quartet, quint. = quintet, sext. = sextuplet, sept. = septuplet, m = multiplet), integration, and coupling constant(s) in Hz. Infrared (IR) spectra were obtained with neat thin films on a sodium chloride disk and were recorded on a Bomem Michelson 100 Fourier transform infrared spectrometer (FTIR). High-resolution mass spectroscopy (HRMS) was performed on a Kratos Concept-11A mass spectrometer with an electron beam of 70 eV at the Ottawa-Carleton Mass Spectrometry Centre.

Materials

Unless otherwise noted, all commercially available materials were purchased from commercial sources and used without further purification.

Substitution-Hydroamination Cascade (Tables 1-2)

Detailed experimental procedures and characterization data has been published and can be obtained in the supporting information of a previous communication from our group.[7] A representative procedure is included below.

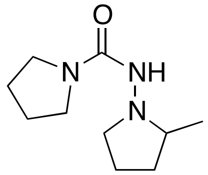

N-(2-Methylpyrrolidin-1-yl)pyrrolidine-1-carboxamide (Table 1, 2a): An oven dried 5 mL microwave tube was charged with a stir bar, capped with a septum and purged with argon and an outlet for 5 minutes. Phenyl 2-(pent-4-en-1-yl)hydrazinecarboxylate (0.197 g, 0.894 mmol), pyrrolidine (0.0699 g, 0.984 mmol), and α,α,α-trifluorotoluene (3.0 mL) were added to the seal tube, while keeping it under an argon atmosphere. The septum was removed and the tube was then quickly sealed with a microwave cap and heated for six hours at 120 °C under microwave irradiation. The tube was cooled to ambient temperature, volatiles were removed under reduced pressure and the title compound was purified by column chromatography (4% MeOH/CH_2Cl_2)

and obtained as a white solid (0.155 g, 88% isolated yield, 88% NMR yield). TLC R_f = 0.25 in 4% $MeOH/CH_2Cl_2$. ^{1}H NMR (300 MHz; $CDCl_3$) δ 4.90 (s, br, 1H), 3.39-3.25 (m, 5H), 2.67 (dquintet, *J* = 9.2, 6.4 Hz, 1H), 2.55 (q, *J* = 8.8 Hz, 1H), 1.96-1.64 (m, 7H), 1.44 (dddd, *J* = 12.1, 10.2, 9.4, 6.5 Hz, 1H), 1.11 (d, *J* = 6.1 Hz, 3H); ^{13}C NMR (100 MHz; $CDCl_3$) 157.3, 62.1 (CH), 56.3 (CH_2), 46.1 (CH_2), 30.5 (CH_2), 25.5 (CH_2), 20.2 (CH_2), 18.3 (CH_3); IR (film) 3230, 2968, 2869, 1645, 1542, 1394 cm^{-1}. HRMS (ES): Exact mass calcd for $C_{10}H_{19}N_3O$ $[M]^-$: 196.1488; found $[M]^-$: 196.0845.

Cascade Synthesis of *N*-Substituted Hydantoins (Tables 3-4)

Detailed experimental procedures and characterization data has been published and can be obtained in the supporting information of a previous communication from our group.[8] a representative procedure is included below.

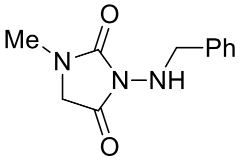

3-(Benzylamino)-1-methylimidazolidine-2,4-dione (Table 3, 5a): An oven dried microwave tube was charged with a stir bar, capped with a septum and purged with argon and an outlet for 5 minutes. The carbazate (1.0 equiv), amino-ester hydrochloride salt (1.1 equiv), *N*,*N*-diisopropylethylamine (1.2 equiv) and α,α,α-trifluorotoluene (MeCN and $MeNO_2$) (0.3 M) were added to the seal tube, while keeping it under an argon atmosphere. The septum was removed and the tube was then quickly sealed with a microwave cap and heated between three and six hours at 80-150 °C. The tube was cooled to ambient temperature, concentrated under reduced pressure and the title compound was purified by column chromatography (20% $EtOAc/CH_2Cl_2$) and was obtained as colorless amorphous solid (0.0610 g, 82 % yield). TLC R_f = 0.19 in 20% $EtOAc/CH_2Cl_2$ ^{1}H NMR (300 MHz; $CDCl_3$): δ 7.42-7.39 (m, 2H), 7.34-7.27 (m, 3H), 4.11 (s, 2H), 3.76 (s, 2H), 2.95 (s, 3H), ^{13}C NMR (75 MHz; $CDCl_3$) δ 167.2 (C), 155.4 (C), 135.7 (C), 129.0 (CH), 128.3 (CH), 127.8 (CH), 54.5 (CH_2), 50.1 (CH_2), 29.8 (CH_2), IR (film) 2982, 1772, 1716, 1440,1260, 1085. HRMS (EI): Exact mass calcd for $C_{11}H_{13}N_3O_2$ $[M]^+$: 219.1008. Found: 219.1003.

Phenyl Carbazate Substitution (Table 5)

General procedure 1: An oven-dried round bottom flask was charged with a stir bar, phenyl carbazate (1.00 equiv.), an amine (1.10 equiv.), DBU (20 mol%), and THF (0.3 M). The mixture was stirred overnight, concentrated under reduced pressure, and purified by flash column chromatography.

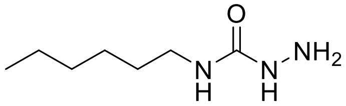

Table 5, entry 1: *N*-Hexylhydrazinecarboxamide (9a): Synthesized according to general procedure **1** using phenyl carbazate (0.152 g, 1.00 mmol), hexylamine (0.15 mL, 1.1 mmol), DBU (0.0300 g, 0.200 mmol), and THF (3.3 mL). The title compound was purified by column chromatography (5% CH_3OH/CH_2Cl_2) to yield a white solid (0.132 g, 83%). TLC Rf = 0.33 in 5% CH_3OH/CH_2Cl_2. ^1H NMR (300 MHz; $CDCl_3$): δ 6.88 (br s, 1H), 6.08 (br s, 1H), 3.66 (br s, 2H), 3.16 (dt, J = 7.1, 6.2 Hz, 2H), 1.47 (m, 2H), 1.26 (m, 6H), 0.85 (m, 3H). ^{13}C NMR (100 MHz; $CDCl_3$): δ 160.8 (C), 39.5 (CH_2), 31.4 (CH_2), 30.1 (CH_2), 26.4 (CH_2), 22.4 (CH_2), 13.9 (CH_3). IR (film): 3340, 2926, 2860, 2359, 1661, 1622, 1548, 1463, 1375, 1160 cm^{-1}. HRMS (EI): Exact mass calcd for $C_7H_{17}N_3O$ $[M]^+$: 159.1372. Found: 159.13553.

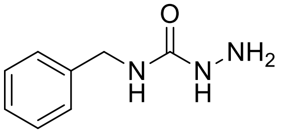

Table 5, entry 2: *N*-Benzylhydrazinecarboxamide (9b): Synthesized according to general procedure **1** using phenyl carbazate (0.152 g, 1.00 mmol), benzylamine (0.12 mL, 1.1 mmol), DBU (0.0300 g, 0.200 mmol), and THF (3.3 mL). The title compound was purified by column chromatography (8% CH_3OH/CH_2Cl_2) to yield an amorphous white solid (0.127 g, 77%). TLC Rf = 0.40 in 8% CH_3OH/CH_2Cl_2. ^1H NMR (300 MHz; $CDCl_3$): δ 7.29 (m, 5H), 6.96 (br s, 1H), 6.48 (br s, 1H), 4.40 (d, J = 6.1 Hz, 2H), 3.62 (br s, 2H). ^{13}C NMR (100 MHz; $CDCl_3$): δ 160.7 (C), 139.4 (C), 128.4 (CH), 127.2 (CH), 127.0 (CH), 43.3 (CH_2). IR (film): 3339, 3300, 3194, 2357, 1618, 1555, 1468, 1452, 1265 cm^{-1}. HRMS (EI): Exact mass calcd for $C_8H_{11}N_3O$ $[M]^+$: 165.0902. Found: 165.09032.

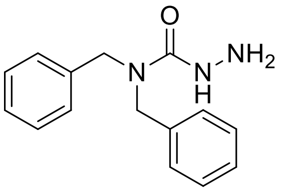

Table 5, entry 3: *N,N*-Dibenzylhydrazinecarboxamide (9c): Synthesized according to general procedure **1** using phenyl carbazate (0.152 g, 1.00 mmol), *N,N*-dibenzylamine (0.21 mL, 1.1 mmol), DBU (0.0300 g, 0.200 mmol), and THF (3.3 mL). The title compound was purified by column chromatography (3% CH_3OH/CH_2Cl_2) to yield an amorphous yellow solid (0.240 g, 94%). TLC Rf = 0.31 in 3% CH_3OH/CH_2Cl_2. ^1H NMR (300 MHz; $CDCl_3$): δ 7.30 (m, 10H), 6.22 (br s, 1H), 4.47 (s, 4H), 3.83 (s, 2H). ^{13}C NMR (100 MHz; $CDCl_3$): δ 160.7 (C), 140.1 (C), 136.9 (C), 128.7 (CH), 128.2 (CH), 127.9 (CH), 127.4 (CH), 127.0 (CH), 126.7 (CH), 52.9 (CH_2), 49.8 (CH_2). IR (film): 3317, 3028, 2359, 2336, 1616, 1494, 1452, 1400, 1364, 1261 cm^{-1}. HRMS (EI): Exact mass calcd for $C_{15}H_{17}N_3O$ $[M]^+$: 255.1372. Found: 255.13965.

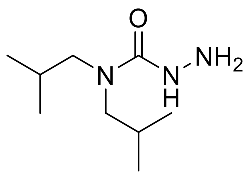

Table 5, entry 4: *N,N*-Diisobutylhydrazinecarboxamide (9d): Synthesized according to general procedure **1** using phenyl carbazate (0.152 g, 1.00 mmol), *N,N*-diisobutylamine (0.19 mL, 1.1 mmol), DBU (0.0300 g, 0.200 mmol), and THF (3.3 mL). The title compound was purified by column chromatography (3% CH_3OH/CH_2Cl_2) to yield an amorphous yellow solid (0.142 g, 76%). TLC Rf = 0.15 in 5% CH_3OH/CH_2Cl_2. ^{1}H NMR (300 MHz; $CDCl_3$): δ 5.75 (br s, 1 H), 3.80 (br s, 2H), 3.02 (d, *J* = 7.5 Hz, 4H), 1.95 (dquint., *J* = 13.8, 6.9 Hz, 2H), 0.88 (d, *J* = 6.7 Hz, 12H). ^{13}C NMR (100 MHz; $CDCl_3$): δ 160.5 (C), 55.2 (CH_2), 27.4 (CH_3), 20.1 (CH). IR (film): 2962, 1713, 1666, 1652, 1616, 1599, 1491, 1265, 1202, 1130 cm^{-1}. HRMS (EI): Exact mass calcd for $C_9H_{21}N_3O$ $[M]^+$: 187.1685. Found: 187.1684.

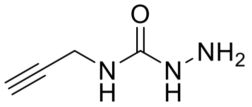

Table 5, entry 5: *N*-Propargylhydrazinecarboxamide (9e): Synthesized according to general procedure **1** using phenyl carbazate (0.152 g, 1.00 mmol), propargylamine (0.070 mL, 1.1 mmol), DBU (0.0300 g, 0.200 mmol), and THF (3.3 mL). The title compound was purified by column chromatography (5% CH_3OH/CH_2Cl_2) to yield an amorphous white solid (0.0600 g, 54%). TLC Rf = 0.13 in 5% CH_3OH/CH_2Cl_2. ^{1}H NMR (300 MHz; DMSO-d_6): δ 7.11 (br s, 1 H), 6.60 (br s, 1H), 4.11 (br s, 2H), 3.80 (dd, *J* = 7.1, 2.5 Hz, 2H), 3.01 (t, *J* = 2.5 Hz, 1H). ^{13}C NMR (100 MHz; DMSO-d_6): δ 159.7 (C), 82.7 (C), 72.2 (CH), 28.5 (CH_2). IR (film): 2350, 1710, 1551, 1357, 1337, 1263, 1040 cm^{-1}. HRMS (EI): Exact mass calcd for $C_4H_7N_3O$ $[M]^+$: 113.0589. Not found. LRMS m/z (relative intensity): 57.1 (7.8%), 55.1(4.6 %), 39.0 (28.4%), 32.0 (73.6%), 32.0 (37.4%), 31.0 (8.1 %), 31.0 (5.3%), 28.0 (100%).

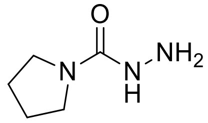

Table 5, entry 6: Pyrrolidine-1-carbohydrazide (9f): Synthesized according to general procedure **1** using phenyl carbazate (0.152 g, 1.00 mmol), pyrrolidine (0.090 mL, 1.1 mmol), DBU (0.0300 g, 0.200 mmol), and THF (3.3 mL). The title compound was purified by column chromatography (8% CH_3OH/CH_2Cl_2) to yield a yellow oil (0.0940 g, 73%). TLC Rf = 0.37 in 8% CH_3OH/CH_2Cl_2. ^{1}H NMR (300 MHz; $CDCl_3$): δ 5.94 (br s, 1H), 3.77 (br s, 2H), 3.28 (t, *J* = 6.7 Hz, 4H), 1.86 (m, 4H). ^{13}C NMR (100 MHz; $CDCl_3$): δ 158.8 (C), 45.3 (CH_2), 25.3 (CH_2). IR (film): 3387, 2978, 2872, 2359, 1616, 1504, 1377 cm^{-1}. HRMS (EI): Exact mass calcd for $C_5H_{11}N_3O$ $[M]^+$: 129.0902. Found: 129.08920.

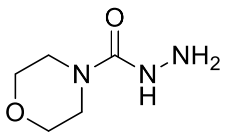

Table 5, entry 7: Morpholine-4-carbohydrazide (9g): Synthesized according to general procedure **1** using phenyl carbazate (0.152 g, 1.00 mmol), morpholine (0.095 mL, 1.1 mmol), DBU (0.0300 g, 0.200 mmol), and THF (3.3 mL). The title compound was obtained by filtration of the solution to yield a white amorphous solid (0.122 g, 84%). TLC Rf = 0.27 in 8% CH_3OH/CH_2Cl_2. ^{1}H NMR (300 MHz; $CDCl_3$): δ 6.49 (br s, 1H), 3.83 (br s, 2H), 3.64 (t, *J* = 4.7 Hz, 4H), 3.33 (t, *J* = 5.1 Hz, 4H). ^{13}C NMR (100 MHz; $CDCl_3$): δ 160.0 (C), 66.3 (CH_2), 43.6 (CH_2). IR (film): 3344, 2862, 1612, 1506, 1398, 1305, 1269, 1113 cm^{-1}. HRMS (EI): Exact mass calcd for $C_5H_{11}N_3O_2$ $[M]^+$: 145.0851. Found: 145.08383.

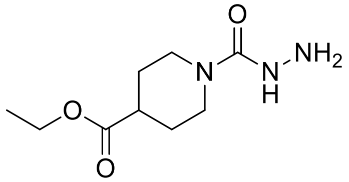

Table 5, entry 8: 1-Hydrazinocarbonyl-piperidine-4-carboxylic acid ethyl ester (9h): Synthesized according to general procedure **1** using phenyl carbazate (0.152 g, 1.00 mmol), ethyl isonipecotate (0.173 g, 1.10 mmol), DBU (0.0300 g, 0.200 mmol), and THF (3.3 mL). The title compound was purified by column chromatography (5% CH_3OH/CH_2Cl_2) to yield a white amorphous solid (0.183 g, 85%). TLC Rf = 0.20 in 5% CH_3OH/CH_2Cl_2. ^{1}H NMR (300 MHz; $CDCl_3$): δ 5.92 (br s, 1H), 4.19-4.09 (m, 2H), 3.93-3.77 (m, 2H), 3.67-3.32 (m, 2H), 2.98-2.85 (m, 2H), 2.55-2.41 (m, 1H), 1.96-1.84 (m, 2H), 1.74-1.59 (m, 2H), 1.28-1.18 (m, 3H). ^{13}C NMR (75 MHz; $CDCl_3$): δ 174.3 (C), 159.8 (C), 60.8 (CH_2), 43.2 (CH_2), 40.9 (CH), 27.8 (CH_2), 14.3 (CH_3). IR (film): 3330, 2952, 1718, 1623, 1606, 1448, 1379, 1313, 1265, 1186, 1164, 1145, 1114, 1095, 1080, 1039 cm^{-1}. HRMS (EI): Exact mass calcd for $C_9H_{17}N_3O_3$ $[M]^+$: 215.1270. Found: 215.1264.

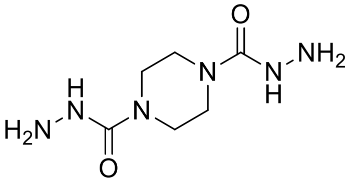

Table 5, entry 9: Piperazine-1,4-dicarbohydrazide (9i): Synthesized according to general procedure **1** using phenylcarbazate (0.152 g, 1.00 mmol), ethyl isonipecotate (0.0430 g, 0.500 mmol), DBU (0.0300 g, 0.200 mmol), and THF (3.3 mL). The title compound precipitated out of solution to afford the pure compound as an amorphous white solid (0.0930 g, 91%). ^{1}H NMR (300 MHz; DMSO-*d*$_6$): δ 7.38 (br s, 2H), 3.72 (br s, 4H), 3.24 (m, 8H). ^{13}C NMR (75 MHz; DMSO-*d*$_6$): δ 159.6 (C), 43.0 (CH_2). IR (film): 3002, 1733, 1718, 1662, 1633, 1604, 1448, 1315, 1255, 1184, 1164, 1039 cm^{-1}. HRMS (EI): Exact mass calcd for $C_{16}H_{12}N_2O_2$ $[M]^+$: 202.1178. Found: 202.1204.

Procedures for Equations 5-7

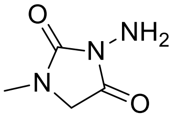

Equation 5: 3-Amino-1-methylimidazolidine-2,4-dione (10a): To a solution of phenyl carbazate (1.04 g, 6.80 mmol) in MeCN (20 mL) was added sarcosine ethyl ester hydrochloride (1.14 g, 7.40 mmol) and DIPEA (1.4 mL, 8.0 mmol) and the solution was stirred in a microwave reactor for 6 hours at 120 °C. The solution was concentrated under reduced pressure and purified by silica gel column chromatography using 8% MeOH/CH_2Cl_2 to afford the pure compound as a white amorphous solid (0.800 g, 91%). TLC Rf = 0.29 in 8% MeOH/CH_2Cl_2. ^{1}H NMR (300 MHz; $CDCl_3$): δ 3.89 (s, 2H), 3.73 (br s, 2H), 3.03 (s, 3H). ^{13}C NMR (75 MHz; $CDCl_3$): δ 167.9 (C), 155.9 (C), 50.2 (CH_2), 29.9 (CH_3). IR (film): 1782, 1717, 1479, 1448, 1418, 1391, 1265, 1242 cm^{-1}. HRMS (EI): Exact mass calcd for $C_4H_7N_3O_2$ $[M]^+$: 129.0538. Found: 129.0536.

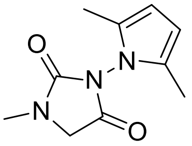

Equation 6: 3-(2,5-Dimethyl-1H-pyrrol-1-yl)-1-methylimidazolidine-2,4-dione (10b): To a solution of amino hydantoin **10a** (0.130 g, 1.00 mmol) in PhMe (4.0 mL) was added hexanedione (0.114 g, 1.00 mmol) and PTSA monohydrate (0.0200 g, 8.00 mmol) and the solution was stirred in a microwave reactor for 1 hour at 150 °C. The solution was concentrated under reduced pressure and purified by silica gel column chromatography using 10% EtOAc/CH_2Cl_2 to afford the pure compound as a white amorphous solid (0.187 g, 90%). TLC Rf = 0.54 in 10% EtOAc/CH_2Cl_2. ^{1}H NMR (300 MHz; $CDCl_3$): δ 5.88 (s, 2H), 4.07 (s, 2H), 3.07 (s, 3H), 2.06 (s, 6H). ^{13}C NMR (75 MHz; $CDCl_3$): δ 166.4 (C), 153.2 (C), 127.6 (C), 105.4 (CH), 50.2 (CH_2), 30.3 (CH_3), 10.9 (CH_3). IR (film): 2984, 1760, 1712, 1450, 1438, 1291, 1265, 1242. HRMS (EI): Exact mass calcd for $C_{10}H_{13}N_3O_2$ $[M]^+$: 207.1008. Found: 207.1003.

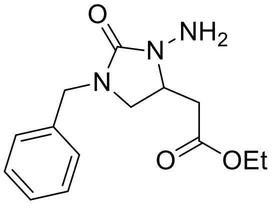

Equation 7: Ethyl 2-(3-amino-1-benzyl-2-oxoimidazolidin-4-yl)acetate (10c): To a solution of phenyl carbazate (0.0910 g, 0.600 mmol) in MeCN (2.0 mL) was added ethyl (*E*)-4-(benzylamino)-2-butenoate (0.145 g, 0.660 mmol) and the solution was stirred in a microwave reactor for 6 hours at 120 °C. The solution was concentrated under reduced pressure and purified

by silica gel column chromatography using EtOAc to afford the pure compound as a white amorphous solid (0.135 g, 81%). TLC Rf = 0.16 in EtOAc. ^{1}H NMR (300 MHz; $CDCl_3$): δ 7.36-7.24 (m, 5H), 6.31 (br s, 1H), 4.61-4.49 (m, 2H), 4.15-4.05 (m, 3H), 3.58-3.46 (m, 1H), 3.29 (dd, *J* = 11.7, 4.4 Hz, 1H), 3.07 (dd, *J* = 11.7, 8.0 Hz, 1H), 2.53-2.36 (m, 2H), 1.21 (t, *J* = 7.2 Hz, 3H). ^{13}C NMR (75 MHz; $CDCl_3$): δ 170.4 (C), 155.6 (C), 137.1 (C), 128.46 (CH), 128.0 (CH), 127.4 (CH), 60.9 (CH_2), 50.5 (CH_2), 50.4 (CH_2), 49.7 (CH), 35.4 (CH_2), 14.0 (CH_3). IR (film): 3335, 1771, 1719, 1684, 1632, 1601, 1445, 1416, 1337 cm^{-1}. HRMS (EI): Exact mass calcd for $C_{14}H_{19}N_3O_3$ $[M]^+$: 277.1426. Found: 277.1427.

Phthalazinones (Table 6)

General procedure 2: An oven-dried round bottom flask was charged with a stir bar, a carbazone ester (1.0 equiv.), an amine (1.1 equiv.), and $PhCF_3$ (0.3 M). The mixture was stirred at 100 °C for 18 or 48 hours. The reaction was cooled to ambient temperature, concentrated under reduced pressure, and purified by silica gel column chromatography to give the corresponding products.

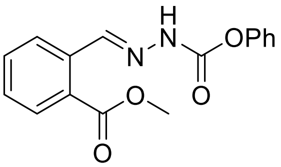

Table 6,: (*E*)-Phenyl-2-(2-(methoxycarbonyl)benzylidene)hydrazinecarboxylate (11): To a solution of phenyl carbazate (0.834 g, 5.48 mmol) in MeOH (30 mL) was added 2-methoxycarbonylbenzaldehyde (0.900 g, 5.48 mmol) and the solution was stirred at room temperature for 5 hours. The solution was condensed over reduced pressure to give a crude solid. Boiling ether was added to the solid and the pure product was collected by filtration as a white amorphous solid (1.45 g, 97%). TLC Rf = 0.12 in 10% EtOAc/CH_2Cl_2. ^{1}H NMR (300 MHz; DMSO-d_6): δ 11.90 (br s, 1H), 8.78 (br s, 1H), 7.97 (dd, *J* = 7.9, 1.3 Hz, 1H), 7.87 (dd, *J* = 7.8, 1.1 Hz, 1H), 7.65 (td, *J* = 7.6, 1.5 Hz, 1H), 7.56-7.51 (m, 1H), 7.46-7.41 (m, 2H), 7.29-7.21 (m, 3H), 3.88 (s, 3H). ^{13}C NMR (75 MHz; $CDCl_3$): δ 167.3 (C), 150.5 (C), 134.6 (C), 132.4 (CH), 130.5 (CH), 129.4 (CH), 129.3 (CH), 128.7 (CH), 127.7 (CH), 125.6 (CH), 125.0 (C), 121.4 (CH), 52.3 (CH_3). IR (film): 1715, 1684, 1564, 1489, 1477, 1435, 1266, 1202 cm^{-1}. HRMS (EI): Exact mass calcd for $C_{16}H_{14}N_2O_4$ $[M]^+$: 298.0954. Not found. Not found. Calcd for $C_{10}H_9N_2O_3$ $[M]^+$: 205.0613 Found: 205.0523 (M – OPh).

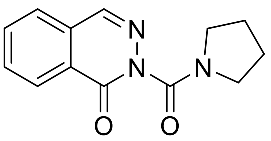

Table 6, entry 1: 2-(Pyrrolidine-1-carbonyl)phthalazin-1(2H)-one (12a): Synthesized according to general procedure **2** using carbazone ester **11** (0.179 g, 0.600 mmol), pyrrolidine (0.0470 g, 0.660 mmol), and $PhCF_3$ (2.0 mL). The crude mixture was purified by silica gel column chromatography using 20% EtOAc/CH_2Cl_2 to afford the pure compound as an

amorphous white solid (0.115 g, 79%). TLC Rf = 0.24 in 20% EtOAc/CH_2Cl_2. ^{1}H NMR (300 MHz; $CDCl_3$): δ 8.44 (dt, *J* = 7.7, 0.8 Hz, 1H), 8.23 (d, *J* = 0.5 Hz, 1H), 7.92-7.72 (m, 3H), 3.74-3.70 (m, 2H), 3.41 (t, *J* = 6.6 Hz, 2H), 2.05-1.88 (m, 4H). ^{13}C NMR (75 MHz; $CDCl_3$): δ 157.5 (C), 151.7 (C), 139.1 (CH), 134.0 (CH), 132.2 (CH), 129.6 (CH), 128.0 (C), 126.9 (C), 126.4 (CH), 41.1 (CH_2), 41.1 (CH_2), 25.5 (CH_2), 24.7 (CH_2). IR (film): 2978, 2880, 1699, 1661, 1612, 1591, 1558, 1456, 1408, 1308, 1225 cm^{-1}. HRMS (EI): Exact mass calcd for $C_{13}H_{13}N_3O_2$ $[M]^+$: 243.1008. Found: 243.1013.

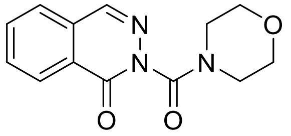

Table 6, entry 2: 2-(Morpholine-4-carbonyl)phthalazin-1(2H)-one (12b): Synthesized according to general procedure **2** using carbazone ester **11** (0.179 g, 0.600 mmol), morpholine (0.0580 g, 0.660 mmol), and $PhCF_3$ (2.0 mL). The crude mixture was purified by silica gel column chromatography using 20% EtOAc/CH_2Cl_2 to afford the pure compound as an amorphous white solid (0.135 g, 87%). TLC Rf = 0.18 in 20% EtOAc/CH_2Cl_2. ^{1}H NMR (300 MHz; $CDCl_3$): δ 8.44-8.41 (m, 1H), 8.25 (d, *J* = 0.5 Hz, 1H), 7.91-7.71 (m, 3H), 3.84 (s, 4H), 3.73-3.70 (m, 2H), 3.37-3.34 (m, 2H). ^{13}C NMR (75 MHz; $CDCl_3$): δ 158.1 (C), 152.5 (C), 139.5 (CH), 134.2 (CH), 132.4 (CH), 129.6 (C), 127.7 (C), 126.9 (CH), 126.6 (CH), 66.5 (CH_2), 66.3 (CH_2), 46.9 (CH_2), 44.4 (CH_2). IR (film): 2568, 1675, 1662, 1433, 1429, 1418, 1270, 1240, 1113 cm^{-1}. HRMS (EI): Exact mass calcd for $C_{13}H_{13}N_3O_3$ $[M]^+$: 259.0957. Found: 259.0963.

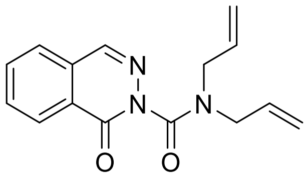

Table 6, entry 3: *N,N*-Diallyl-1-oxophthalazine-2(1H)-carboxamide (12c): Synthesized according to general procedure **2** using carbazone ester **11** (0.179 g, 0.600 mmol), diallylamine (0.0640 g, 0.660 mmol), and $PhCF_3$ (2.0 mL). The crude mixture was purified by silica gel column chromatography using 5% EtOAc/CH_2Cl_2 to afford the pure compound as a colorless oil (0.161 g, 97%). TLC Rf = 0.43 in 5% EtOAc/CH_2Cl_2. ^{1}H NMR (300 MHz; $CDCl_3$): δ 8.41-8.38 (m,1H), 8.22 (s, 1H), 7.87-7.71 (m, 3H), 5.96-5.83 (m, 1H), 5.81-5.70 (m, 1H), 5.40 (dd, *J* = 17.2, 1.3 Hz, 1H), 5.28 (dd, *J* = 10.1, 1.0 Hz, 1H), 5.13-5.06 (m, 2H), 4.17 (d, *J* = 5.5 Hz, 2H), 3.80 (d, *J* = 6.1 Hz, 2H). ^{13}C NMR (75 MHz; $CDCl_3$): δ 160.0 (C), 153.7 (C), 139.1 (CH), 134.0 (CH), 132.3 (CH), 131.2 (CH), 129.5 (C), 127.7 (C), 126.7 (CH), 126.5 (CH), 118.5 (CH_2), 118.1 (CH_2), 50.8 (CH_2), 50.0 (CH_2). IR (film): 1713, 1666, 1591, 1414, 1265, 1227, 1177 cm^{-1}. HRMS (EI): Exact mass calcd for $C_{15}H_{15}N_3O_2$ $[M]^+$: 269.1164. Found: 269.1153.

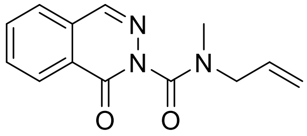

Table 6, entry 4: ***N*-Allyl-*N*-methyl-1-oxophthalazine-2(1H)-carboxamide (12d):** Synthesized according to general procedure **2** using carbazone ester **11** (0.0900 g, 0.300 mmol), *N*-methyl allylamine (0.0240 g, 0.330 mmol), and $PhCF_3$ (1.0 mL). The crude mixture was purified by silica gel column chromatography using 6% EtOAc/CH_2Cl_2 to afford the pure compound as a slightly yellow oil (0.0730 g, 100%). TLC Rf = 0.22 in 6% EtOAc/CH_2Cl_2. ^{1}H NMR (300 MHz; $CDCl_3$): δ 8.42-8.38 (m, 1H), 8.24-8.21 (m, 1H), 7.88-7.70 (m, 3H), 5.96-5.72 (m, 1H), 5.44-5.14 (m, 2H), 4.19-4.16 (m, 1.2H), 3.81-3.78 (m, 0.8H), 3.14 (s, 1.2H), 2.89 (s, 1.8H). ^{13}C NMR (75 MHz; $CDCl_3$): δ 160.0 (C), 157.7 (C), 153.8 (C), 153.7 (C), 139.3 (CH), 139.1 (CH), 134.0 (CH), 132.3 (CH), 132.2 (CH), 132.2 (CH), 131.1 (CH), 129.6 (C), 129.5 (C), 127.7 (C), 126.8 (CH), 126.7 (CH), 126.5 (CH), 126.5 (CH), 118.8 (CH_2), 118.1 (CH_2), 53.4 (CH_2), 51.7 (CH_2), 35.2 (CH_3), 34.3 (CH_3). IR (film): 1717, 1668, 1558, 1477, 1266, 1236, 1202 cm^{-1}. HRMS (EI): Exact mass calcd for $C_{13}H_{13}N_3O_2$ $[M]^+$: 243.1008. Found: 234.1029.

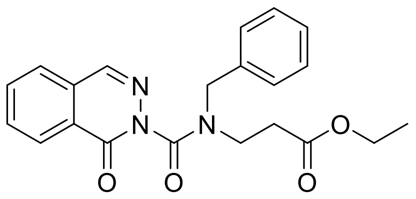

Table 6, entry 5: **Ethyl 3-(*N*-benzyl-1-oxo-1,2-dihydrophthalazine-2-carboxamido)propanoate (12e):** Synthesized according to general procedure **2** using carbazone ester **11** (0.0900 g, 0.300 mmol), *N*-benzyl-3-aminopropionic acid ethyl ester (0.0670 g, 0.330 mmol), and $PhCF_3$ (1.0 mL). The crude mixture was purified by silica gel column chromatography using 6% EtOAc/CH_2Cl_2 to afford the pure compound as a slightly yellow oil (0.103 g, 90%). TLC Rf = 0.28 in 6% EtOAc/CH_2Cl_2. ^{1}H NMR (300 MHz; $CDCl_3$): δ 8.47-8.41 (m, 1H), 8.28-8.21 (m, 1H), 7.91-7.69 (m, 3H), 7.49-7.21 (m, 5H), 4.86 (s, 0.9H), 4.48 (s, 1.1H), 4.19-3.96 (m, 2H), 3.76-3.71 (m, 1H), 3.54-3.49 (m, 1H), 2.81-2.76 (m, 1H) 2.59-2.55 (m, 1H), 1.30-1.20 (m, 3H). ^{13}C NMR (75 MHz; $CDCl_3$): δ 171.6 (C), 170.8 (C), 158.2 (C), 154.6 (C), 153.9 (CH), 139.4 (CH), 139.3 (CH), 135.6 (C), 135.4 (C), 134.1 (CH), 134.1 (CH), 132.4 (CH), 132.3 (CH), 129.6 (C), 129.6 (C), 128.8 (CH), 128.7 (CH), 128.0 (CH), 127.9 (CH), 127.7 (CH), 126.8 (CH), 126.8 (CH), 126.6 (CH), 126.5 (CH), 60.7 (CH_2), 60.7 (CH_2), 53.2 (CH_2), 50.7 (CH_2), 43.7 (CH_2), 43.1 (CH_2), 33.3 (CH_2), 31.8 (CH_2), 14.1 (CH_4), 13.9 (CH_3). IR (film): 3335, 1771, 1723, 1654, 1632, 1616, 1601, 1265, 1171 cm^{-1}. HRMS (EI): Exact mass calcd for $C_{21}H_{21}N_3O_4$ $[M]^+$: 379.1532. Found: 379.1556.

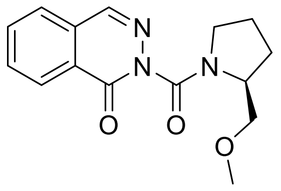

Table 6, entry 6: (*S*)-2-(2-(Methoxymethyl)pyrrolidine-1-carbonyl)phthalazin-1(2H)-one (12f):

Synthesized according to general procedure **2** using carbazone ester **11** (0.0900 g, 0.300 mmol), (*S*)-(+)-2-(methoxymethyl)pyrrolidine (0.0380 g, 0.330 mmol), and $PhCF_3$ (1.0 mL). The crude mixture was purified by silica gel column chromatography using 30% EtOAc/CH_2Cl_2 to afford the pure compound as a colorless oil (0.0560 g, 65%). TLC Rf = 0.28 in 30% EtOAc/CH_2Cl_2. ^{1}H NMR (300 MHz; $CDCl_3$): δ 8.40 (d, *J* = 7.6 Hz, 1H), 8.22 (s, 1H), 7.88-7.71 (m, 3H), 4.39-4.32 (m, 0.75H), 4.07-3.99 (m, 0.125H), 3.83-3.58 (m, 2H), 3.46-3.09 (m, 5H), 2.11-1.78 (m, 4H). ^{13}C NMR (75 MHz; $CDCl_3$): δ 157.5 (C), 151.8 (C), 139.0 (CH), 138.8 (CH), 133.9 (CH), 132.2 (CH), 129.6 (C), 127.9 (C), 126.7 (CH), 126.4 (CH), 73.7 (CH_2), 71.4 (CH_2), 59.2 (CH), 58.9 (C), 58.0 (CH_3), 57.8 (CH_3), 47.7 (CH_2), 28.7 (CH_2), 27.8 (CH_2), 23.6 (CH_2), 22.5 (CH_3). IR (film): 1730, 1650, 1445, 1432, 1408, 1265, 1067 cm^{-1}. HRMS (EI): Exact mass calcd for $C_{15}H_{17}N_3O_3$ $[M]^+$: 287.1270. Found: 287.1274.

Pyrazoles (Tables 7)

General procedure 3 (substrate preparation): An oven-dried round bottomed flask was charged with a stir bar, phenylcarbazate (1.00 equiv.), a ketone (1.10 equiv.), acetic acid (0.150 equiv.), and MeOH (0.3 M). The contents were refluxed for 16 hours. The crude mixture was concentrated under reduced pressure and dissolved in a 99:1 THF:MeOH solution (0.1 M). TBAF (1.10 equiv.) was added dropwise at –78 °C, and the resulting solution was stirred for 15 minutes. The reaction was quenched with a saturated aqueous solution of NH_4Cl and the organic phase was extracted with CH_2Cl_2. The reaction was concentrated under reduced pressure and purification by silica gel column chromatography or recrystallization gave the corresponding hydrazones.

General procedure 4: An oven-dried round bottom flask was charged with a stir bar, hydrazone (1.00 equiv.), an amine (1.10 equiv.), $PhCF_3$ or THF (0.3 M), and DBU (0.200 equiv.). The reaction was conducted at room temperature or 50 °C. The reaction was concentrated under reduced pressure and purified by silica gel column chromatography to give the corresponding products.

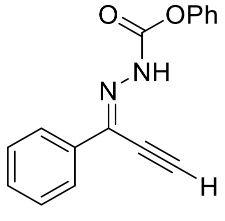

(*E*)-Phenyl-2-(1-phenylprop-2-yn-1-ylidene)hydrazinecarboxylate (13a): Synthesized according to general procedure **3** using phenylcarbazate (1.90 g, 12.5 mmol), 1-phenyl-3-(trimethylsilyl)prop-2-yn-1-one[1] (2.78 g, 13.8 mmol), acetic acid (0.113 g, 0.150 mmol), and CH_3OH (42 mL, 0.3 M). TBAF (13.8 mL of a 1 M solution in THF, 13.8 mmol) was added dropwise at -78 °C. The reaction was quenched after 15 minutes and the organic phase was extracted with CH_2Cl_2. The crude mixture was purified by silica gel column chromatography using 40% hexanes/CH_2Cl_2 to afford the pure compound as an amorphous pale yellow solid

(2.80 g, 85% over 2 steps). TLC Rf = 0.35 in 40% hexanes/CH_2Cl_2. ^{1}H NMR (300 MHz; $CDCl_3$): δ 9.15 (br s, 1H), 8.00 (dt, *J* = 3.9, 2.8 Hz, 2H), 7.44-7.40 (m, 5H), 7.29-7.24 (m, 3H), 4.09 (s, 1H). ^{13}C NMR (75 MHz; $CDCl_3$): δ 133.7 (C), 130.4 (CH), 129.6 (CH), 128.6 (CH), 126.8 (CH), 126.1 (CH), 121.6 (CH), 93.1 (C), 72.5 (CH). IR (film): 1770, 1737, 1683, 1481, 1455, 1423, 1363, 1348, 1257, 1249, 1191, 1114 cm^{-1}. HRMS (EI): Exact mass calcd for $C_{16}H_{12}N_2O_2$ $[M]^+$: 264.0899. Found: 264.0895.

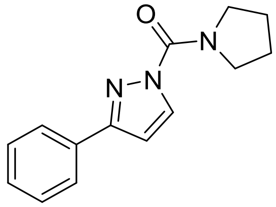

Table 7, entry 14a: (3-Phenyl-1H-pyrazol-1-yl)(pyrrolidin-1-yl)methanone: Synthesized according to general procedure **4** using hydrazone **13a** (0.159 g, 0.600 mmol), pyrrolidine (0.0470 g, 0.660 mmol), DBU (0.020 mL, 0.12 mmol), and THF (2.0 mL) at room temperature for 16 hours. The crude mixture was purified by silica gel column chromatography using 10% hexanes/CH_2Cl_2 to afford the pure compound as an amorphous white solid (0.129 g, 89%). TLC Rf = 0.15 in 10% hexanes/CH_2Cl_2. ^{1}H NMR (300 MHz; $CDCl_3$): δ 8.30 (d, *J* = 2.8 Hz, 1H), 7.88-7.84 (m, 2H), 7.47-7.34 (m, 4H), 6.69 (d, *J* = 2.8 Hz, 1H), 4.14 (br s, 2H), 3.72 (br s, 2H), 1.99 (br s, 4H). ^{13}C NMR (75 MHz; $CDCl_3$): δ 153.2 (C), 149.6 (C), 132.4 (C), 132.1 (CH), 128.5 (CH), 128.4 (CH) 125.8 (CH), 104.2 (CH), 50.2 (CH_2), 48.8 (CH_2), 26.7 (CH_2), 23.7 (CH_2). IR (film): 1717, 1670, 1558, 1423, 1265, 1202 cm^{-1}. HRMS (EI): Exact mass calcd for $C_{14}H_{15}N_3O$ $[M]^+$: 241.1215. Found: 241.1230.

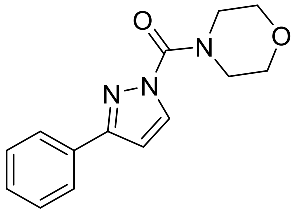

Table 7, entry 14b: Morpholino(3-phenyl-1H-pyrazol-1-yl)methanone: Synthesized according to general procedure **4** using carbazone **13a** (0.159 g, 0.600 mmol), morpholine (0.0871 g, 0.660 mmol), DBU (0.020 mL, 0.12 mmol), and THF (2.0 mL) at room temperature for 16 hours. The crude mixture was purified by silica gel column chromatography using a gradient of CH_2Cl_2 to 5% EtOAc/CH_2Cl_2 to afford the pure compound as a crystalline white solid (0.139 g, 90%). TLC Rf = 0.60 in 10% EtOAc/CH_2Cl_2. ^{1}H NMR (300 MHz; $CDCl_3$): δ 8.18 (d, *J* = 2.8 Hz, 1H), 7.84-7.81 (m, 2H), 7.46-7.37 (m, 3H), 6.69 (d, *J* = 2.8 Hz, 1H), 3.99 (br s, 4H), 3.83 (m, 4H). ^{13}C NMR (75 MHz; $CDCl_3$): δ 154.0 (C), 151.6 (C), 133.6 (CH), 132.4 (C), 129.1 (CH), 129.0 (CH), 126.3 (CH), 105.2 (CH), 67.1 (CH_2), 47.8 (CH_2). IR (film): 3032, 1683, 1533, 1452, 1426, 1363, 1348, 1301, 1255, 1247, 1188, 1116, 1074, 1045, 1031 cm^{-1}. HRMS (EI): Exact mass calcd for $C_{14}H_{15}N_3O_2$ $[M]^+$: 257.1164. Found: 257.1136.

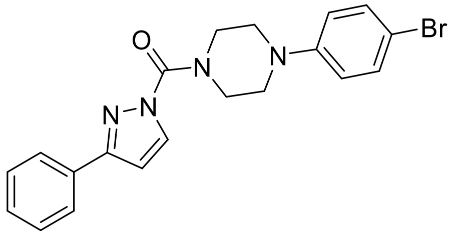

Table 7, entry 14c: (4-(4-Bromophenyl)piperazin-1-yl)(3-phenyl-1H-pyrazol-1-yl)methanone: Synthesized according to general procedure **4** using carbazone **13a** (0.0795 g, 0.300 mmol), 1-(4-bromophenyl)piperazine (0.0800 g, 0.330 mmol), DBU (0.0090 mL, 0.060 mmol), and THF (1.0 mL) at room temperature for 16 hours. The crude mixture was purified by Et_3N-treated silica gel column chromatography using 10% EtOAc/hexanes to afford the pure compound as an amorphous white solid (0.112 g, 91%). TLC Rf = 0.20 in 10% EtOAc/hexanes. ^{1}H NMR (300 MHz; $CDCl_3$): δ 8.20 (d, *J* = 2.8 Hz, 1H), 7.86-7.83 (m, 2H), 7.46-7.42 (m, 2H), 7.40-7.36 (m, 3H), 6.85-6.81 (m, 2H), 6.71 (d, *J* = 2.8 Hz, 1H), 4.12 (br s, 4H), 3.31 (t, *J* = 5.2 Hz, 4H). ^{13}C NMR (75 MHz; $CDCl_3$): δ 153.9 (C), 151.1 (C), 150.1 (C), 133.5 (CH), 132.3 (C), 132.2 (CH), 129.0 (C), 128.9 (CH), 126.2 (CH), 118.3 (CH), 112.8 (C), 105.1 (CH), 49.3 (CH_2), 46.7 (CH_2). IR (film): 3880, 3768, 1733, 1687, 1662, 1456, 1265 cm^{-1}. HRMS (EI): Exact mass calcd for $C_{20}H_{19}BrN_4O$ $[M]^+$: 410.0742. Found: 410.0753.

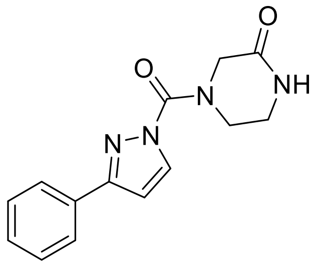

Table 7, entry 14d: 4-(3-Phenyl-1H-pyrazole-1-carbonyl)piperazin-2-one: Synthesized according to general procedure **4** using carbazone **13a** (0.159 g, 0.600 mmol), 2-oxopiperazine (0.0661 g, 0.660 mmol), DBU (0.020 mL, 0.12 mmol), and THF (2.0 mL) at room temperature for 16 hours. The crude mixture was purified by silica gel column chromatography using 5% CH_3OH/CH_2Cl_2 to afford the pure compound as a crystalline white solid (0.151 g, 93%). TLC Rf = 0.20 in 5% CH_3OH/CH_2Cl_2. ^{1}H NMR (300 MHz; $CDCl_3$): δ 8.20 (d, *J* = 2.8 Hz, 1H), 7.84-7.82 (m, 2H), 7.46-7.37 (m, 3H), 6.72 (d, *J* = 2.8 Hz, 1H), 6.69 (br s, 1H), 4.67 (br s, 2H), 4.15 (br s, 2H), 3.62 (br s, 2H). ^{13}C NMR (75 MHz; $CDCl_3$): δ 167.1 (C), 154.7 (C), 150.7 (C), 133.3 (CH), 131.8 (C), 129.1 (CH), 128.8 (CH), 126.2 (CH), 105.4 (CH). IR (film): 3267, 3060, 2900, 1710, 1654, 1537, 1452, 1427, 1352, 1332, 1315, 1249, 1105, 1061, 1034 cm^{-1}. HRMS (EI): Exact mass calcd for $C_{14}H_{14}N_4O_2$ $[M]^+$: 270.1117. Found: 270.1097.

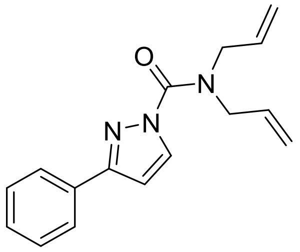

Table 7, entry 14e: *N*,*N*-Diallyl-3-phenyl-1H-pyrazole-1-carboxamide: Synthesized according to general procedure **4** using hydrazone **13a** (0.159 g, 0.600 mmol), diallylamine (0.0800 mL, 0.660 mmol), DBU (0.020 mL, 0.12 mmol), and THF (2.0 mL) at room temperature for 16 hours. The crude mixture was purified by silica gel column chromatography using 20% hexanes/CH_2Cl_2 to afford the pure compound as an amorphous white solid (0.141 g, 88%). TLC Rf = 0.42 in 20% hexanes/CH_2Cl_2. ^{1}H NMR (300 MHz; $CDCl_3$): δ 8.22 (d, J = 2.8 Hz, 1H), 7.87-7.83 (m, 2H), 7.47-7.34 (m, 4H), 6.70 (d, J = 2.8 Hz, 1H), 6.15-5.97 (m, 2H), 5.31-5.25 (m, 4H), 4.27 (s, 4H). ^{13}C NMR (75 MHz; $CDCl_3$): δ 153.4 (C), 151.6 (C), 133.0 (CH), 132.4 (C), 128.7 (CH), 128.6 (CH), 126.0 (CH), 118.2 (CH_2), 104.6 (CH), 116.9 (CH_2), 105.9 (CH), 51.0 (CH_2). IR (film): 1714, 1654, 1522, 1481, 1424, 1259, 1180 cm^{-1}. HRMS (EI): Exact mass calcd for $C_{16}H_{17}N_3O$ $[M]^+$: 267.1372. Found: 267.1355.

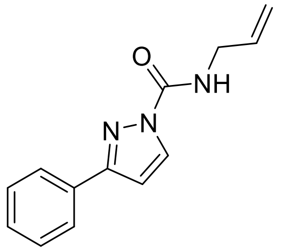

Table 7, entry 14f: *N*-Allyl-3-phenyl-1H-pyrazole-1-carboxamide: Synthesized according to general procedure **4** using hydrazone **13a** (0.159 g, 0.600 mmol), allylamine (0.050 mL, 0.66 mmol), DBU (0.020 mL, 0.12 mmol), and THF (2.0 mL) at 50 °C for 24 hours. The crude mixture was purified by Et_3N-treated silica gel column chromatography using 20% hexanes/CH_2Cl_2 to afford the pure compound as an amorphous white solid (0.100 g, 73%). TLC Rf = 0.31 in 20% hexanes/CH_2Cl_2. ^{1}H NMR (300 MHz; $CDCl_3$): δ 8.27 (d, J = 2.8 Hz, 1H), 7.88-7.84 (m, 2H), 7.48-7.36 (m, 4H), 6.73 (d, J = 2.8 Hz, 1H), 5.96 (ddt, J = 17.2, 10.2, 5.6 Hz, 1H), 5.37-5.21 (m, 2H), 4.12-4.07 (m, 2H). ^{13}C NMR (75 MHz; $CDCl_3$): δ 154.0 (C), 133.5 (C), 132.0 (C), 129.9 (CH), 128.9 (CH), 128.7 (CH), 126.1 (CH), 116.9 (CH_2), 105.9 (CH), 42.7 (CH_2). IR (film): 1718, 1680, 1651, 1558, 1539, 1423, 1354, 1265 cm^{-1}. HRMS (EI): Exact mass calcd for $C_{13}H_{13}N_3O$ $[M]^+$: 227.1059. Found: 227.1085.

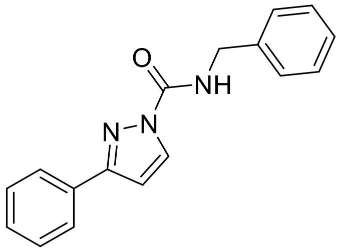

Table 7, entry 14g: *N*-Benzyl-3-phenyl-1H-pyrazole-1-carboxamide: Synthesized according to general procedure **4** using carbazone **13a** (0.159 g, 0.600 mmol), benzylamine (0.0701 g, 0.660 mmol), DBU (0.020 mL, 0.12 mmol), and THF (2.0 mL) at room temperature for 24 hours. The crude mixture was purified by Et_3N-treated silica gel column chromatography using 20% hexanes/CH_2Cl_2 to afford the pure compound as an amorphous white solid (0.130 g, 78%). TLC Rf = 0.60 in 20% hexanes/CH_2Cl_2. ^1H NMR (300 MHz; $CDCl_3$): δ 8.27 (d, *J* = 2.8 Hz, 1H), 7.82-7.80 (m, 2H), 7.58 (br s, 1H), 7.43-7.28 (m, 3H), 6.71 (d, *J* = 2.8 Hz, 1H), 4.63 (d, *J* = 6.13 Hz, 2H). ^{13}C NMR (75 MHz; $CDCl_3$): δ 154.1 (C), 149.9 (C), 137.6 (C), 131.9 (CH), 130.0 (CH), 128.9 (CH), 128.8 (CH), 128.7 (CH), 127.7 (CH), 126.2 (CH), 106.1 (CH), 44.4 (CH_2). IR (film): 3357, 1716, 1689, 1543, 1500, 1456, 1353, 1284, 1257, 1238, 1080, 1043 cm^{-1}. HRMS (EI): Exact mass calcd for $C_{17}H_{15}N_3O$ $[M]^+$: 277.1215. Found: 277.1217.

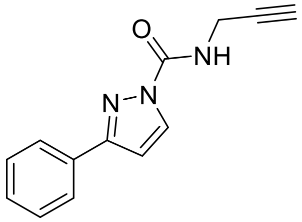

Table 7, entry 14h: 3-Phenyl-*N*-(prop-2-yn-1-yl)-1H-pyrazole-1-carboxamide: Synthesized according to general procedure **4** using carbazone **13a** (0.159 g, 0.600 mmol), propargylamine (0.0363 g, 0.660 mmol), DBU (0.020 mL, 0.12 mmol), and THF (2.0 mL) at 50 °C for 24 hours. The crude mixture was purified by Et_3N-treated silica gel column chromatography using 40% hexanes/CH_2Cl_2 to afford the pure compound as an amorphous white solid (0.100 g, 79%). TLC Rf = 0.35 in 40% hexanes/CH_2Cl_2. ^1H NMR (300 MHz; $CDCl_3$): δ 8.26 (d, *J* = 2.8 Hz, 1H), 7.86-7.84 (m, 2H), 7.47-7.37 (m, 4H), 6.73 (d, *J* = 2.8 Hz, 1H), 4.26 (dd, *J* = 5.7, 2.6 Hz, 2H), 2.32 (t, *J* = 2.55, 1H). ^{13}C NMR (75 MHz; $CDCl_3$): δ 154.5 (C), 149.6 (C), 132.0 (C), 130.1 (CH), 129.1 (CH), 128.9 (CH), 126.3 (CH), 106.4 (CH), 78.9 (C), 72.4 (CH), 30.1 (CH_2). IR (film): 3105, 1739, 1683, 1652, 1554, 1515, 1455, 1339, 1266 cm^{-1}. HRMS (EI): Exact mass calcd for $C_{13}H_{11}N_3O$ $[M]^+$: 225.0902. Found: 225.0897.

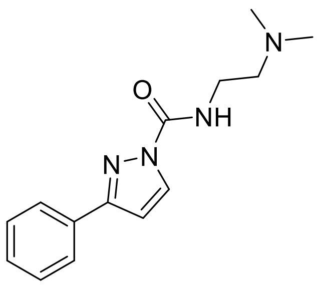

Table 7, entry 14i: *N*-(2-(Dimethylamino)ethyl)-3-phenyl-1H-pyrazole-1-carboxamide: Synthesized according to general procedure **4** using carbazone **13a** (0.159 g, 0.600 mmol), *N*,*N*-dimethylethylenediamine (0.0582 g, 0.660 mmol), DBU (0.020 mL, 0.12 mmol), and $PhCF_3$ (2.0 mL) at 50 °C for 16 hours. The crude mixture was purified by silica gel column chromatography using a gradient of 10% $EtOAc/CH_2Cl_2$ to 50% CH_3OH/CH_2Cl_2 to afford the pure compound as a colorless oil (0.138 g, 89%). TLC Rf = 0.10 in 10% $EtOAc/CH_2Cl_2$. ^1H NMR (300 MHz; $CDCl_3$): δ 8.25 (d, *J* = 2.7 Hz, 1H), 7.86-7.84 (m, 2H), 7.57 (br s, 1H), 7.45-7.35 (m, 3H), 6.70 (d, *J* = 2.7 Hz, 1H), 3.54 (q, *J* = 6.0 Hz, 2H), 2.56 (t, *J* = 6.2 Hz, 1H), 2.31 (s, 6H). ^{13}C NMR (75 MHz; $CDCl_3$): δ 154.0 (C), 150.1 (C), 132.2 (C), 129.9 (CH), 128.9 (CH), 128.8 (CH), 126.2 (CH), 105.8 (CH), 58.1 (CH_2), 45.4 (CH_3), 38.0 (CH_2). IR (film): 3023, 1733, 1716, 1683, 1662, 1526, 1455, 1353, 1262 cm^{-1}. HRMS (EI): Exact mass calcd for $C_{14}H_{18}N_4O$ $[M]^+$: 258.1481. Found: 258.1400.

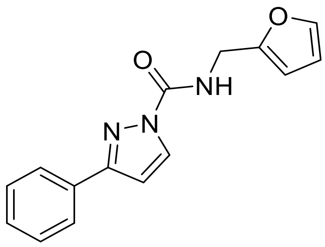

Table 7, entry 14j: *N*-(Furan-2-ylmethyl)-3-phenyl-1H-pyrazole-1-carboxamide: Synthesized according to general procedure **4** using carbazone **13a** (0.159 g, 0.600 mmol), furfurylamine (0.0641 g, 0.660 mmol), DBU (0.020 mL, 0.12 mmol), and THF (2.0 mL) at room temperature for 18 hours. The crude mixture was purified by Et_3N-treated silica gel column chromatography using CH_2Cl_2 to afford the pure compound as an amorphous white solid (0.137 g, 82%). TLC Rf = 0.60 in CH_2Cl_2. ^1H NMR (300 MHz; $CDCl_3$): δ 8.25 (d, *J* = 2.8 Hz, 1H), 7.83-7.80 (m, 2H), 7.54 (br s, 1H), 7.44-7.36 (m, 4H), 6.70 (d, *J* = 2.8 Hz, 1H), 6.33 (m, 2H) 4.62 (d, *J* = 5.9 Hz, 2H). ^{13}C NMR (75 MHz; $CDCl_3$): δ 154.1 (C), 149.9 (C), 137.6 (C), 131.9 (CH), 130.0 (CH), 128.9 (CH), 128.8 (CH), 128.7 (CH), 127.7 (CH), 126.2 (CH), 106.1 (CH), 44.4 (CH_2). IR (film): 3111, 2989, 1716, 1683, 1647, 1444, 1353, 1262, 1238, 1161 cm^{-1}. HRMS (EI): Exact mass calcd for $C_{15}H_{13}N_3O_2$ $[M]^+$: 267.1008. Found: 267.1023.

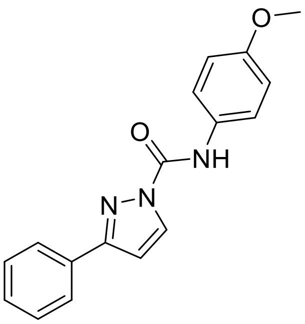

Table 7, entry 14k: *N*-(4-Methoxyphenyl)-3-phenyl-1H-pyrazole-1-carboxamide: Synthesized according to general procedure **4** using carbazone **13a** (0.159 g, 0.600 mmol), *p*-methoxyaniline (0.0813 g, 0.660 mmol), DBU (0.020 mL, 0.12 mmol), and THF (2.0 mL) at room temperature for 16 hours. The crude mixture was purified by Et_3N-treated silica gel column chromatography using 40% hexanes/CH_2Cl_2 to afford the pure compound as an amorphous white solid (0.133 g, 76%). TLC Rf = 0.40 in 40% hexanes/CH_2Cl_2. ^{1}H NMR (300 MHz; $CDCl_3$): δ 9.05 (br s, 1H) 8.34 (d, J = 2.8 Hz, 1H), 7.90-7.88 (m, 2H), 7.57-7.53 (m, 2H), 7.49-7.39 (m, 3H), 6.95-6.93 (m, 2H), 6.76 (d, J = 2.8 Hz, 1H), 3.82 (s, 3H). ^{13}C NMR (75 MHz; $CDCl_3$): δ 156.9 (C), 154.3 (C), 147.3 (C), 131.9 (C), 130.0 (CH), 129.7 (C), 129.2 (CH), 128.9 (CH), 126.4 (CH), 121.8 (CH), 114.5 (CH), 106.6 (CH), 55.6 (CH_3). IR (film): 3062, 1733, 1716, 1652, 1596, 1502, 1456, 1419, 1355, 1299, 1247, 1226, 1039 cm^{-1}. HRMS (EI): Exact mass calcd for $C_{17}H_{15}N_3O_2$ $[M]^+$: 293.1164. Found: 293.1213.

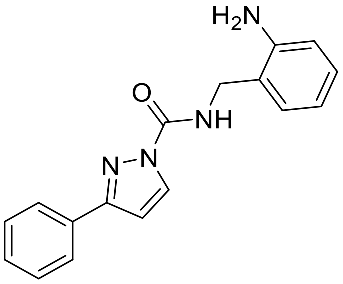

Table 7, entry 14l: *N*-(2-Aminobenzyl)-3-phenyl-1H-pyrazole-1-carboxamide: Synthesized according to general procedure **4** using carbazone **13a** (0.159 g, 0.600 mmol), 2-aminobenzylamine (0.0701 g, 0.660 mmol), DBU (0.020 mL, 0.12 mmol), and THF (2.0 mL) at room temperature for 24 hours. The product precipitated out of solution as an amorphous white solid and was filtrated with Et_2O to give the desired pure product (0.157 g, 90%). TLC Rf = 0.10 in 10% EtOAc/CH_2Cl_2. ^{1}H NMR (300 MHz; DMSO-d_6): δ 8.67 (br s, 1H), 8.29 (m, 1H), 7.90 (m, 2H), 7.39 (m, 3H), 7.10 (m, 1H), 6.95 (m, 2H), 6.62 (m, 1H), 6.52 (br s, 1H), 4.99 (m, 2H), 4.34 (m, 3H). ^{13}C NMR (75 MHz; DMSO-d_6): δ 158.3 (C), 155.0 (C), 151.4 (C), 137.0 (C), 135.7 (C), 134.6 (CH), 134.1 (CH), 134.0 (CH), 133.3 (CH), 131.2 (CH), 126.6 (C), 121.0 (CH), 120.0 (CH), 111.3 (CH), 45.7 (CH_2). IR (film): 3002, 1670, 1515, 1455, 1348, 1249, 1184, 1114, 1072, 1031 cm^{-1}. HRMS (EI): Exact mass calcd for $C_{17}H_{16}N_4O$ $[M]^+$: 292.1324. Found: 292.1324.

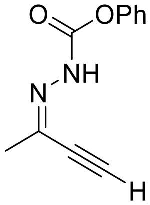

Carbazone 13b: (*Z*)-Phenyl-2-(but-3-yn-2-ylidene)hydrazinecarboxylate: Synthesized according to general procedure **3** using phenylcarbazate (1.52 g, 10.0 mmol), the corresponding ketone (1.40 g, 10.0 mmol), and CH_3OH (50 mL, 0.2 M). TBAF (10.0 mL of a 1M solution in THF, 10.0 mmol) was added dropwise at -78 °C. The reaction was quenched after 15 minutes and the organic phase was extracted with CH_2Cl_2. The crude mixture was purified by silica gel column chromatography using CH_2Cl_2 to afford the pure compound as an amorphous pale yellow solid (1.70 g, 84%). TLC Rf = 0.31 in CH_2Cl_2. ^{1}H NMR (300 MHz; DMSO-d_6): δ 10.40 (br s, 1H), 7.46-7.39 (m, 2H), 7.29-7.23 (m, 1H), 7.21-7.17 (m, 2H), 5.13 (s, 1H), 2.10 (s, 3H). ^{13}C NMR (75 MHz; DMSO-d_6): δ 151.7 (C), 150.4 (C), 129.5 (CH), 125.6 (CH), 121.8 (CH), 93.9 (C), 74.9 (CH), 22.6 (CH_3). IR (film): 2966, 1755, 1647, 1627, 1593, 1467, 1387, 1265, 1200, 1132, 1109 cm^{-1}. HRMS (EI): Exact mass calcd for $C_{11}H_{10}N_2O_2$ $[M]^+$: 202.0747. Found: 202.0754.

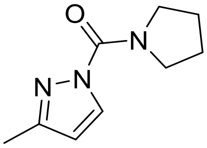

Table 7, entry 14m: (3-Methyl-1H-pyrazol-1-yl)(pyrrolidin-1-yl)methanone: Synthesized according to general procedure **4** using hydrazone **13b** (0.122 g, 0.600 mmol), pyrrolidine (0.0470 g, 0.660 mmol), DBU (0.020 mL, 0.12 mmol), and THF (2.0 mL) at 50 °C for 24 hours. The crude mixture was purified by silica gel column chromatography using 3% EtOAc/CH_2Cl_2 to afford the pure compound as an amorphous white solid (0.0900 g, 85%). TLC Rf = 0.29 in 3% EtOAc/CH_2Cl_2. ^{1}H NMR (300 MHz; $CDCl_3$): δ 8.12 (d, *J* = 2.7 Hz, 1H), 6.13 (d, *J* = 2.7 Hz, 1H), 4.06-3.54 (m, 4H), 2.29 (s, 3H), 1.95-1.90 (m, 4H). ^{13}C NMR (75 MHz; $CDCl_3$): δ 151.3 (C), 131.7 (CH), 107.2 (CH), 13.8 (CH_3). IR (film): 1705, 1663, 1558, 1458, 1437, 1420, 1310, 1265, 1157 cm^{-1}. HRMS (EI): Exact mass calcd for $C_9H_{13}N_3O$ $[M]^+$: 179.1059. Found: 179.1053.

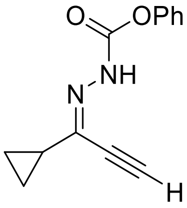

Carbazone 13c: (*Z*)-Phenyl-2-(1-cyclopropylprop-2-yn-1-ylidene)hydrazinecarboxylate: Synthesized according to general procedure **3** using phenyl carbazate (1.32 g, 8.70 mmol), 1-

cyclopropyl-3-(trimethylsilyl)prop-2-yn-1-one[2] (1.59 g, 9.57 mmol), acetic acid (0.113 g, 0.131 mmol), and CH_3OH (30 mL, 0.3 M). TBAF (9.6 mL of a 1 M solution in THF, 9.6 mmol) was added dropwise at -78 °C. The reaction was quenched after 15 minutes and the organic phase was extracted with CH_2Cl_2. The crude mixture was purified by silica gel column chromatography using a gradient of 100% CH_2Cl_2 to 2.5% CH_3OH/CH_2Cl_2 to afford the pure compound as an amorphous white solid (1.32 g, 66% over 3 steps). TLC Rf = 0.40 in 2.5% CH_3OH/CH_2Cl_2. ^{1}H NMR (300 MHz; $CDCl_3$): δ 8.77 (br s, 1H), 7.39 (t, *J* = 7.9 Hz, 2H), 7.24 (d, *J* = 7.5 Hz, 1H), 7.20 (d, 2H), 3.75 (s, 1H) 2.02-1.99 (m, 1H), 0.90 (ddd, *J* = 9.9, 3.3, 1.2 Hz, 4H). ^{13}C NMR (75 MHz; $CDCl_3$): δ 150.8 (C), 129.6 (CH), 126.0 (CH), 121.5 (CH), 91.3 (C), 71.0 (CH), 16.1 (CH), 6.2 (CH_2). IR (film): 3244, 2090, 1753, 1733, 1674, 1662, 1505, 1481, 1340, 1199, 1025, 1004 cm^{-1}. HRMS (EI): Exact mass calcd for $C_{13}H_{12}N_2O_2$ $[M]^+$: 228.0899. Found: 228.0887.

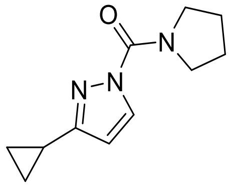

Table 7, entry 14n: (3-Cyclopropyl-1H-pyrazol-1-yl)(pyrrolidin-1-yl)methanone: Synthesized according to general procedure **4** using carbazone **13c** (0.137 g, 0.600 mmol), pyrrolidine (0.0470 g, 0.660 mmol), DBU (0.020 mL, 0.12 mmol), and THF (2.0 mL) at room temperature for 16 hours. The crude mixture was purified by Et_3N-treated silica gel column chromatography using 2.5% $EtOAc/CH_2Cl_2$ to afford the pure compound as an amorphous white solid (0.111 g, 90%). TLC Rf = 0.20 in 2.5% $EtOAc/CH_2Cl_2$. ^{1}H NMR (300 MHz; $CDCl_3$): δ 8.10 (d, *J* = 2.7 Hz, 1H), 6.02 (d, *J* = 2.7 Hz, 1H), 3.95 (br s, 2H), 3.64 (br s, 2H), 1.96-1.90 (m, 5H), 0.96-0.91 (m, 2H), 0.79-0.75 (m, 2H). ^{13}C NMR (75 MHz; $CDCl_3$): δ 157.8 (C), 150.8 (C), 131.7 (CH), 104.3 (CH), 50.1 (CH_2), 48.8 (CH_2), 27.0 (CH_2), 24.2 (CH_2), 9.50 (CH_3), 8.3 (CH_2). IR (film): 3142, 1786, 1699, 1675, 1505,1461, 1331, 1299, 1111, 1009 cm^{-1}. HRMS (EI): Exact mass calcd for $C_{11}H_{15}N_3O$ $[M]^+$: 205.1215. Found: 205.1248.

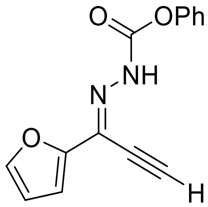

Carbazone 13d: (*E*)-Phenyl-2-(1-(furan-2-yl)prop-2-yn-1-ylidene)hydrazinecarboxylate: Synthesized according to general procedure **3** using phenyl carbazate (0.959 g, 6.31 mmol), 1-furan-3-(trimethylsilyl)prop-2-yn-1-one (1.33 g, 6.94 mmol), acetic acid (0.0570 g, 0.150 mmol), and CH_3OH (21 mL, 0.3 M). TBAF (6.9 mL of a 1 M solution in THF, 6.9 mmol) was added dropwise at -78 °C. The reaction was quenched after 15 minutes and the organic phase was extracted with CH_2Cl_2. The crude mixture was purified by silica gel column chromatography using a gradient of 60% hexanes/CH_2Cl_2 to 40% hexanes/CH_2Cl_2, followed by 100% CH_2Cl_2 to afford the pure compound as an amorphous pale yellow solid (1.39 g, 87% over 2 steps). TLC Rf = 0.30 in 40% hexanes/CH_2Cl_2. ^{1}H NMR (300 MHz; $CDCl_3$): δ 9.15 (br s, 1H), 8.00 (dt, *J* = 3.9, 2.8 Hz, 2H), 7.44-7.40 (m, 5H), 7.29-7.24 (m, 3H), 4.09 (s, 1H). ^{13}C NMR (75 MHz; $CDCl_3$): δ

133.7 (C), 130.4 (CH), 129.6 (CH), 128.6 (CH), 126.8 (CH), 126.1 (CH), 121.6 (CH), 93.1 (C), 72.5 (CH). IR (film): 3004, 2889, 1570, 1541, 1429, 1382, 1371, 1359, 1265, 1253, 1242, 1225, 1199, 1183, 1060, 1037, 1026 cm^{-1}. HRMS (EI): Exact mass calcd for $C_{14}H_{10}N_2O_3$ $[M]^+$: 254.0691. Found: 254.0723.

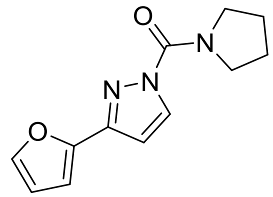

Table 7, entry 14o: (3-(Furan-2-yl)-1H-pyrazol-1-yl)(pyrrolidin-1-yl)methanone: Synthesized according to general procedure **4** using carbazone **14d** (0.153 g, 0.600 mmol), pyrrolidine (0.0470 g, 0.660 mmol), DBU (0.020 mL, 0.12 mmol), and THF (2.0 mL) at room temperature for 16 hours. The crude mixture was purified by Et_3N-treated silica gel column chromatography using 2.5% EtOAc/CH_2Cl_2 to afford the pure compound as an amorphous pale yellow solid (0.131 g, 94%). TLC Rf = 0.20 in 2.5% EtOAc/CH_2Cl_2. ^1H NMR (300 MHz; $CDCl_3$): δ 8.23 (d, *J* = 2.8 Hz, 1H), 7.48 (dd, *J* = 1.8, 0.8 Hz, 1H), 6.74 (dd, *J* = 3.4, 0.8 Hz, 1H), 6.58 (d, *J* = 2.8 Hz, 1H), 6.48 (dd, *J* = 3.4, 1.8 Hz, 1H), 4.05 (br s, 2H), 3.68 (br s, 2H), 1.95 (br s,4H). ^{13}C NMR (75 MHz; $CDCl_3$): δ 149.9 (C), 148.2 (C), 146.0 (C), 142.7 (CH), 132.2 (CH), 111.6 (CH), 107.4 (CH), 104.5 (CH), 50.3 (CH_2), 48.8 (CH_2), 26.9 (CH_2), 24.1 (CH_2). IR (film): 2989, 2883, 1674, 1544, 1430, 1386, 1359, 1348, 1267, 1211, 1039 cm^{-1}. HRMS (EI): Exact mass calcd for $C_{12}H_{13}N_3O_2$ $[M]^+$: 231.1008. Found: 231.0999.

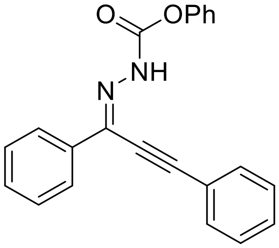

Carbazone 13e: (*Z*)-Phenyl 2-(1,3-diphenylprop-2-yn-1-ylidene)hydrazinecarboxylate: Synthesized according to general procedure **3** using phenyl carbazate (1.23 g, 8.12 mmol), 1,3-diphenylprop-2-yn-1-one[3] (1.84 g, 8.93 mmol), acetic acid (0.0730 g, 1.22 mmol), and CH_3OH (30 mL, 0.3 M). The crude mixture was purified by silica gel column chromatography using 5% EtOAc/hexanes to afford the pure compound as a crystalline pale yellow solid (0.911 g, 33%). TLC Rf = 0.20 in 10% EtOAc/hexanes. ^1H NMR (300 MHz; $CDCl_3$): δ 9.15 (br s, 1H), 8.07-8.05 (m, 2H), 7.66 (m, 2H), 7.48-7.39 (m, 8H), 7.26 (m, 3H). ^{13}C NMR (75 MHz; $CDCl_3$): δ 133.7 (C), 130.4 (CH), 129.6 (CH), 128.6 (CH), 126.8 (CH), 126.1 (CH), 121.6 (CH), 93.1 (C), 72.5 (CH). IR (film): 3336, 3085, 2198, 1762, 1733, 1716, 1704, 1593, 1506, 1473, 1434, 1357, 1334, 1315, 1307, 1266, 1161 1136, 1070, 1051, 1026, 1002 cm^{-1}. HRMS (EI): Exact mass calcd for $C_{22}H_{16}N_2O_2$ $[M]^+$: 340.1212. Found: 340.1219.

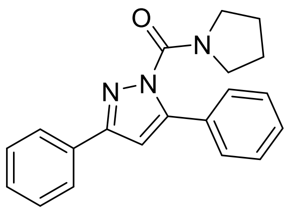

Table 7, entry 14p: (3,5-Diphenyl-1H-pyrazol-1-yl)(pyrrolidin-1-yl)methanone: Synthesized according to general procedure **4** using carbazone **13e** (0.102 g, 0.300 mmol), pyrrolidine (0.0237 g, 0.330 mmol), DBU (0.0090 mL, 0.060 mmol), and THF (1.0 mL) at 50 °C for 16 hours. The crude mixture was purified by Et_3N-treated silica gel column chromatography using 2.5% EtOAc/CH_2Cl_2 to afford the pure compound as a colorless oil (0.100 g, 98%). TLC Rf = 0.30 in 2.5% EtOAc/CH_2Cl_2. ^{1}H NMR (300 MHz; $CDCl_3$): δ 7.90-7.87 (m, 2H), 7.53 (dq, *J* = 6.2, 2.0 Hz, 2H), 7.46-7.34 (m, 6H), 6.76 (s, 1H), 3.63 (t, *J* = 6.7, 2H), 3.56 (t, *J* = 6.5, 2H), 1.91 (m, 4H). ^{13}C NMR (75 MHz; $CDCl_3$): δ 152.3 (C), 150.9 (C), 146.0 (C), 132.6 (C), 130.1 (C), 128.8 (CH) 128.7 (CH) 128.6 (CH) 128.5 (CH) 127.9 (CH), 126.1 (CH), 104.8 (CH), 48.9 (CH_2), 47.5 (CH_2), 26.1 (CH_2), 24.5 (CH_2). IR (film): 2989, 1670, 1544, 1429, 1419, 1386, 1363, 1348, 1255, 1043, 987 cm^{-1}. HRMS (EI): Exact mass calcd for $C_{20}H_{19}N_3O$ $[M]^+$: 317.1528. Found: 317.1541.

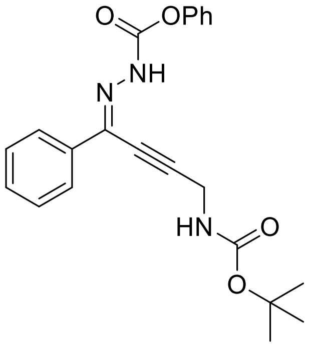

Carbazone 13f: (*Z*)-Phenyl-2-(4-((tert-butoxycarbonyl)amino)-1-phenylbut-2-yn-1-ylidene) hydrazinecarboxylate: Synthesized according to general procedure **3** using phenyl carbazate (0.305 g, 2.00 mmol), *tert*-butyl-(4-oxo-4-phenylbut-2-yn-1-yl)carbamate[3] (0.519 g, 2.00 mmol), and CH_3OH (10 mL, 0.3 M). The crude mixture was purified by silica gel column chromatography using CH_2Cl_2 to afford the pure compound as an amorphous red solid (0.386 g, 49%). TLC Rf = 0.35 in 40% hexanes/CH_2Cl_2. ^{1}H NMR (300 MHz; $CDCl_3$): δ 9.51 (br s, 1H), 7.96 (ddd, *J* = 5.43, 2.85, 1.29 Hz, 2H), 7.42-7.37 (m, 6H), 7.24 (t, *J* = 7.4 Hz, 3H), 5.17 (s, 1H), 4.23 (d, *J* = 5.71 Hz, 2H), 1.47 (s, 9H). ^{13}C NMR (75 MHz; $CDCl_3$): δ 155.5 (C), 134.0 (C) 130.0 (CH), 129.4 (CH), 128.4 (CH), 126.7 (CH), 125.8 (CH), 121.5 (CH), 102.0 (C), 80.7 (C), 72.2 (C), 31.4 (CH_2), 28.3 (CH_3). IR (film): 2332, 1689, 1652, 1558, 1508, 1436, 1265, 1195 cm^{-1}. HRMS (EI): Exact mass calcd for $C_{22}H_{23}N_3O_4$ $[M]^+$: 393.1689. Found: 393.1680.

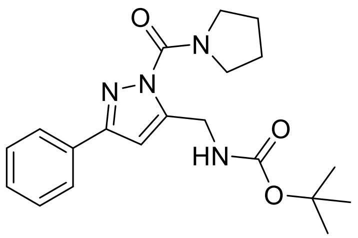

Table 7, entry 14q: *tert*-Butyl((3-phenyl-1-(pyrrolidine-1-carbonyl)-1H-pyrazol-5-yl)methyl) carbamate: Synthesized according to general procedure **4** using carbazone **13f** (0.118 g, 0.300 mmol), pyrrolidine (0.0237 g, 0.330 mmol), DBU (0.0090 mL, 0.060 mmol), and THF (1.0 mL) at 50 °C for 16 hours. The crude mixture was purified by Et_3N-treated silica gel column chromatography using 2.5% EtOAc/CH_2Cl_2 to afford the pure compound as a colorless oil (0.093 g, 83%). TLC Rf = 0.15 in 2.5% EtOAc/CH_2Cl_2. ^{1}H NMR (300 MHz; $CDCl_3$): δ 7.82-7.79 (m, 2H), 7.41-7.34 (m, 3H), 6.66 (s, 1H), 5.81 (s, 1H), 4.51 (d, *J* = 6.5 Hz, 2H), 4.00-3.97 (m, 2H), 3.68-3.65 (m, 2H), 1.99-1.95 (m, 4H), 1.43 (s, 9H). ^{13}C NMR (75 MHz; $CDCl_3$): δ 152.3 (C), 150.9 (C), 146.0 (C), 132.6 (C), 130.1 (C), 128.8 (CH), 128.7 (CH), 128.6 (CH), 128.5 (CH), 127.9 (CH), 126.1 (CH), 104.8 (CH), 48.9 (CH_2), 47.5 (CH_2), 26.1 (CH_2), 24.5 (CH_2). IR (film): 2989, 1683, 1558, 1444, 1393, 1367, 1353, 1255, 1238, 1195, 1154 cm^{-1}. HRMS (EI): Exact mass calcd for $C_{20}H_{26}N_4O_3$ $[M]^+$: 370.2005. Found: 370.2253.

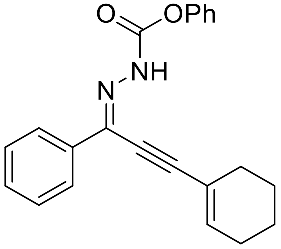

Carbazone 13g: (*Z*)-Phenyl-2-(3-(cyclohex-1-en-1-yl)-1-phenylprop-2-yn-1-ylidene) hydrazinecarboxylate: Synthesized according to general procedure **3** using phenyl carbazate (0.329 g, 2.16 mmol), 3-(cyclohex-1-en-1-yl)-1-phenylprop-2-yn-1-one[4] (0.500 g, 2.38 mmol), acetic acid (0.0200 g, 0.150 mmol), and CH_3OH (7.2 mL, 0.3 M) refluxed at 65 ^{o}C. The crude mixture was purified by silica gel column chromatography using 50% hexanes/CH_2Cl_2 to afford the pure compound as an amorphous white solid (0.588 g, 79%). TLC Rf = 0.20 in 50% hexanes/CH_2Cl_2. ^{1}H NMR (300 MHz; $CDCl_3$): δ 9.05 (br s, 1H), 7.98 (m, 2H), 7.43-7.38 (m, 5H), 7.28-7.24 (m, 3H), 6.50 (dt, *J* = 3.9, 2.0 Hz, 1H), 2.34-2.31 (m, 2H), 2.25-2.21 (m, 2H), 1.76-1.65 (m, 4H). ^{13}C NMR (75 MHz; $CDCl_3$): δ 140.4 (C), 130.1 (C), 129.6 (CH), 128.5 (CH), 126.9 (CH), 125.9 (C), 121.7 (C), 119.3 (C), 75.5 (C), 29.0 (CH_2), 26.1 (CH_2), 22.2 (CH_2), 21.3 (CH_2). IR (film): 2952, 2204, 1766, 1762, 1730, 1718, 1647, 1554, 1506, 1475, 1265, 1186, 1161, 1139 cm^{-1}. HRMS (EI): Exact mass calcd for $C_{22}H_{20}N_2O_2$ $[M]^+$: 344.1525. Found: 344.1512.

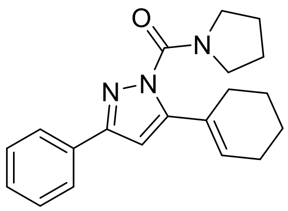

Table 7, entry 14r: (5-(Cyclohex-1-en-1-yl)-3-phenyl-1H-pyrazol-1-yl)(pyrrolidin-1-yl)methanone: Synthesized according to general procedure **4** using carbazone **13g** (0.103 g, 0.300 mmol), pyrrolidine (0.0237 g, 0.330 mmol), DBU (0.0090 mL, 0.060 mmol), and THF (1.0 mL) at 50 °C for 16 hours. The crude mixture was purified by Et_3N-treated silica gel column chromatography using 2.5% EtOAc/CH_2Cl_2 to afford the pure compound as a white solid (0.0783 g, 81%). TLC Rf = 0.15 in 100% CH_2Cl_2. ^{1}H NMR (300 MHz; $CDCl_3$): δ 7.82-7.79 (m, 2H), 7.41-7.30 (m, 3H), 6.48 (s, 1H), 6.02 (s, 1H), 3.66-3.59 (m, 4H), 2.35-2.31 (m, 2H), 2.21-2.17 (m, 2H), 1.99-1.91 (m, 4H), 1.75 (m, 2H) 1.67 (m, 2H). ^{13}C NMR (75 MHz; $CDCl_3$): δ 151.8 (C), 151.4 (C), 132.9 (C), 128.8 (C), 128.7 (C), 128.3 (CH), 127.9 (CH), 126.0 (CH), 103.2 (CH), 49.0 (CH_2), 47.5 (CH_2), 27.9 (CH_2), 26.25 (CH_2), 25.7 (CH_2), 24.6 (CH_2), 22.7 (CH_2), 21.9 (CH_2). IR (film): 3042, 2221, 1772, 1733, 1697, 1683, 1473, 1446, 1436, 1265, 1188 cm^{-1}. HRMS (EI): Exact mass calcd for $C_{20}H_{23}N_3O$ $[M]^+$: 321.1841. Found: 321.1920.

Other Attempts to form Pyrazoles (Not Shown in Table 7)

Table S1

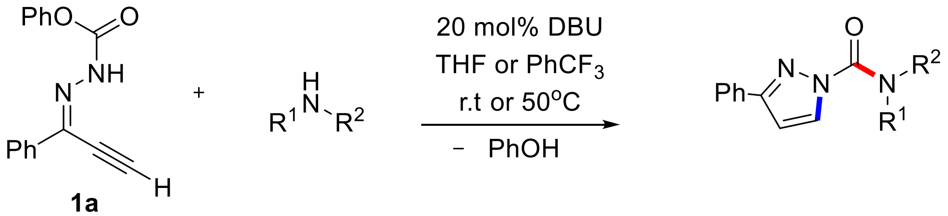

Solvent	Temp (°C)	Nucleophile	Product	Yield (%)[b]
THF	r.t.	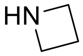	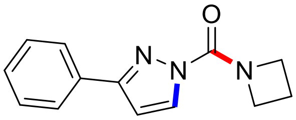	**35**
THF	50	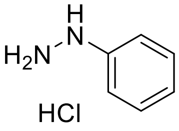	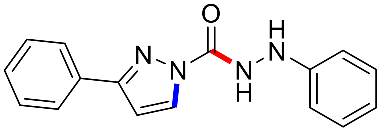	**0**
$PhCF_3$	50	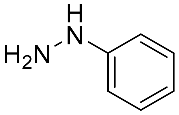	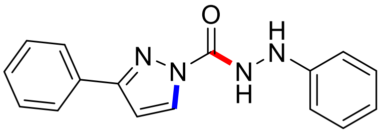	**0**
THF	50	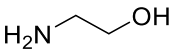	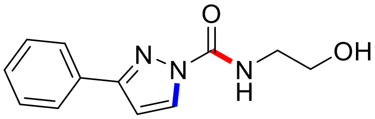	**67[b]**

THF	50	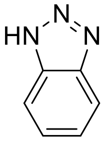	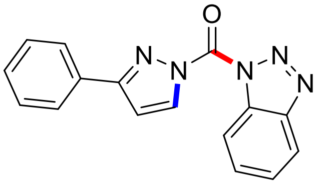	**0**
THF	r.t.	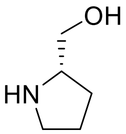	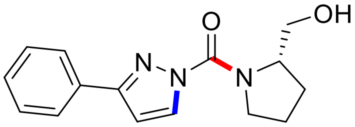	**0**
$PhCF_3$	50	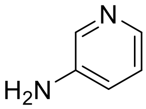	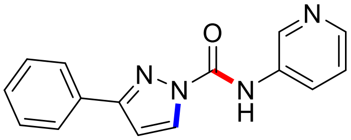	**65**[b]
THF	r.t.	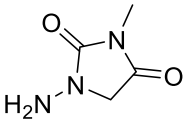	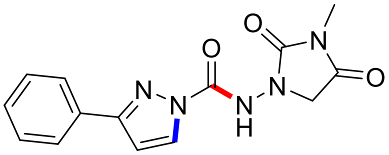	**55**

[a]Conditions: Alkynyl carbazone (1.00 equiv), amine (1.10 equiv) and DBU (0.20 equiv.) in $PhCF_3$ or THF (0.3 M) at room temperature or 50 °C for 16 to 24 hours in a closed vial. All reactions conducted at 0.1 mmol scale are NMR yields, taken using 1,3,5-trimethoxybenzene (TMB) as internal standard. [b]Isolated yield (0.6 mmol scale).

Table S2

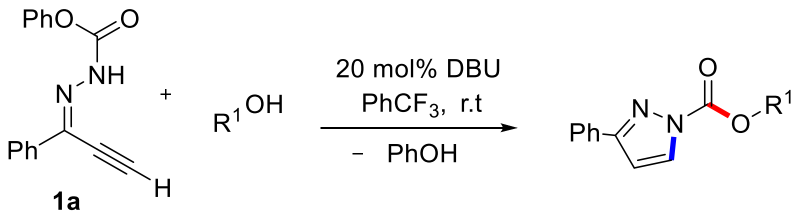

**Nucleophile**	**Product**	**Yield (%)**
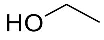	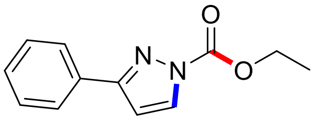	**20**
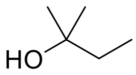	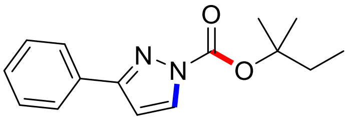	**0**
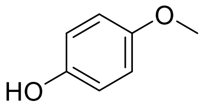	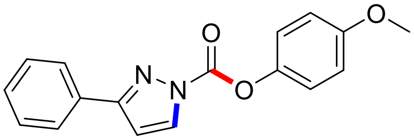	**40**

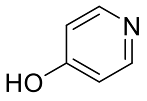	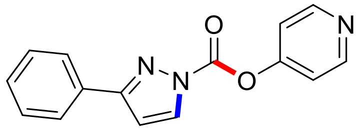	**0**
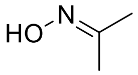	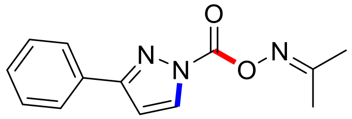	**21**
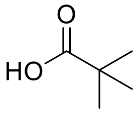	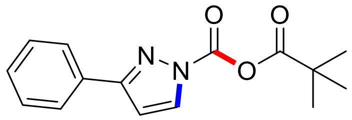	**0**

[a]Conditions: Alkynyl carbazone (2.00 equiv), alcohol (1.00 equiv) and DBU (0.20 equiv.) in $PhCF_3$ (0.3 M) at room temperature for 1 – 5 minutes in a closed vial. All reactions conducted at 0.1 mmol scale are NMR yields, taken using 1,3,5-trimethoxybenzene (TMB) as internal standard.

Azauracils (Tables 8-9)

General procedure 5: An oven-dried microwave tube was charged with a stir bar, a carbazone ester (1.0 equiv.), an amine (1.1 equiv.), and MeCN (0.3 M). The septum was removed and the tube was then quickly sealed with a microwave cap and heated for 6 hours at 175 °C. The tube was cooled to ambient temperature, concentrated under reduced pressure and purified by silica gel column chromatography to give the corresponding products.

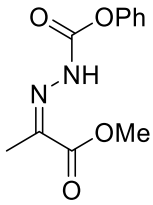

Carbazone 15a: (*Z*)-Phenyl 2-(1-methoxy-1-oxopropan-2-ylidene)hydrazinecarboxylate: To a solution of methyl pyruvate (1.53 g, 15.0 mmol) in MeOH (75 mL) was added phenyl carbazate (2.29 g, 15.0 mmol) and the solution was stirred overnight. The crude mixture was purified by filtration to afford the pure product as an amorphous white solid (2.72 g, 77%). TLC Rf = 0.75 in 30% EtOAc/CH_2Cl_2. ^{1}H NMR (300 MHz; DMSO-d_6): δ 11.07 (br s, 1H), 7.48-7.41 (m, 2H), 7.31-7.21 (m, 3H), 3.74 (s, 3H), 2.11 (s, 3H). ^{13}C NMR (75 MHz; DMSO-d_6): δ 164.8 (C), 150.3 (C), 129.6 (CH), 125.8 (CH), 121.8 (CH), 52.3 (CH_3), 13.1 (CH_3). IR (film): 3223, 1769, 1740, 1709, 1533, 1495, 1437, 1219, 1196, 1155, 1136 cm^{-1}. HRMS (EI): Exact mass calcd for $C_{11}H_{12}N_2O_4$ [M]$^+$: 236.0797. Found: 236.0797.

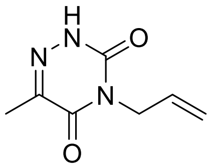

Table 8, entry 16a: 4-Allyl-6-methyl-1,2,4-triazine-3,5(2H,4H)-dione: Synthesized according to general procedure **5** using carbazone ester **15a** (0.355 g, 1.50 mmol), allylamine (0.12 mL, 1.6 mmol), and MeCN (5 mL). The crude mixture was purified by silica gel column chromatography using 30% EtOAc/hexanes to afford the pure compound as an amorphous white solid (0.185 g, 74%). TLC Rf = 0.21 in 30% EtOAc/hexanes. ^{1}H NMR (300 MHz; $CDCl_3$): δ 9.53 (br s, 1H), 5.88 (ddt, *J* = 17.1, 10.2, 6.0 Hz, 1H), 5.36-5.24 (m, 2H), 4.54 (dt, *J* = 6.0, 1.3 Hz, 2H), 2.26 (s, 3H). ^{13}C NMR (75 MHz; $CDCl_3$): δ 156.1 (C), 150.2 (C), 143.7 (C), 130.1 (CH), 119.3 (CH_2), 42.2 (CH_2), 16.7 (CH_3). IR (film): 3205, 3086,307, 2928, 1717, 1647, 1728, 1616, 1448, 1379, 1348, 1277, 1209, 1173 cm^{-1}. HRMS (EI): Exact mass calcd for $C_7H_9N_3O_2$ $[M]^+$: 167.0695. Found: 167.0674.

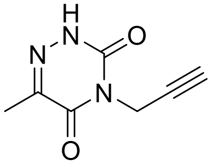

Table 8, entry 16b: 6-Methyl-4-(prop-2-yn-1-yl)-1,2,4-triazine-3,5(2H,4H)-dione: Synthesized according to general procedure **5** using carbazone ester **15a** (0.177 g, 0.750 mmol), propargylamine (0.0460 g, 0.830 mmol), and MeCN (2.5 mL). The crude mixture was purified by silica gel column chromatography using 30% EtOAc/hexanes to afford the pure compound as an amorphous beige solid (0.105 g, 85%). TLC Rf = 0.12 in 30% EtOAc/hexanes. ^{1}H NMR (300 MHz; DMSO-*d*$_6$): δ 12.38 (br s, 1H), 4.49 (d, *J* = 2.5 Hz, 2H). 3.17 (t, *J* = 2.4 Hz, 1H), 2.10 (s, 3H). ^{13}C NMR (75 MHz; DMSO-*d*$_6$): δ 155.6 (C), 148.7 (C), 78.0 (C), 74.6 (CH), 28.6 (CH_2), 16.5 (CH_3). IR (film): 3246, 2955, 2924, 2116, 1720, 1655, 1647, 1612, 1448, 1410, 1379, 1348, 1275, 1209 cm^{-1}. HRMS (EI): Exact mass calcd for $C_7H_7N_3O_2$ $[M]^+$: 165.0538. Found: 165.0546.

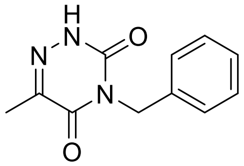

Table 8, entry 16c: 4-Benzyl-6-methyl-1,2,4-triazine-3,5(2H,4H)-dione: Synthesized according to general procedure **5** using carbazone ester **15a** (0.177 g, 0.750 mmol), benzylamine (0.0890 g, 0.830 mmol), and MeCN (2.5 mL). The crude mixture was purified by recrystallization using EtOAc/hexanes to afford the pure compound as an amorphous beige solid

(0.105 g, 85%). TLC Rf = 0.28 in 30% EtOAc/hexanes. ^{1}H NMR (300 MHz; DMSO-d_6): δ 12.34 (br s, 1H), 7.35-7.22 (m, 5H). 4.94 (s, 2H), 2.10 (s, 3H). ^{13}C NMR (75 MHz; DMSO-d_6): δ 156.4 (C), 149.5 (C), 142.1 (C), 136.2 (C), 128.4 (CH), 127.8 (CH), 127.4 (CH), 42.5 (CH_2), 16.6 (CH_3). IR (film): 3227, 3096, 2922, 1717, 1647, 1612, 1448, 1352, 1280. 1201. HRMS (EI): Exact mass calcd for $C_{11}H_{11}N_3O_2$ $[M]^+$: 217.0851. Found: 217.0860.

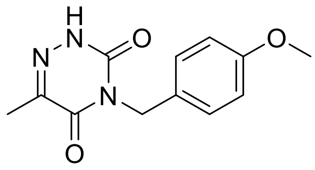

Table 8, entry 16d: 4-(4-Methoxybenzyl)-6-methyl-1,2,4-triazine-3,5(2H,4H)-dione: Synthesized according to general procedure **5** using carbazone ester **15a** (0.177 g, 0.750 mmol), 4-methoxybenzylamine (0.114 g, 0.830 mmol), and MeCN (2.5 mL). The crude mixture was purified by silica gel column chromatography using 30% EtOAc/hexanes to afford the pure compound as an amorphous white solid (0.131 g, 75%). TLC Rf = 0.24 in 30% EtOAc/hexanes. ^{1}H NMR (300 MHz; DMSO-d_6): δ 12.31 (br s, 1H), 7.29-7.24 (m, 2H), 6.88-6.83 (m, 2H) 4.86 (s, 2H), 3.71 (s, 3H), 2.08 (s, 3H). ^{13}C NMR (75 MHz; DMSO-d_6): δ 158.6 (C), 156.4 (C), 149.5 (C), 129.7 (CH), 128.2 (C), 113.7 (CH), 55.1 (CH_3), 42.0 (CH_2), 16.6 (CH_3). IR (film): 3292, 2958, 2927, 1717, 1655, 1647, 1610, 1512, 1445, 1352, 1302, 1246, 1202, 1175 cm^{-1}. HRMS (EI): Exact mass calcd for $C_{12}H_{13}N_3O_3$ $[M]^+$: 247.0957. Found: 247.0958.

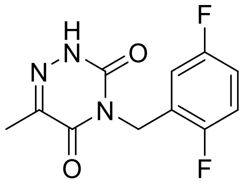

Table 8, entry 16e: 4-(2,5-Difluorobenzyl)-6-methyl-1,2,4-triazine-3,5(2H,4H)-dione: Synthesized according to general procedure **5** using carbazone ester **15a** (0.177 g, 0.750 mmol), 2,5-difluorobenzylamine (0.119 g, 0.830 mmol), and MeCN (2.5 mL). The crude mixture was purified by recrystallization using CH_2Cl_2 to afford the pure compound as an amorphous off-white solid (0.160 g, 84%). TLC Rf = 0.17 in 30% EtOAc/hexanes. ^{1}H NMR (300 MHz; DMSO-d_6): δ 12.34 (br s, 1H), 7.29-7.21 (m, 1H), 7.19-7.11 (m, 2H) 4.96 (s, 2H), 2.11 (s, 3H). ^{13}C NMR (75 MHz; DMSO-d_6): δ 156.9 (C), 149.9 (C), 142.6 (C), 125.6 (125.5, 125.4, 125.3, coupling with fluorine) (C), 117.3 (117.2, 117.0, 116.9, coupling with fluorine) (CH), 116.2 (116.0, 116.0, 115.8, 115.7, 115.7, 115.6, coupling with fluorine) (CH), 37.1 (37.0, coupling with fluorine) (CH_2), 17.0 (CH_3). IR (film): 3267, 2924, 2851, 2368, 1717, 1645, 1632, 1489, 1437, 1369, 1348, 1273, 1182 cm^{-1}. HRMS (EI): Exact mass calcd for $C_{11}H_9F_2N_3O_2$ $[M]^+$: 253.0663 Found: 253.0694.

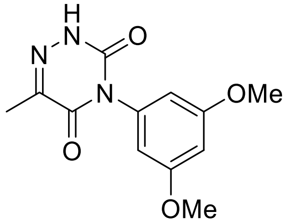

Table 8, entry 16f: 4-(3,5-dimethoxyphenyl)-6-methyl-1,2,4-triazine-3,5(2H,4H)-dione: Synthesized according to general procedure **5** using carbazone ester **15a** (0.177 g, 0.750 mmol), 3,5-dimethoxyaniline (0.127 g, 0.830 mmol), and MeCN (2.5 mL). The crude mixture was purified by filtration to afford the pure compound as an amorphous white solid (0.120 g, 61%). TLC Rf = 0.58 in 30% EtOAc/CH_2Cl_2. ^{1}H NMR (300 MHz; DMSO-*d*$_6$): δ 12.28 (br s, 1H), 6.58-6.56 (m, 1H), 6.51 (t, *J* = 2.2 Hz, 2H), 3.73 (s, 6H), 2.11 (s, 3H). ^{13}C NMR (75 MHz; DMSO-*d*$_6$): δ 160.6 (C), 156.4 (C), 149.2 (C), 142.5 (C), 135.4 (C), 106.8 (CH), 100.5 (CH), 55.4 (CH_3), 16.6 (CH_3). IR (film): 3205, 3117, 2916, 1718, 1653, 1610, 1597, 1558, 1477, 1429, 1213, 1161 cm^{-1}. HRMS (EI): Exact mass calcd for $C_{12}H_{13}N_3O_4$ [M]$^+$: 263.0906. Found: 263.0897.

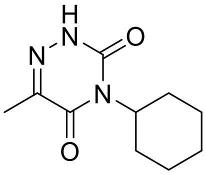

Table 8, entry 16g: 4-Cyclohexyl-6-methyl-1,2,4-triazine-3,5(2H,4H)-dione: Synthesized according to general procedure **5** using carbazone ester **15a** (0.177 g, 0.750 mmol), cyclohexylamine (0.0820g, 0.830 mmol), and MeCN (2.5 mL). The crude mixture was purified by filtration to afford the pure compound as an amorphous white solid (0.0700 g, 51%). TLC Rf = 0.48 in 30% EtOac/hexanes. ^{1}H NMR (300 MHz; DMSO-*d*$_6$): δ 12.04 (br s, 1H), 4.52 (t, *J* = 11.6 Hz, 1H), 2.31-2.17 (m, 2H), 2.05 (s, 3H), 1.84-1.69 (m, 2H), 1.66-1.45 (m, 4H), 1.35-0.95 (m, 4H). ^{13}C NMR (75 MHz; DMSO-*d*$_6$): δ 52.3 (CH), 33.3 (CH_2), 27.8 (CH_2), 25.7 (CH_2), 25.0 (CH_2), 24.5 (CH_2), 16.6 (CH_3). IR (film): 3259, 3248, 2930, 2858, 1728, 1639, 1616, 1558, 1435, 1217 cm^{-1}. HRMS (EI): Exact mass calcd for $C_{10}H_{15}N_3O_2$ [M]$^+$: 209.1164. Not found. LRMS m/z (relative intensity): 162.0 (3.8%), 143.1 (24.6%), 99.1 (24.6%), 56.1 (100%).

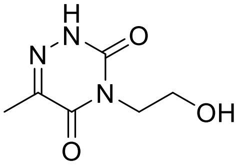

Table 8, entry 16h: 4-(2-hydroxyethyl)-6-methyl-1,2,4-triazine-3,5(2H,4H)-dione: Synthesized according to general procedure **5** using carbazone ester **15a** (0.177 g, 0.750 mmol), ethanolamine (0.0510 g, 0.830 mmol), and MeCN (2.5 mL). The crude mixture was purified by silica gel column chromatography using 85% EtOAc/hexanes to afford the pure compound as an amorphous white solid (0.610 g, 48%). TLC Rf = 0.30 in 85% EtOAc/hexanes. ^{1}H NMR (300 MHz; DMSO-*d*$_6$): δ 3.84 (t, *J* = 6.3 Hz, 2H), 3.52 (t, *J* = 6.3 Hz, 2H) 2.07 (s, 3H). ^{13}C NMR (75 MHz; DMSO-*d*$_6$): δ 156.6 (C), 149.6 (C), 141.8 (C), 57.1 (CH_2), 41.5 (CH_2) 16.6 (CH_3). IR

(film): 3242, 2918, 2851, 2355, 1734, 1726,, 1684, 1674, 1506, 1450, 1207 cm^{-1} . HRMS (EI): Exact mass calcd for $C_6H_9N_3O_3$ $[M]^+$: 171.0644. Found: 128.1 (– C_2H_5O).

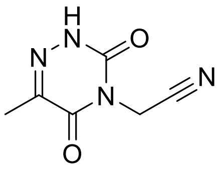

Table 8, entry 16i: 2-(6-Methyl-3,5-dioxo-2,3-dihydro-1,2,4-triazin-4(5H)-yl)acetonitrile: Synthesized according to general procedure **5** using carbazone ester **15a** (0.177 g, 0.750 mmol), aminoacetonitrile hydrochloride (0.0770g, 0.830 mmol), DIPEA (0.160 mL, 0.900 mmol), and MeCN (2.5 mL). The crude mixture was purified by silica gel column chromatography using 10% EtOAC/CH_2Cl_2 to afford the pure compound as an amorphous white solid (0.105 g, 84%). TLC Rf = 0.50 in 30% EtOAc/CH_2Cl_2. ^{1}H NMR (300 MHz; DMSO-d_6): δ 12.49 (br s, 1H), 4.79 (s, 2H), 2.11 (s, 3H). ^{13}C NMR (75 MHz; DMSO-d_6): δ 155.6 (C), 148.5 (C), 142.0 (C), 115.0 (C), 27.1 (CH_2), 16.5 (CH_3). IR (film): 2922, 2853, 1734, 1647, 1635, 1607, 1445, 1379, 1331, 1277, 1213, 1175 cm^{-1}. HRMS (EI): Exact mass calcd for $C_6H_8N_4O_3$ $[M]^+$: 166.0491. Found: 166.0498.

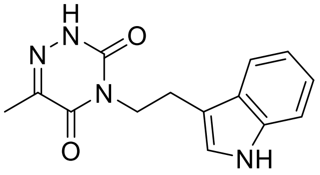

Table 8, entry 16j: 4-(2-(1H-Indol-3-yl)ethyl)-6-methyl-1,2,4-triazine-3,5(2H,4H)-dione: Synthesized according to general procedure **5** using carbazone ester **15a** (0.177 g, 0.750 mmol), tryptamine (0.133 g, 0.830 mmol), and MeCN (2.5 mL). The product was purified by filtration to afford the pure compound as an amorphous white solid (0.125 g, 61%). TLC Rf = 0.85 in 30% EtOAc/CH_2Cl_2. ^{1}H NMR (300 MHz; DMSO-d_6): δ 12.26 (br s, 1H), 10.86 (br s, 1H), 7.63 (d, J = 8.1 Hz, 1H), 7.35 (d, J = 8.1 Hz, 1H), 7.21 (d, J = 2.2 Hz, 1H), 7.11-6.98 (m, 2H), 4.04-3.98 (m, 2H), 2.97-2.92 (m, 2H), 2.10 (s, 3H). ^{13}C NMR (75 MHz; DMSO-d_6): δ 156.4 (C), 149.4 (C), 141.9 (C), 136.4 (C), 127.1 (C), 122.9 (CH), 121.0 (CH), 118.4 (CH), 118.1 (CH), 111.4 (CH), 110.6 (CH), 39.9 (CH_2), 22.8 (CH_2), 16.6 (CH_3). IR (film): 3344, 1715, 1649, 1636, 1448, 1348, 1285, 1229, 1159 cm^{-1}. HRMS (EI): Exact mass calcd for $C_{14}H_{14}N_4O_2$ $[M]^+$: 270.1117. Found: 270.1100.

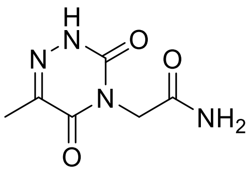

Table 8, entry 16k: 2-(6-Methyl-3,5-dioxo-2,3-dihydro-1,2,4-triazin-4(5H)-yl)acetamide: Synthesized according to general procedure **5** using carbazone ester **15a** (0.177 g, 0.750 mmol), glycinamide hydrochloride (0.0920 g, 0.830 mmol), DIPEA (0.16 mL, 0.90 mmol), and MeCN (2.5 mL). The crude mixture was purified by silica gel column chromatography using EtOAc to afford the pure compound as an amorphous white solid (0.0550 g, 35%). TLC Rf = 0.14 in EtOAc. ^{1}H NMR (300 MHz; DMSO-d_6): δ 12.31 (br s, 1H), 7.60 (br s, 1H), 7.18 (br s, 1H) 4.30 (s, 2H), 2.10 (s, 3H). ^{13}C NMR (75 MHz; DMSO-d_6): δ 167.6 (C), 156.2 (C), 149.3 (C), 141.8 (C), 41.5 (CH_2), 16.5 (CH_3). IR (film): 3205, 3194, 3159, 2957, 1772, 1701, 1647, 1628, 1445, 1398, 1375, 1290, 1205, 1178, 1165 cm^{-1}. HRMS (EI): Exact mass calcd for $C_6H_8N_4O_3$ $[M]^+$: 184.0596. No mass found: 162.0, 142.1, 128.1, 100.0, 83.0, 72.0, 56.0.

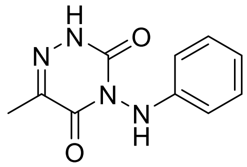

Table 8, entry 16l: 6-Methyl-4-(phenylamino)-1,2,4-triazine-3,5(2H,4H)-dione: Synthesized according to general procedure **5** using carbazone ester **15a** (0.177 g, 0.750 mmol), phenylhydrazine (0.082 mL, 0.830 mmol), and MeCN (2.5 mL). The crude mixture was purified by silica gel column chromatography using 15% EtOAc/CH_2Cl_2 to afford the pure compound as an amorphous white solid (0.0980 g, 60%). TLC Rf = 0.45 in 30% EtOAc/CH_2Cl_2. ^{1}H NMR (300 MHz; DMSO-d_6): δ 12.42 (br s, 1H), 8.53 (s, 1H) 7.17-7.14 (m, 2H). 6.78-6.69 (m, 3H), 2.14 (s, 3H). ^{13}C NMR (75 MHz; DMSO-d_6): δ 156.4 (C), 149.8 (C), 146.9 (C), 142.9 (C), 128.9 (CH), 119.9 (CH), 112.7 (CH), 16.9 (CH_3). IR (film): 3263, 2968, 2926, 1728, 1662, 1647, 1601, 1497, 1447, 1418, 1379, 1246, 1221, 1182 cm^{-1}. HRMS (EI): Exact mass calcd for $C_{10}H_{10}N_4O_2$ $[M]^+$: 218.0804 Found: 218.0816.

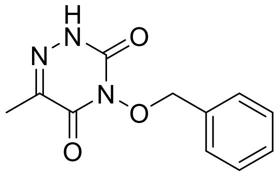

Table 8, entry 16m: 4-(Benzyloxy)-6-methyl-1,2,4-triazine-3,5(2H,4H)-dione: Synthesized according to general procedure **5** using carbazone ester **15a** (0.177 g, 0.750 mmol), *O*-benzyl hydroxylamine (0.102 g, 0.830 mmol), and MeCN (2.5 mL). The crude mixture was condensed *in vacuo* and the impurities were dissolved in CH_2Cl_2 before the product was purified by filtration to afford the pure compound as an amorphous white solid (0.105 g, 60%). TLC Rf = 0.71 in 30% EtOAc/CH_2Cl_2. ^{1}H NMR (300 MHz; DMSO-d_6): δ 12.42 (br s, 1H), 7.55-7.52 (m, 2H), 7.45-7.36 (m, 3H), 5.07 (s, 2H), 2.12 (s, 3H). ^{13}C NMR (75 MHz; DMSO-d_6): δ 153.9 (C),

148.1 (C), 143.3 (C), 134.1 (C), 129.5 (CH), 129.0 (CH), 128.4 (CH), 77.6 (CH_2), 16.5 (CH_3). IR (film): 3182, 3126, 2937, 1715, 1682, 1641, 1454, 1420, 1379, 1286, 1236, 1211, 1171 cm^{-1}. HRMS (EI): Exact mass calcd for $C_{11}H_{11}N_3O_3$ $[M]^+$: 233.0800. Not found. Calcd for $C_4H_5N_3O_2$ $[M+H]^+$: 128.0460 Found: 128.0468 (M – OBn).

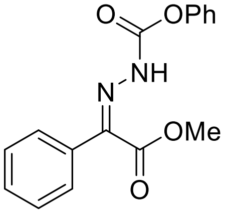

Carbazone 15b: (*Z*)-Phenyl-2-(2-methoxy-2-oxo-1-phenylethylidene)hydrazinecarboxylate: To a solution of methyl benzolyformate (0.821g, 5.00 mmol) in MeOH (25 mL) was added phenyl carbazate (0.761 g, 5.00 mmol) and the solution was stirred overnight at 60 °C. The crude mixture was purified by recrystalisation with ether to afford the pure product as an amorphous white solid (1.25 g, 84%). TLC Rf =0.25 in 20% EtOAc/hexanes. ^{1}H NMR (300 MHz; DMSO-d_6): δ 11.67 (br s, 1H), 7.64-7.58 (m, 2H), 7.48-7.42 (m, 5H), 7.32-7.24 (m, 3H) 3.94 (s, 3H). ^{13}C NMR (75 MHz; DMSO-d_6): δ 163.1 (C), 151.9 (C), 150.3 (C), 130.1 (C), 130.0 (CH), 129.6 (CH), 128.7 (CH), 126.7 (CH), 125.8 (CH), 121.8 (CH), 53.0 (CH_3). IR (film): 1770, 1734, 1719, 1628, 1475, 1458, 1437, 1325, 1265, 1231, 1134 cm^{-1}. HRMS (EI): Exact mass calcd for $C_{16}H_{14}N_2O_4$ $[M]^+$: 298.0954. Found: 298.0927.

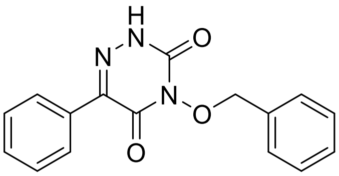

Table 8, entry 16n: 4-(Benzyloxy)-6-phenyl-1,2,4-triazine-3,5(2H,4H)-dione: Synthesized according to general procedure **5** using carbazone ester **15b** (0.224 g, 0.750 mmol), *O*-benzyl hydroxylamine (0.102 g, 0.830 mmol), and MeCN (2.5 mL). The crude mixture was condensed *in vacuo* and the impurities were dissolved in CH_2Cl_2 before the product was purified by filtration to afford the pure compound as an amorphous white solid (0.155 g, 70%). TLC Rf = 0.85 in 30% EtOAc/CH_2Cl_2. ^{1}H NMR (300 MHz; DMSO-d_6): δ 12.93 (br s, 1H), 7.90-7.84 (m, 2H), 7.61-7.53 (m, 2H), 7.49-7.38 (m, 6H), 5.14 (s, 2H). ^{13}C NMR (75 MHz; DMSO-d_6): δ 153.4 (C), 147.8 (C), 134.1 (C), 132.3 (C), 129.6 (CH), 129.6 (CH), 129.0 (CH), 128.4 (CH), 128.1 (CH), 128.0 (CH), 77.6 (CH_2). IR (film): 3242, 3182, 2918, 1755, 1747, 1734, 1680, 1553, 1493, 1445, 1404, 1238, 1176 cm^{-1}. HRMS (EI): Exact mass calcd for $C_{16}H_{13}N_3O_3$ $[M]^+$: 295.0957. Found: 295.0962.

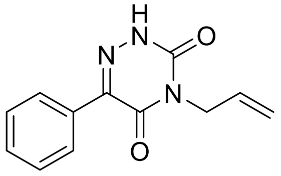

Table 8, entry 16o: 4-Allyl-6-phenyl-1,2,4-triazine-3,5(2H,4H)-dione: Synthesized according to general procedure **5** using carbazone ester **15b** (0.224 g, 0.750 mmol), allylamine (0.060 mL, 0.83 mmol), and MeCN (2.5 mL). The crude mixture was purified by silica gel column chromatography using 20% EtOAc/hexanes to afford the pure compound as an amorphous yellow solid (0.100 g, 58%). TLC Rf = 0.22 in 20% EtOAc/hexanes. ^{1}H NMR (300 MHz; $CDCl_3$): δ 9.42 (br s, 1H), 7.99-7.93 (m, 2H), 7.48-7.41 (m, 3H), 5.94 (ddt, *J* = 17.1, 10.2, 6.1 Hz, 1H), 5.42-5.27 (m, 2H), 4.62 (dt, *J* = 6.1, 1.4 Hz, 2H). ^{13}C NMR (75 MHz; $CDCl_3$): δ 155.2 (C), 149.8 (C), 142.3 (C), 131.7 (C), 130.2 (CH), 130.1 (C), 128.3 (C), 128.3 (C), 119.6 (CH_2), 42.6 (CH_2). IR (film): 3242, 3207, 3105, 3091, 2988, 1713, 1651, 1643, 1558, 1495, 1441, 1297, 1223, 1178, 1124 cm^{-1}. HRMS (EI): Exact mass calcd for $C_{12}H_{11}N_3O_2$ $[M]^+$: 229.0851. Found: 229.0839.

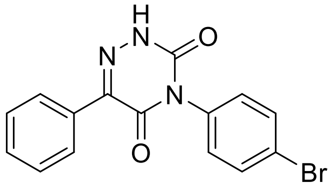

Table 8, entry 16p: 4-(4-Bromophenyl)-6-phenyl-1,2,4-triazine-3,5(2H,4H)-dione: Synthesized according to general procedure **5** using carbazone ester **15b** (0.224 g, 0.750 mmol), 3-bromoaniline (0.143 g, 0.830 mmol), and MeCN (2.5 mL). The crude mixture was purified by filtration to afford the pure compound as an amorphous white solid (0.200 g, 77%). TLC Rf = 0.85 in $EtOAc/CH_2Cl_2$. ^{1}H NMR (300 MHz; DMSO-d_6): δ 12.84 (br s, 1H), 7.90-7.84 (m, 2H), 7.75-7.70 (m, 2H), 7.48-7.43 (m, 3H), 7.39-7.34 (m, 2H). ^{13}C NMR (75 MHz; DMSO-d_6): δ 155.7 (C), 148.9 (C), 141.3 (C), 133.2 (C), 132.6 (C), 132.0 (CH), 130.9 (CH), 129.5 (CH), 128.1 (CH), 121.9 (C). IR (film): 2920, 2862, 1724, 1647, 1632, 1558, 1489, 1445, 1302, 1232, 1186, 1068 cm^{-1}. HRMS (EI): Exact mass calcd for $C_{15}H_{10}Br_1N_3O_2$ $[M]^+$: 342.9956. Found: 342.9956.

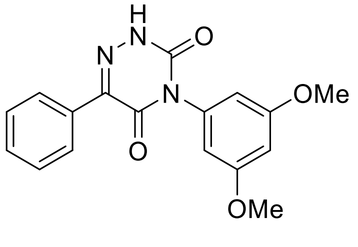

Table 8, entry 16q: 4-(3,5-Dimethoxyphenyl)-6-phenyl-1,2,4-triazine-3,5(2H,4H)-dione: Synthesized according to general procedure **5** using carbazone ester **15b** (0.224 g, 0.750 mmol), 3,5-dimethoxyaniline (0.127 g, 0.830 mmol), and MeCN (2.5 mL). The crude mixture was purified by filtration to afford the pure compound as an amorphous white solid (0.220 g, 90%).

TLC Rf = 0.85 in 30% EtOAc/CH_2Cl_2. ^{1}H NMR (300 MHz; DMSO-d_6): δ 12.79 (br s, 1H), 7.88 (br s, 2H), 7.45 (br s, 3H), 6.60 (br s, 3H), 3.75 (br s, 6H). ^{13}C NMR (75 MHz; DMSO-d_6): δ 160.6 (C), 155.7 (C), 148.9 (C), 141.3 (CH), 135.6 (C), 132.7 (C), 129.4 (CH), 128.1 (CH), 109.6 (CH), 100.5 (CH), 55.4 (CH_3). IR (film): 1715, 1564, 1444, 1370, 1290, 1265, 1118, 1065 cm^{-1}. HRMS (EI): Exact mass calcd for $C_{17}H_{15}N_3O_4$ $[M]^+$: 325.1063. Found: 325.1075.

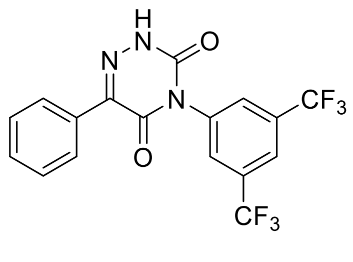

Table 8, entry 16r: 4-(3,5-Bis(trifluoromethyl)phenyl)-6-methyl-1,2,4-triazine-3,5(2H,4H)-dione: Synthesized according to general procedure **5** using carbazone ester **15b** (0.224 g, 0.750 mmol), 3,5-bistrifluoromethylaniline (0.190 g, 0.830 mmol), and MeCN (2.5 mL). The crude mixture was purified by filtration to afford the pure compound as an amorphous white solid (0.250 g, 83%). TLC Rf = 0.9 in 30 % EtOAc/CH_2Cl_2. ^{1}H NMR (300 MHz; DMSO-d_6): δ 13.03 (br s, 1H), 8.31-8.27 (m, 3H), 7.93-7.87 (m, 2H), 7.50-7.45 (m, 3H). ^{13}C NMR (75 MHz; DMSO-d_6): δ 155.7 (C), 148.8 (C), 141.4 (C), 136.0 (C), 132.4 (C), 131.3 (C), 130.9 (C), 130.4 (CH), 130.3 (CH), 129.7 (CH), 128.2 (CH), 128.1 (CH). IR (film): 1770, 1718, 1684, 1558, 1472, 1437, 1373, 1265, 1225, 1177, 1136 cm^{-1}. HRMS (EI): Exact mass calcd for $C_{17}H_{91}N_3O_2$ $[M]^+$: 401.0599. Found: 400.9956.

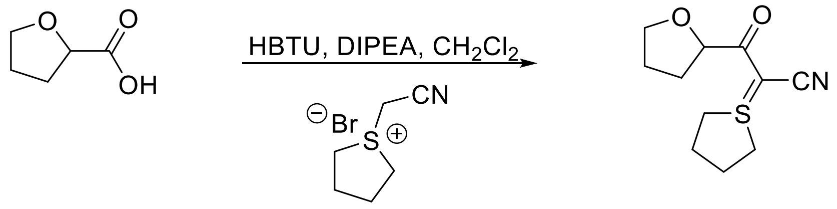

Adaptation of a known literature preparation[6]: Tetrahydro-2-furoic acid (1.16 g, 10.0 mmol) was dissolved in CH_2Cl_2 (100 mL). HBTU (4.18 g, 11.0 mmol), DIPEA (5.29 mL, 30.0 mmol), and 1- (cyanomethyl)tetrahydro-1H-thiophenium bromide salt (2.29 g, 13.0 mmol) were added and the reaction was stirred at room temperature for 3 hours. The reaction was poured into saturated aqueous NH_4Cl (100 mL) and the aqueous layer was extracted with CH_2Cl_2 (3 x 100 mL). The combined organic layers were washed with brine, dried over $MgSO_4$, filtered, and concentrated. The crude oil was purified over silica gel by flash column chromatography using a gradient of 1:1 EtOAc:acetone to acetone to yield a white solid (2.10 g, 91%). TLC Rf = 0.32 in acetone. ^{1}H NMR (300 MHz; DMSO-d_6): δ 4.41-4.37 (m, 1H), 3.86-3.77 (m, 1H), 3.75-3.68 (m, 1H). 3.60-3.51 (m, 2H), 3.14-3.01 (m, 2H) 2.37-2.21 (m, 2H), 2.07-1.97 (m, 3H), 1.87-1.73 (m, 3H). ^{13}C NMR (75 MHz; DMSO-d_6): δ 189.8 (C), 119.9 (C), 79.7 (CH), 68.3 (CH_2), 45.5 (CH_2), 45.2 (CH_2), 29.8 (CH_2), 28.0 (CH_2), 28.0 (CH_2), 25.4 (CH_2).

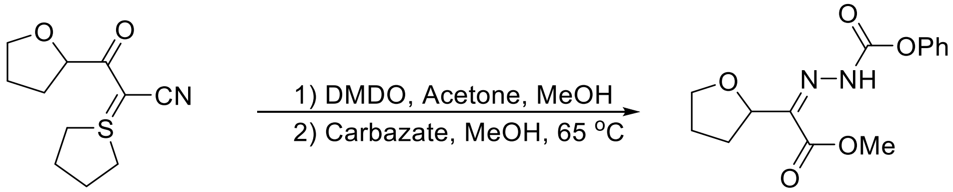

The ylide (0.225 g, 1.00 mmol) was dissolved in MeOH (10 mL). A solution of oxone in acetone (0.1 M, 20 mL, 2 mmol) was added slowly and the solution was stirred for 1 hour. An additional 5 mL were added and the solution was stirred for another hour. The solution was condensed under reduced pressure and the crude oil was dissolved in MeOH (5 mL) and phenyl carbazate (0.152 g, 1.00 mmol) was added. The solution was stirred at reflux overnight. The solution was concentrated and purified by silica gel flash column chromatography with 5% EtOAc:CH_2Cl_2 to afford the impure semi-carbazone **15c** as a colorless oil (0.215 g, 74% , overestimated since material is not pure).

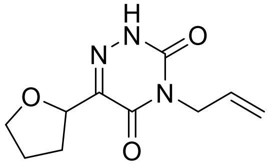

Table 8, entry 16s: 4-Allyl-6-(tetrahydrofuran-2-yl)-1,2,4-triazine-3,5(2H,4H)-dione: Synthesized according to general procedure **5** using impure carbazone ester **15c** (0.190 g, 0.650 mmol), allylamine (0.050 mL, 0.72 mmol), and MeCN (2.0 mL). The crude mixture was purified by silica gel column chromatography using 20% EtOAc/CH_2Cl_2 to afford the pure compound as an amorphous white solid (0.0600 g, 42%). TLC Rf = 0.18 in 20% EtOAc/CH_2Cl_2. ^{1}H NMR (300 MHz; DMSO-d_6): δ 12.46 (br s, 1H), 5.81 (ddt, J = 17.5, 10.1, 5.3 Hz, 1H), 5.15 (q, J = 1.4 Hz, 1H), 5.10 (dq, J = 4.8, 1.4 Hz, 1H), 4.87 (t, J = 6.8 Hz, 1H), 4.35 (dt, J = 5.3, 1.5 Hz, 2H), 3.86-3.72 (m, 2H), 2.11-1.82 (m, 4H). ^{13}C NMR (75 MHz; DMSO-d_6): δ 149.0 (C), 143.6 (C), 131.5 (CH), 117.2 (CH_2), 75.0 (CH), 68.0 (CH_2), 41.2 (CH_2), 28.6 (CH_2), 25.4 (CH_2). IR (film): 3225, 2988, 2870, 1717, 1659, 1645, 1558, 1456, 1425, 1406, 1337, 1225, 1173, 1065 cm^{-1}. HRMS (EI): Exact mass calcd for $C_{10}H_{13}N_3O_3$ [M]$^+$: 223.0957. Found: 223.0960.

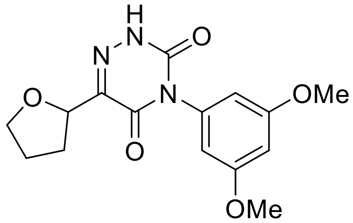

Table 8, entry 16t: 4-(3,5-Dimethoxyphenyl)-6-(tetrahydrofuran-2-yl)-1,2,4-triazine-3,5(2H,4H)-dione: Synthesized according to general procedure **5** using impure carbazone ester **15c** (0.175 g, 0.600 mmol), 3,5-dimethoxyaniline (0.102 g, 0.660 mmol), and MeCN (2.0 mL). The crude mixture was purified by silica gel column chromatography using 20% EtOAc/CH_2Cl_2 to afford the pure compound as an amorphous white solid (0.110 g, 57%). TLC Rf = 0.21 in 20% EtOAc/CH_2Cl_2. ^{1}H NMR (300 MHz; DMSO-d_6): δ 12.48 (br s, 1H), 6.56-6.57 (m, 1H), 6.54-

6.43 (m, 2H), 4.90 (t, J = 6.7 Hz, 1H), 4.35 (dt, J = 5.3, 1.5 Hz, 2H), 3.88-3.74 (m, 8H), 2.13-1.80 (m, 4H). ^{13}C NMR (75 MHz; DMSO-d_6): δ 160.6 (C), 155.4 (C), 149.0 (C), 144.2 (C), 135.2 (C), 106.8 (CH), 100.6 (CH), 75.0 (CH), 68.0 (CH_2), 55.4 (CH_3), 28.7 (CH_2), 25.4 (CH_2). IR (film): 3286, 3246, 2959, 1734, 1670, 1610, 1597, 1558, 1474, 1429, 1346, 1205, 1155 cm^{-1}. HRMS (EI): Exact mass calcd for $C_{15}H_{17}N_3O_5$ $[M]^+$: 319.1168. Found: 319.1159.

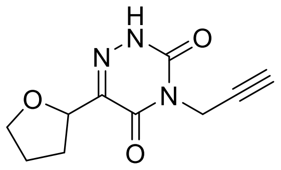

Table 8, entry 16u: 4-(Prop-2-yn-1-yl)-6-(tetrahydrofuran-2-yl)-1,2,4-triazine-3,5(2H,4H)-dione: Synthesized according to general procedure **5** using impure carbazone ester **15c** (0.175 g, 0.600 mmol), propargylamine (0.0360 g, 0.660 mmol), and MeCN (2.0 mL). The crude mixture was purified by silica gel column chromatography using 20% EtOAc/CH_2Cl_2 to afford the pure compound as an amorphous white solid (0.0700 g, 42%). TLC Rf = 0.20 in 20% EtOAc/CH_2Cl_2. ^{1}H NMR (300 MHz; DMSO-d_6): δ 12.59 (br s, 1H), 4.87 (t, J = 6.7 Hz, 1H), 4.48 (d, J = 2.2 Hz, 2H), 3.86-3.72 (m, 2H), 3.19 (s, 1H) 2.10-1.82 (m, 4H). ^{13}C NMR (75 MHz; DMSO-d_6): δ 154.5 (C), 148.5 (C), 143.6 (C), 78.0 (C), 75.1 (CH), 73.7 (CH), 28.6 (CH_2), 28.5 (CH_2), 25.4 (CH_2). IR (film): 3244, 2924, 1713, 1655, 1620, 1531, 1452, 1425, 1346, 1242, 1223, 1167, 1055 cm^{-1}. HRMS (EI): Exact mass calcd for $C_{10}H_{11}N_3O_3$ $[M]^+$: 221.0800. Found: 221.0784.

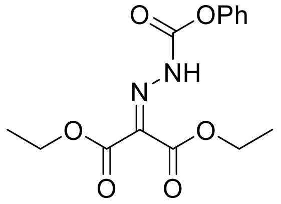

Carbazone 15d: Diethyl 2-(2-(phenoxycarbonyl)hydrazono)malonate: To a solution of diethylketomalonate (0.46 mL, 3.0 mmol) in MeOH (15 mL) was added phenyl carbazate (0.456 g, 3.00 mmol) and the solution was stirred overnight at 60 °C. The crude mixture was purified by silica gel column chromatography using 30% EtOAc/hexanes to afford the pure product as an amorphous white solid (0.404 g, 44%). TLC Rf = 0.52 in 30% EtOAc/hexanes. ^{1}H NMR (300 MHz; DMSO-d_6): δ 12.06 (br s, 1H), 7.47-7.41 (m, 2H), 7.32-7.22 (m, 3H), 4.36-4.22 (m, 4H) 1.29-1.21 (m, 6H). ^{13}C NMR (75 MHz; DMSO-d_6): δ 161.4 (C), 160.7 (C), 151.6 (C), 150.2 (C), 134.0 (C), 130.0 (CH), 126.5 (CH), 121.9 (CH), 62.7 (CH_2), 62.3 (CH_2), 14.1 (CH_3), 13.9 (CH_3). IR (film): 3252, 2984, 2355, 1784, 1732, 1697, 1558, 1474, 1369, 1256, 1140, 1090 cm^{-1}. HRMS (EI): Exact mass calcd for $C_{14}H_{16}N_2O_6$ $[M]^+$: 308.1008. Found: 308.1039.

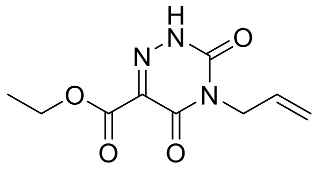

Table 8, entry 16v: Ethyl 4-allyl-3,5-dioxo-2,3,4,5-tetrahydro-1,2,4-triazine-6-carboxylate: Synthesized according to general procedure **5** using carbazone ester **15d** (0.231 g, 0.750 mmol), allylamine (0.060 mL, 0.83 mmol), and MeCN (2.5 mL). The crude mixture was purified by silica gel column chromatography using 30% EtOAc/hexanes to afford the pure compound as an amorphous yellow solid (0.108 g, 64%). TLC Rf = 0.17 in 30% EtOAC/hexanes. ^{1}H NMR (300 MHz; $CDCl_3$): δ 10.68 (br s, 1H), 5.86 (ddt, *J* = 16.9, 10.3, 6.2 Hz, 1H), 5.39-5.26 (m, 2H), 4.54 (d, *J* = 6.1 Hz, 2H), 4.44 (q, *J* = 7.2 Hz, 2H), 1.40 (t, *J* = 7.1 Hz, 3 H). ^{13}C NMR (75 MHz; $CDCl_3$): δ 160.3 (C), 152.6 (C), 148.8 (C), 134.9 (C), 129.4 (CH), 120.2 (CH_2), 62.7 (CH_2), 42.7 (CH_2), 14.0 (CH_3). IR (film): 3267, 3001, 1717, 1670, 1647, 1578, 1431, 1408, 1317, 1242, 1136, 1014 cm^{-1}. HRMS (EI): Exact mass calcd for $C_9H_{11}N_3O_4$ $[M]^+$: 225.0750. Found: 225.0781.

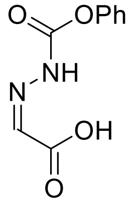

Carbazone 15e' (*Z*)-2-(2-(Phenoxycarbonyl)hydrazono)acetic acid: To a solution of glycoxylic acid monohydrate (0.460 g, 5.00 mmol) in MeOH (25 mL) was added phenyl carbazate (0.761 g, 5.00 mmol) and the solution was stirred overnight. The crude mixture was purified by recrystalisation with ether to afford the pure product as an amorphous white solid (0.953 g, 92%). TLC Rf = 0.64 in 30% EtOAc/CH_2Cl_2. ^{1}H NMR (300 MHz; DMSO-d_6): δ 13.12 (br s, 1H), 12.13 (br s, 1H), 7.46-7.40 (m, 3H), 7.30-7.20 (m, 3H). ^{13}C NMR (75 MHz; DMSO-d_6): δ 164.3 (C), 129.6 (CH), 125.9 (CH), 121.8 (CH). IR (film): 3410, 3005, 2991, 1759, 1724, 1653, 1636, 1558, 1495, 1474, 1423 cm^{-1}. HRMS (EI): Exact mass calcd for $C_{16}H_{14}N_2O_4$ $[M]^+$: 208.0489. Not found. LRMS m/z (relative intensity) 181.0 (24.6%), 162.0 (2.3%), 112.0 (1.5%), 94.0 (69.2%), 69.0 (100%).

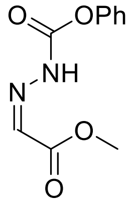

Carbazone 15: (*Z*)-Phenyl 2-(2-methoxy-2-oxoethylidene)hydrazinecarboxylate: To a solution of carbazone **15e'** (0.208 g, 1.00 mmol) in CH_2Cl_2 (2.5 mL) was added *N,N'*-dicyclohexylcarbodiimide (0.206 g, 1.00 mmol), 4-dimethylaminopyridine (0.0120 g, 0.100 mmol), and MeOH (0.080 mL, 2.0 mmol) and the solution was stirred overnight. The crude mixture was purified by silica gel column chromatography using 6% EtOAc/CH_2Cl_2 to afford the pure product as an amorphous white solid (0.185 g, 83%). TLC Rf = 0.20 in 6% EtOAc/CH_2Cl_2. ^{1}H NMR (300 MHz; DMSO-d_6): δ 12.23 (br s, 1H), 7.51-7.37 (m, 3H), 7.31-7.18 (m, 3H). ^{13}C NMR (75 MHz; DMSO-d_6): δ 163.0 (C), 129.6 (CH), 125.9 (CH), 121.7 (CH), 52.0 (CH_2). IR (film): 1713, 1597, 1555, 1537, 1493, 1437, 1350, 1196, 1132, 1047 cm^{-1}. HRMS (EI): Exact mass calcd for $C_{10}H_{10}N_2O_4$ $[M]^+$: 222.0641. Found: 222.0624.

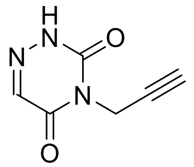

Table 8, entry 16w: 4-(Prop-2-yn-1-yl)-1,2,4-triazine-3,5(2H,4H)-dione: Synthesized according to general procedure **5** using carbazone ester **15e** (0.167 g, 0.750 mmol), propargylamine (0.0460 g, 0.830 mmol), and MeCN (2.5 mL). The crude mixture was purified by silica gel column chromatography using 30% EtOAc/hexanes to afford the pure compound as an amorphous white solid (0.0700 g, 62%). TLC Rf = 0.14 in 30% EtOAc/hexanes. ^{1}H NMR (300 MHz; DMSO-d_6): δ 12.71 (br s, 1H), 7.56 (s, 1H), 4.47 (d, J = 2.5 Hz, 2H). 3.19 (t, J = 2.5 Hz, 1H),). ^{13}C NMR (75 MHz; DMSO-d_6): δ 154.8 (C), 148.8 (C), 135.2 (CH), 74.1 (CH), 28.9 (CH_2). IR (film): 3285, 3246, 2912, 1730, 1655, 1593, 1558, 1429, 1342, 1209, 1151, 1107 cm^{-1}. HRMS (EI): Exact mass calcd for $C_6H_5N_3O_2$ $[M]^+$: 151.0382. Found: 151.0391.

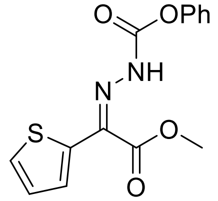

Carbazone 15f: (*E*)-Phenyl-2-(2-methoxy-2-oxo-1-(thiophen-2-yl)ethylidene)hydrazine carboxylate: Synthesized according to a known procedure[5] using 2-oxo-2-(2-thienyl)acetic acid (0.500 g, 3.20 mmol). The crude product was directly reacted with phenyl carbazate (0.442 g, 2.91 mmol) in CH_3OH (15 mL) at 65 °C for 16 hours. The crude mixture was purified by silica gel flash column chromatography using 20% EtOAc/pet. ether. TLC Rf = 0.35 in 20% EtOAc/pet. ether. ^{1}H NMR (300 MHz; $CDCl_3$): δ 11.9 (br s, 1H), 7.61 (dd, J = 3.8, 1.2 Hz, 1H), 7.41-7.33 (m, 3H), 7.24-7.21 (m, 3H), 7.02 (dd, J = 5.1, 3.8 Hz, 1H), 3.99 (s, 3H). ^{13}C NMR (75 MHz; $CDCl_3$): δ 161.9 (C), 150.7 (C), 130.1 (C), 129.6 (CH), 129.2 (CH), 128.5 (CH), 127.5 (CH), 126.0 (CH), 121.5 (CH), 53.1 (CH_3). IR (film): 3269, 1772, 1733, 1701, 1647, 1515, 1455, 1318, 1266, 1164, 1137, 1029 cm^{-1}. HRMS (EI): Exact mass calcd for $C_{14}H_{12}N_2O_4S$ $[M]^+$: 304.0518. Found: 304.0506.

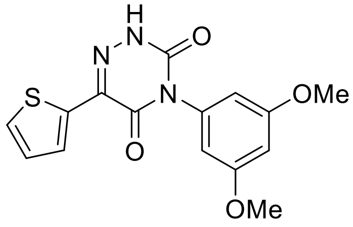

Table 8, entry 16x: 4-(3,5-Dimethoxyphenyl)-6-(thiophen-2-yl)-1,2,4-triazine-3,5(2H,4H)-dione: Synthesized according to general procedure **5** using carbazone ester **15f** (0.091 g, 0.300 mmol), 3,5-dimethoxyaniline (0.051 g, 0.330 mmol), and MeCN (1.0 mL). The crude mixture was purified by filtration to afford the pure compound as an amorphous white solid (0.220 g,

90%). TLC Rf = 0.30 in 10% EtOAc/CH_2Cl_2.. ^{1}H NMR (300 MHz; DMSO-d_6): δ 12.70 (s, 1H), 7.89 (dd, J = 3.7, 1.2 Hz, 1H), 7.65 (dd, J = 5.1, 1.2 Hz, 1H), 7.12 (dd, J = 5.1, 3.7 Hz, 1H), 6.56 (s, 3H), 3.71 (s, 6H). ^{13}C NMR (75 MHz; DMSO-d_6): δ 160.6 (C), 154.9 (C), 148.7 (C), 137.1 (C), 135.4 (C), 135.2 (C), 128.9 (CH), 128.1 (CH), 127.7 (CH), 106.8 (CH), 100.6 (CH), 55.4 (CH_3). IR (film): 1772, 1733, 1716, 1668, 1647, 1558, 1455, 1265, 1188, 1141 cm^{-1}. HRMS (EI): Exact mass calcd for $C_{15}H_{13}N_3O_4S$ $[M]^+$: 331.0627. Found: 331.0647.

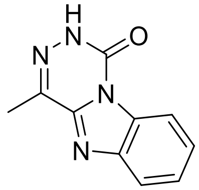

Table 9, entry 1: 4-Methylbenzo[4,5]imidazo[1,2-d][1,2,4]triazin-1(2H)-one: Synthesized according to general procedure **5** using carbazone ester 15a (0.177 g, 0.750 mmol), 1,2-phenylenediamine (0.0900 g, 0.830 mmol), and MeCN (2.5 mL). The crude mixture was purified by filtration to afford the pure compound as an amorphous white solid (0.100 g, 67%). TLC Rf = 0.80 in 30% EtOAc/CH_2Cl_2. ^{1}H NMR (300 MHz; DMSO-d_6): δ 8.33-8.27 (m, 1H), 7.96-7.90 (m, 1H) 7.60-7.51 (m, 2H), 2.53 (s, 3H). ^{13}C NMR (75 MHz; DMSO-d_6): δ 146.1 (C), 143.0 (C), 139.4 (C), 129.3 (C), 126.2 (CH), 125.3 (CH), 120.5 (CH), 115.0 (CH), 108.7 (C), 16.8 (CH_3). IR (film): 2920, 2854, 2372, 1716, 1705, 1653, 1569, 1366, 1151 cm^{-1}. HRMS (EI): Exact mass calcd for $C_{10}H_8N_4O$ $[M]^+$: 200.0698. Found: 200.0668.

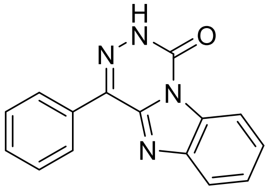

Table 9, entry 2: 4-Phenylbenzo[4,5]imidazo[1,2-d][1,2,4]triazin-1(2H)-one: Synthesized according to general procedure **5** using carbazone ester **15b** (0.224 g, 0.750 mmol), 1,2-phenylenediamine (0.0900 g, 0.830 mmol), and MeCN (2.5 mL). The crude mixture was purified by filtration to afford the pure compound as an amorphous white solid (0.150 g, 76%). TLC Rf = 0.80 in 30% EtOAc/CH_2Cl_2. ^{1}H NMR (300 MHz; DMSO-d_6): δ 13.17 (br s, 1H), 8.44-8.36 (m, 3H), 8.03-7.98 (m, 1H) 7.63-7.50 (m, 5H). ^{13}C NMR (75 MHz; DMSO-d_6): δ 146.6 (C), 143.0 (C), 141.8 (C), 138.1 (C), 132.6 (C), 129.9 (CH), 128.9 (C), 128.4 (CH), 128.0 (C), 126.2 (CH), 125.3 (CH), 120.4 (CH), 115.0 (CH). IR (film): 2941, 1742, 1707, 1647, 1558, 1522, 1441, 1373, 1277, 1211. HRMS (EI): Exact mass calcd for $C_{15}H_{10}N_4O$ $[M]^+$: 262.0855. Found: 262.0840.

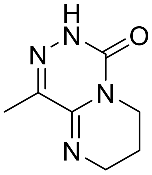

Table 9, entry 3: 9-Methyl-3,4-dihydro-2H-pyrimido[1,2-d][1,2,4]triazin-6(7H)-one: Synthesized according to general procedure **5** using carbazone ester **15a** (0.177 g, 0.750 mmol), diaminopropane (0.070 mL, 0.83 mmol), and MeCN (2.5 mL). The crude mixture was purified by silica gel column chromatography using 50% EtOAc/CH_2Cl_2 to pure EtOAc to afford the pure compound as an amorphous white solid (0.0800 g, 64%). TLC Rf = 0.08 in 50% EtOAc/CH_2Cl_2. ^{1}H NMR (300 MHz; DMSO-d_6): δ 11.27 (br s, 1H), 3.63 (t, J = 5.9 Hz, 2H) 3.42 (t, J = 5.3 Hz, 2H), 1.97 (s, 3H), 1.75 (quint., J = 5.7 Hz, 2 H). ^{13}C NMR (75 MHz; DMSO-d_6): δ 148.4 (C), 142.8 (C), 142.0 (C), 43.4 (CH_2), 36.6 (CH_2), 19.0 (CH_2), 17.3 (CH_3). IR (film): 1684, 1626, 1551, 1433, 1373, 1315, 1265, 1194. HRMS (EI): Exact mass calcd for $C_7H_{10}N_4O$ $[M]^+$: 166.0855. Found: 166.0847.

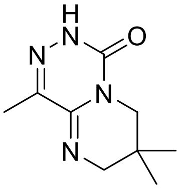

Table 9, entry 4: 3,3,9-Trimethyl-3,4-dihydro-2H-pyrimido[1,2-d][1,2,4]triazin-6(7H)-one: Synthesized according to general procedure **5** using carbazone ester **15a** (0.177 g, 0.750 mmol), 2,2-dimethyl-1,3-diaminopropane (0.850 g, 0.830 mmol), and MeCN (2.5 mL). The crude mixture was purified by silica gel column chromatography using 50% EtOAc/CH_2Cl_2 to afford the pure compound as an amorphous white solid (0.105 g, 72%). TLC Rf = 0.21 in 50% EtOAc/CH_2Cl_2. ^{1}H NMR (300 MHz; DMSO-d_6): δ 11.32 (br s, 1H), 3.33 (s, 2H) 3.15 (s, 2H), 2.00 (s, 3H), 0.89 (s, 6H). ^{13}C NMR (75 MHz; DMSO-d_6): δ 148.6 (C), 142.6 (C), 141.0 (C), 55.6 (CH_2), 48.7 (CH_2), 25.8 (C), 24.1 (CH_3) 17.4 (CH_3). IR (film): 3225, 3082, 2964, 1684, 1676, 1622, 1558, 1475, 1437, 1375, 1300, 1265, 1155. HRMS (EI): Exact mass calcd for $C_9H_{14}N_4O$ $[M]^+$: 194.1168. Found: 194.1183.

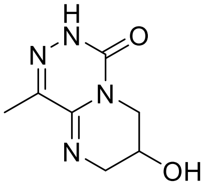

Table 9, entry 5: 3-Hydroxy-9-methyl-3,4-dihydro-2H-pyrimido[1,2-d][1,2,4]triazin-6(7H)-one: Synthesized according to general procedure **5** using carbazone ester **15a** (0.177 g, 0.750 mmol), 1,3-diamino-2-propanol (0.750 g, 0.830 mmol), and MeCN (2.5 mL). The crude mixture was purified by removing soluble by-products in CH_2Cl_2 to afford the pure compound as an amorphous white solid (0.0750 g, 55%). TLC Rf = 0.03 in 50 % EtOAc/CH_2Cl_2. ^{1}H NMR (300 MHz; DMSO-d_6): δ 11.27 (br s, 1H), 5.16-5.08 (m, 1H) 3.98 (br s, 1H), 3.71-3.62 (m, 1H), 3.54-

3.43 (m, 2H), 3.38-3.27 (m, 2H), 1.99 (s, 3H).^{13}C NMR (75 MHz; DMSO-d_6): δ 148.8 (C), 142.6 (C), 141.5 (C), 58.7 (CH), 50.0 (CH_2), 44.2 (CH_2), 17.3 (CH_3). IR (film): 1716, 1662, 1617, 1558, 1434, 1427, 1418, 1265, 1236, 1225, 1177. HRMS (EI): Exact mass calcd for $C_7H_{10}N_4O_2$ $[M]^+$: 182.0804. Found: 182.0821.

References

1. Ketone synthesized according to: D. Arnold, M. LaPorte, S. Anderson and P. Wipf, *Tetrahedron*, 2013, **69**, 7731.
2. Ketone synthesized according to: B. Willy, F. Rominger and T. Muller, *Synthesis,* 2008, **2**, 303.
3. Ketone synthesized according to: R. Cox, D. Ritson, T. Dane, J. Berge, J. Charmant and A. Kantacha, *Chem. Commun.*, 2005, 1039.
4. Ketone synthesized according to: J. Waldo and R. Larock, *Org. Lett.*, 2005, **7**, 5205.
5. Monsanto Technology LLC Patent: US6359156 B1, *2002*; Location in patent: Page column 11-12.
6. L. Ju, A. L. Lippert and J. W. Bode, J. *Am. Chem. Soc*., 2008, **130**, 4253-4255.
7. C. Clavette, J.-F. Vincent-Rocan, and A. M. Beauchemin, *Angew. Chem. Int. Ed.,* 2013, **52**, 12705
8. J.-F Vincent-Rocan, C. Clavette, K. Leckett and A. M. Beauchemin, *Chem. Eur. J.*, 2015, **21**, 3886.

Spectra

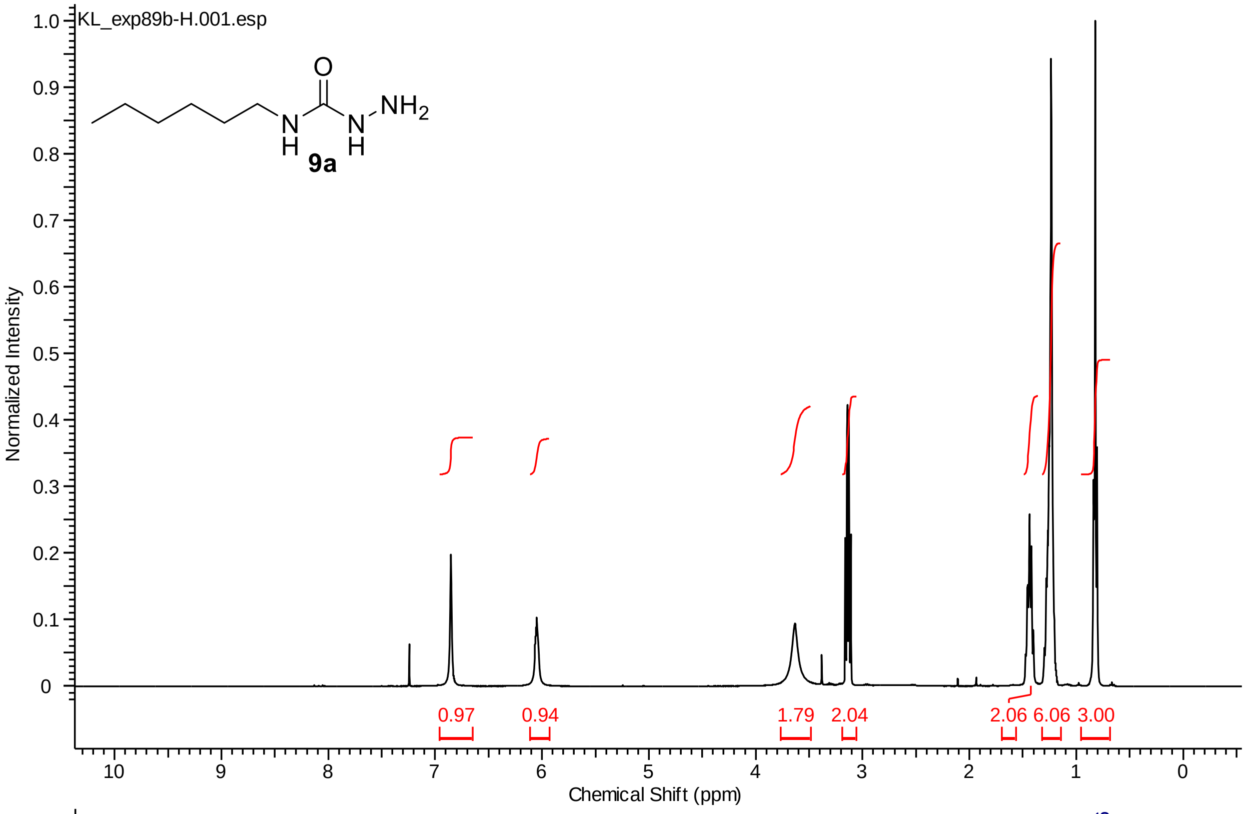
KL_exp89b-H.001.esp
O
NH2
N
H
N
H
9a
0.97
0.94
1.79
2.04
2.06
6.06
3.00
Normalized Intensity
Chemical Shift (ppm)

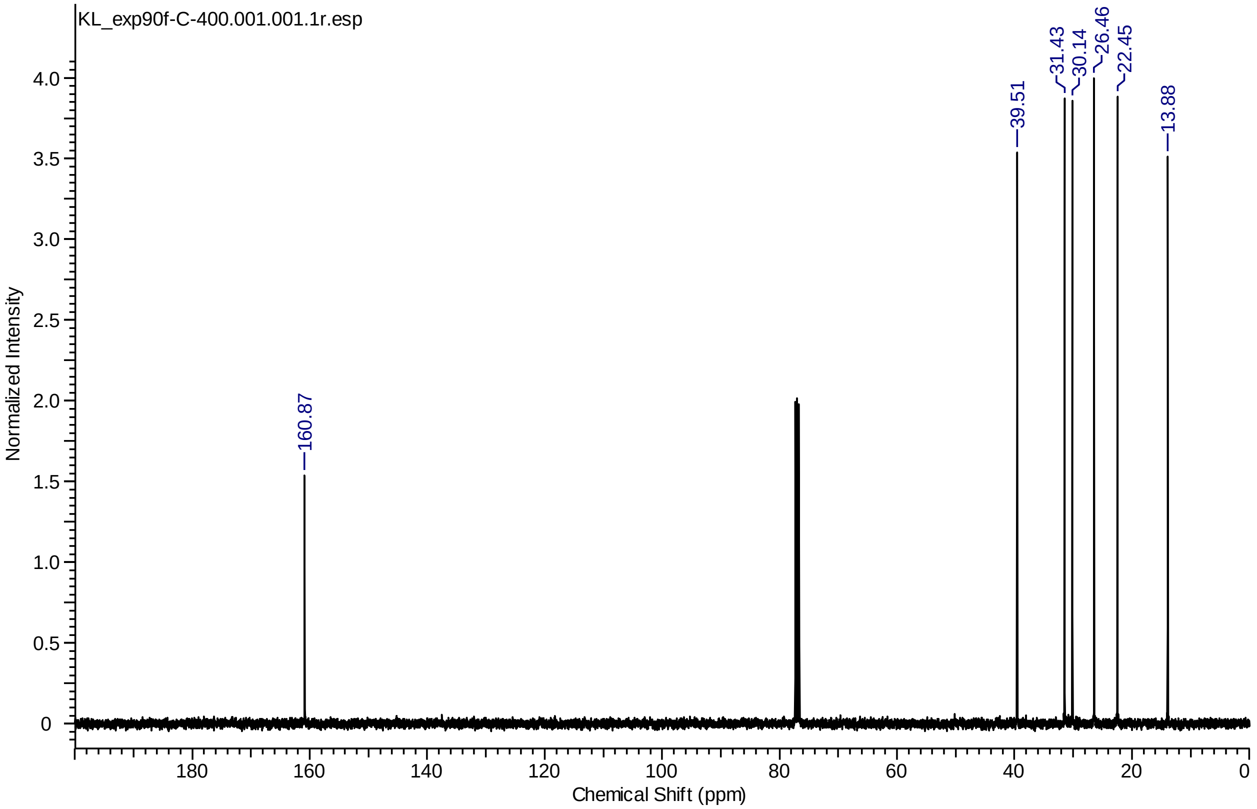
KL_exp90f-C-400.001.001.1r.esp
160.87
39.51
31.43
30.14
26.46
22.45
13.88
Normalized Intensity
Chemical Shift (ppm)

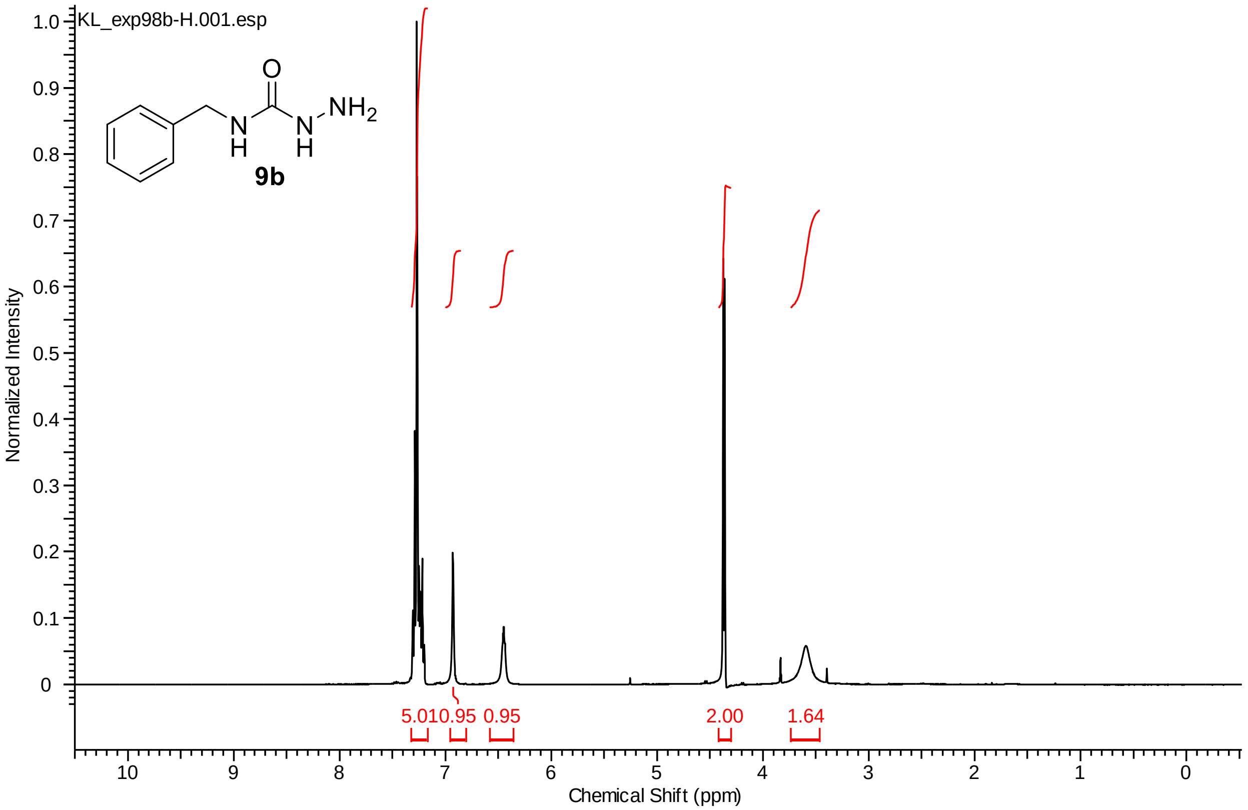
KL_exp98b-H.001.esp
9b
5.01
0.95
0.95
2.00
1.64
Chemical Shift (ppm)
Normalized Intensity

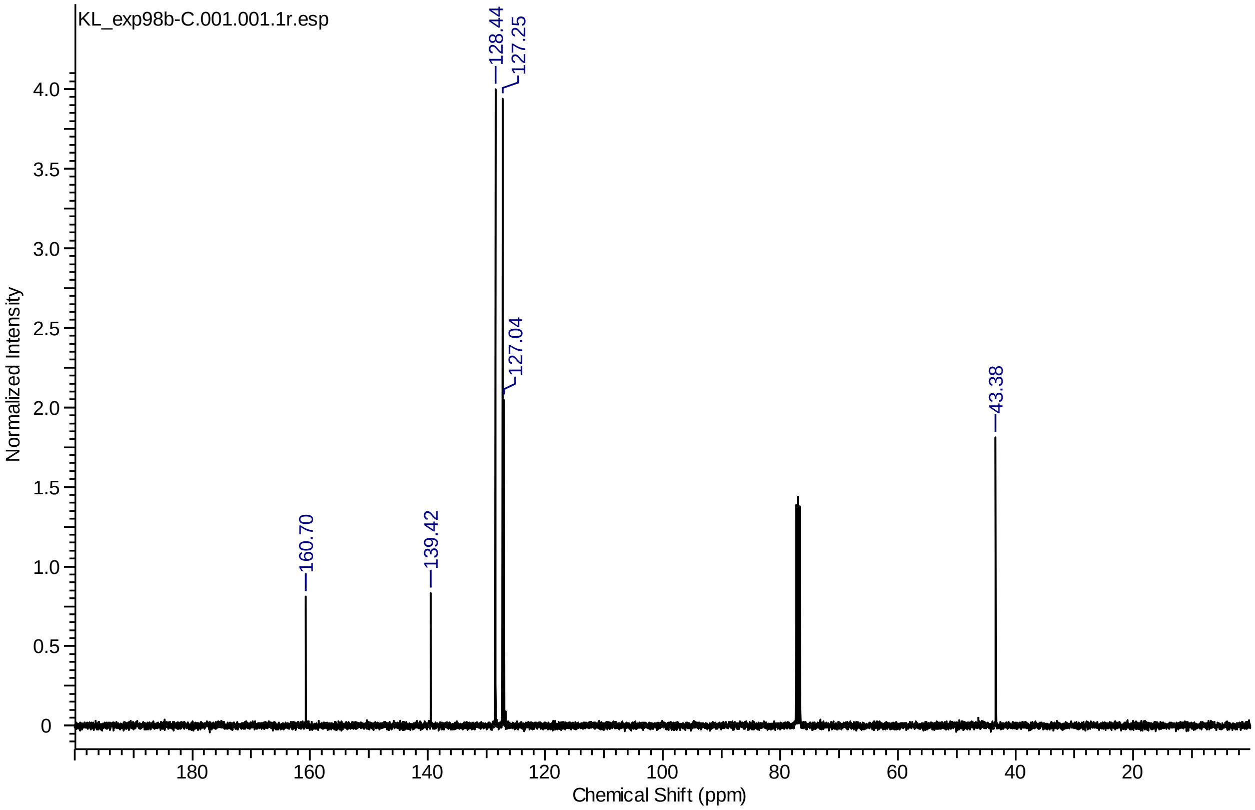
KL_exp98b-C.001.001.1r.esp
160.70
139.42
128.44
127.25
127.04
43.38
Chemical Shift (ppm)
Normalized Intensity

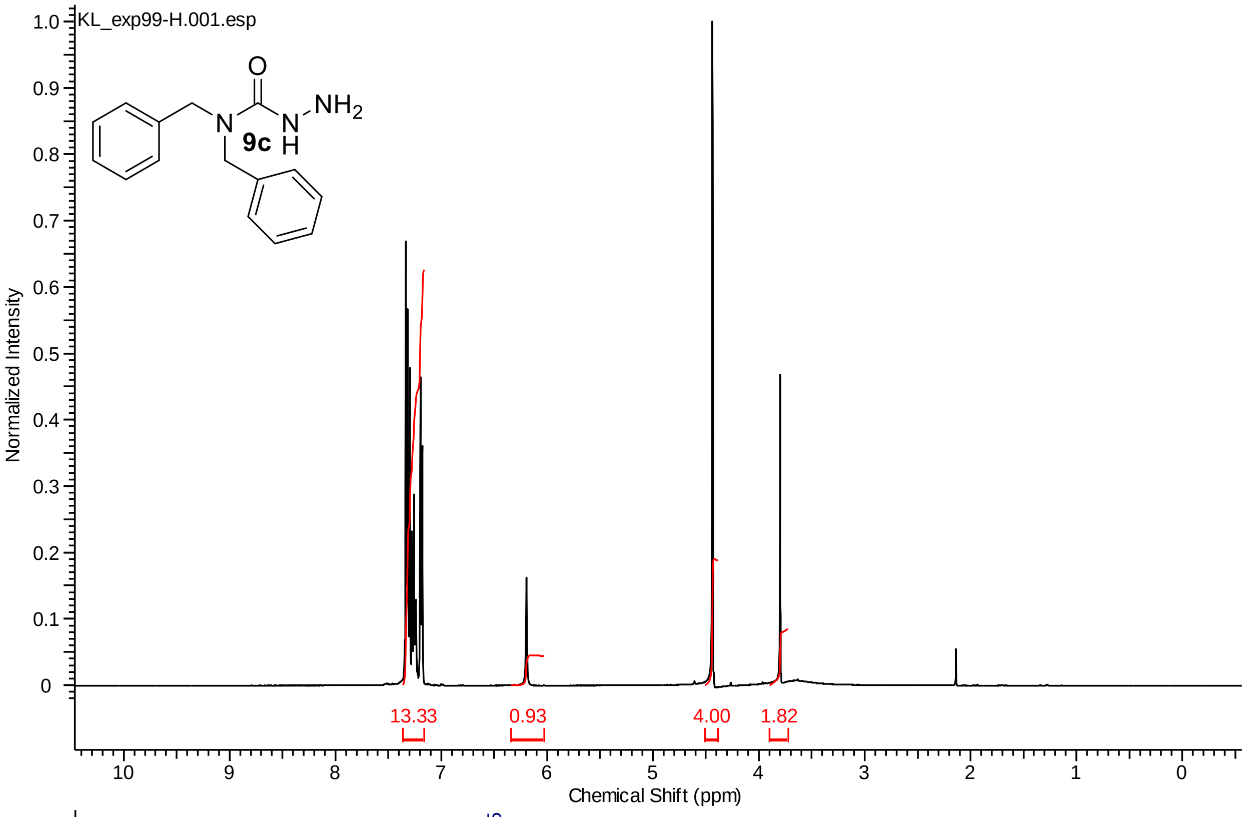
KL_exp99-H.001.esp
9c
13.33
0.93
4.00
1.82
Chemical Shift (ppm)
Normalized Intensity

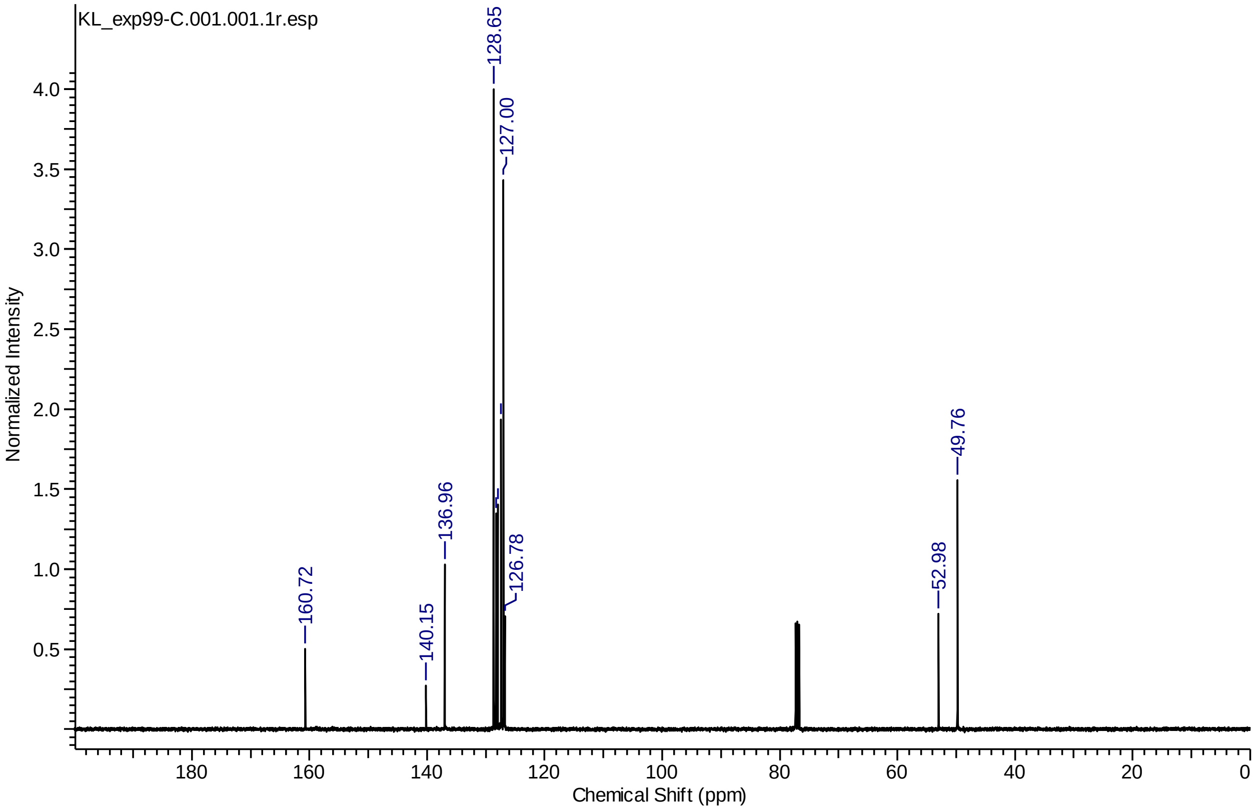
KL_exp99-C.001.001.1r.esp
160.72
140.15
136.96
128.65
127.00
126.78
52.98
49.76
Chemical Shift (ppm)
Normalized Intensity

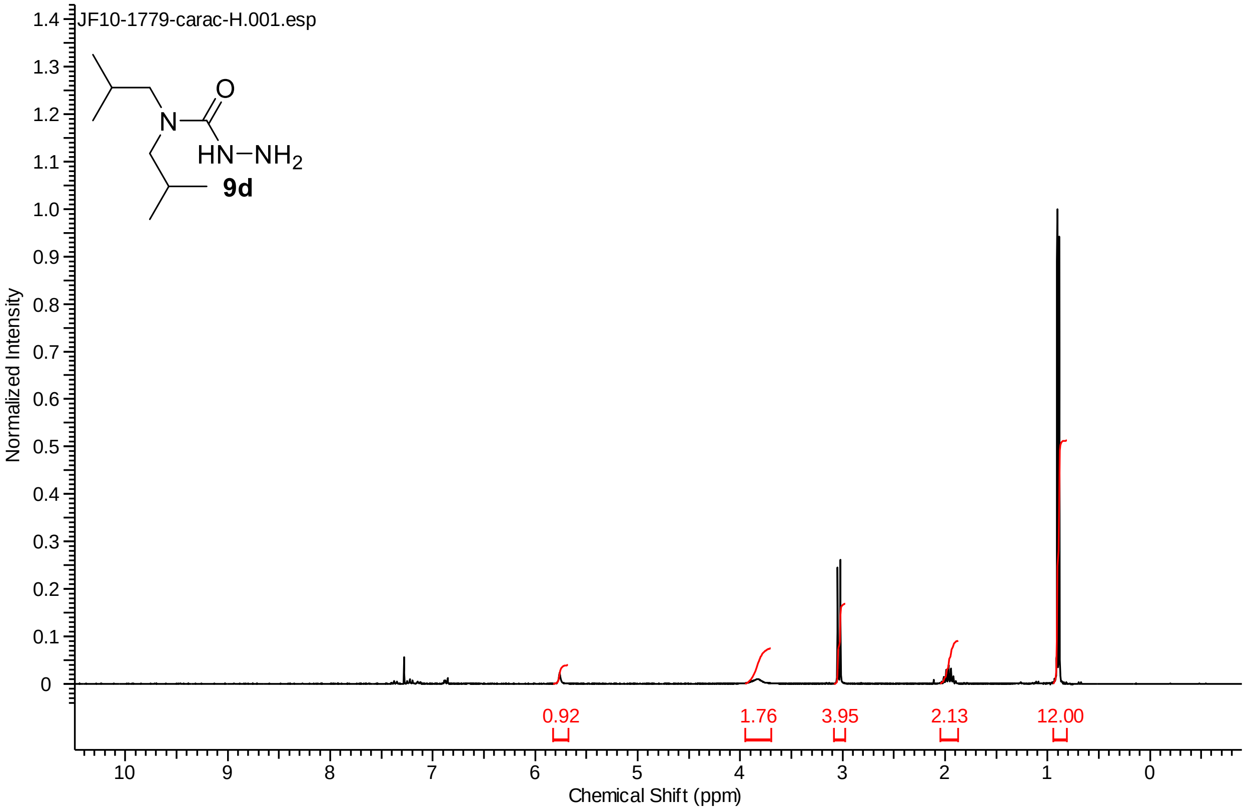
JF10-1779-carac-H.001.esp
O
N
HN−NH2
9d
Normalized Intensity
0.92
1.76
3.95
2.13
12.00
Chemical Shift (ppm)

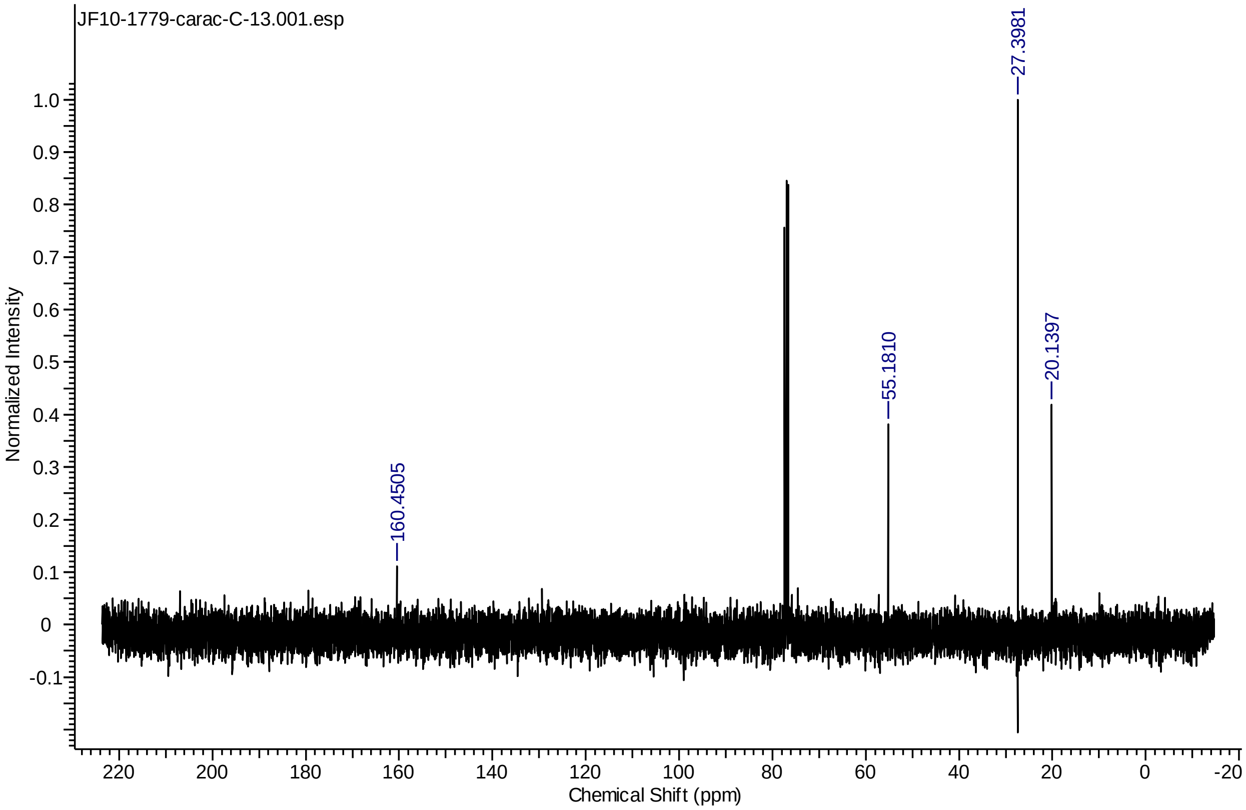
JF10-1779-carac-C-13.001.esp
160.4505
55.1810
27.3981
20.1397
Normalized Intensity
Chemical Shift (ppm)

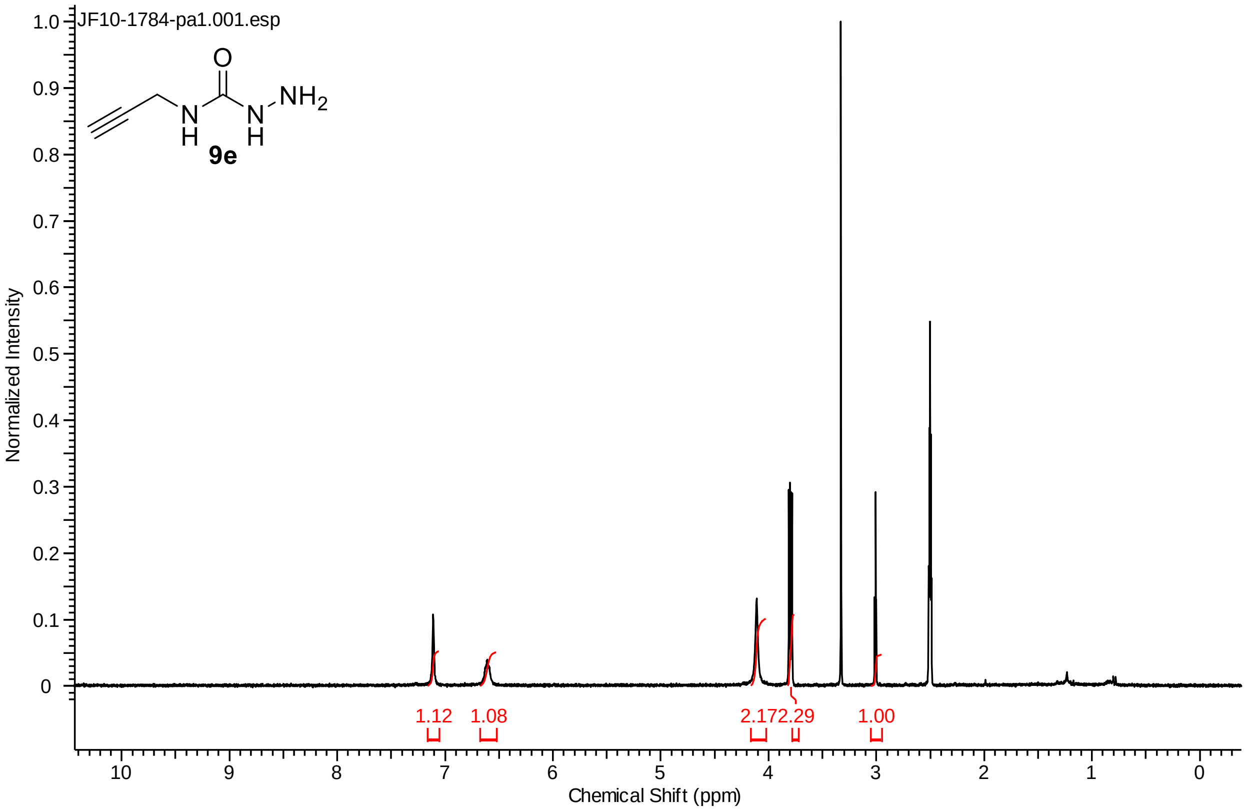
JF10-1784-pa1.001.esp
O
NH2
N
H
N
H
9e
Normalized Intensity
1.12
1.08
2.17
2.29
1.00
Chemical Shift (ppm)

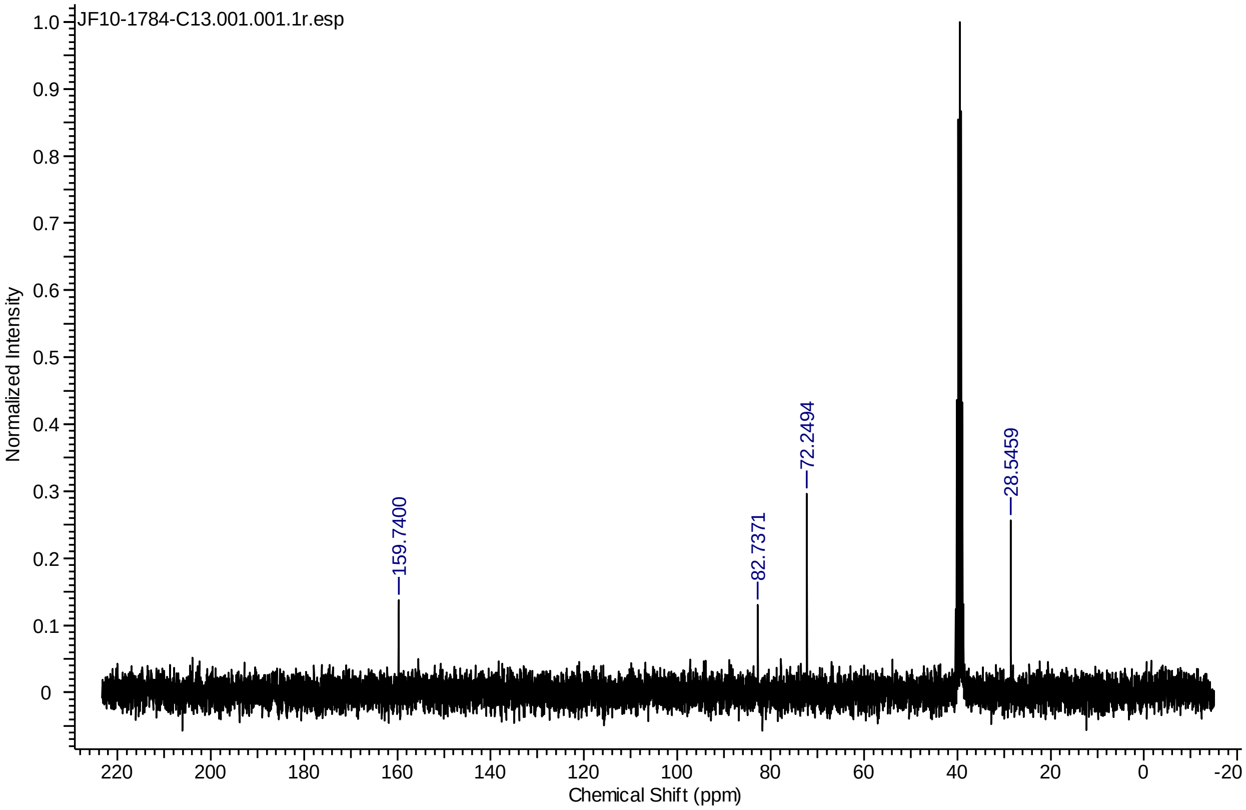
JF10-1784-C13.001.001.1r.esp
Normalized Intensity
159.7400
82.7371
72.2494
28.5459
Chemical Shift (ppm)

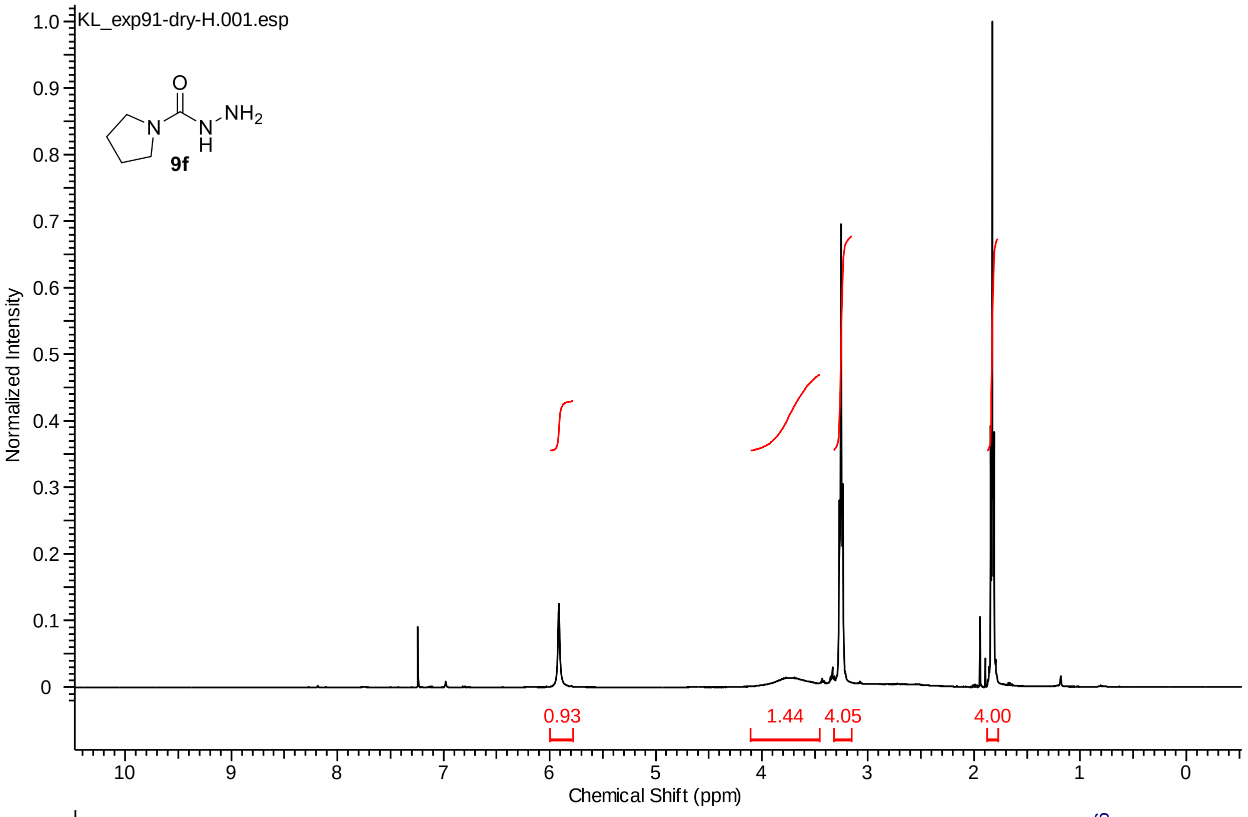
KL_exp91-dry-H.001.esp
O
N
N
H
NH2
9f
Normalized Intensity
0.93
1.44
4.05
4.00
Chemical Shift (ppm)

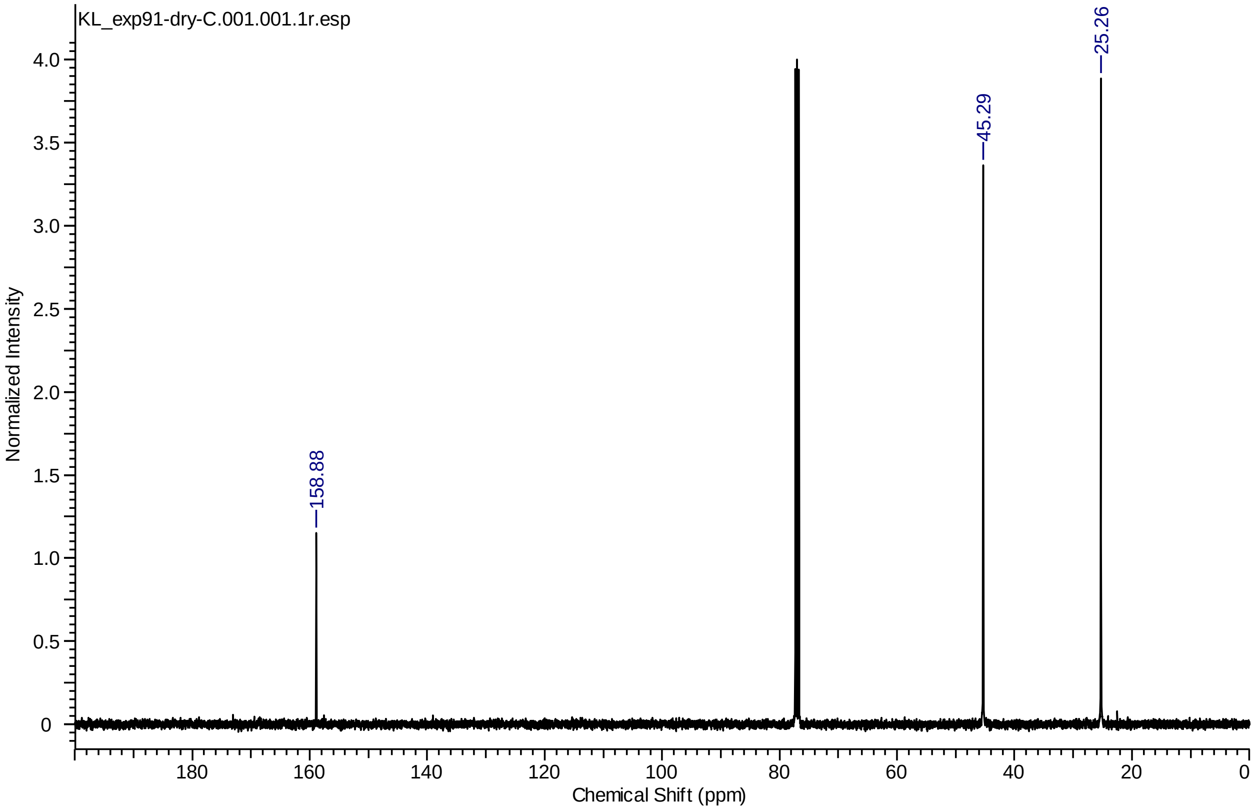
KL_exp91-dry-C.001.001.1r.esp
158.88
45.29
25.26
Normalized Intensity
Chemical Shift (ppm)

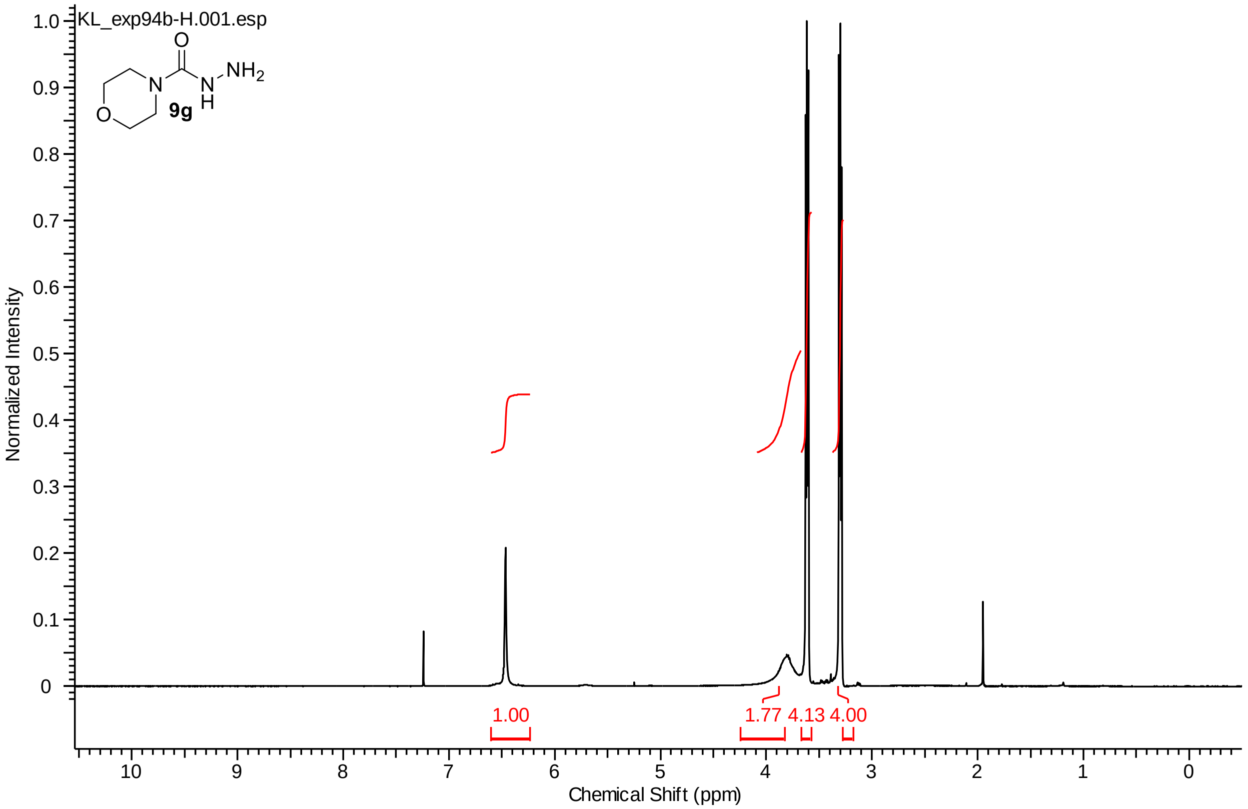
KL_exp94b-H.001.esp
O
N
NH2
N
H
O
9g
Normalized Intensity
1.00
1.77
4.13
4.00
Chemical Shift (ppm)

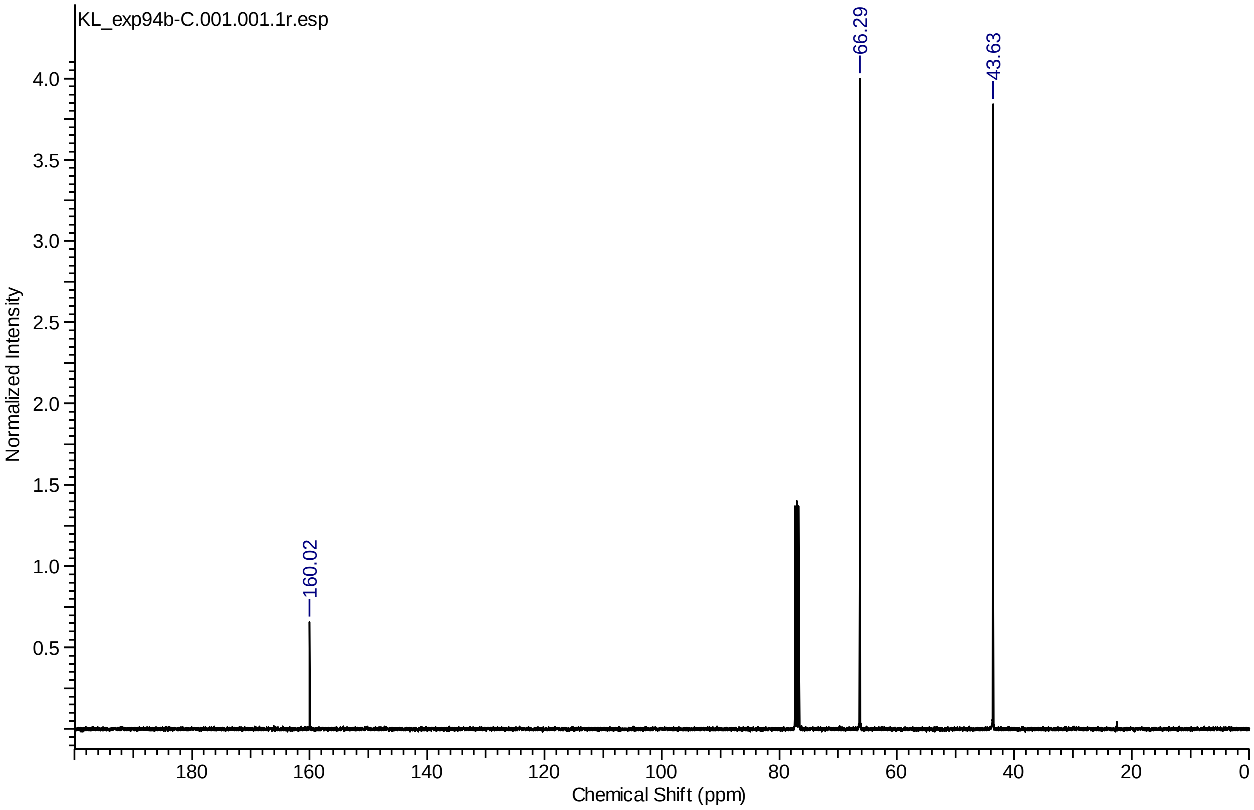
KL_exp94b-C.001.001.1r.esp
Normalized Intensity
160.02
66.29
43.63
Chemical Shift (ppm)

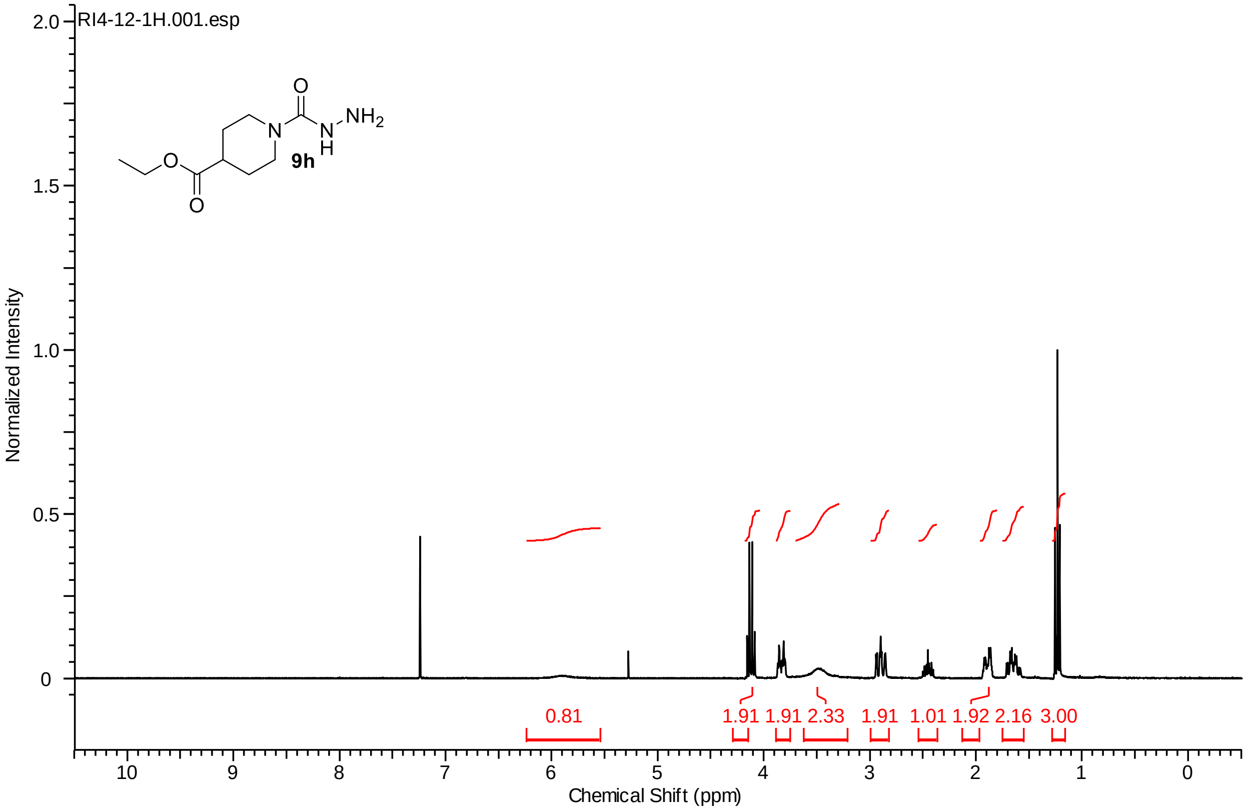
RI4-12-1H.001.esp
9h
Normalized Intensity
Chemical Shift (ppm)
0.81
1.91
1.91
2.33
1.91
1.01
1.92
2.16
3.00

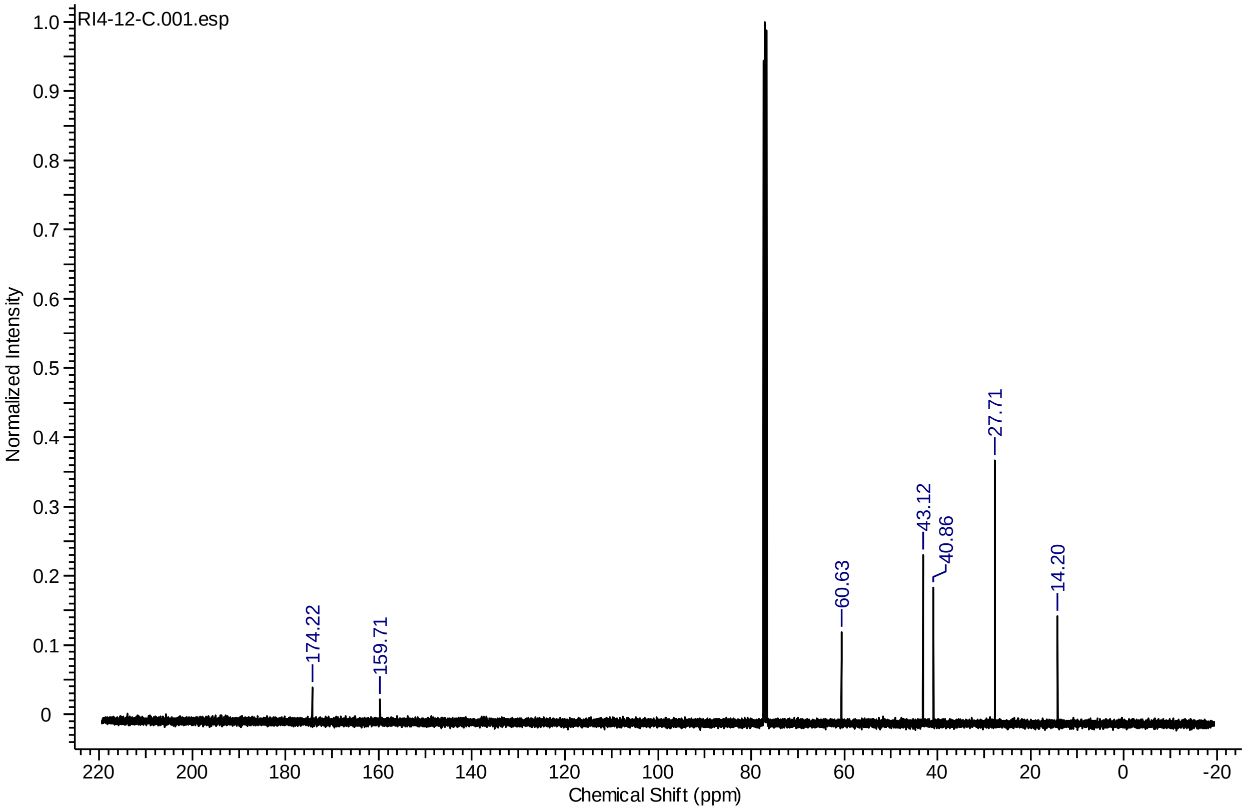
RI4-12-C.001.esp
Normalized Intensity
Chemical Shift (ppm)
174.22
159.71
60.63
43.12
40.86
27.71
14.20

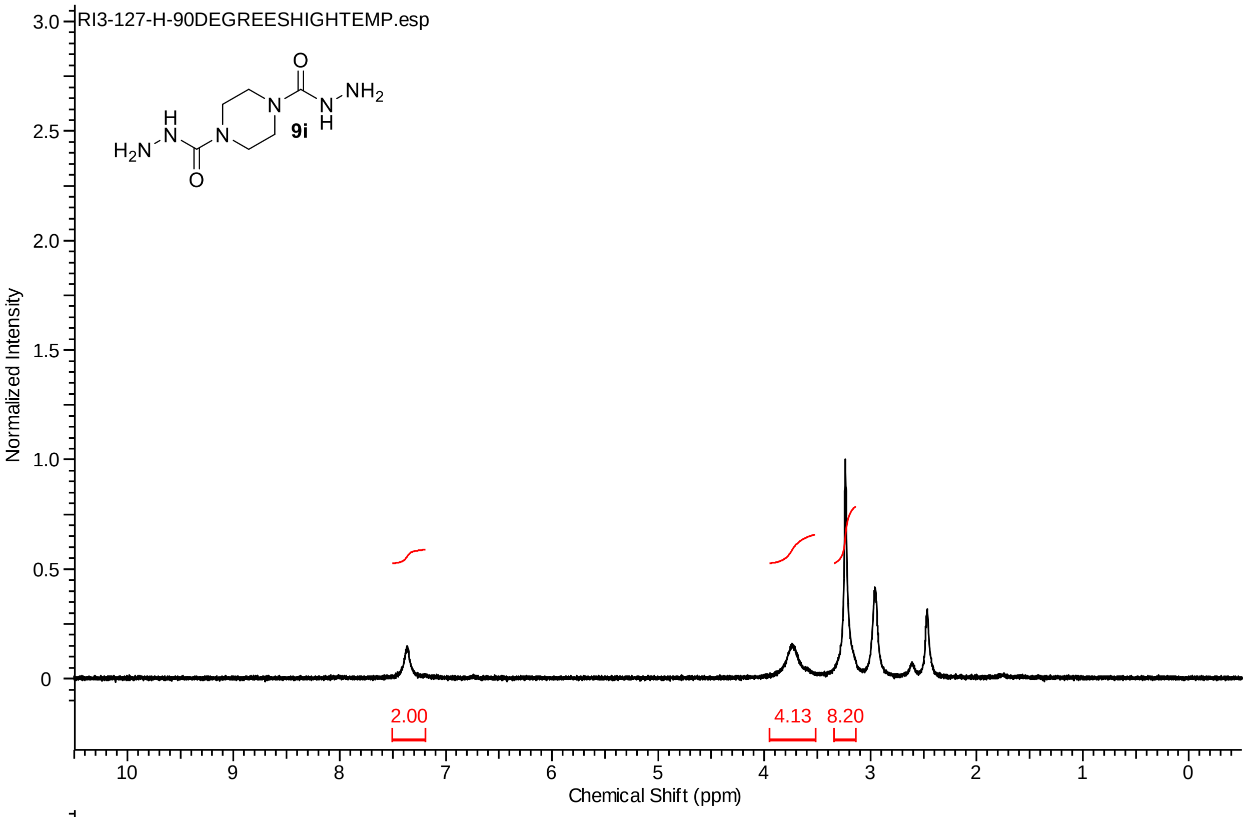
RI3-127-H-90DEGREESHIGHTEMP.esp
9i
Normalized Intensity
2.00
4.13
8.20
Chemical Shift (ppm)

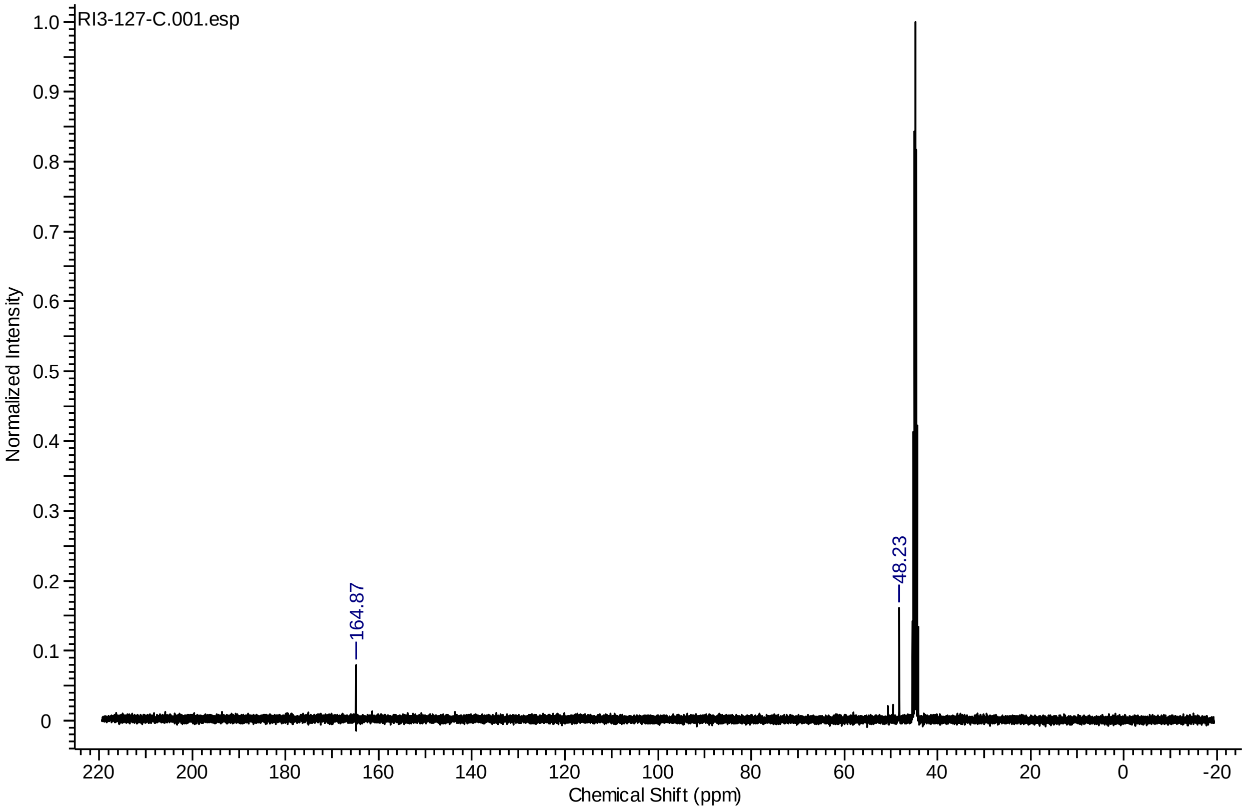
RI3-127-C.001.esp
Normalized Intensity
164.87
48.23
Chemical Shift (ppm)

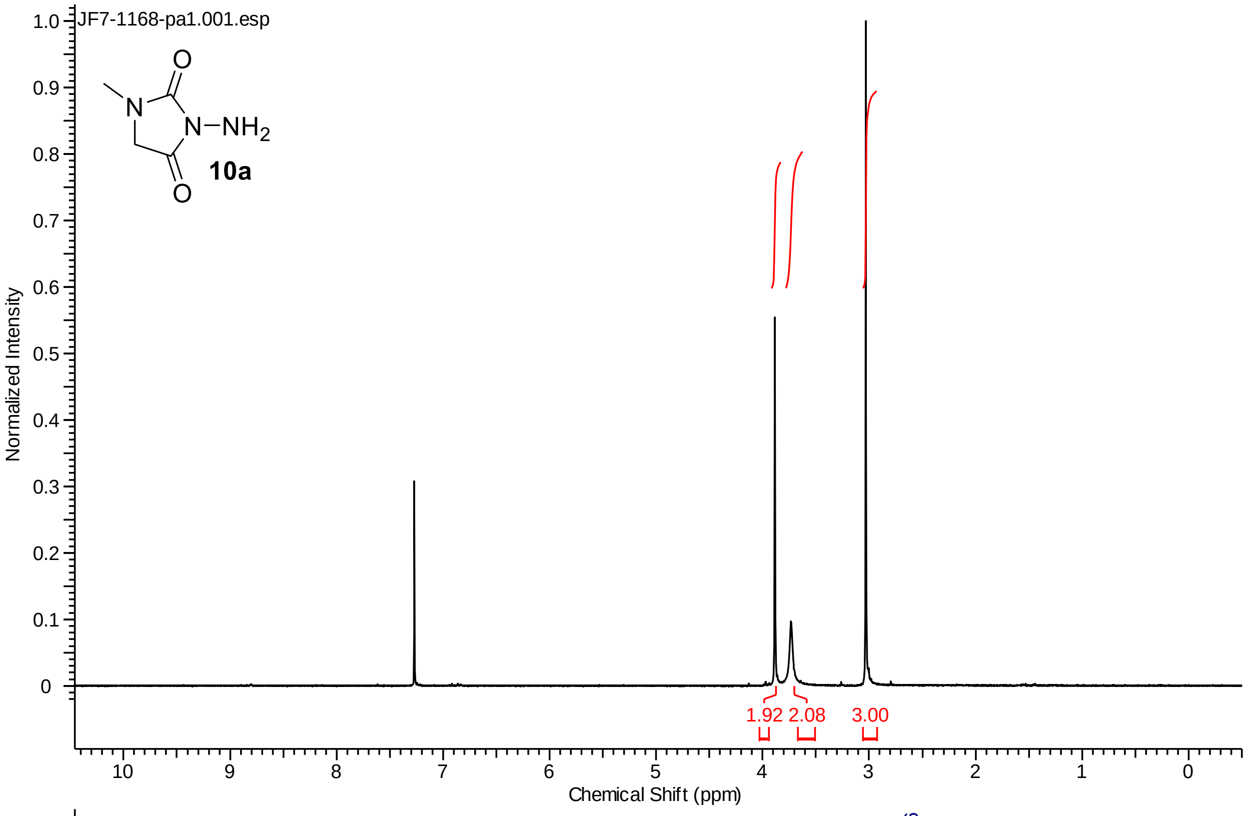
JF7-1168-pa1.001.esp
10a
1.92
2.08
3.00
Chemical Shift (ppm)
Normalized Intensity

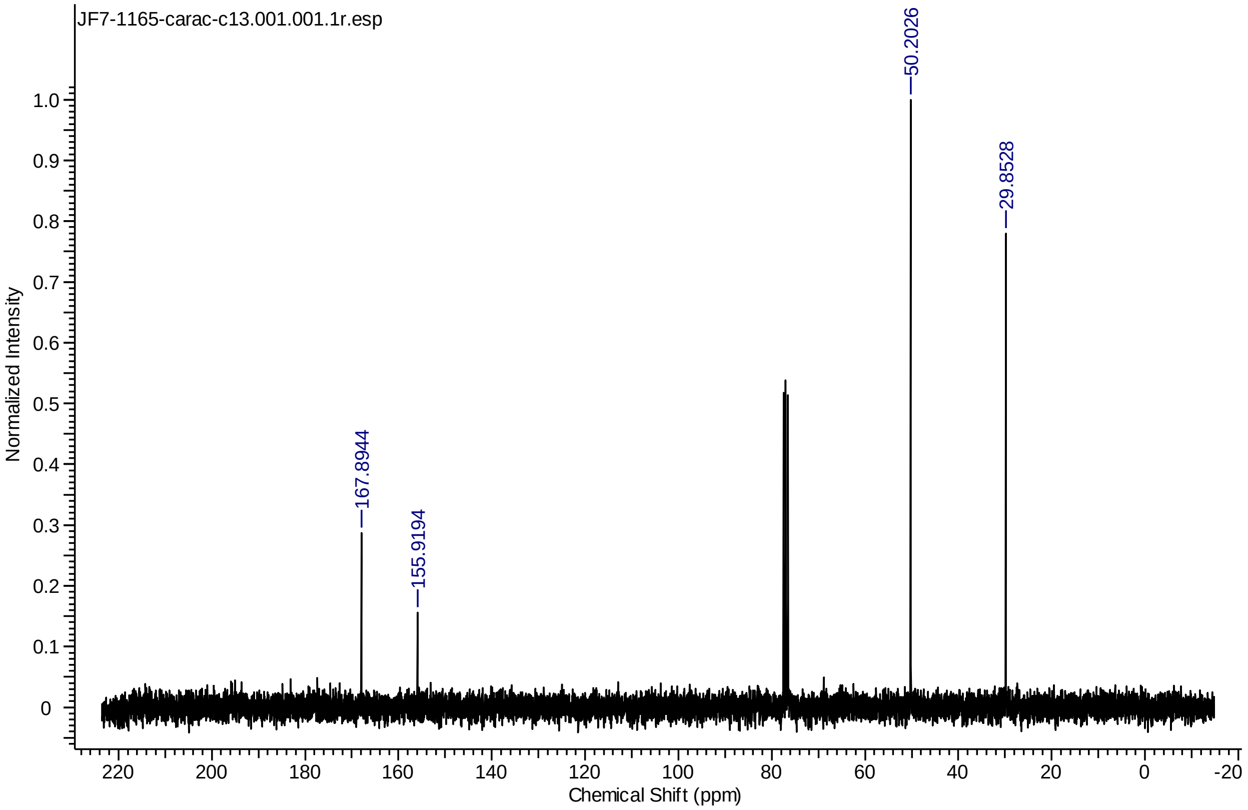
JF7-1165-carac-c13.001.001.1r.esp
167.8944
155.9194
50.2026
29.8528
Chemical Shift (ppm)
Normalized Intensity

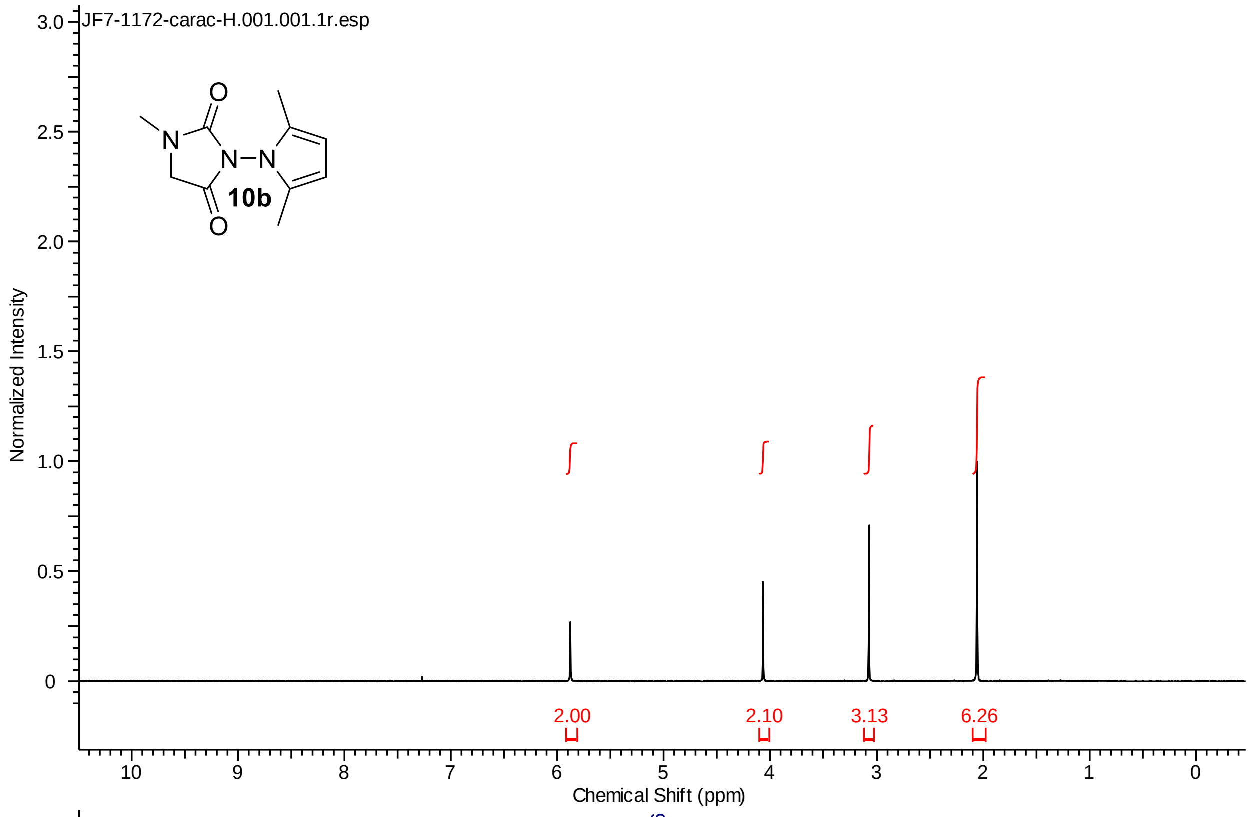
JF7-1172-carac-H.001.001.1r.esp
10b
2.00
2.10
3.13
6.26
Normalized Intensity
Chemical Shift (ppm)

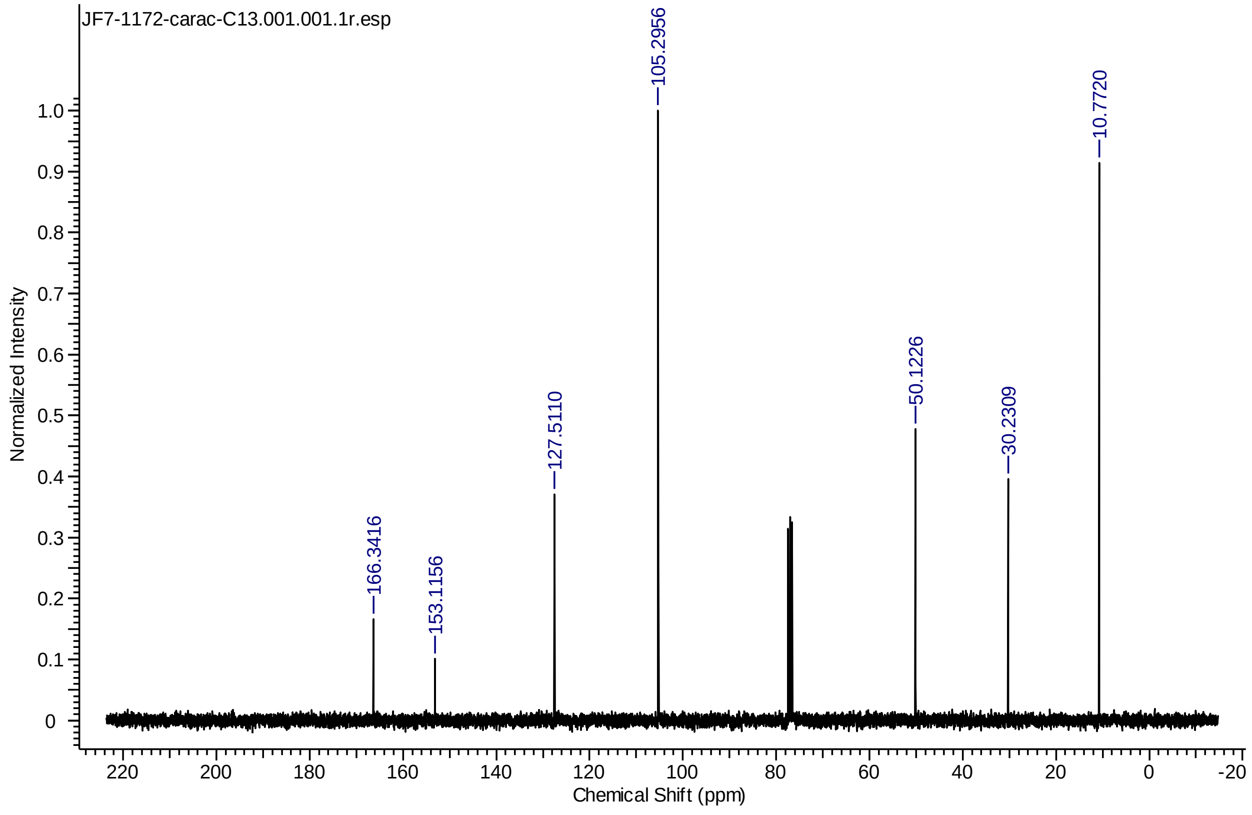
JF7-1172-carac-C13.001.001.1r.esp
166.3416
153.1156
127.5110
105.2956
50.1226
30.2309
10.7720
Normalized Intensity
Chemical Shift (ppm)

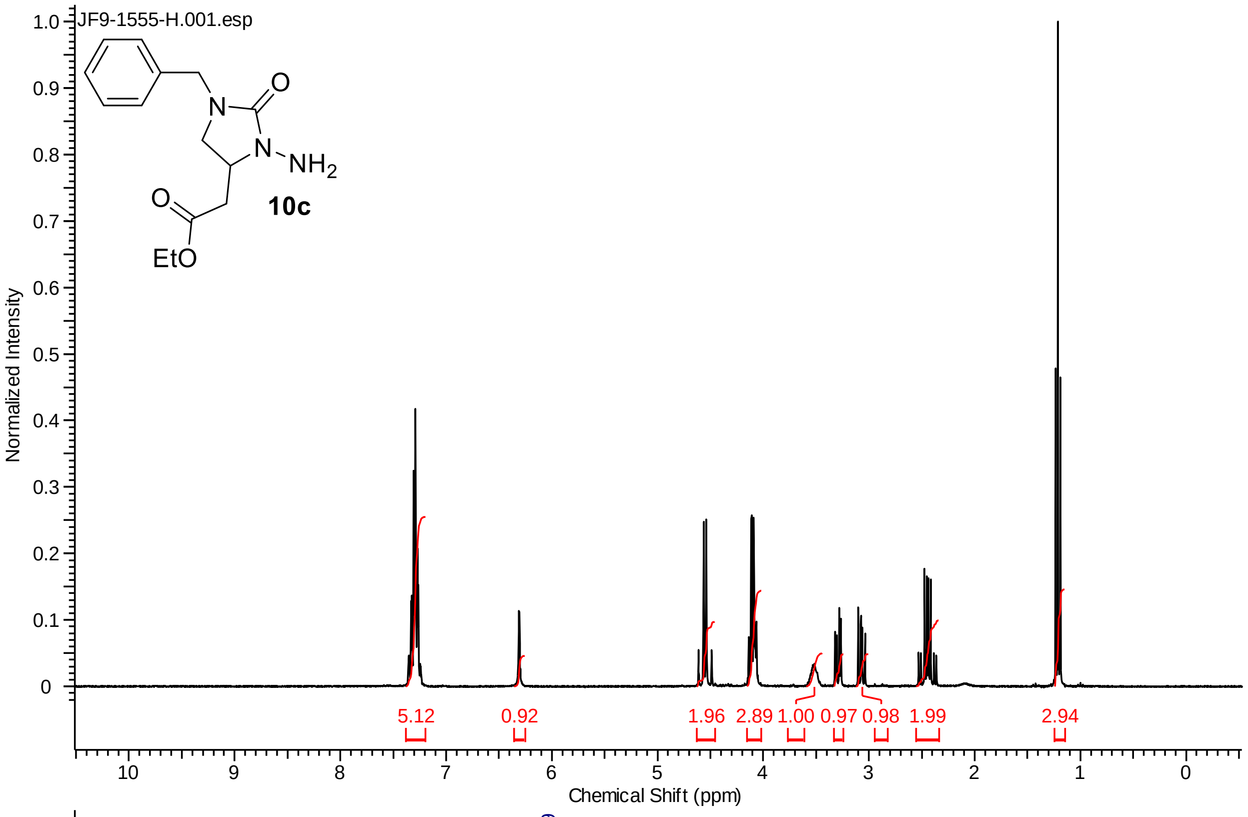
JF9-1555-H.001.esp
O
N
N
NH2
O
EtO
10c
Normalized Intensity
5.12
0.92
1.96
2.89
1.00
0.97
0.98
1.99
2.94
Chemical Shift (ppm)

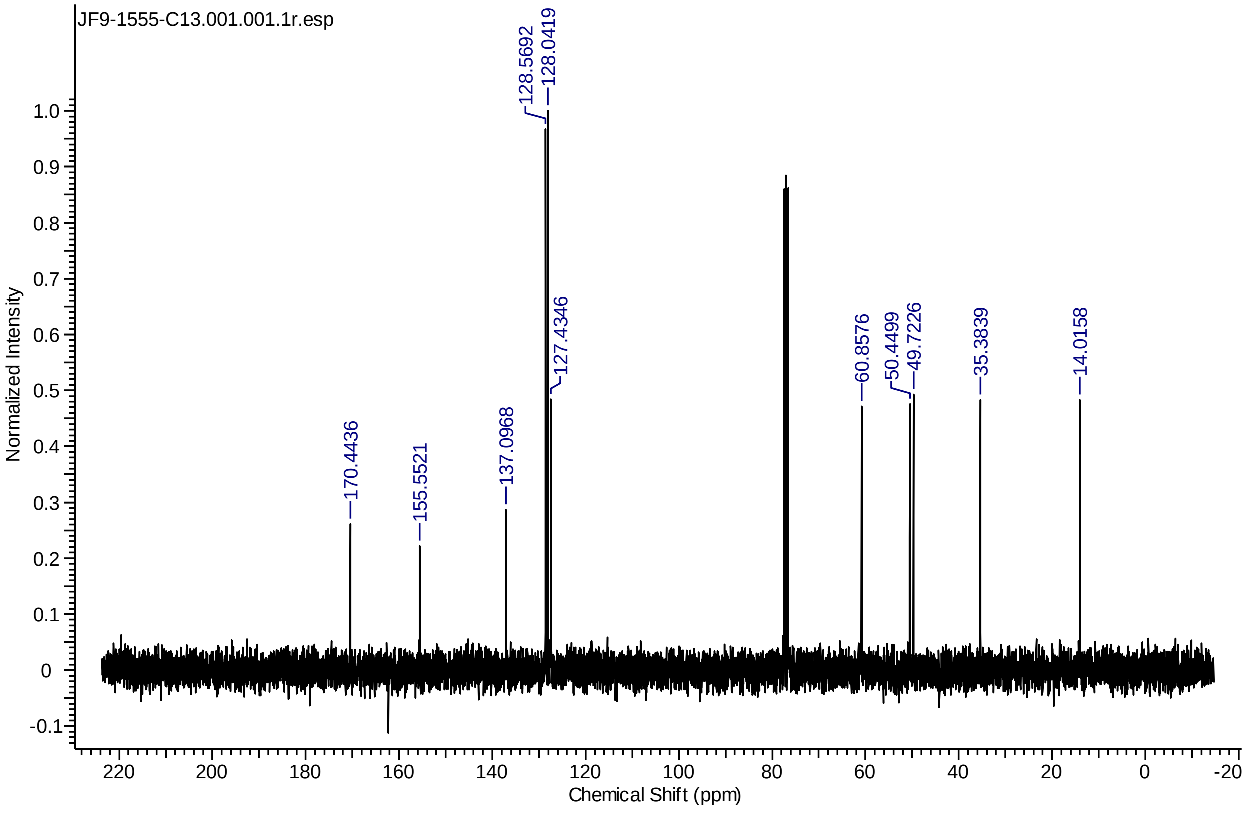
JF9-1555-C13.001.001.1r.esp
128.5692
128.0419
127.4346
170.4436
155.5521
137.0968
60.8576
50.4499
49.7226
35.3839
14.0158
Normalized Intensity
Chemical Shift (ppm)

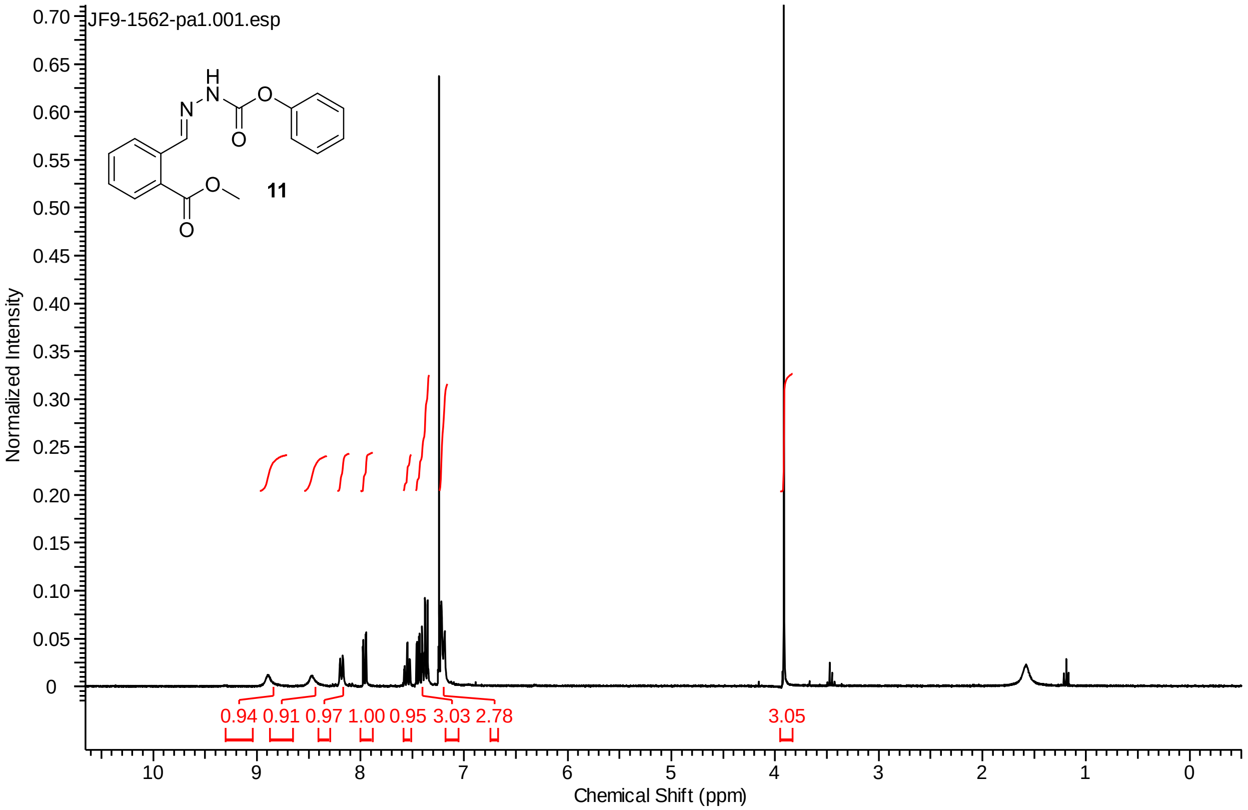
JF9-1562-pa1.001.esp
11
Normalized Intensity
0.94 0.91 0.97 1.00 0.95 3.03 2.78
3.05
Chemical Shift (ppm)

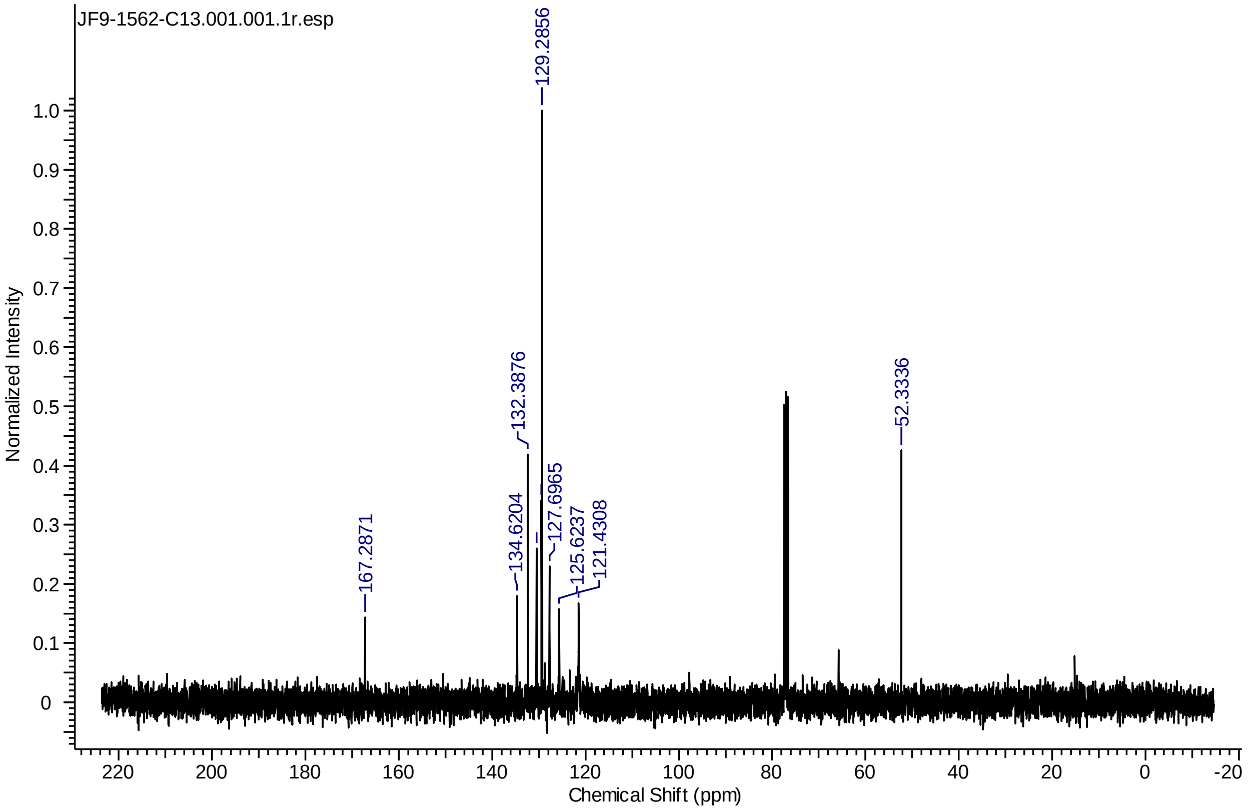
JF9-1562-C13.001.001.1r.esp
129.2856
132.3876
52.3336
167.2871
134.6204
127.6965
125.6237
121.4308
Normalized Intensity
Chemical Shift (ppm)

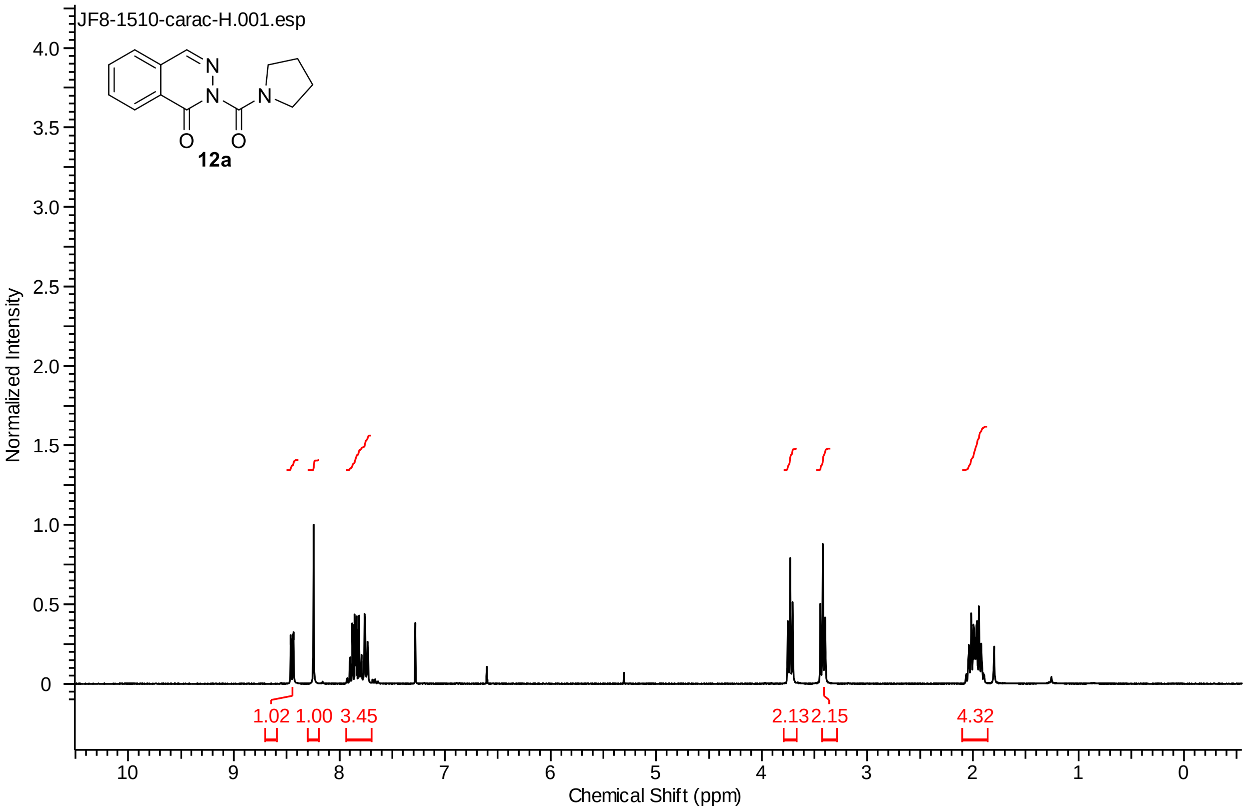
JF8-1510-carac-H.001.esp
12a
Normalized Intensity
1.02 1.00 3.45
2.13 2.15
4.32
Chemical Shift (ppm)

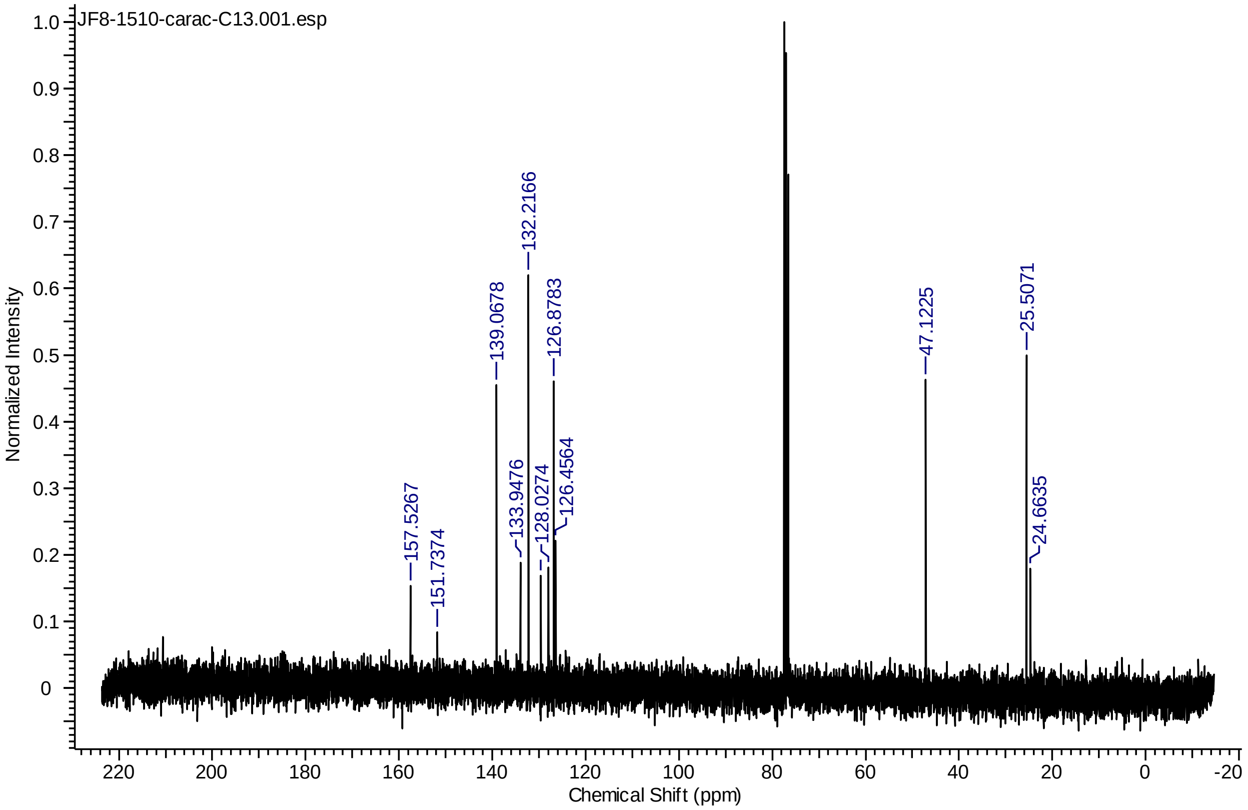
JF8-1510-carac-C13.001.esp
Normalized Intensity
157.5267
151.7374
139.0678
133.9476
132.2166
128.0274
126.8783
126.4564
47.1225
25.5071
24.6635
Chemical Shift (ppm)

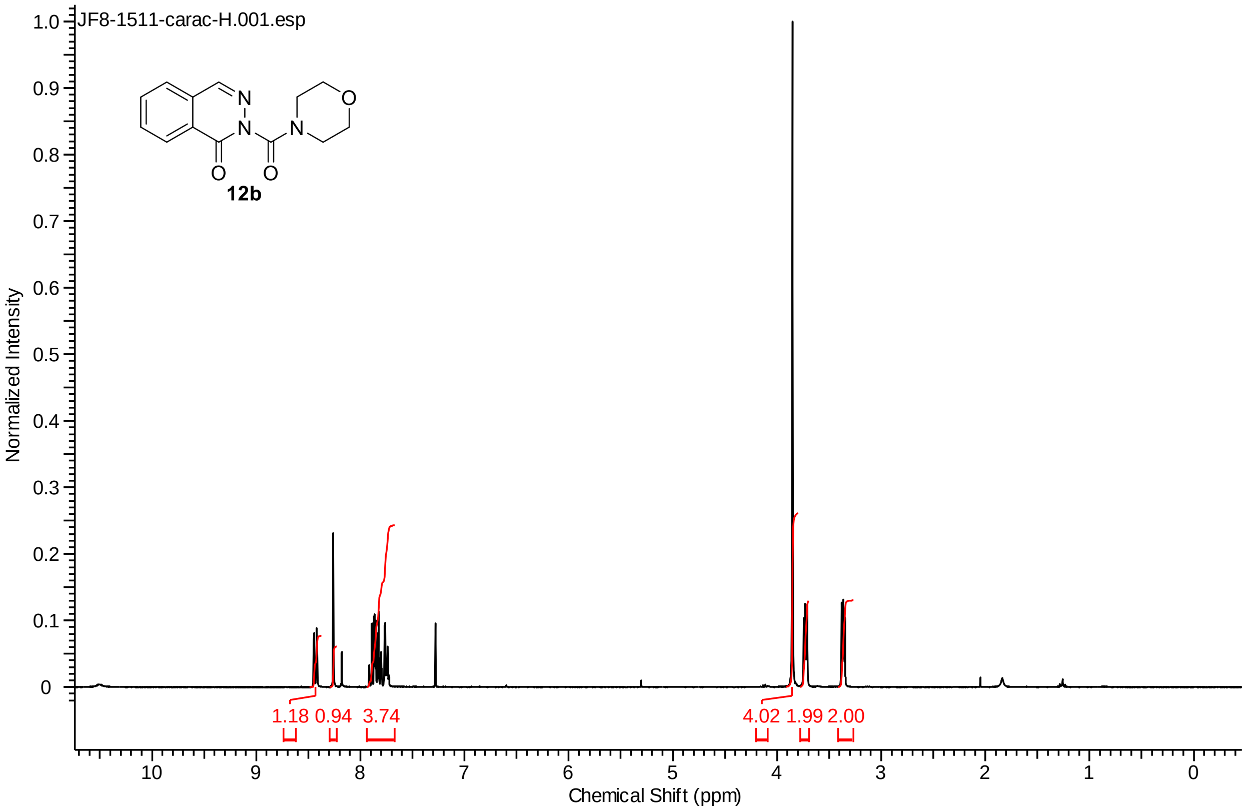
JF8-1511-carac-H.001.esp
12b
Normalized Intensity
1.18
0.94
3.74
4.02
1.99
2.00
Chemical Shift (ppm)

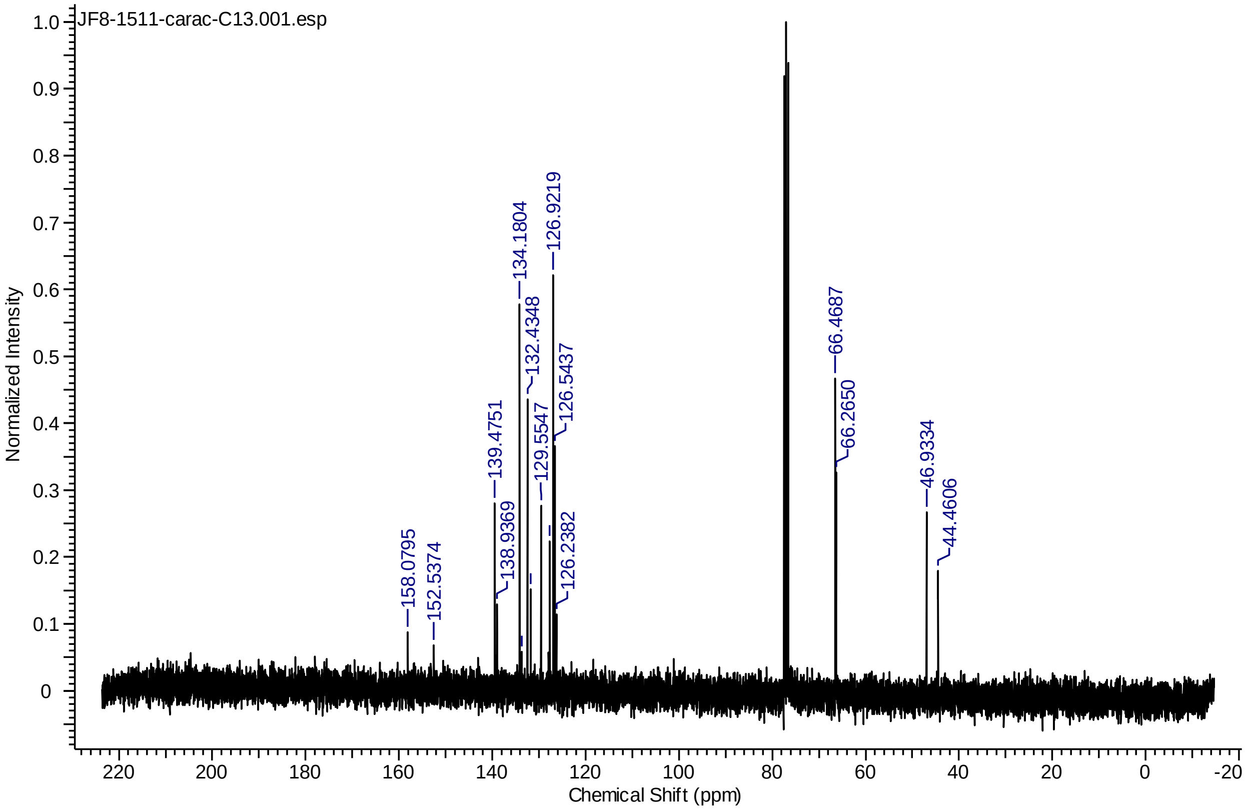
JF8-1511-carac-C13.001.esp
Normalized Intensity
158.0795
152.5374
139.4751
138.9369
134.1804
132.4348
129.5547
126.9219
126.5437
126.2382
66.4687
66.2650
46.9334
44.4606
Chemical Shift (ppm)

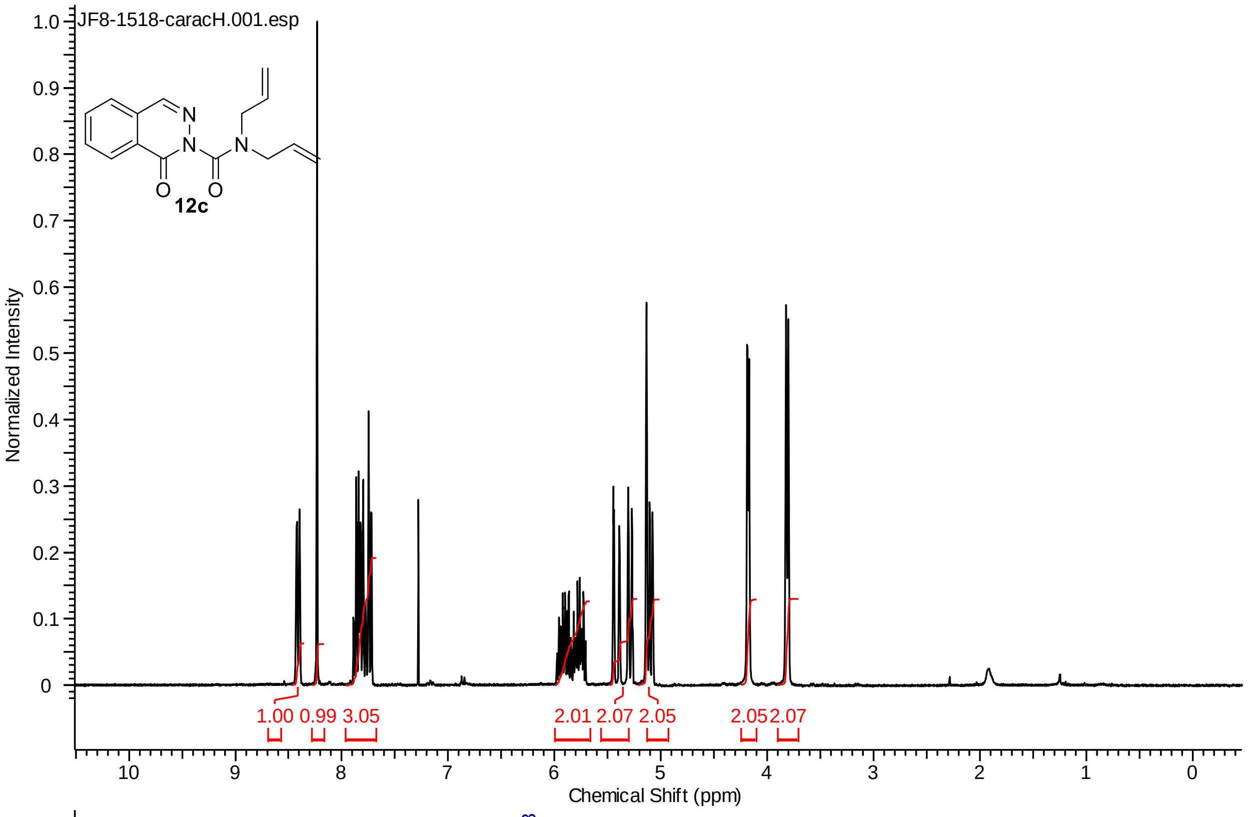
JF8-1518-caracH.001.esp
12c
Normalized Intensity
Chemical Shift (ppm)
1.00 0.99 3.05 2.01 2.07 2.05 2.05 2.07

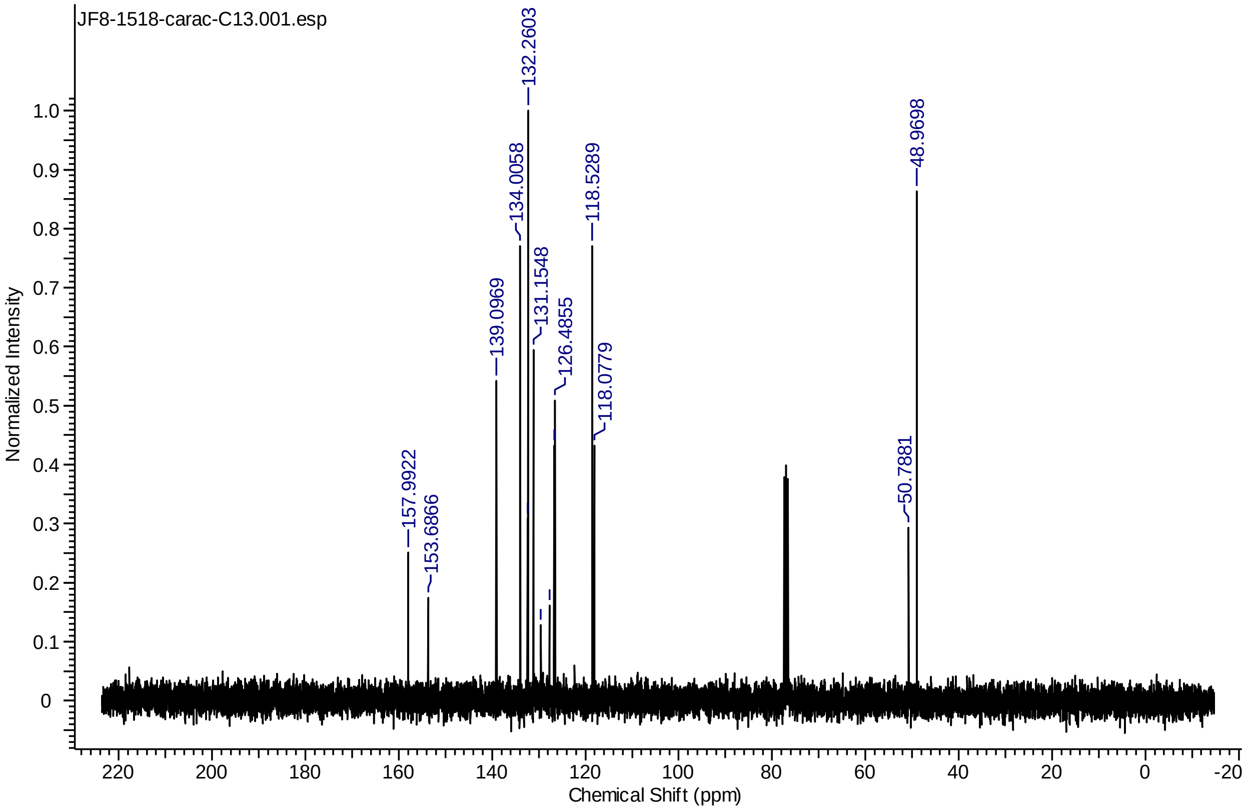
JF8-1518-carac-C13.001.esp
Normalized Intensity
Chemical Shift (ppm)
157.9922
153.6866
139.0969
134.0058
132.2603
131.1548
126.4855
118.5289
118.0779
50.7881
48.9698

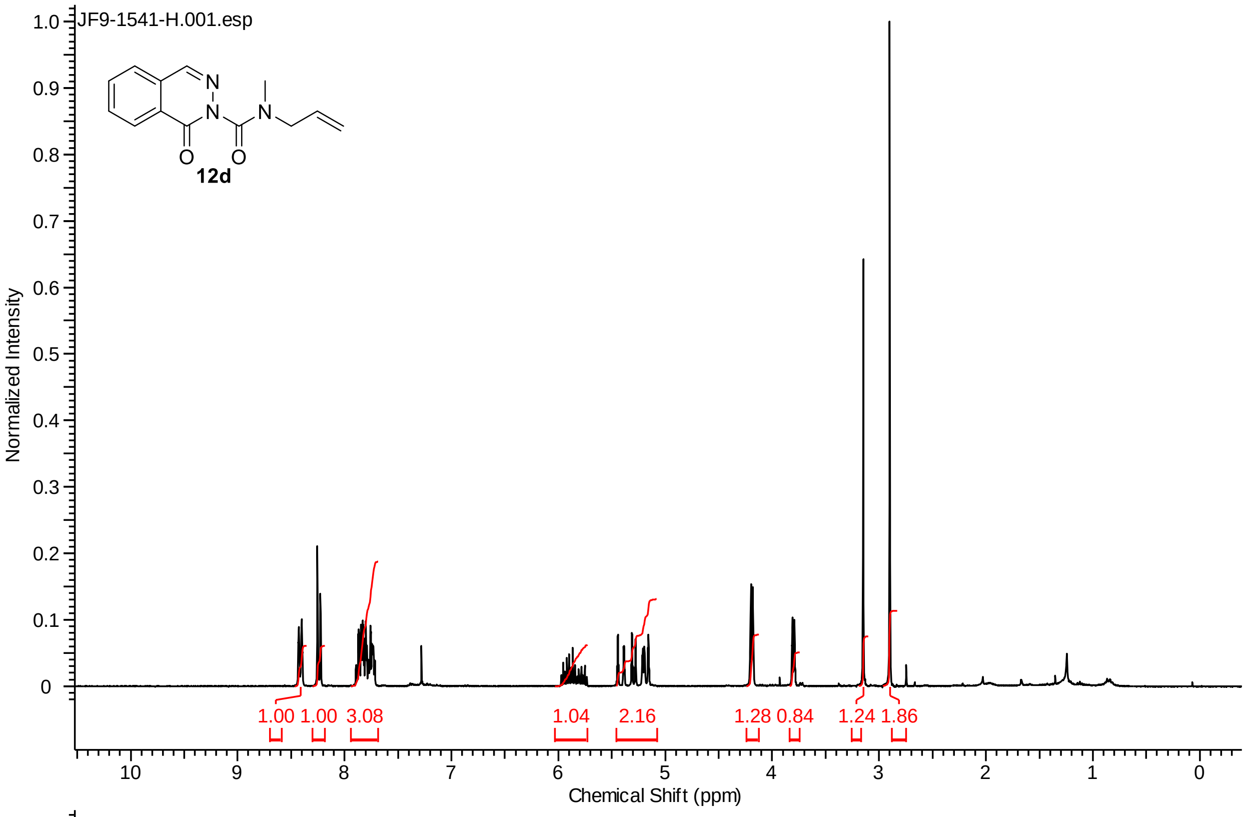
JF9-1541-H.001.esp
12d
Normalized Intensity
1.00 1.00 3.08
1.04
2.16
1.28 0.84
1.24 1.86
Chemical Shift (ppm)

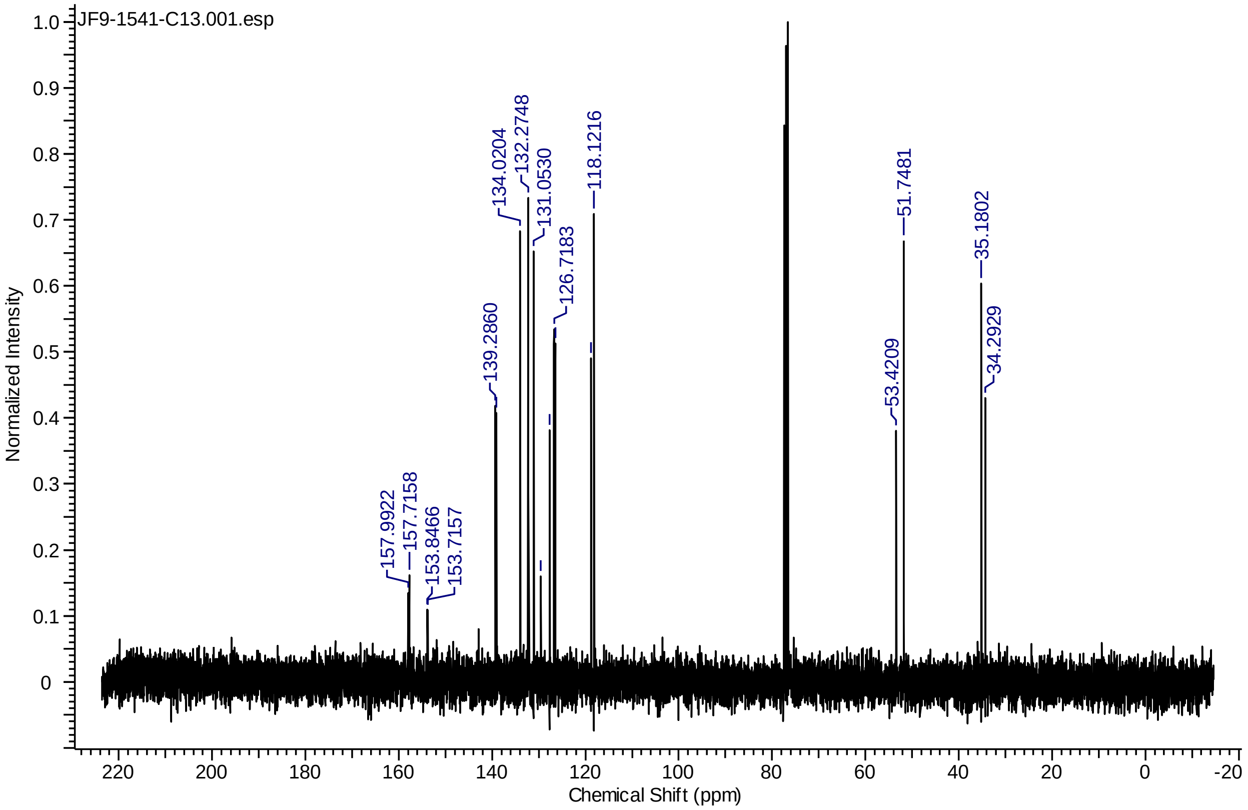
JF9-1541-C13.001.esp
Normalized Intensity
157.9922
157.7158
153.8466
153.7157
139.2860
134.0204
132.2748
131.0530
126.7183
118.1216
53.4209
51.7481
35.1802
34.2929
Chemical Shift (ppm)

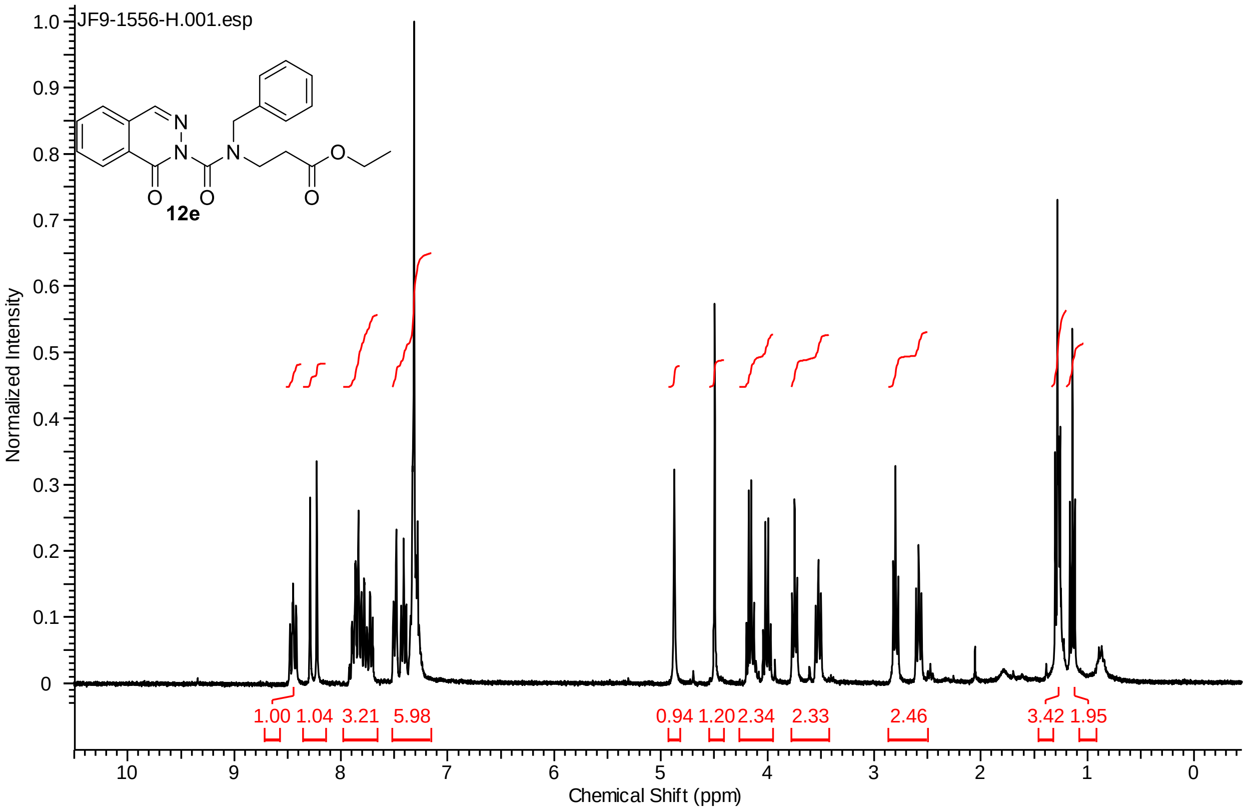
JF9-1556-H.001.esp
12e
1.00
1.04
3.21
5.98
0.94
1.20
2.34
2.33
2.46
3.42
1.95
Normalized Intensity
Chemical Shift (ppm)

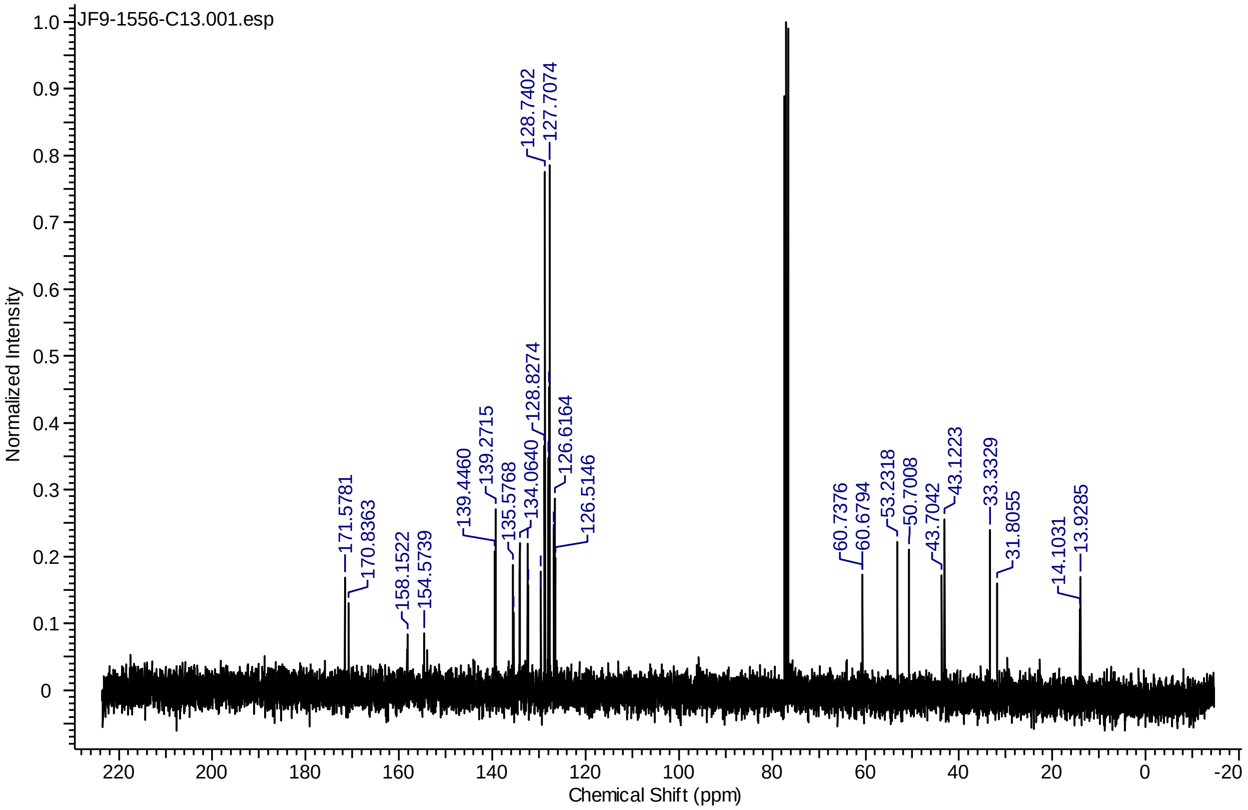
JF9-1556-C13.001.esp
171.5781
170.8363
158.1522
154.5739
139.4460
139.2715
135.5768
134.0640
128.8274
128.7402
127.7074
126.6164
126.5146
60.7376
60.6794
53.2318
50.7008
43.7042
43.1223
33.3329
31.8055
14.1031
13.9285
Normalized Intensity
Chemical Shift (ppm)

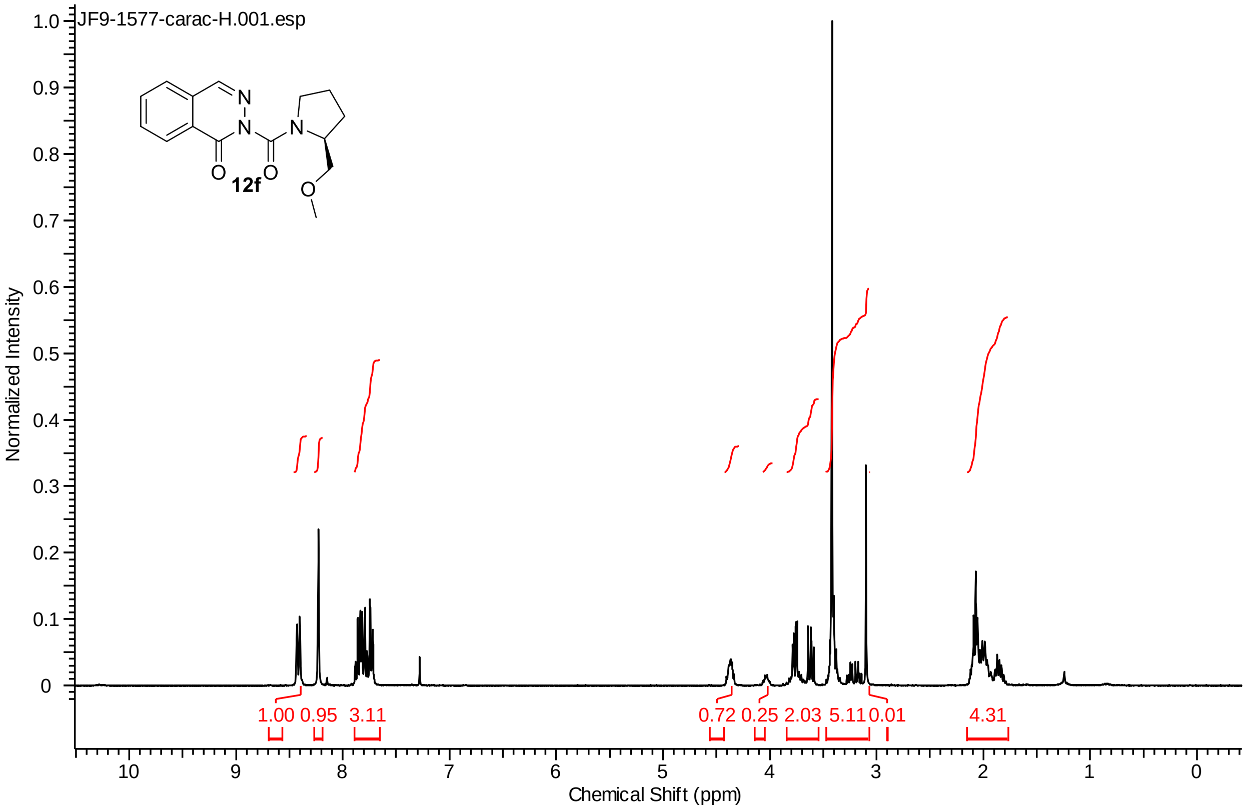
JF9-1577-carac-H.001.esp
12f
1.00 0.95 3.11
0.72 0.25 2.03 5.11 0.01
4.31
Normalized Intensity
Chemical Shift (ppm)

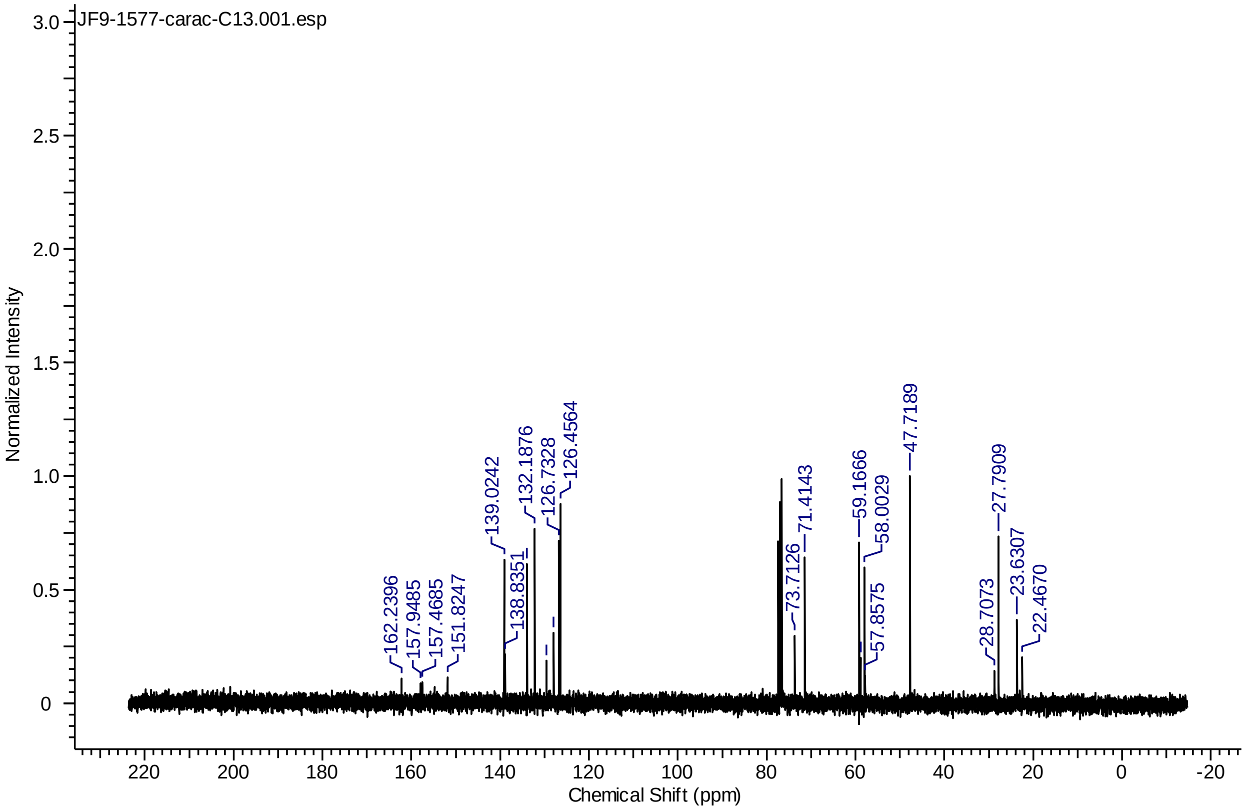
JF9-1577-carac-C13.001.esp
162.2396
157.9485
157.4685
151.8247
139.0242
138.8351
132.1876
126.7328
126.4564
73.7126
71.4143
59.1666
58.0029
57.8575
47.7189
28.7073
27.7909
23.6307
22.4670
Normalized Intensity
Chemical Shift (ppm)

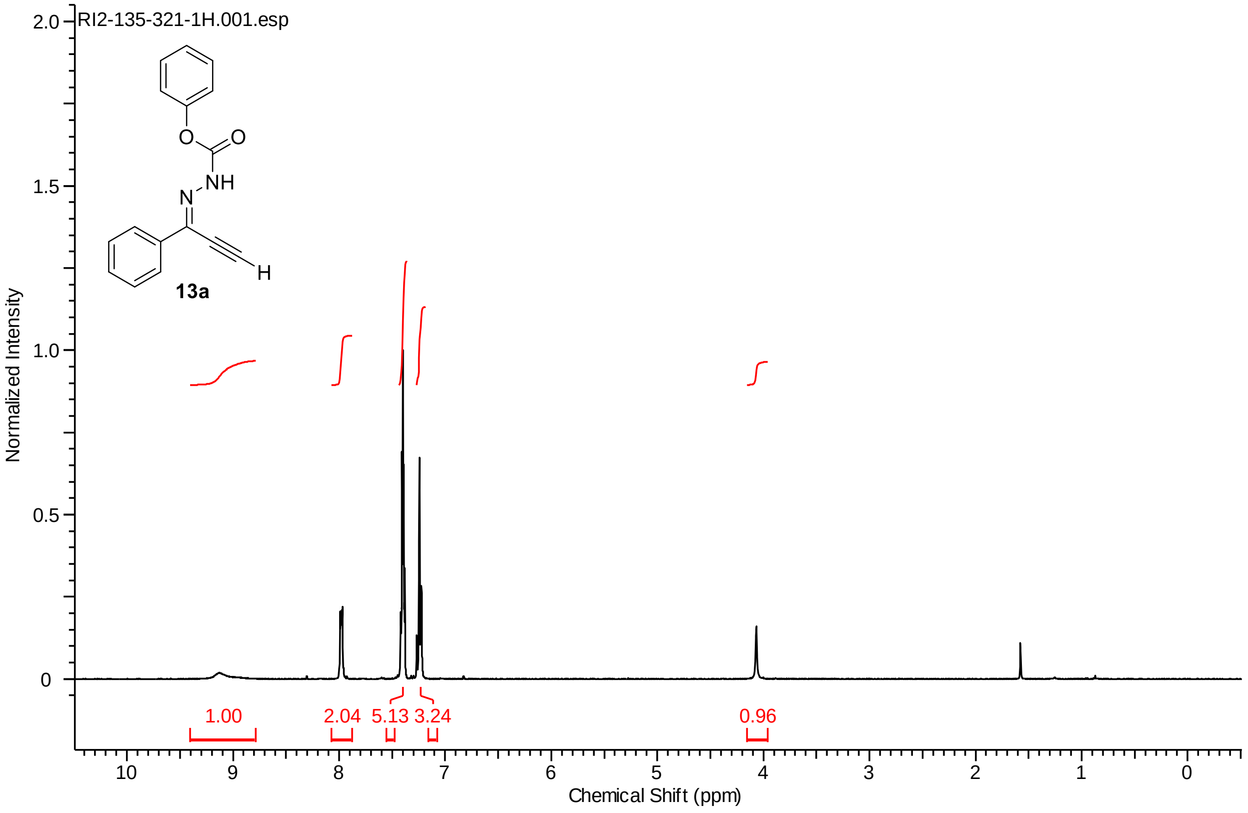
RI2-135-321-1H.001.esp
13a
Normalized Intensity
Chemical Shift (ppm)
1.00
2.04
5.13
3.24
0.96

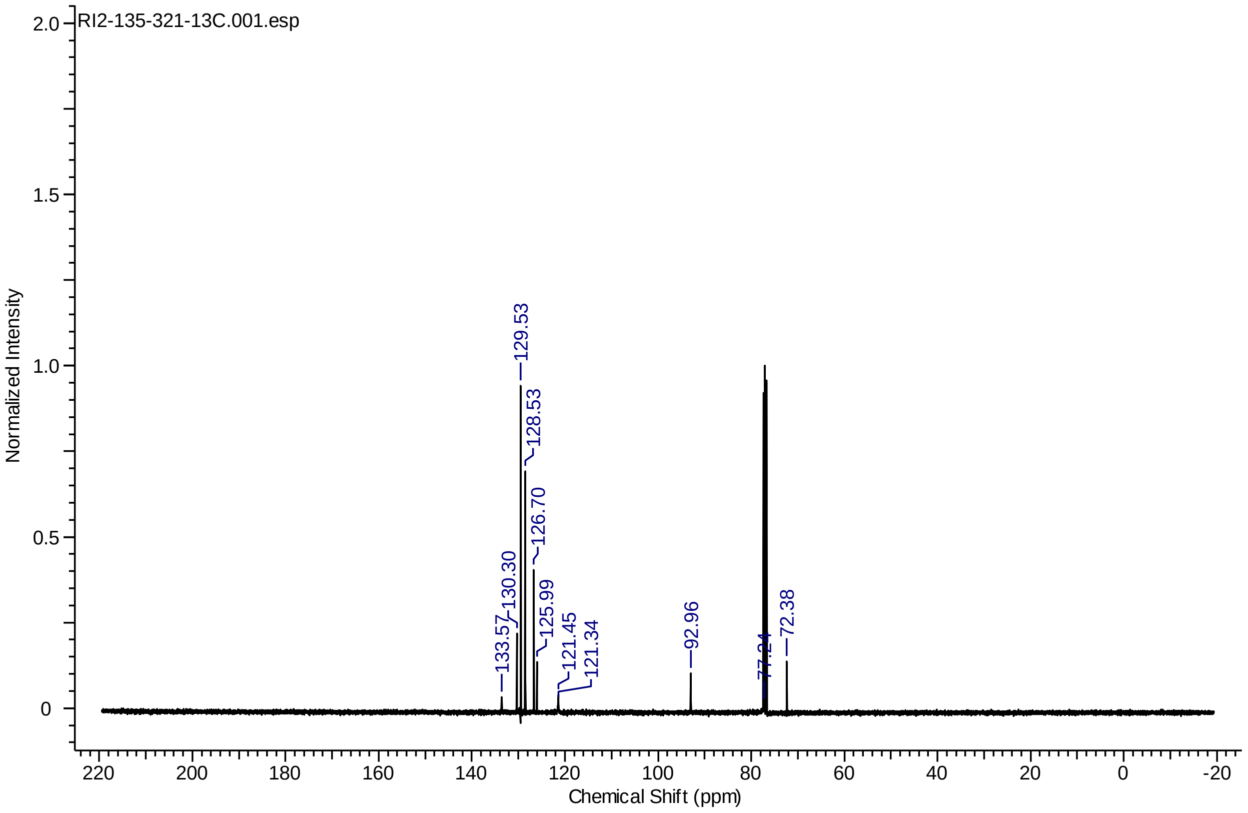
RI2-135-321-13C.001.esp
Normalized Intensity
Chemical Shift (ppm)
133.57
130.30
129.53
128.53
126.70
125.99
121.45
121.34
92.96
77.24
72.38

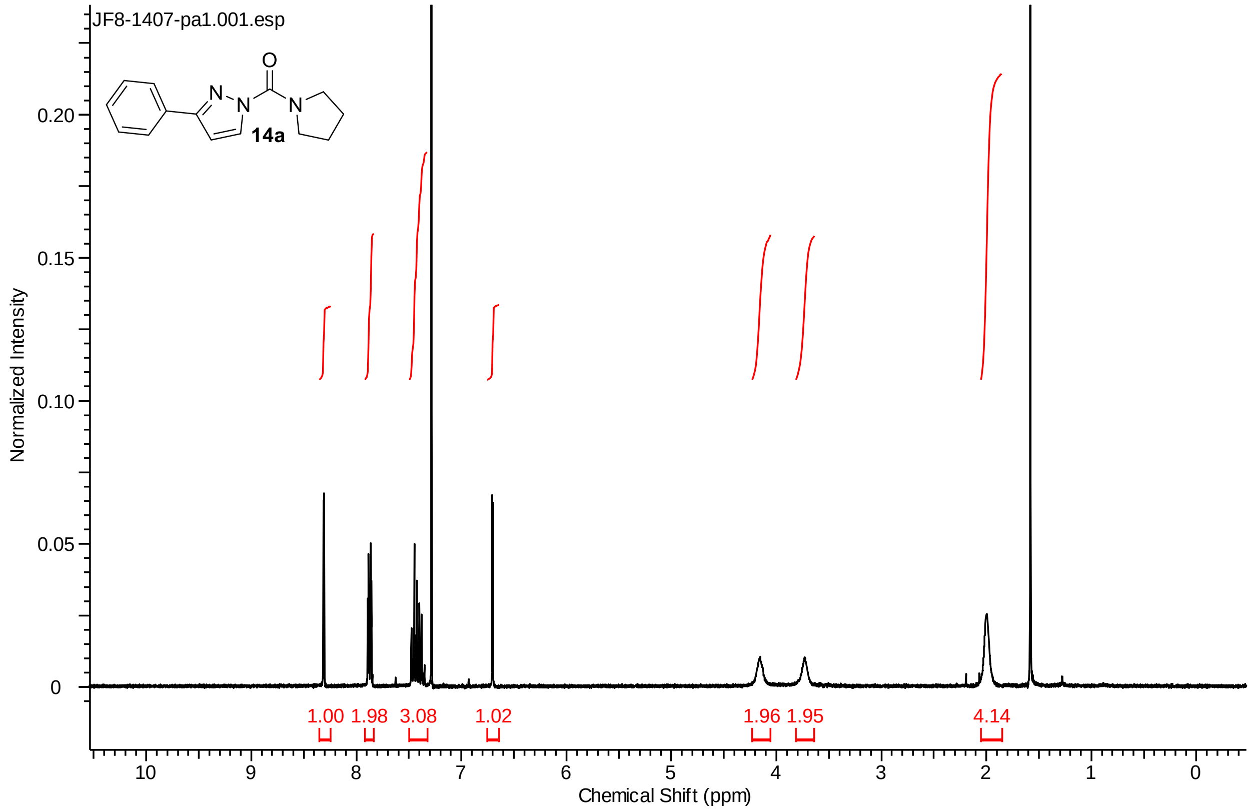
JF8-1407-pa1.001.esp
O
N
N
N
14a
Normalized Intensity
1.00
1.98
3.08
1.02
1.96
1.95
4.14
Chemical Shift (ppm)

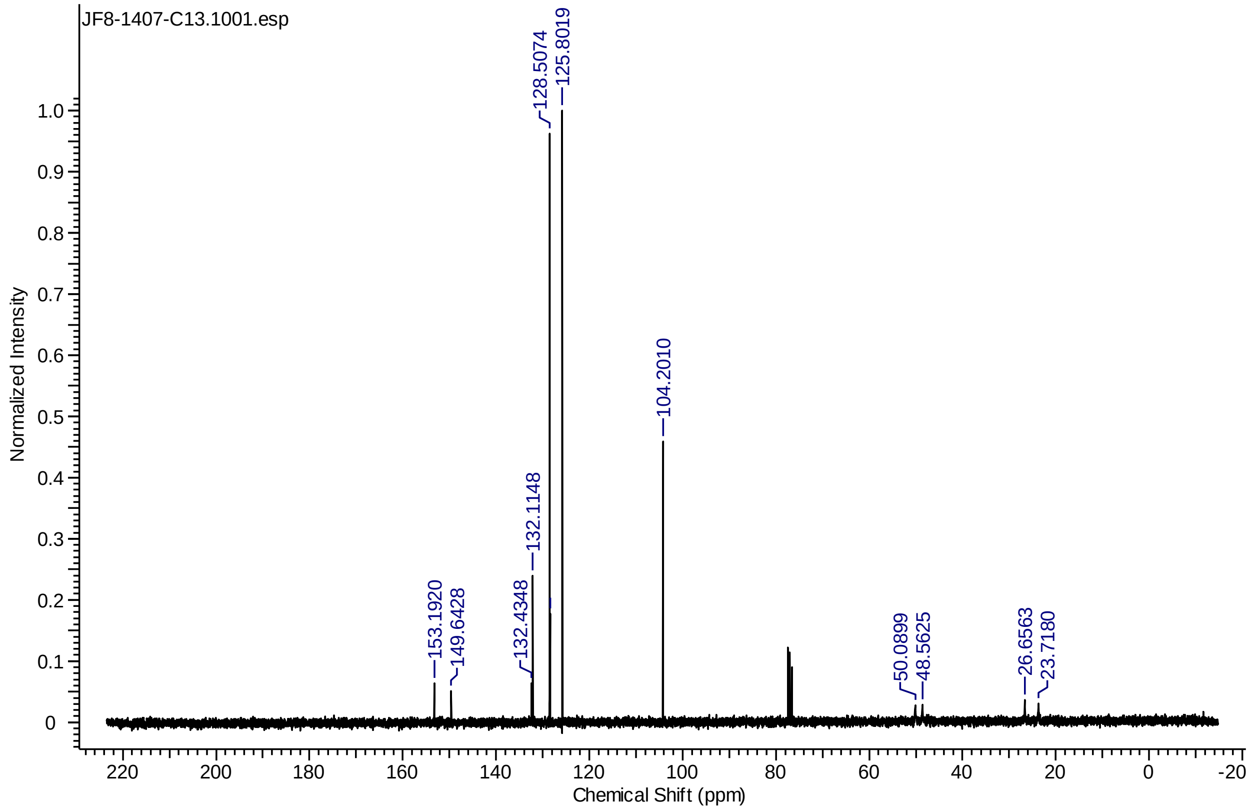
JF8-1407-C13.1001.esp
Normalized Intensity
153.1920
149.6428
132.4348
132.1148
128.5074
125.8019
104.2010
50.0899
48.5625
26.6563
23.7180
Chemical Shift (ppm)

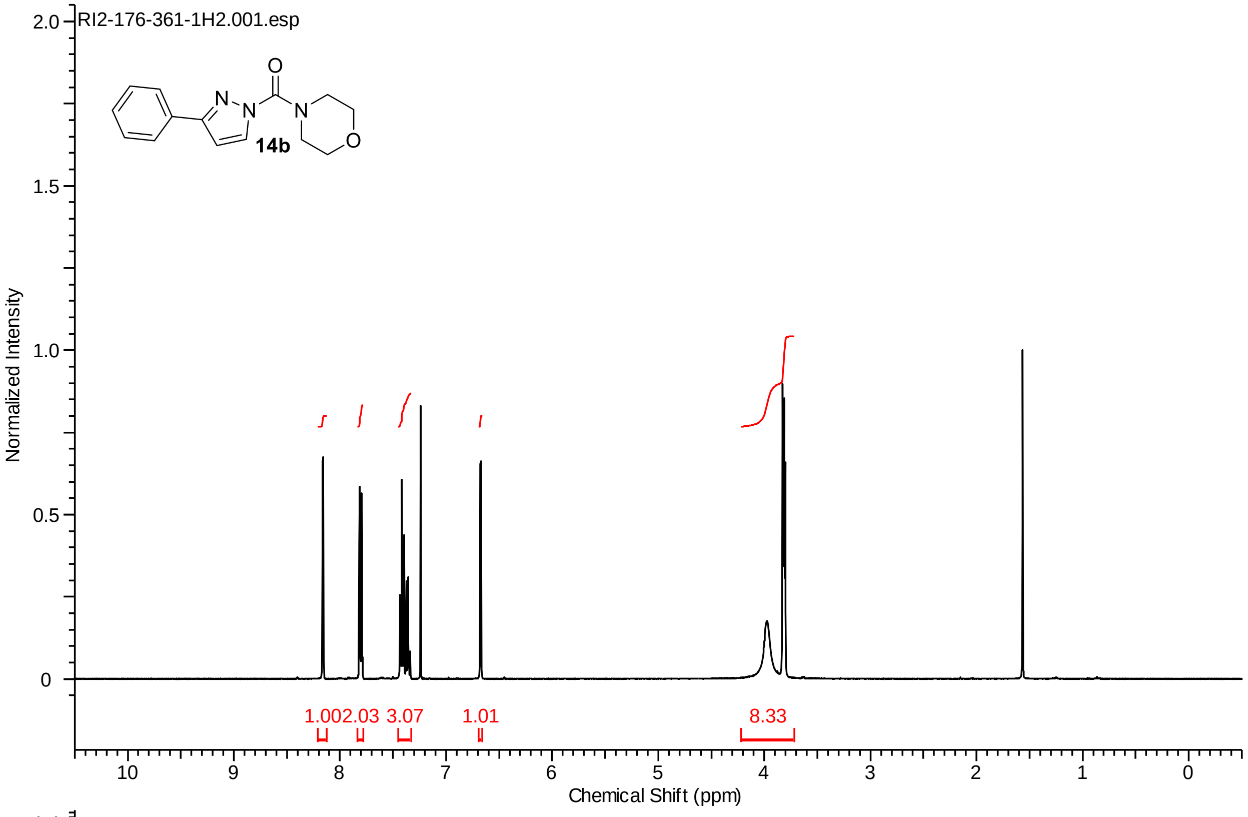
RI2-176-361-1H2.001.esp
14b
Normalized Intensity
1.00
2.03
3.07
1.01
8.33
Chemical Shift (ppm)

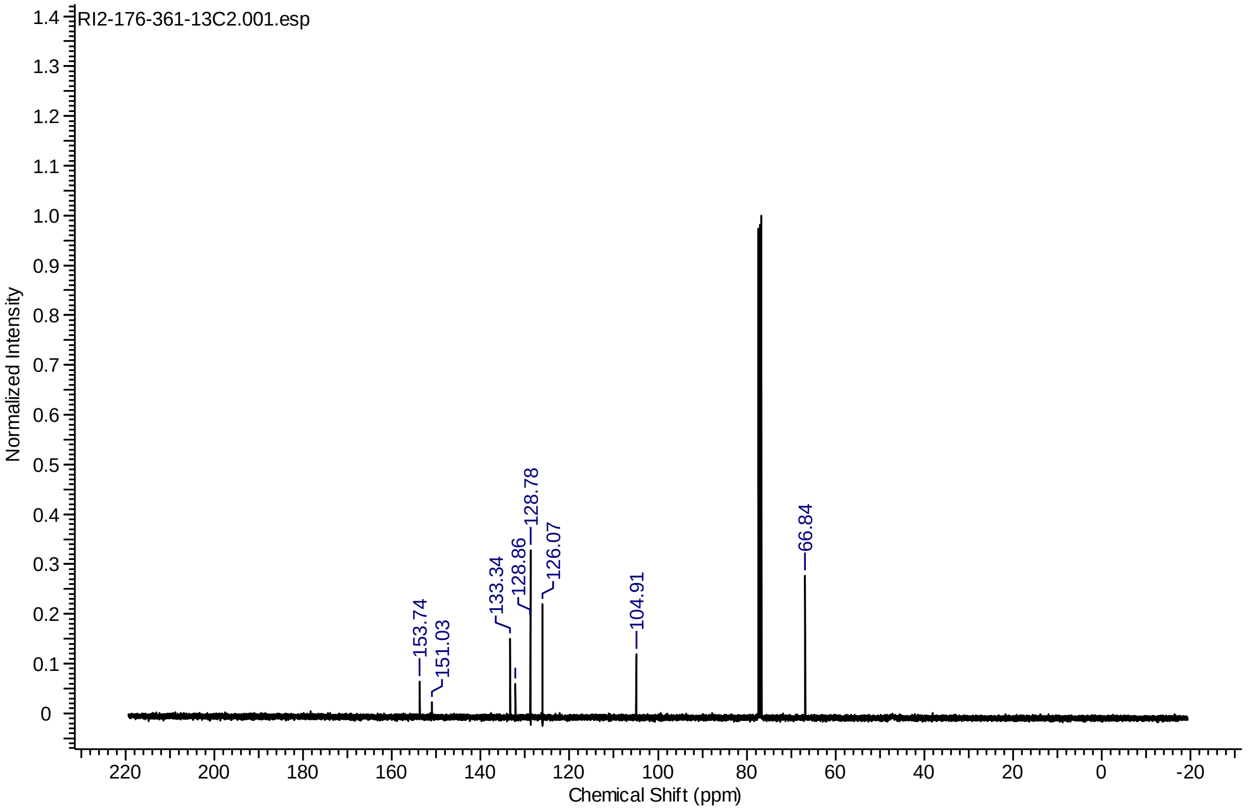
RI2-176-361-13C2.001.esp
Normalized Intensity
153.74
151.03
133.34
128.86
128.78
126.07
104.91
66.84
Chemical Shift (ppm)

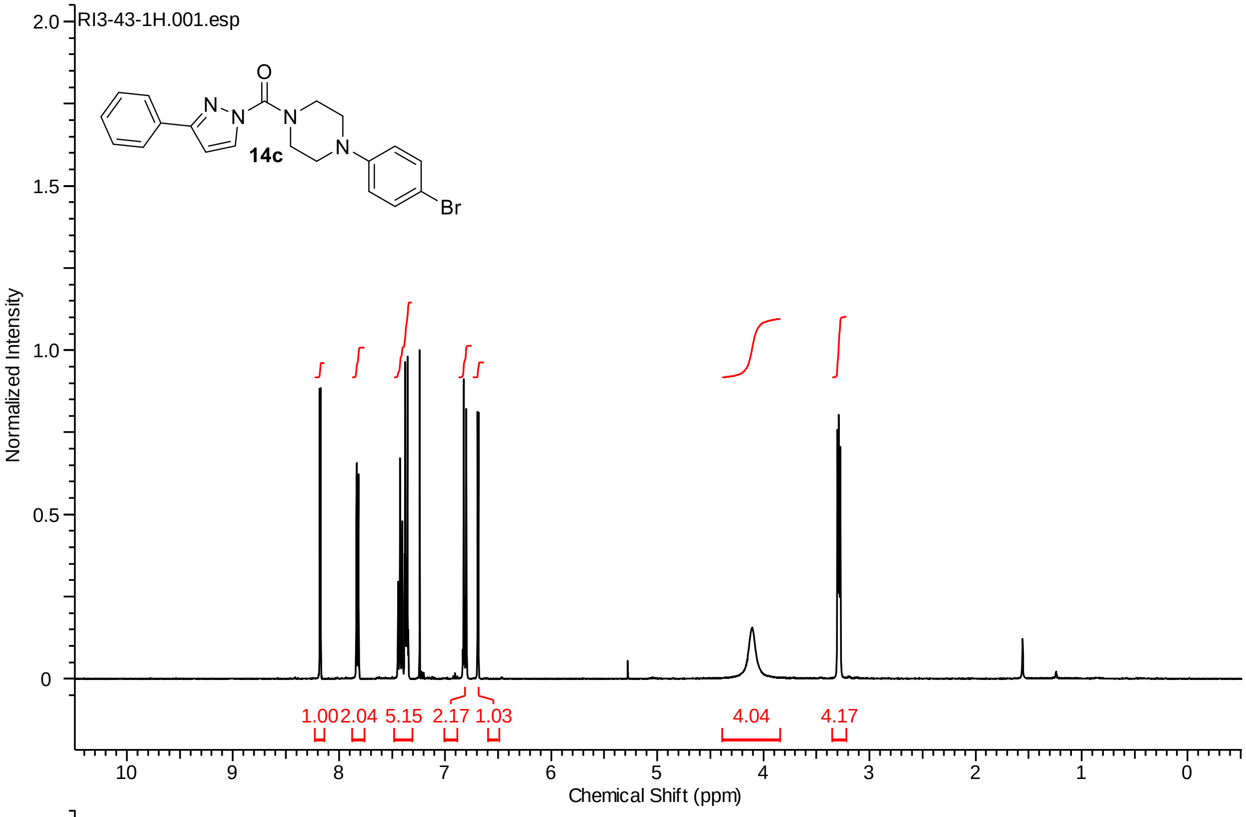
RI3-43-1H.001.esp
Normalized Intensity
2.0
1.5
1.0
0.5
0
O
N
N
N
N
14c
Br
1.00
2.04
5.15
2.17
1.03
4.04
4.17
10
9
8
7
6
5
4
3
2
1
0
Chemical Shift (ppm)

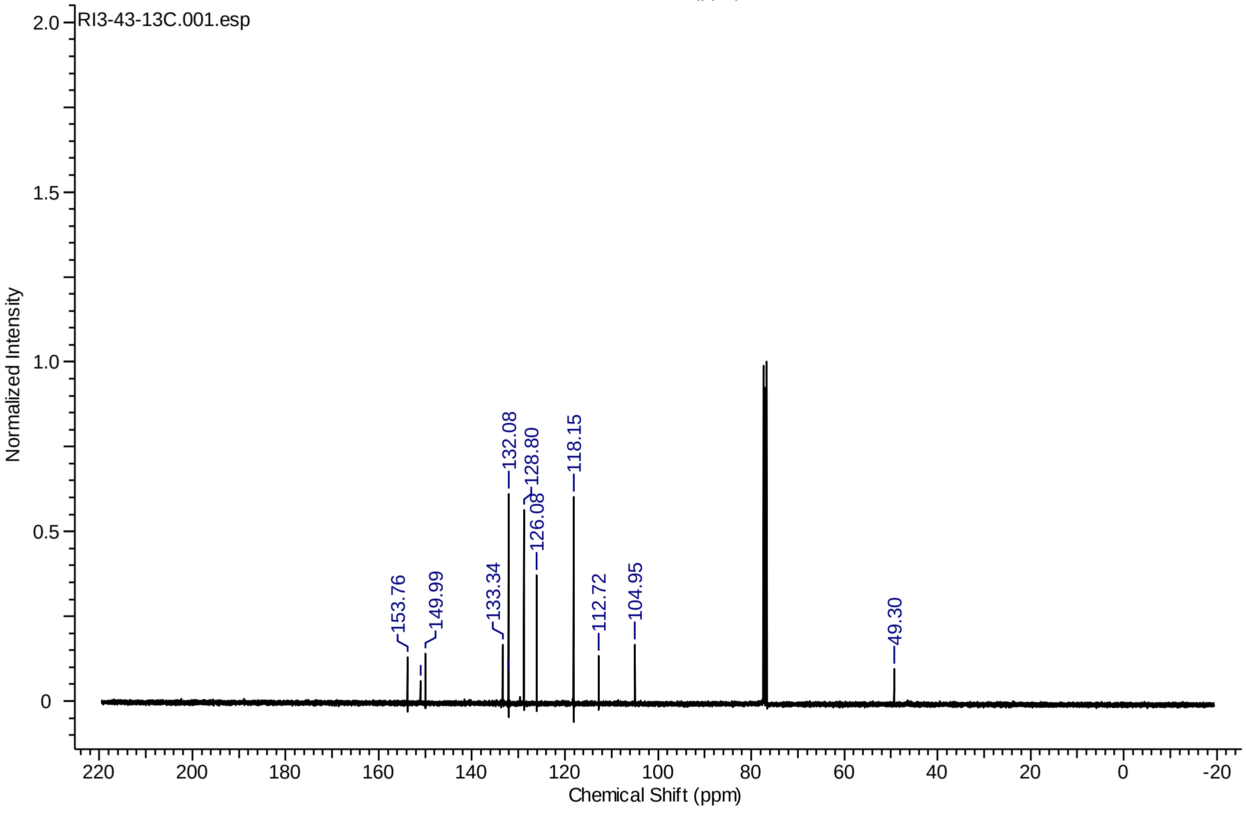
RI3-43-13C.001.esp
Normalized Intensity
2.0
1.5
1.0
0.5
0
153.76
149.99
133.34
132.08
128.80
126.08
118.15
112.72
104.95
49.30
220
200
180
160
140
120
100
80
60
40
20
0
-20
Chemical Shift (ppm)

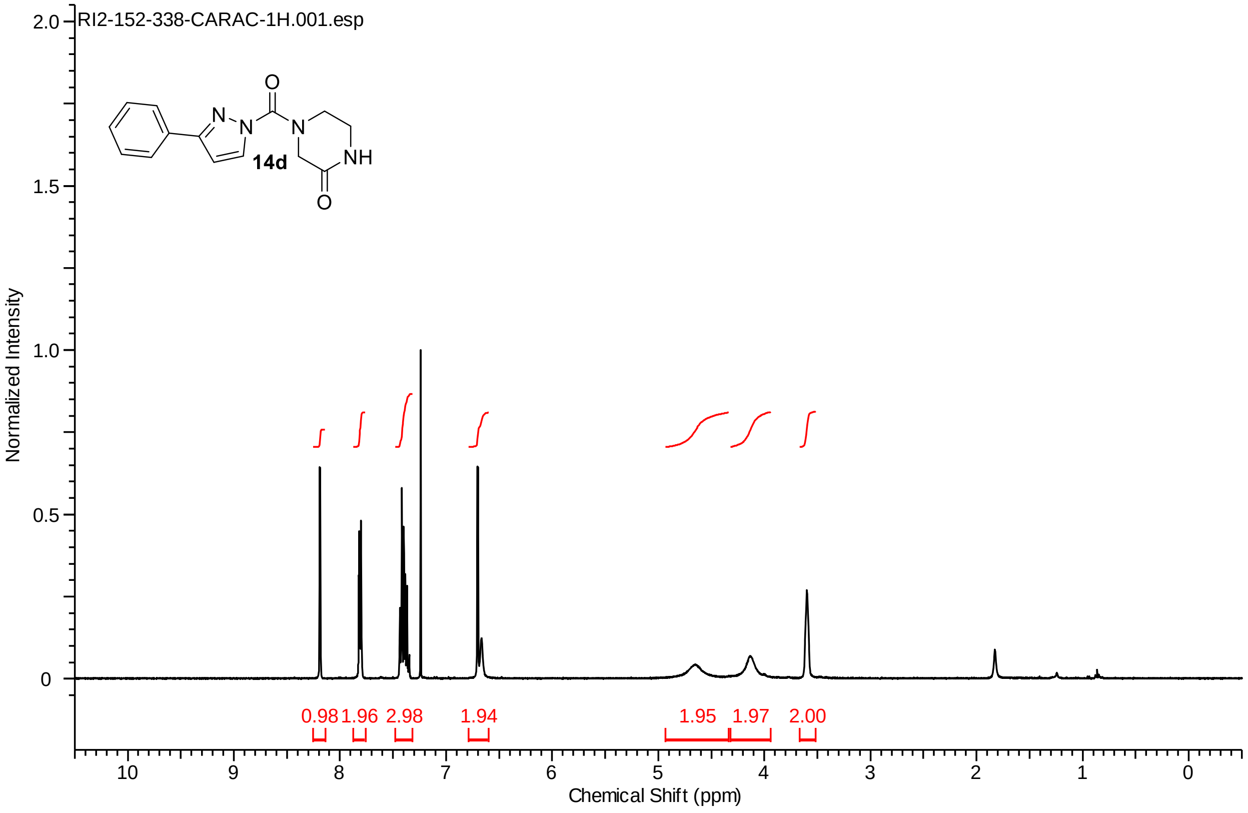
RI2-152-338-CARAC-1H.001.esp
14d
Normalized Intensity
Chemical Shift (ppm)
0.98
1.96
2.98
1.94
1.95
1.97
2.00

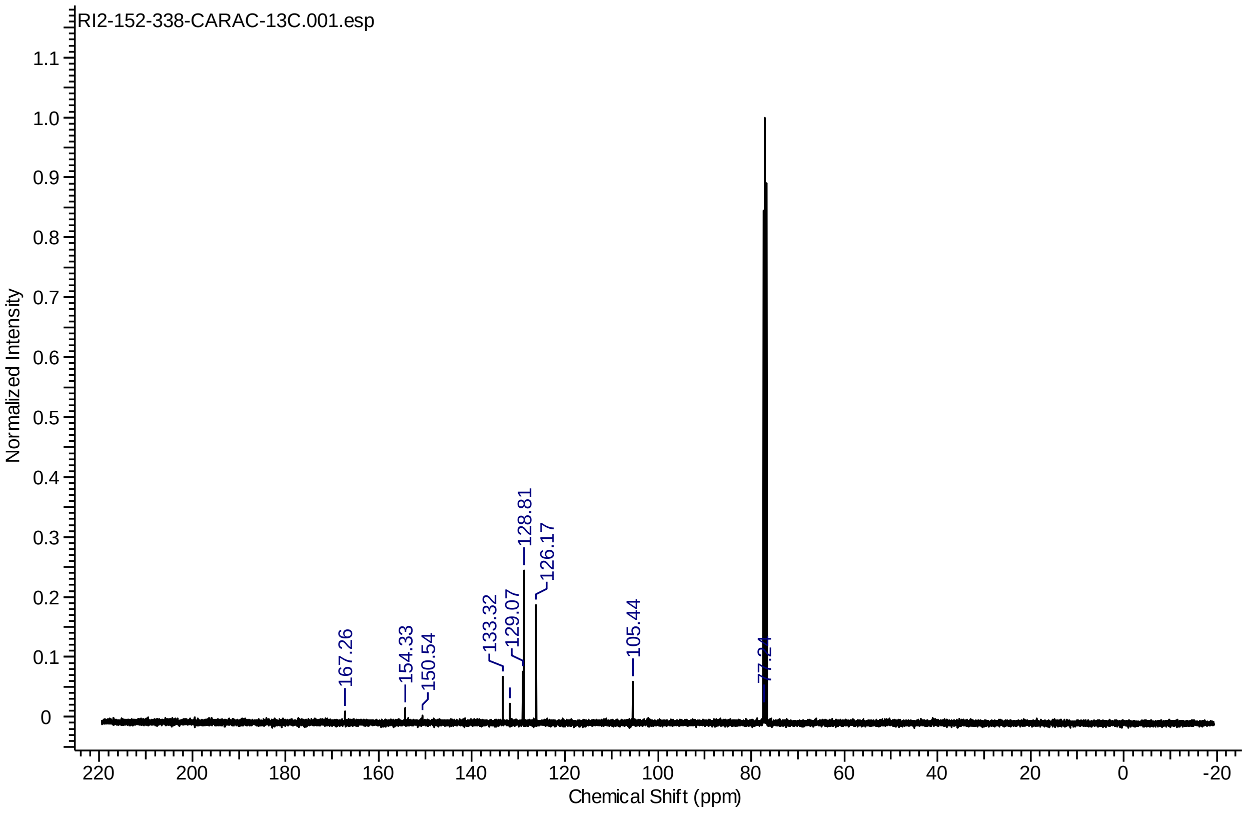
RI2-152-338-CARAC-13C.001.esp
Normalized Intensity
Chemical Shift (ppm)
167.26
154.33
150.54
133.32
129.07
128.81
126.17
105.44
77.24

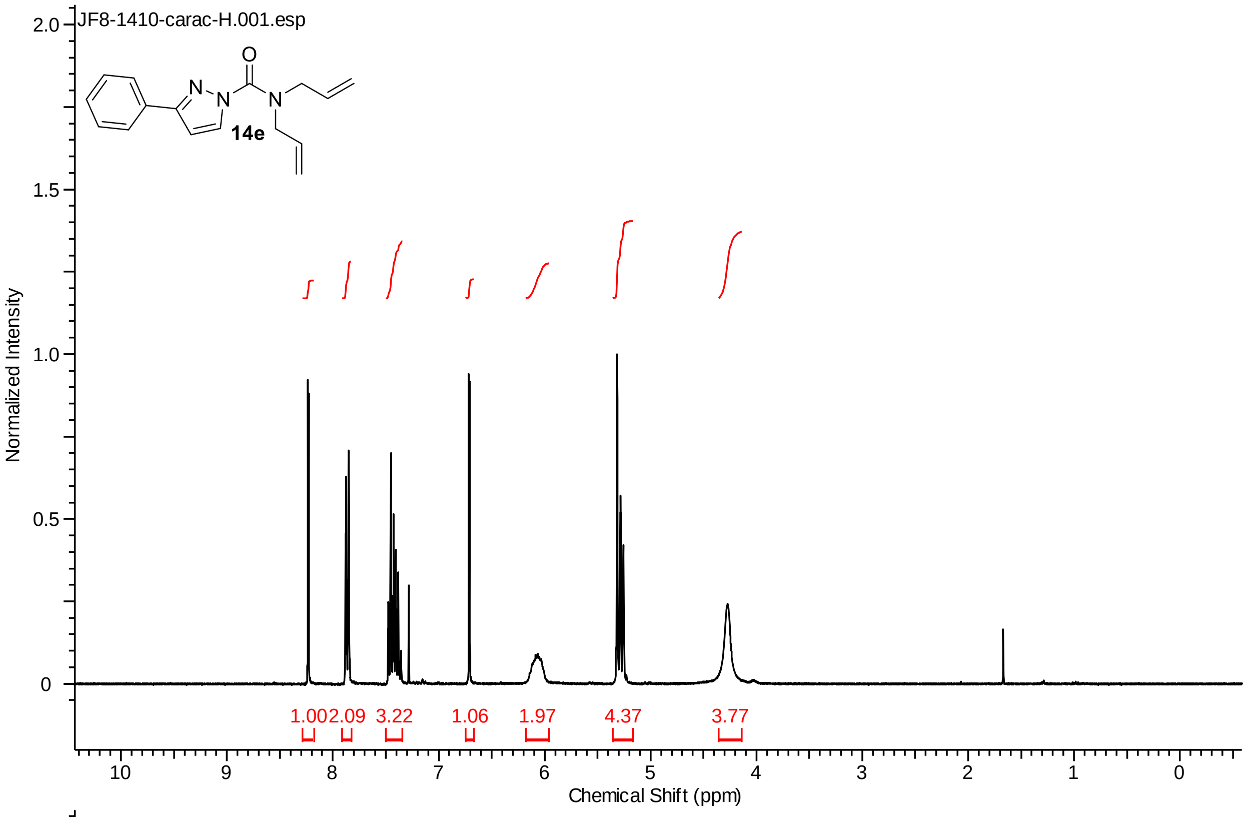
JF8-1410-carac-H.001.esp
14e
Normalized Intensity
1.00
2.09
3.22
1.06
1.97
4.37
3.77
Chemical Shift (ppm)

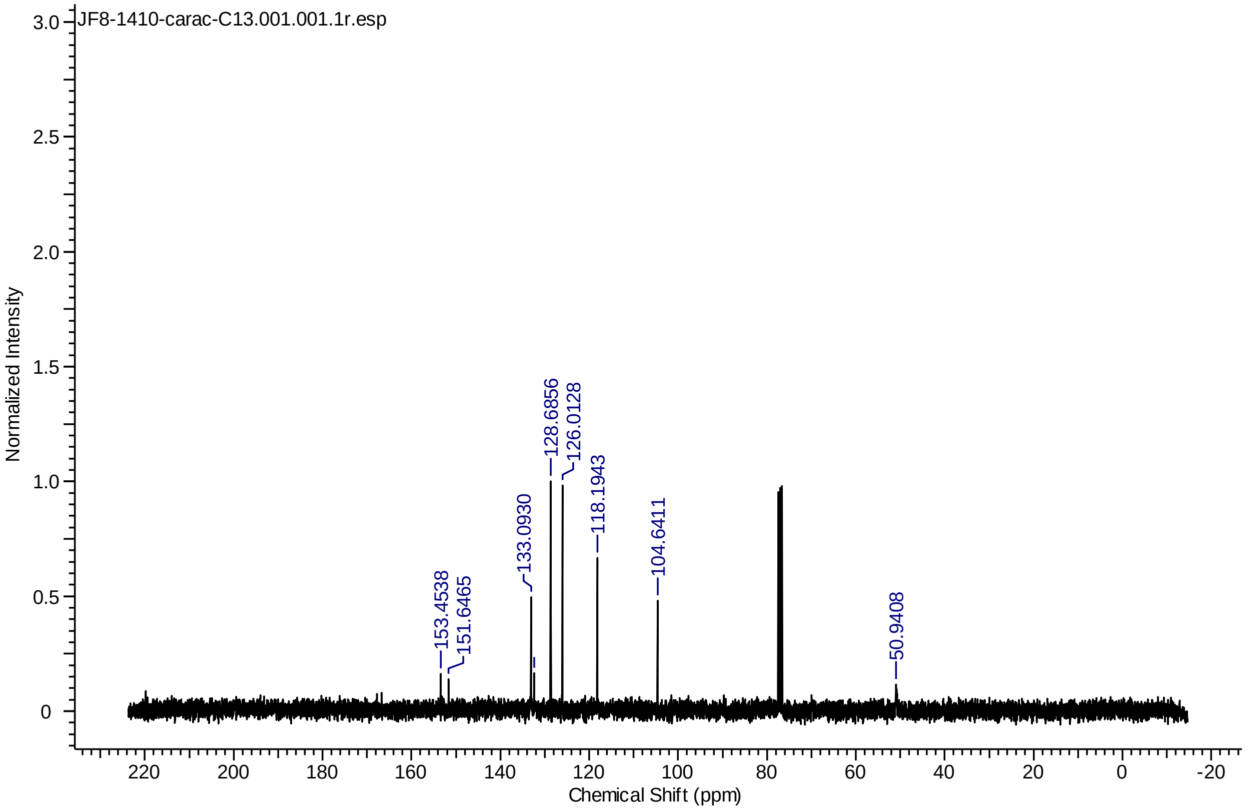
JF8-1410-carac-C13.001.001.1r.esp
Normalized Intensity
153.4538
151.6465
133.0930
128.6856
126.0128
118.1943
104.6411
50.9408
Chemical Shift (ppm)

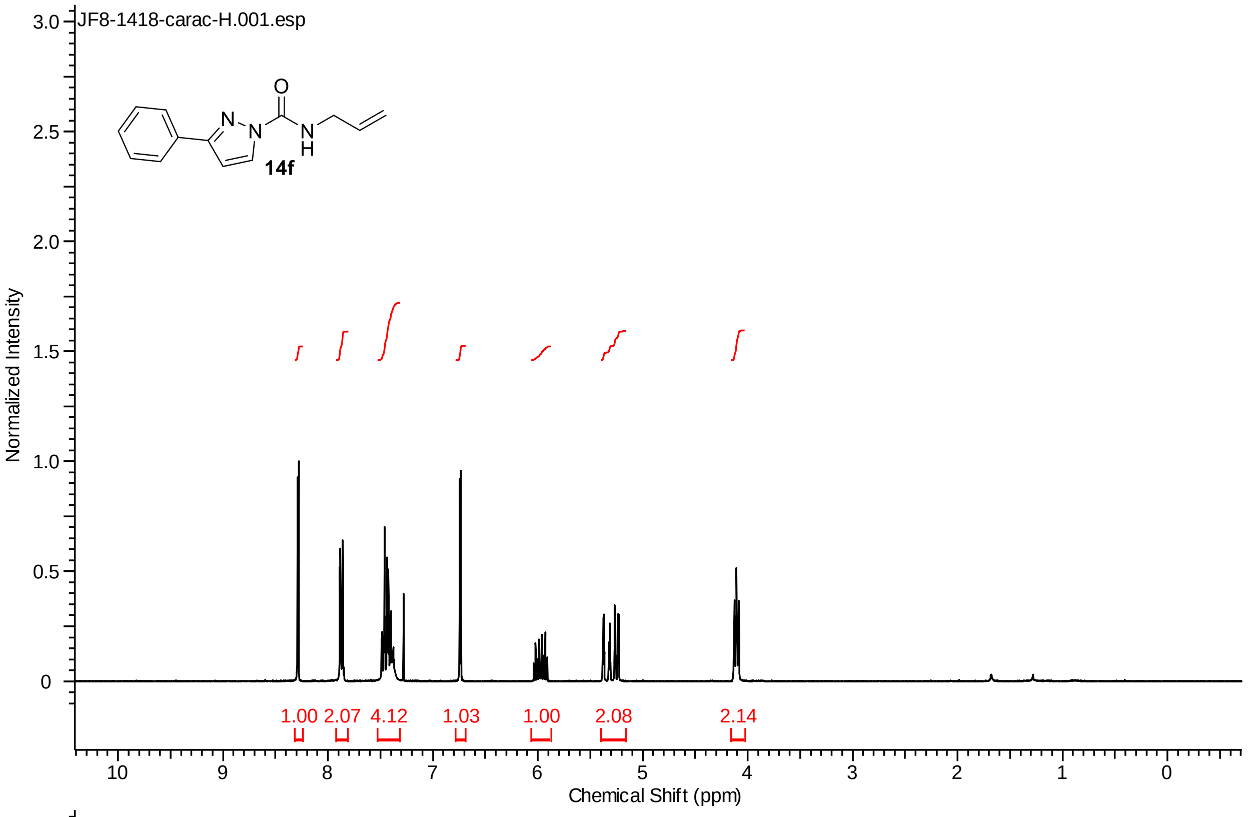
JF8-1418-carac-H.001.esp
O
N
N
N
H
14f
Normalized Intensity
1.00 2.07 4.12 1.03 1.00 2.08 2.14
Chemical Shift (ppm)

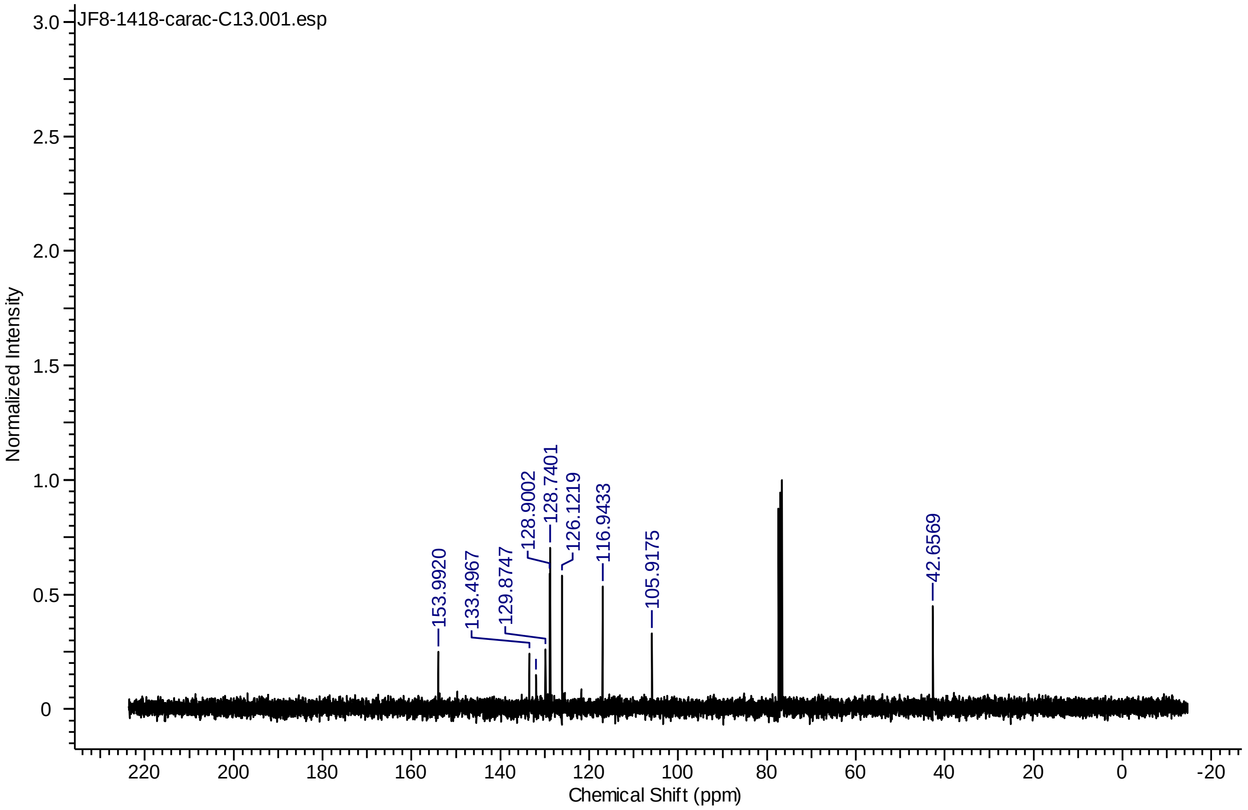
JF8-1418-carac-C13.001.esp
Normalized Intensity
153.9920
133.4967
129.8747
128.9002
128.7401
126.1219
116.9433
105.9175
42.6569
Chemical Shift (ppm)

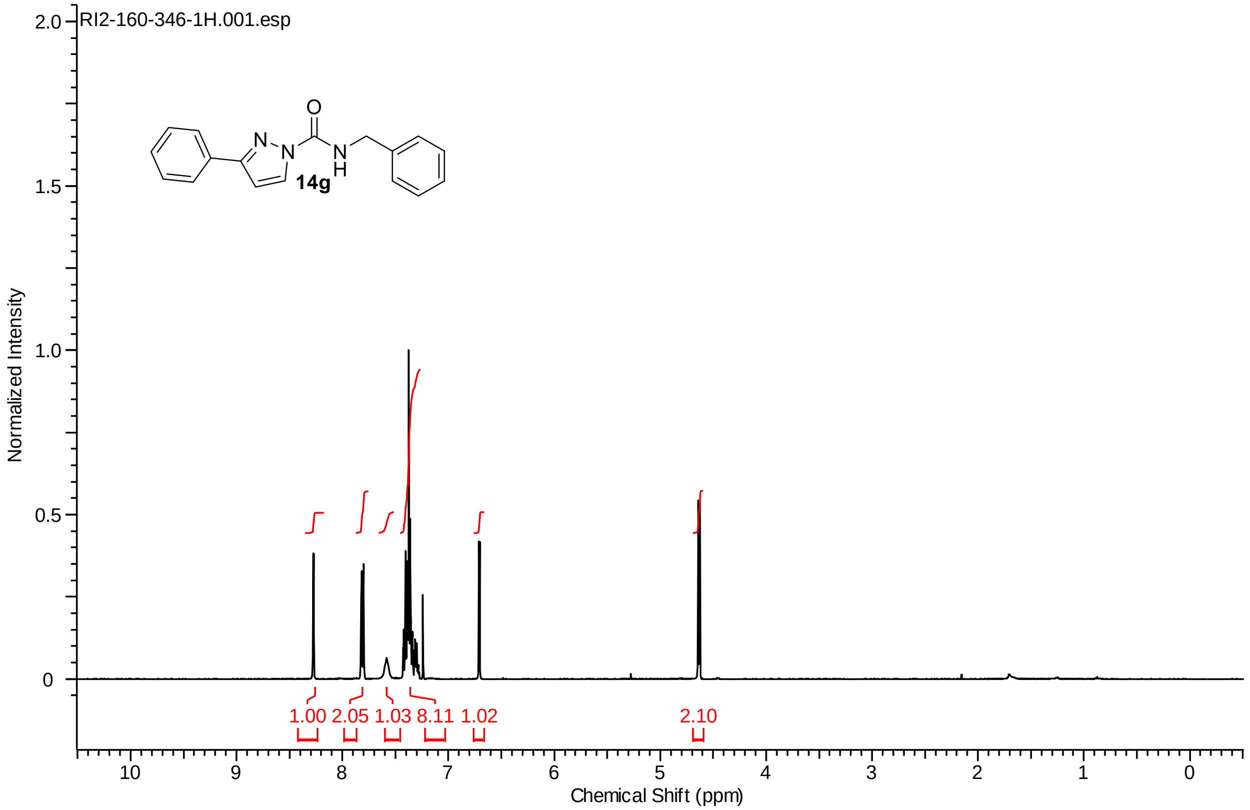
RI2-160-346-1H.001.esp
14g
1.00
2.05
1.03
8.11
1.02
2.10
Normalized Intensity
Chemical Shift (ppm)

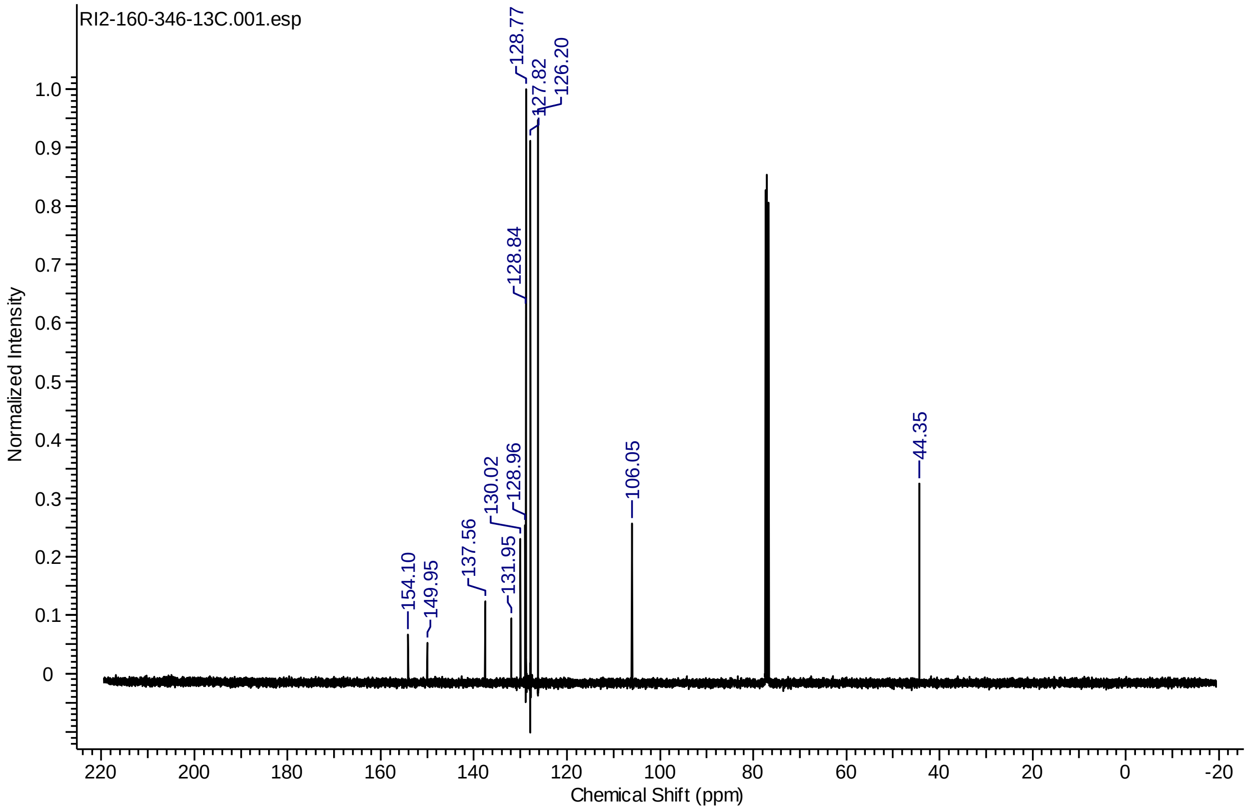
RI2-160-346-13C.001.esp
128.77
127.82
126.20
128.84
154.10
149.95
137.56
131.95
130.02
128.96
106.05
44.35
Normalized Intensity
Chemical Shift (ppm)

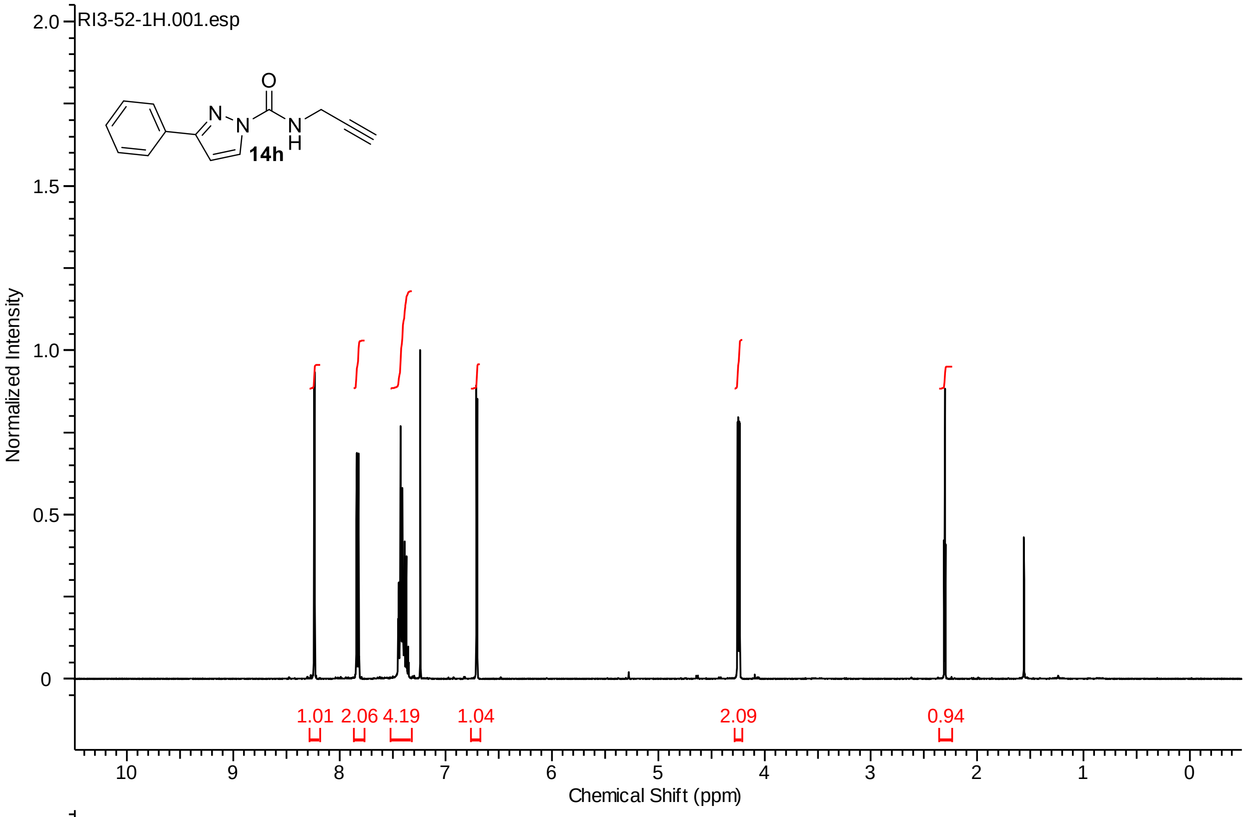
RI3-52-1H.001.esp
14h
1.01
2.06
4.19
1.04
2.09
0.94
Normalized Intensity
Chemical Shift (ppm)

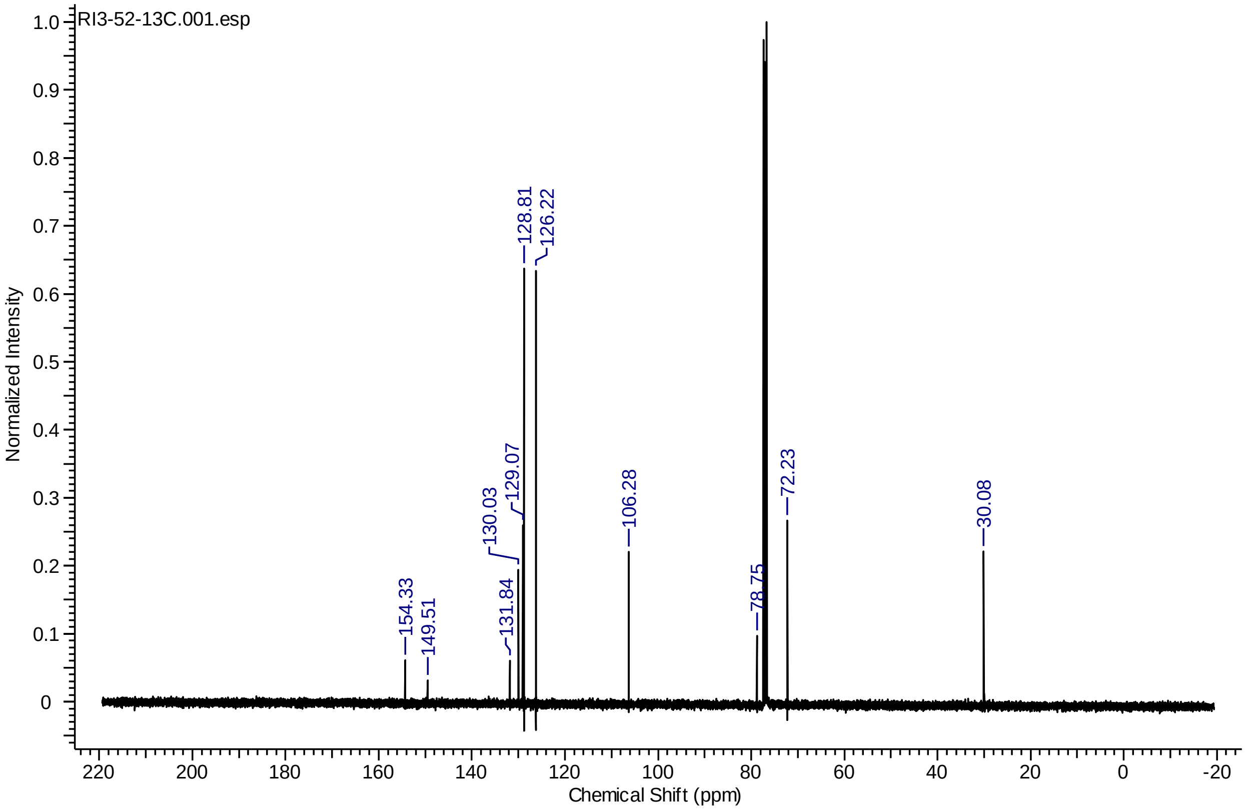
RI3-52-13C.001.esp
154.33
149.51
131.84
130.03
129.07
128.81
126.22
106.28
78.75
72.23
30.08
Normalized Intensity
Chemical Shift (ppm)

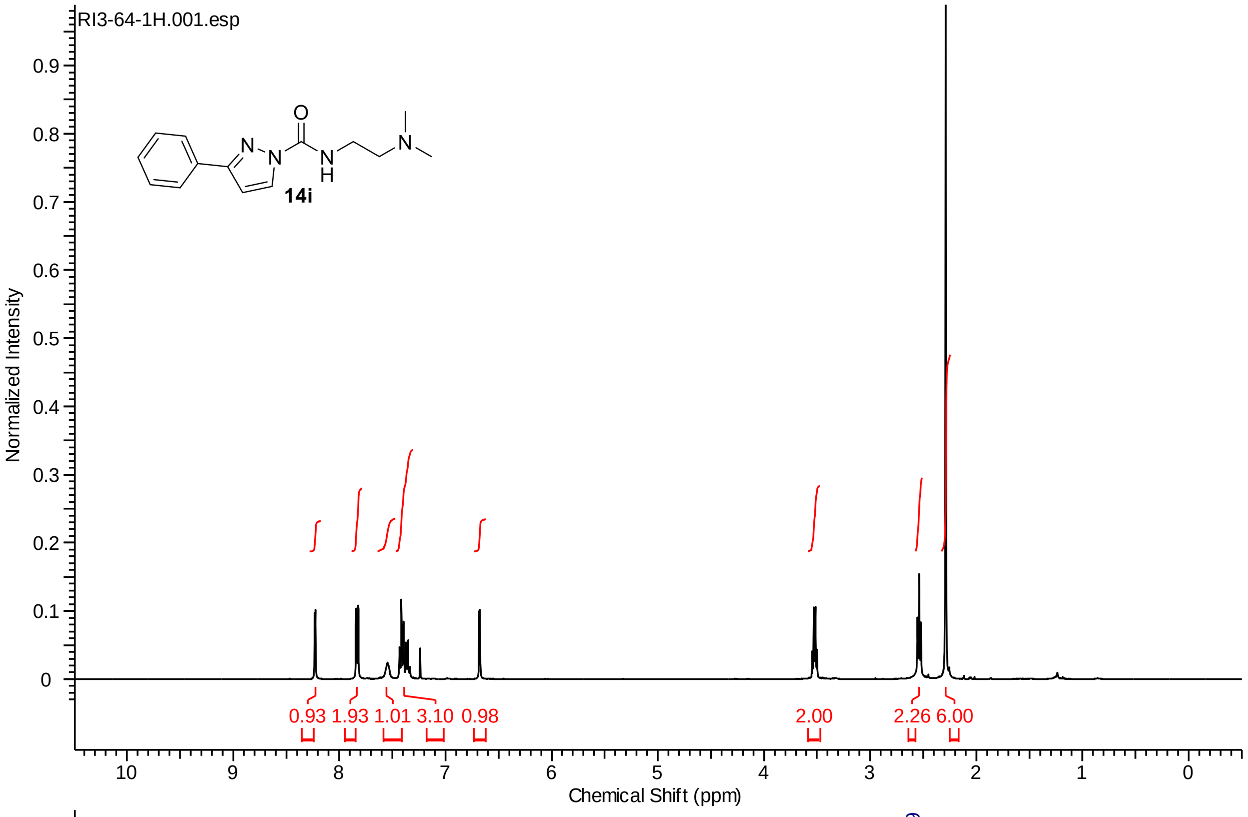
RI3-64-1H.001.esp
14i
0.93
1.93
1.01
3.10
0.98
2.00
2.26
6.00
Normalized Intensity
Chemical Shift (ppm)

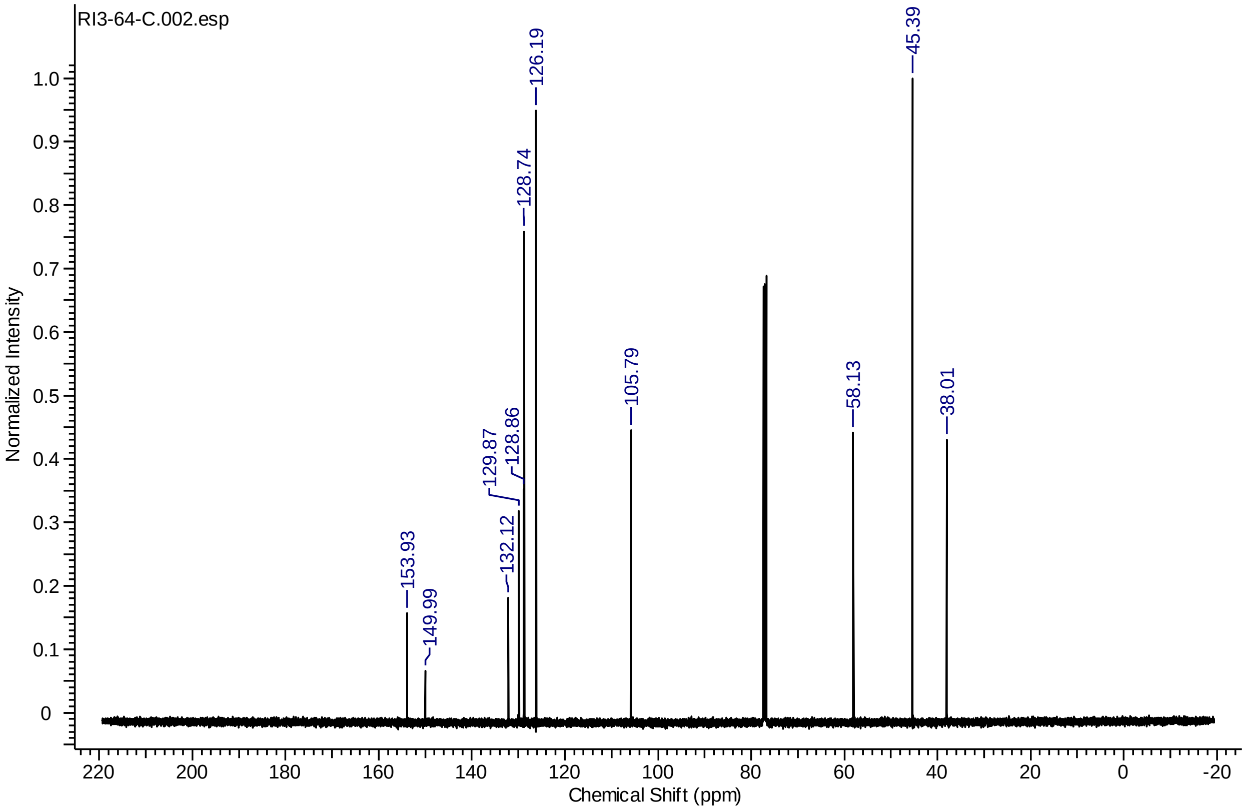
RI3-64-C.002.esp
153.93
149.99
132.12
129.87
128.86
128.74
126.19
105.79
58.13
45.39
38.01
Normalized Intensity
Chemical Shift (ppm)

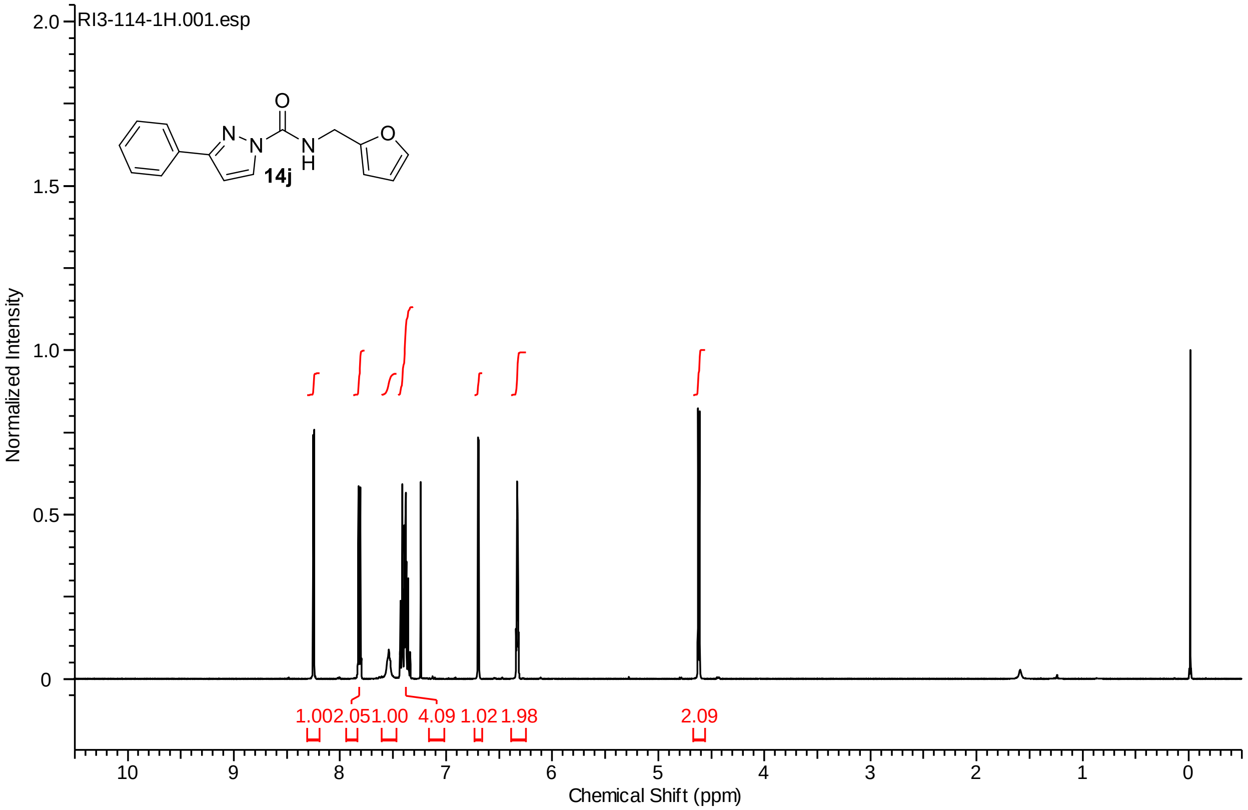
RI3-114-1H.001.esp
14j
Normalized Intensity
Chemical Shift (ppm)
1.00
2.05
1.00
4.09
1.02
1.98
2.09

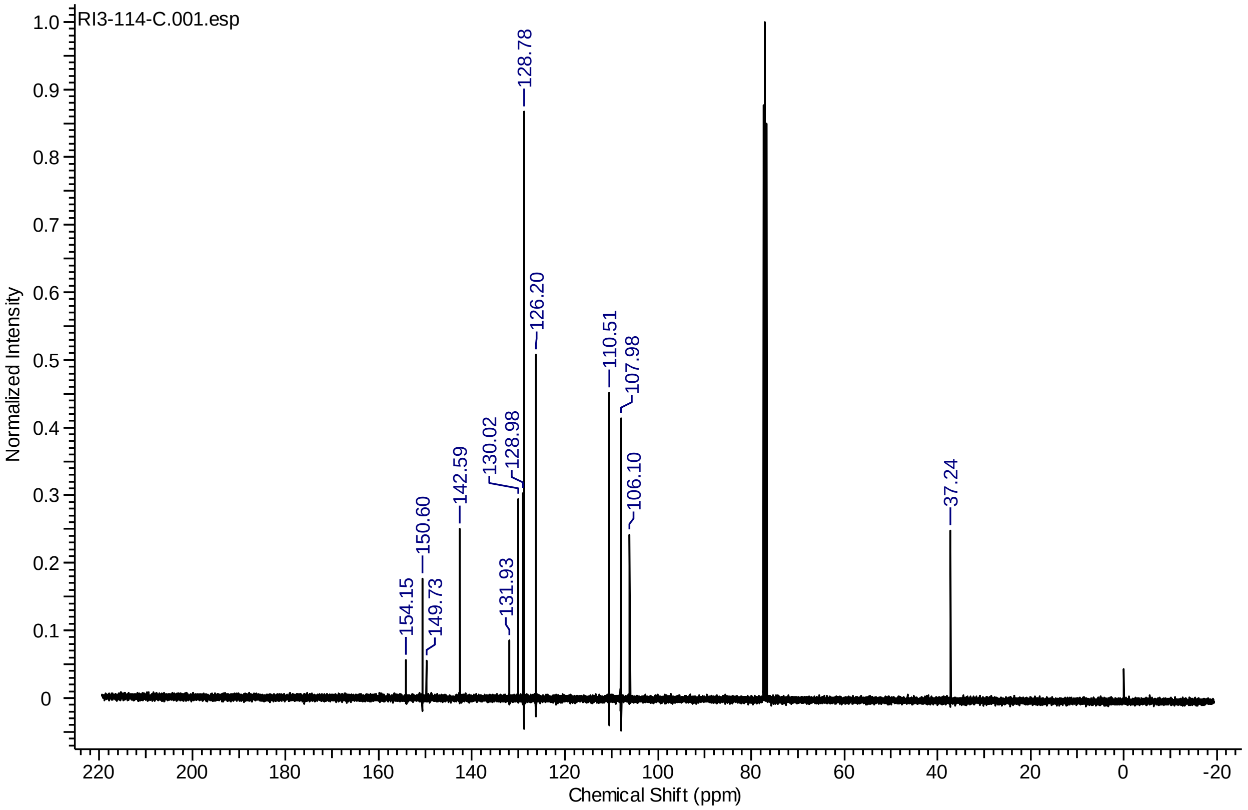
RI3-114-C.001.esp
Normalized Intensity
Chemical Shift (ppm)
154.15
150.60
149.73
142.59
131.93
130.02
128.98
128.78
126.20
110.51
107.98
106.10
37.24

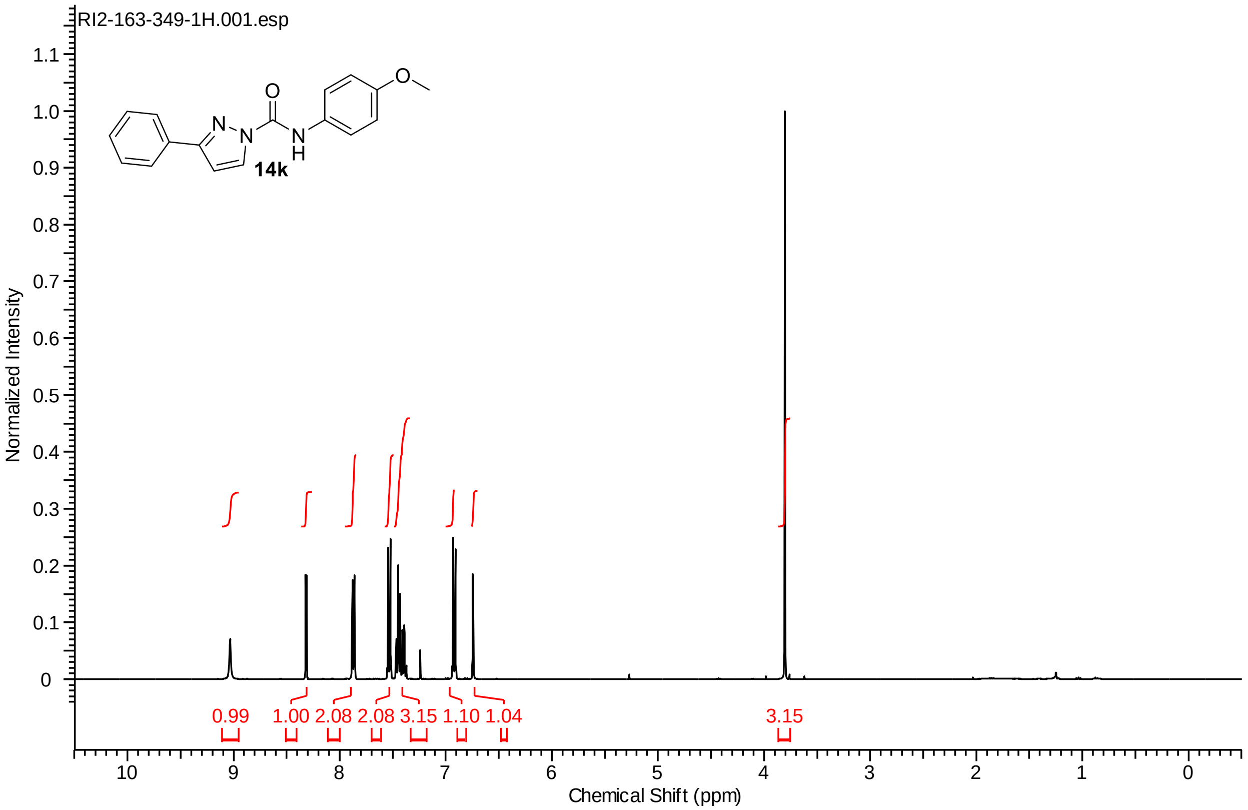
RI2-163-349-1H.001.esp
14k
Normalized Intensity
0.99
1.00
2.08
2.08
3.15
1.10
1.04
3.15
Chemical Shift (ppm)

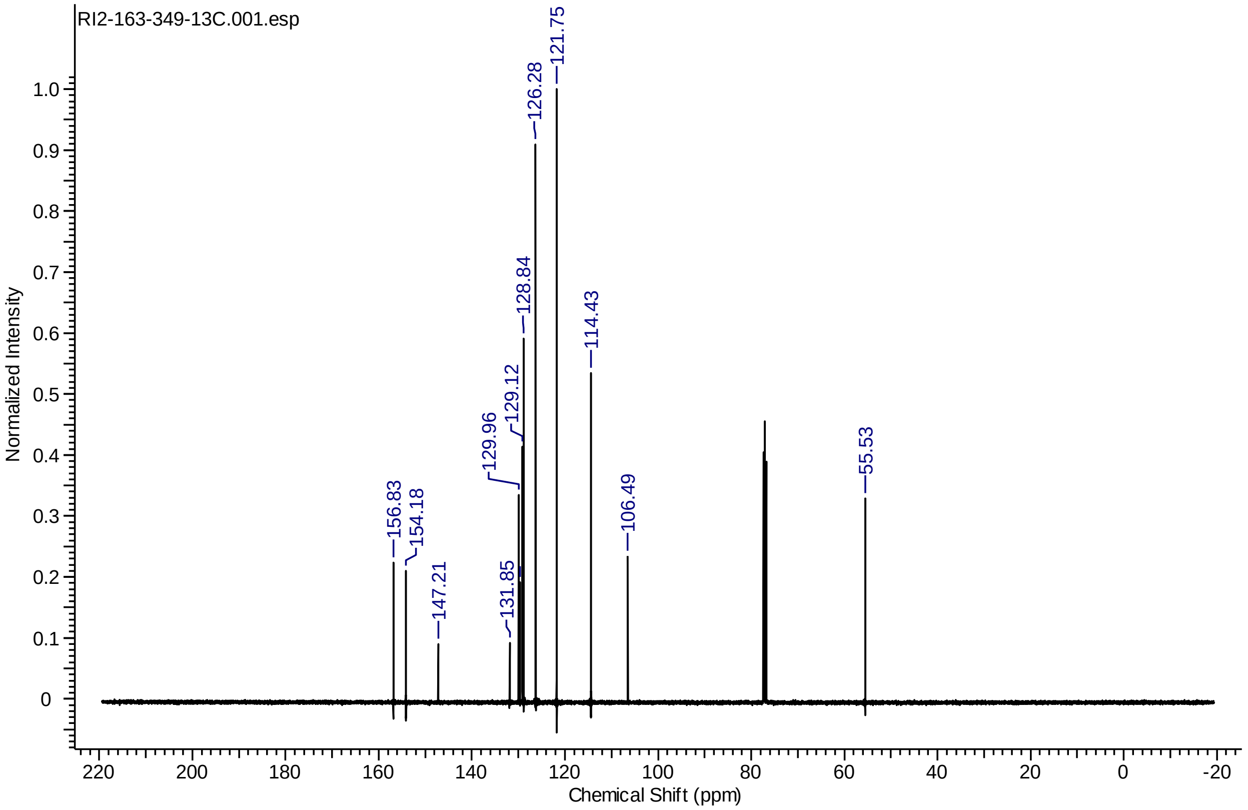
RI2-163-349-13C.001.esp
Normalized Intensity
156.83
154.18
147.21
131.85
129.96
129.12
128.84
126.28
121.75
114.43
106.49
55.53
Chemical Shift (ppm)

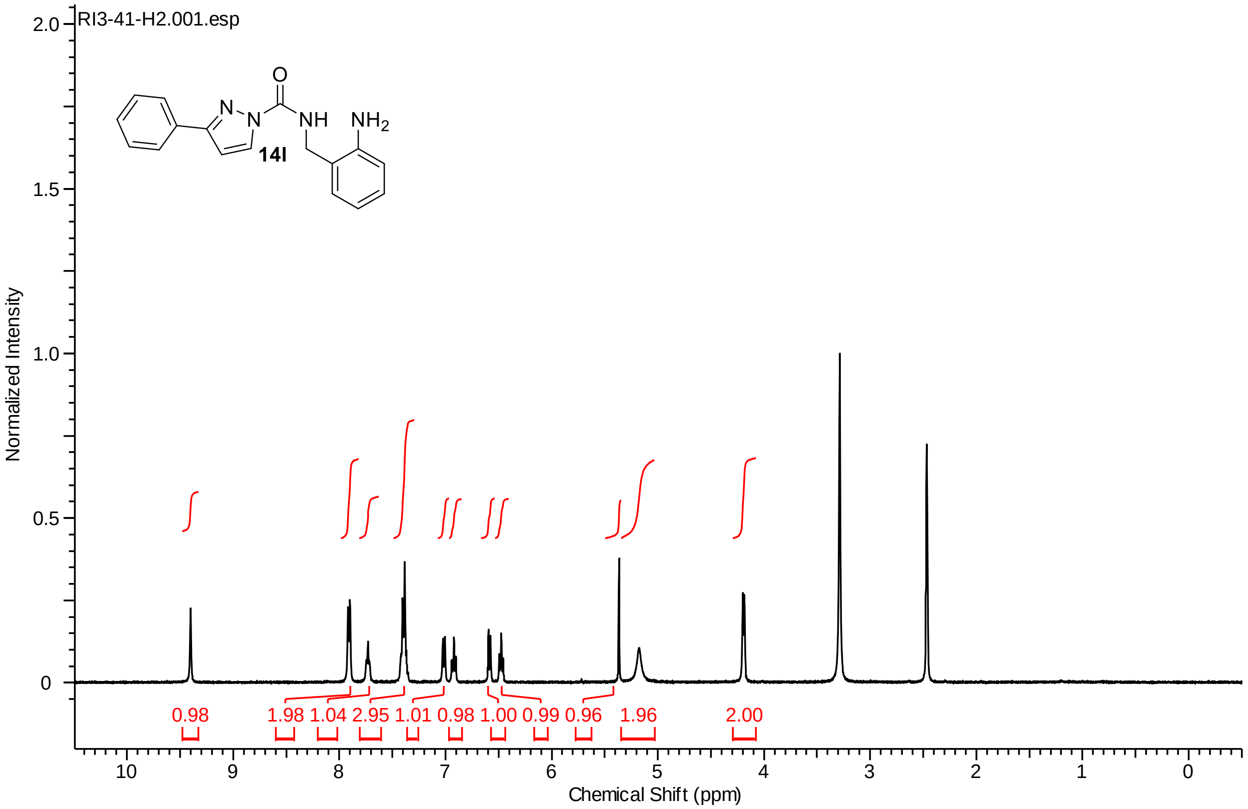
RI3-41-H2.001.esp
14l
NH
NH_2
0.98
1.98
1.04
2.95
1.01
0.98
1.00
0.99
0.96
1.96
2.00
Normalized Intensity
Chemical Shift (ppm)

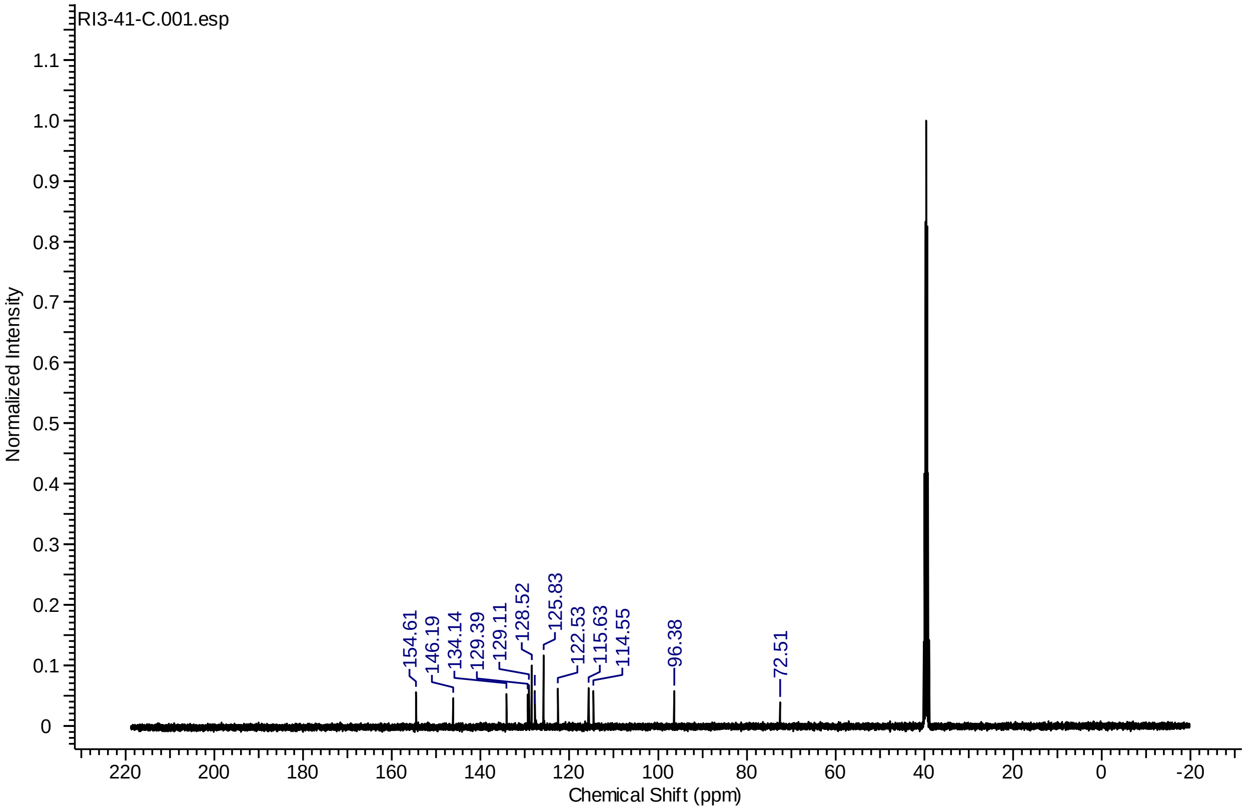
RI3-41-C.001.esp
154.61
146.19
134.14
129.39
129.11
128.52
125.83
122.53
115.63
114.55
96.38
72.51
Normalized Intensity
Chemical Shift (ppm)

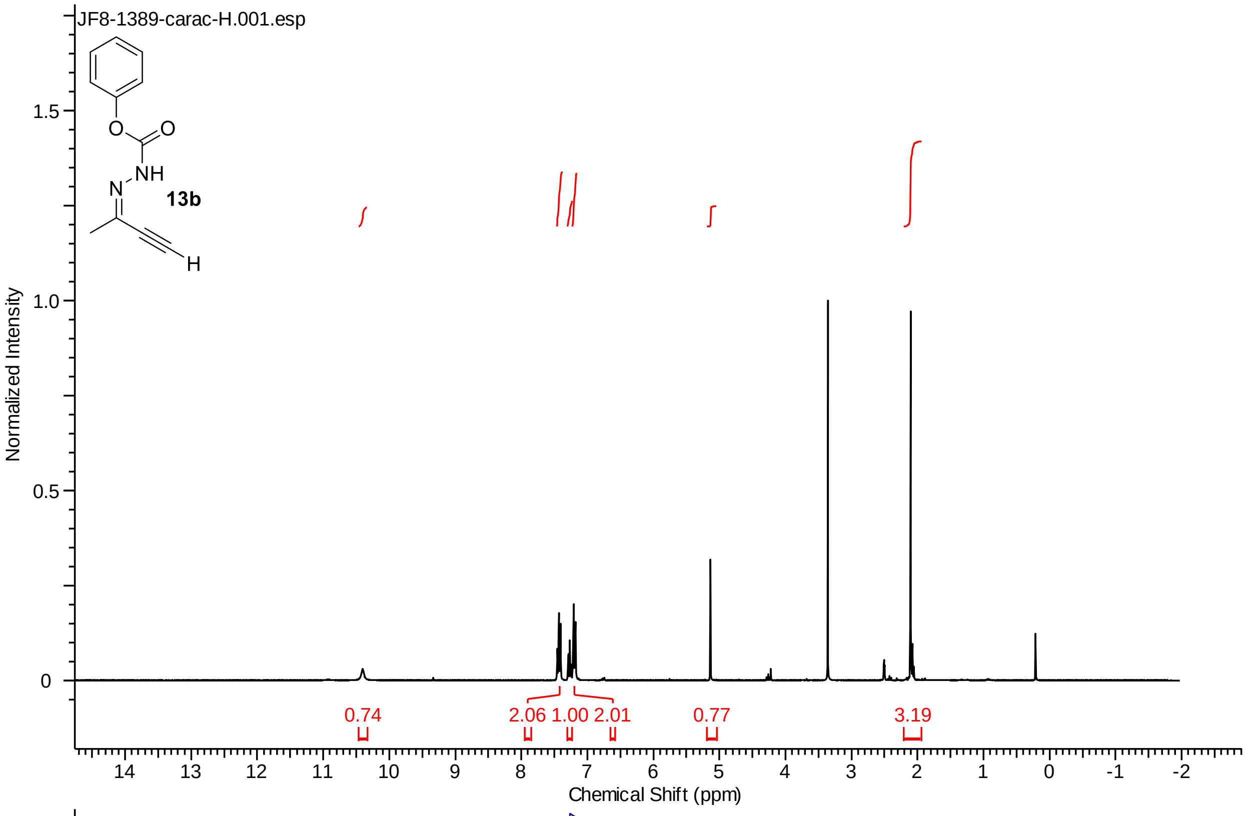
JF8-1389-carac-H.001.esp
O
O
NH
N
13b
H
Normalized Intensity
1.5
1.0
0.5
0
0.74
2.06 1.00 2.01
0.77
3.19
14 13 12 11 10 9 8 7 6 5 4 3 2 1 0 -1 -2
Chemical Shift (ppm)

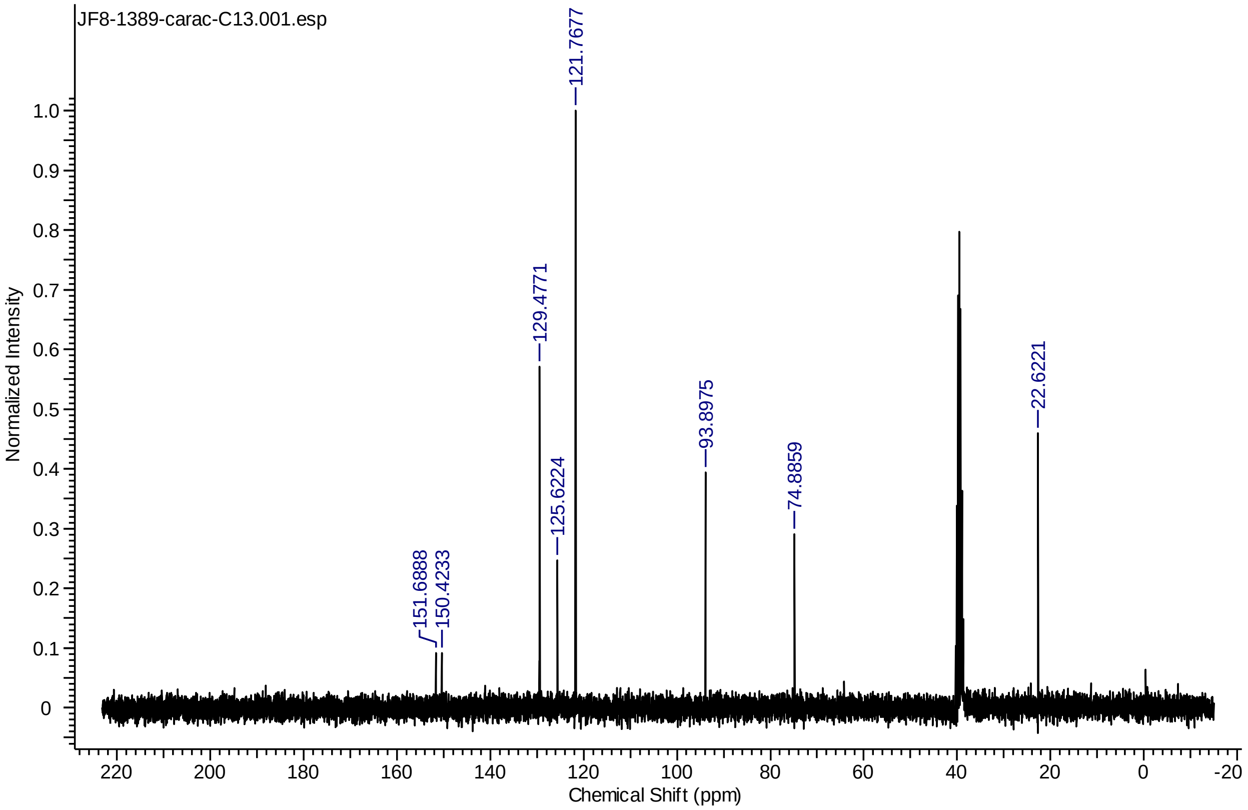
JF8-1389-carac-C13.001.esp
Normalized Intensity
1.0
0.9
0.8
0.7
0.6
0.5
0.4
0.3
0.2
0.1
0
151.6888
150.4233
129.4771
125.6224
121.7677
93.8975
74.8859
22.6221
220 200 180 160 140 120 100 80 60 40 20 0 -20
Chemical Shift (ppm)

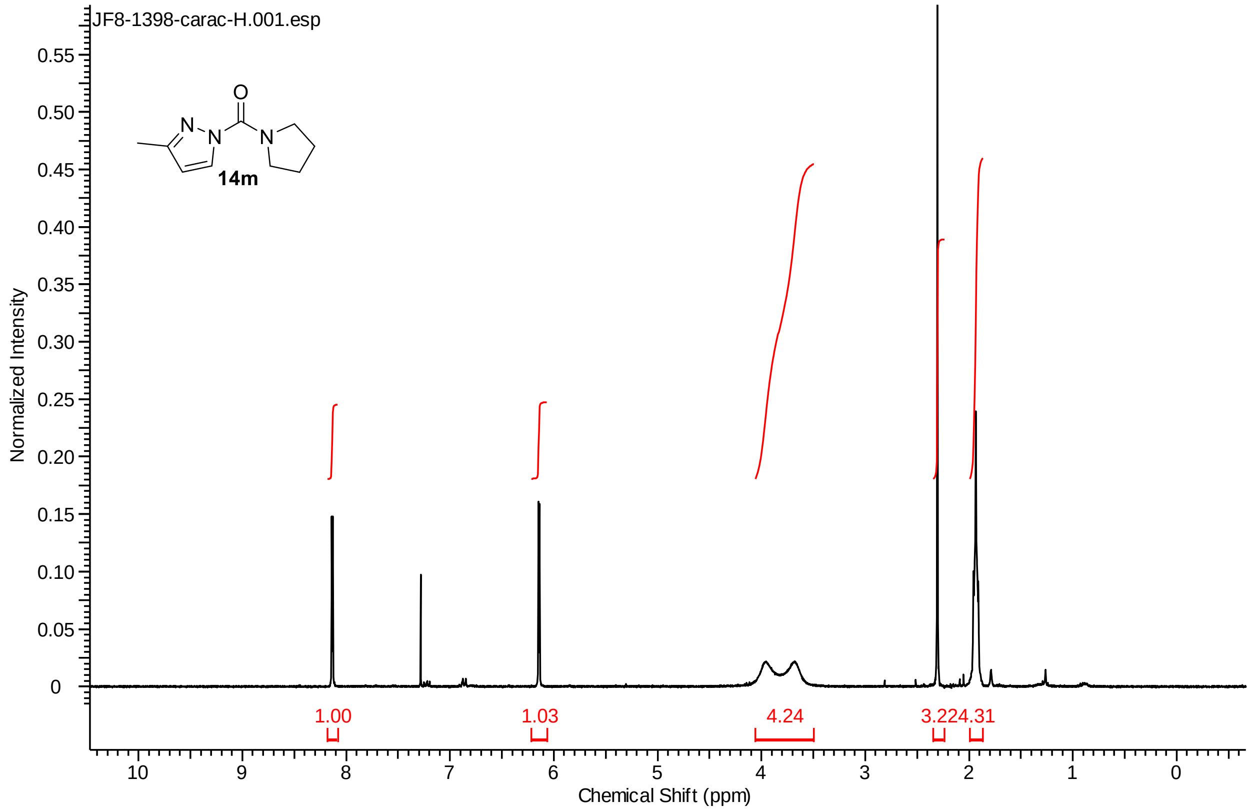
JF8-1398-carac-H.001.esp
O
N
N
N
14m
Normalized Intensity
1.00
1.03
4.24
3.22
4.31
Chemical Shift (ppm)

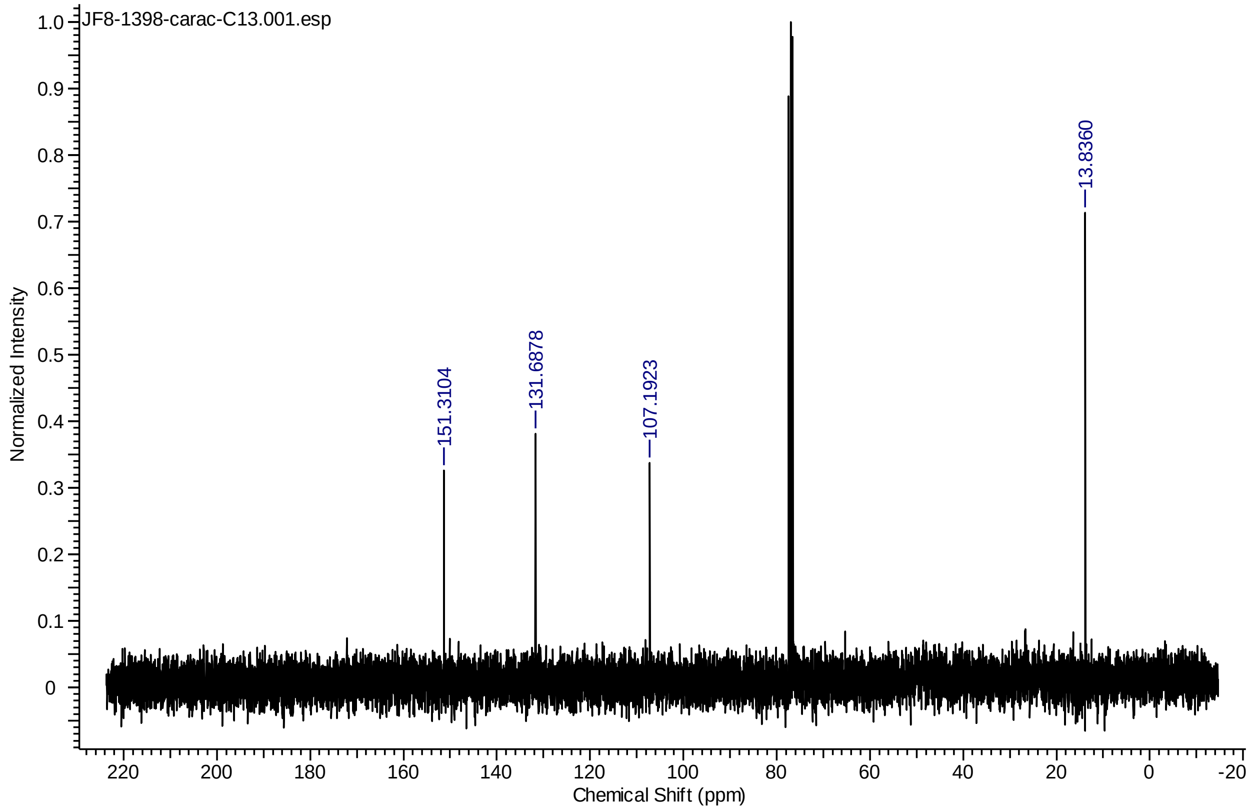
JF8-1398-carac-C13.001.esp
Normalized Intensity
151.3104
131.6878
107.1923
13.8360
Chemical Shift (ppm)

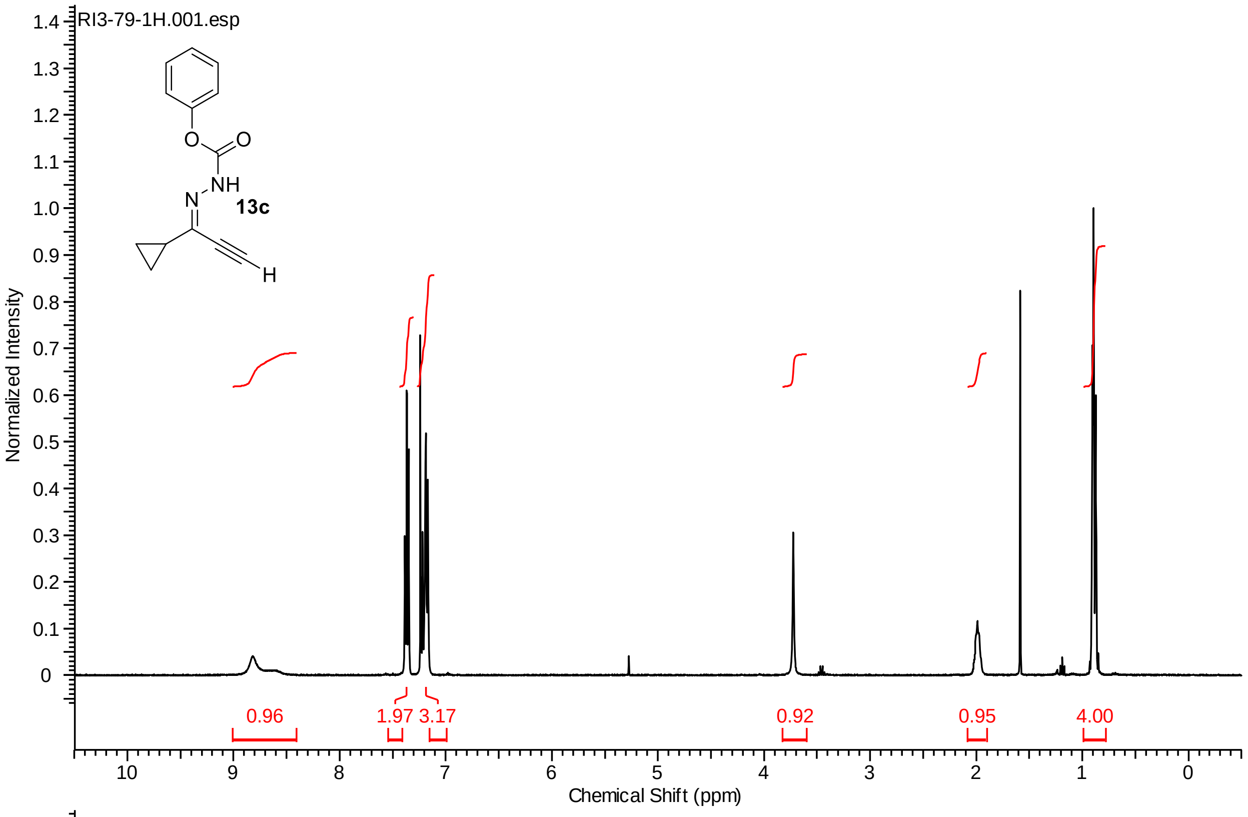
RI3-79-1H.001.esp
13c
O
O
NH
N
H
Normalized Intensity
0.96
1.97
3.17
0.92
0.95
4.00
Chemical Shift (ppm)

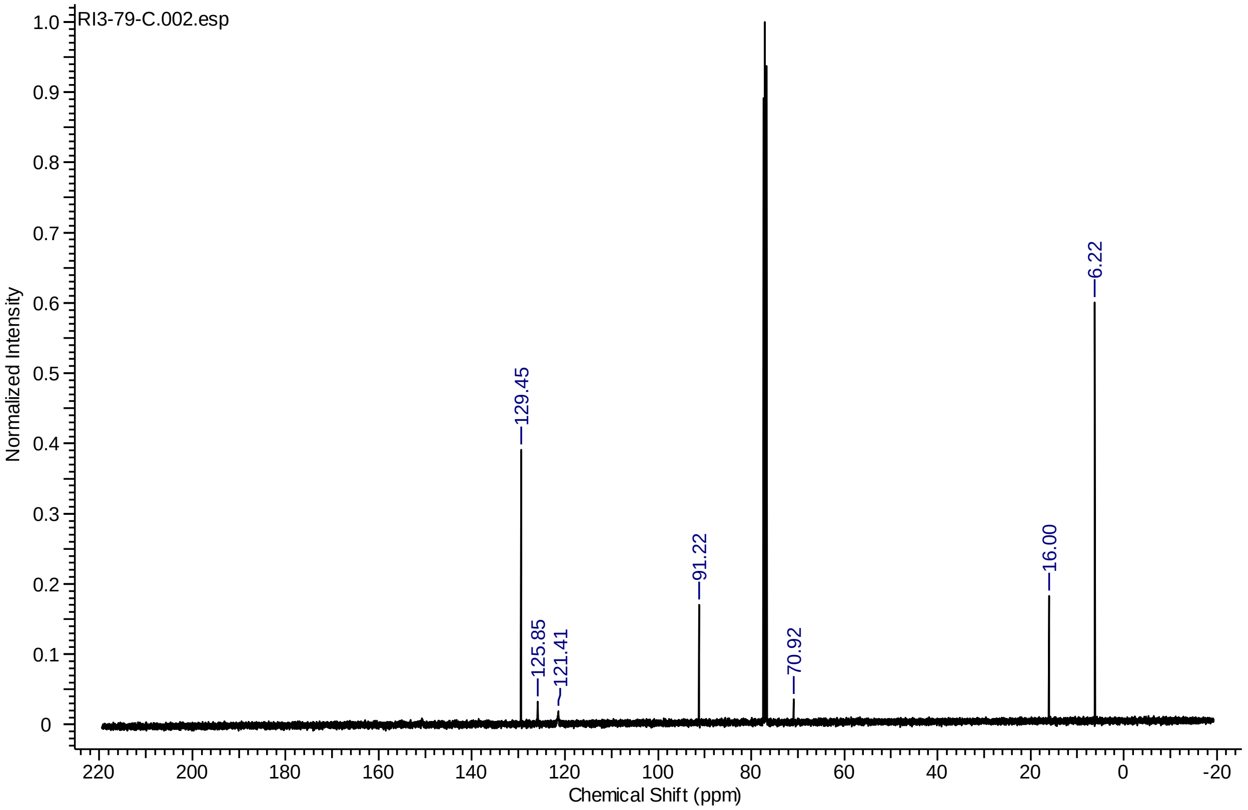
RI3-79-C.002.esp
129.45
125.85
121.41
91.22
70.92
16.00
6.22
Normalized Intensity
Chemical Shift (ppm)

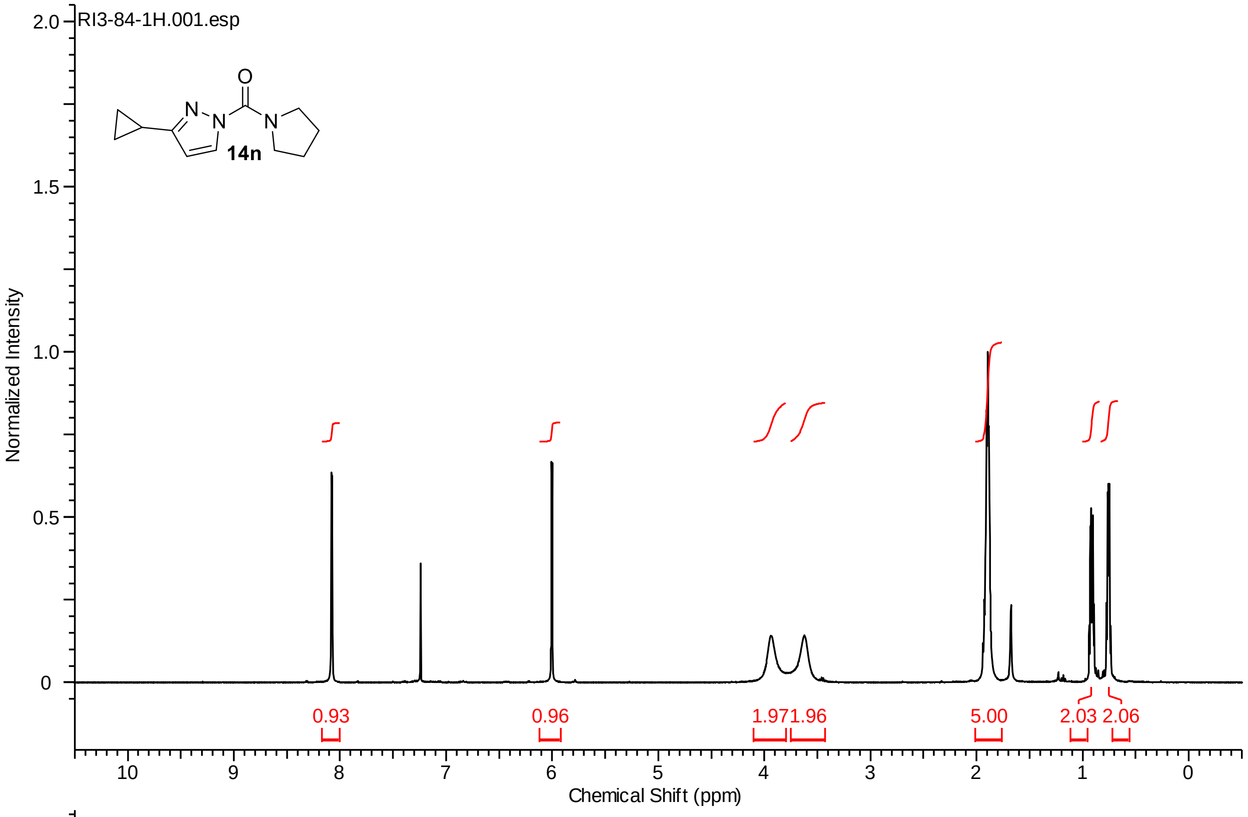
RI3-84-1H.001.esp
14n
0.93
0.96
1.97
1.96
5.00
2.03
2.06
Normalized Intensity
Chemical Shift (ppm)

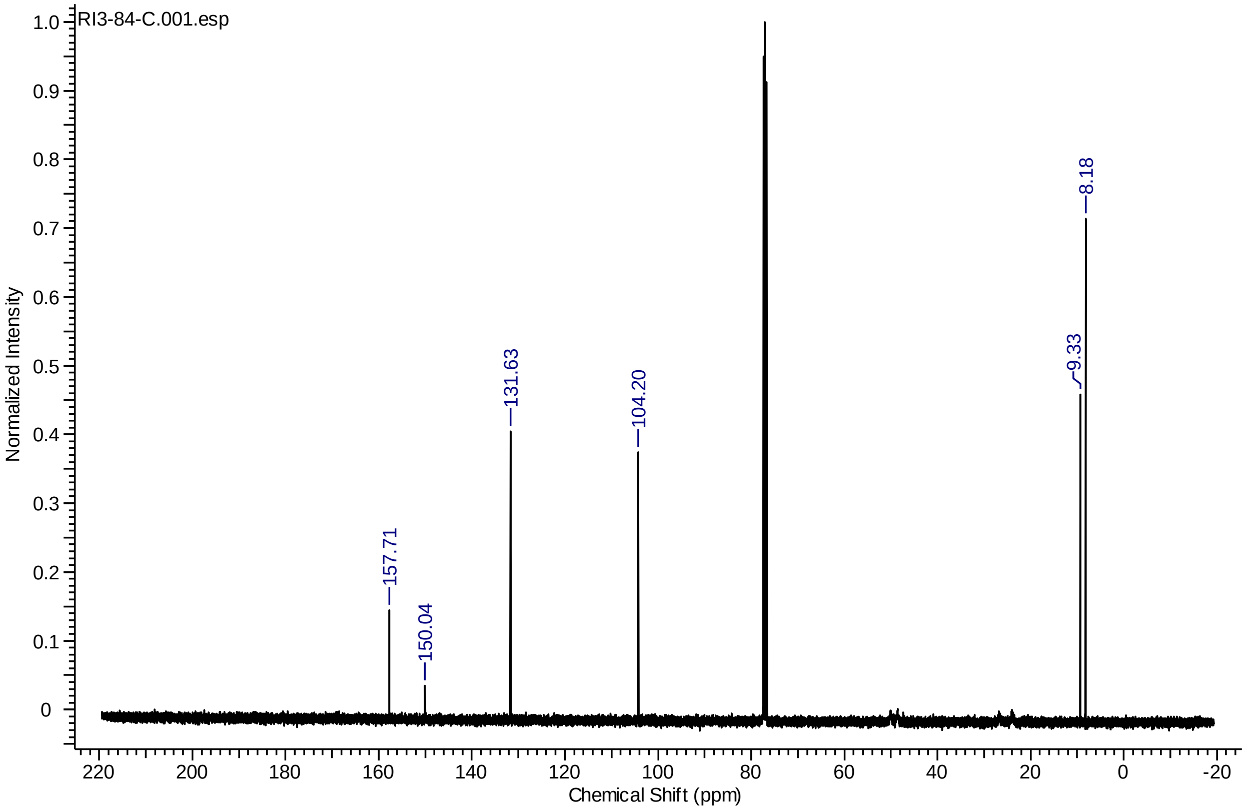
RI3-84-C.001.esp
157.71
150.04
131.63
104.20
9.33
8.18
Normalized Intensity
Chemical Shift (ppm)

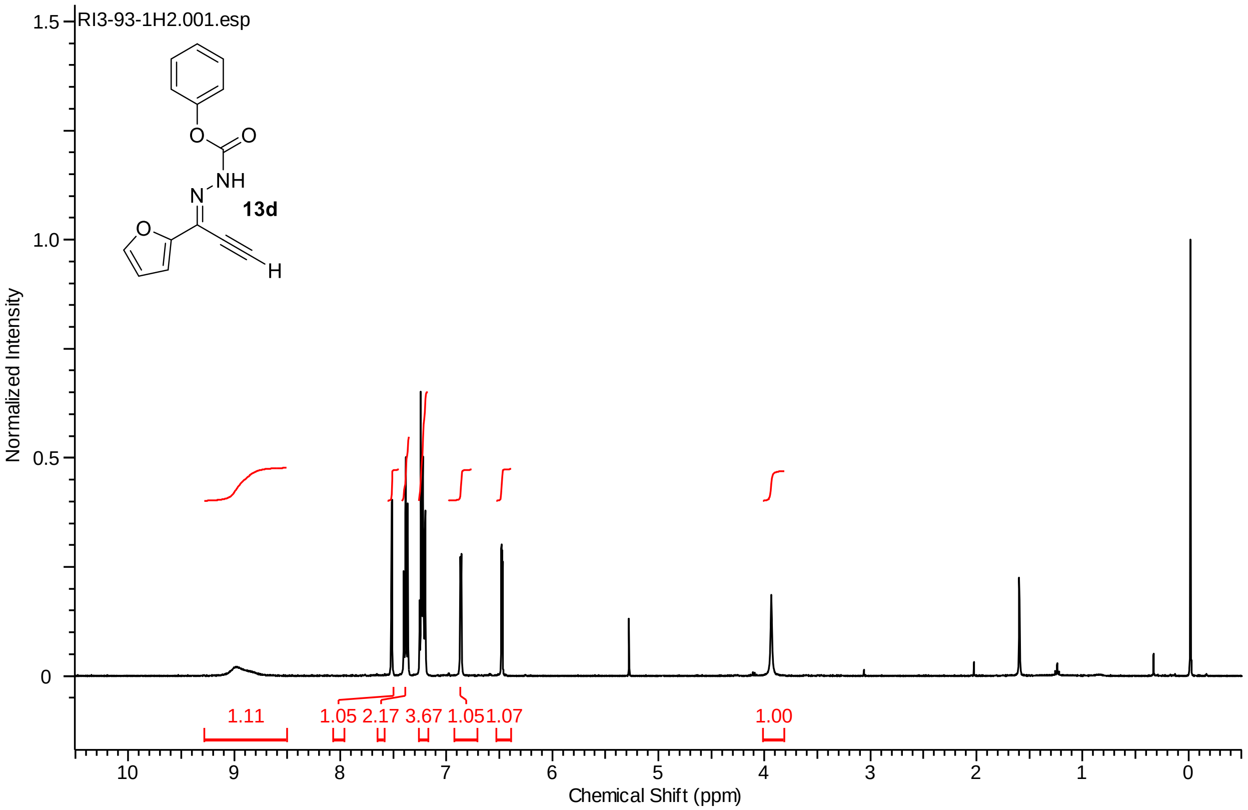
RI3-93-1H2.001.esp
13d
1.11
1.05
2.17
3.67
1.05
1.07
1.00
Normalized Intensity
Chemical Shift (ppm)

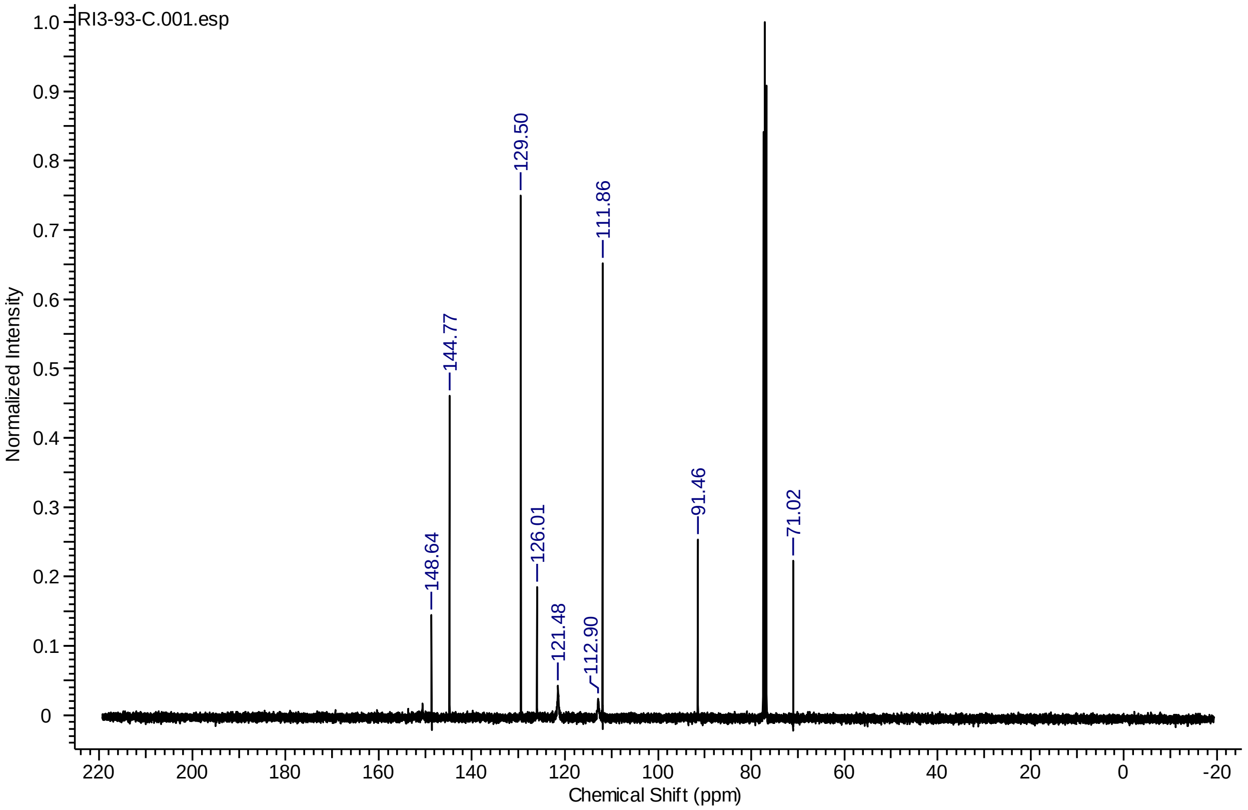
RI3-93-C.001.esp
148.64
144.77
129.50
126.01
121.48
112.90
111.86
91.46
71.02
Normalized Intensity
Chemical Shift (ppm)

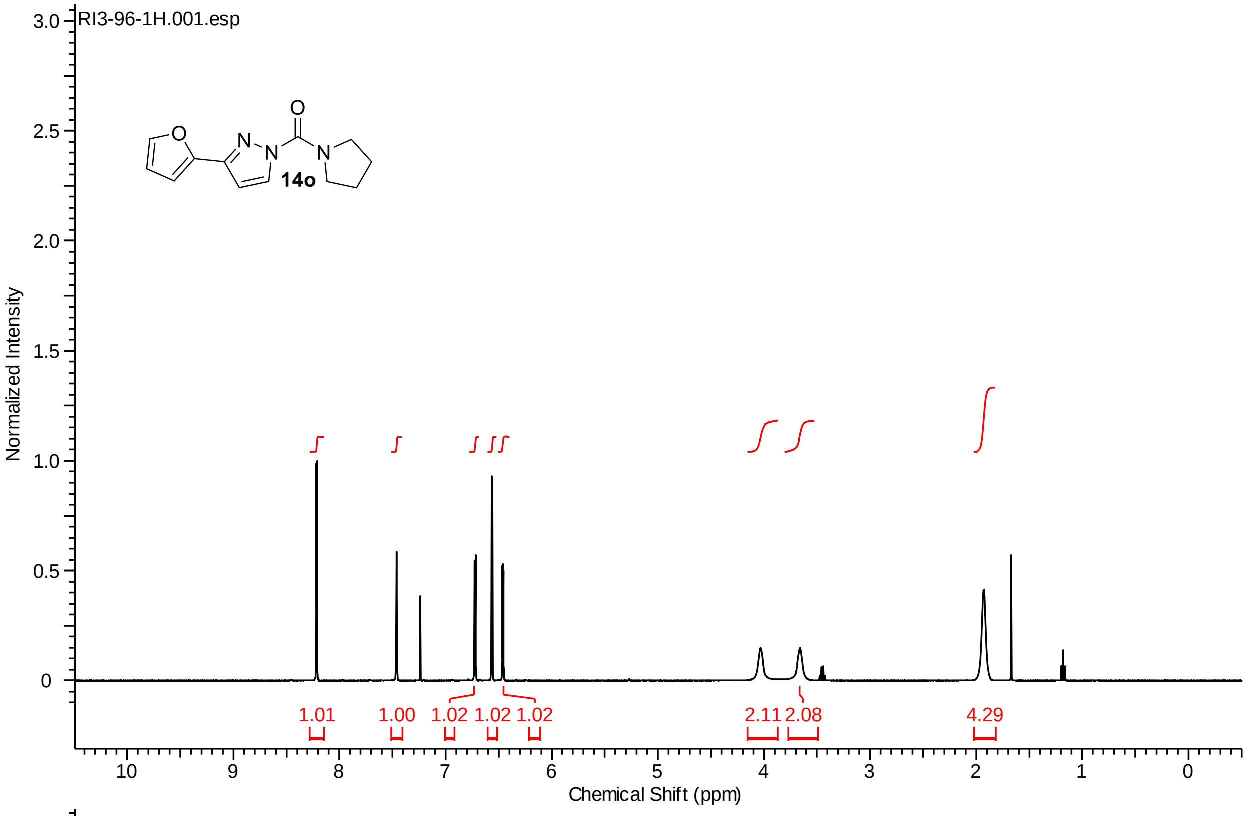
RI3-96-1H.001.esp
14o
Normalized Intensity
Chemical Shift (ppm)
1.01
1.00
1.02
1.02
1.02
2.11
2.08
4.29

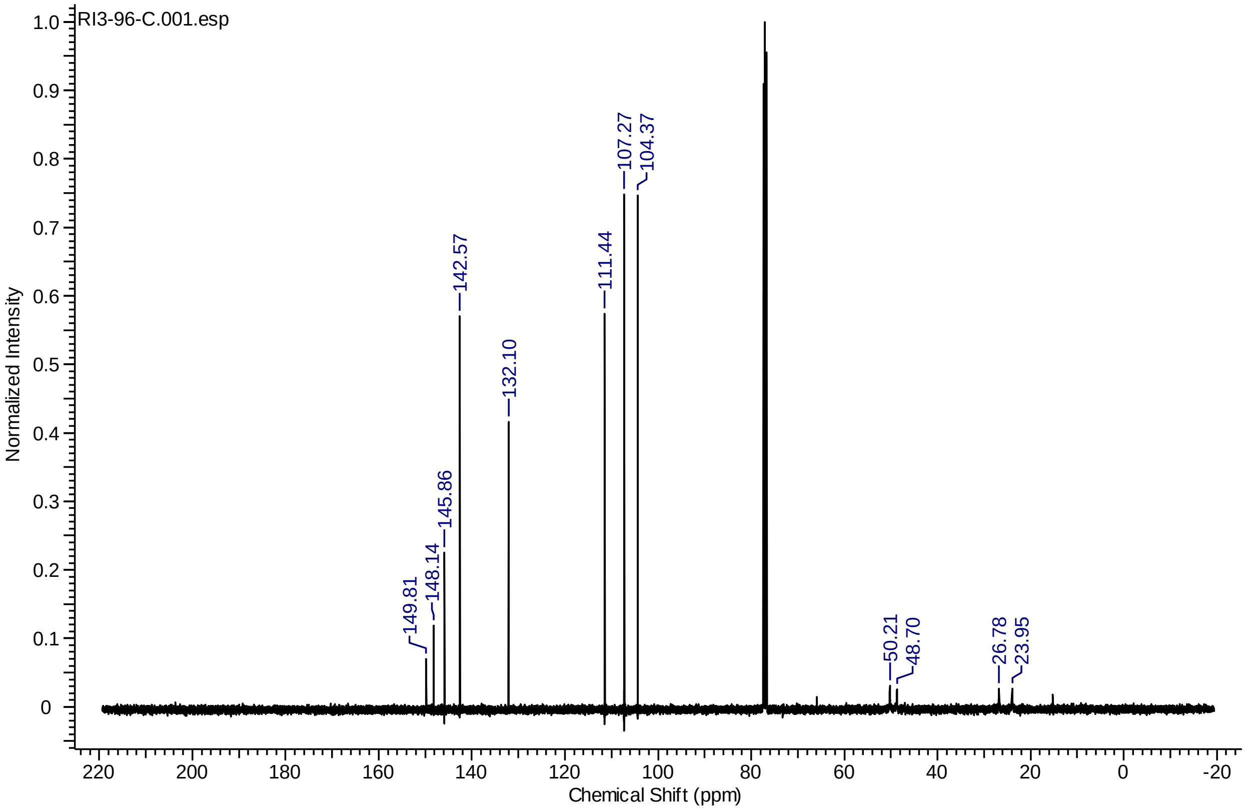
RI3-96-C.001.esp
Normalized Intensity
Chemical Shift (ppm)
149.81
148.14
145.86
142.57
132.10
111.44
107.27
104.37
50.21
48.70
26.78
23.95

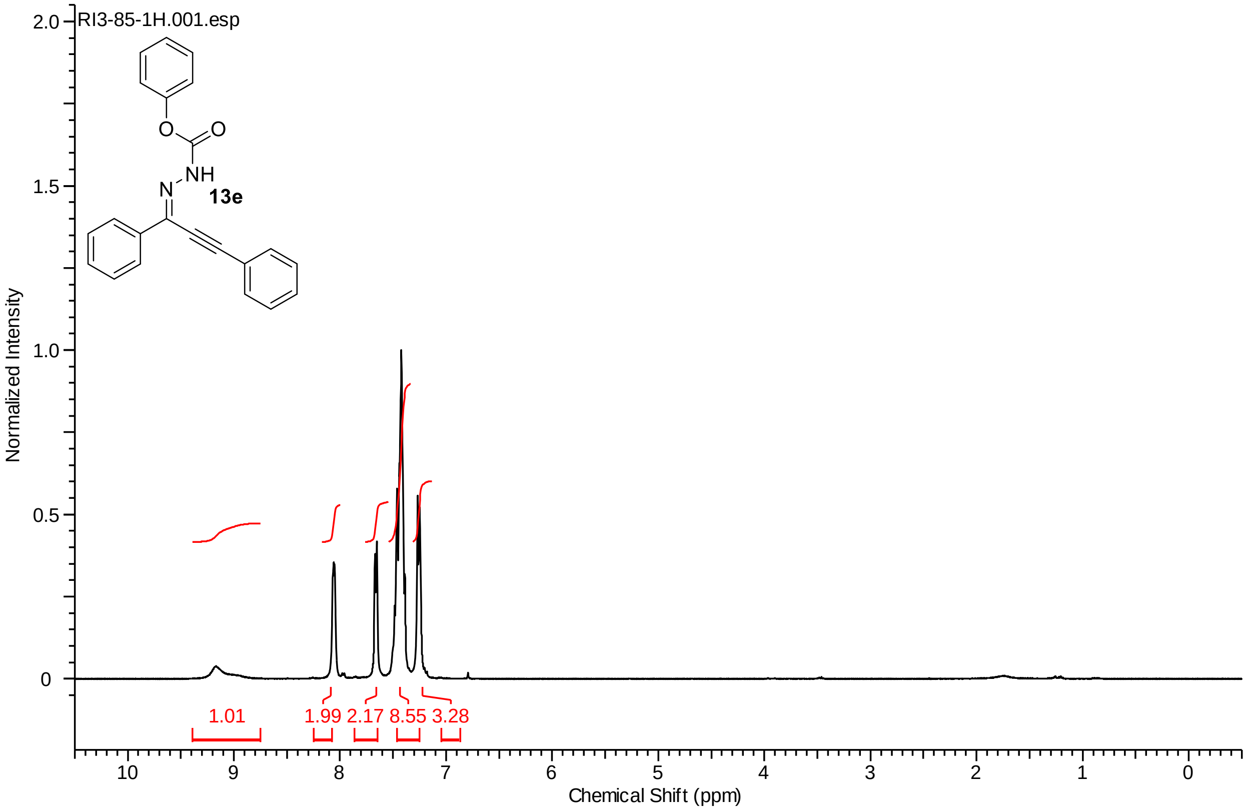
RI3-85-1H.001.esp
13e
O
O
NH
N
1.01
1.99
2.17
8.55
3.28
Normalized Intensity
Chemical Shift (ppm)

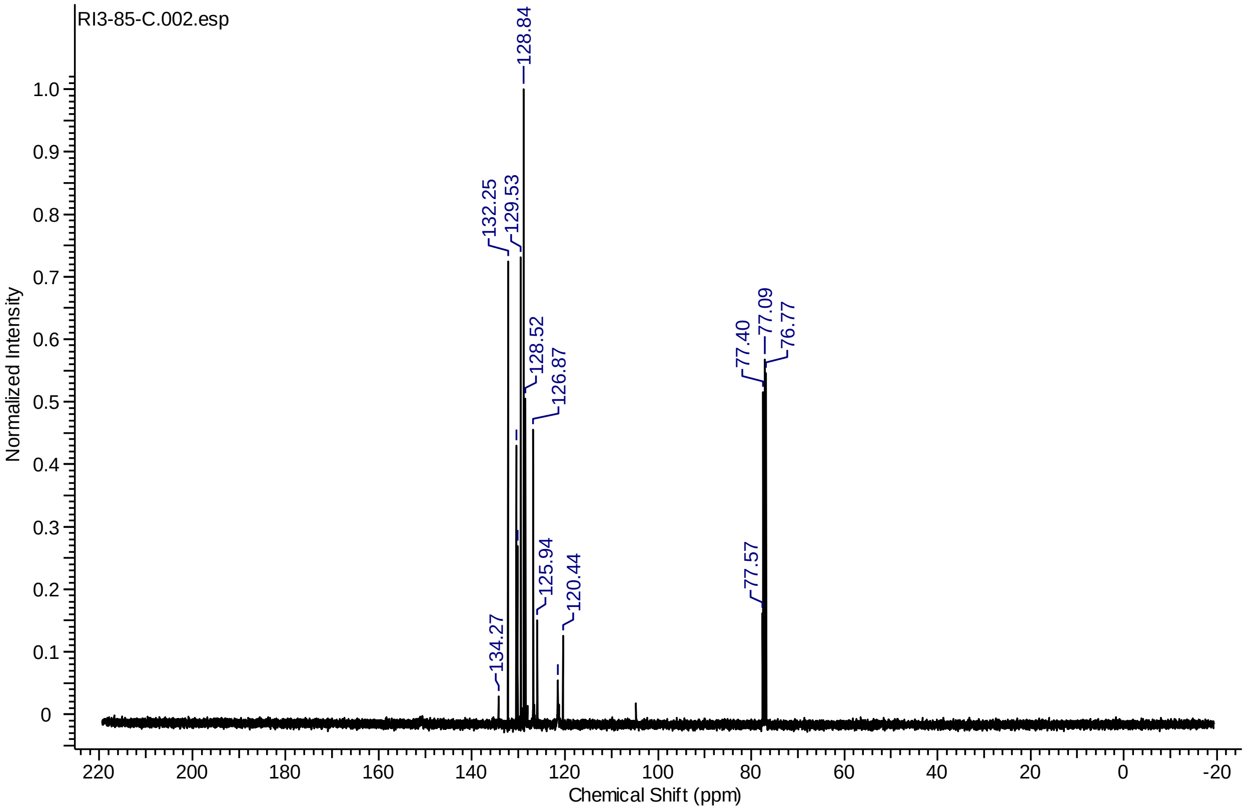
RI3-85-C.002.esp
128.84
132.25
129.53
128.52
126.87
125.94
120.44
134.27
77.40
77.09
76.77
77.57
Normalized Intensity
Chemical Shift (ppm)

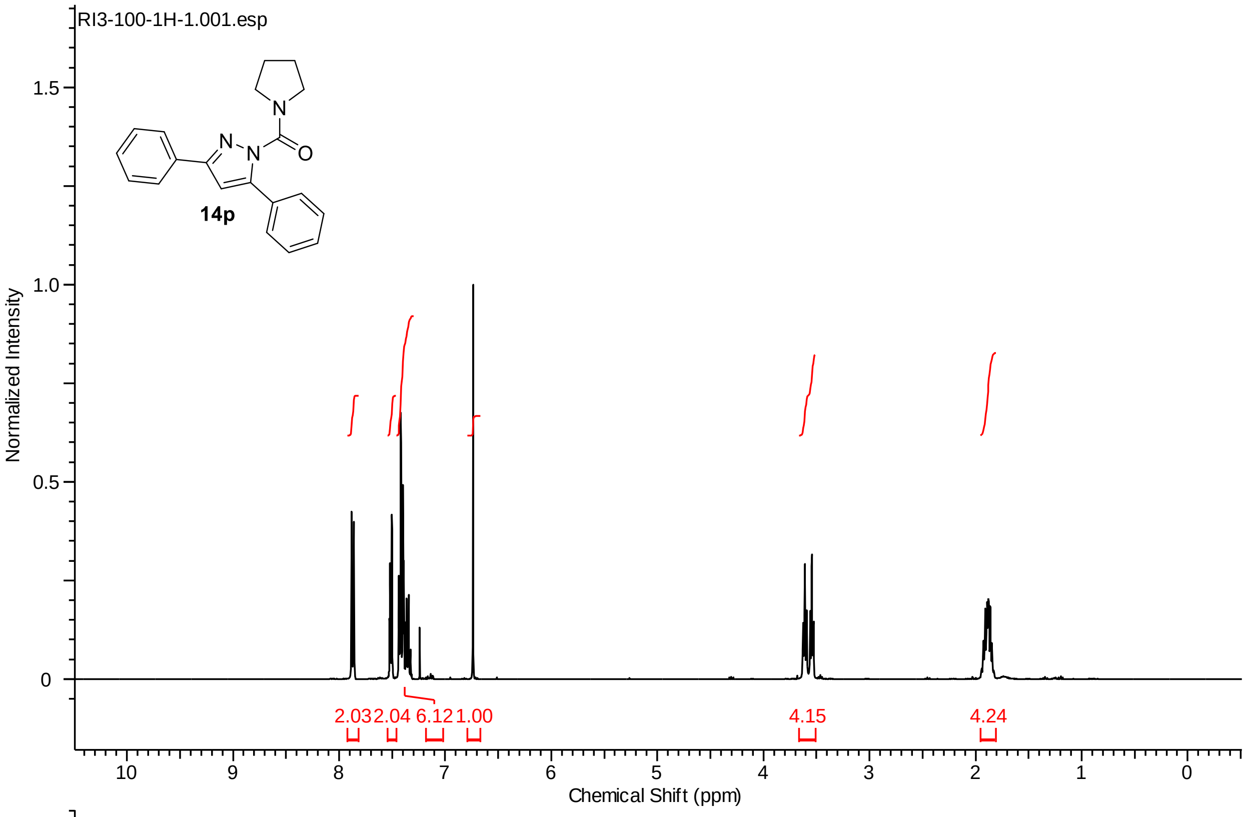
RI3-100-1H-1.001.esp
14p
N
N
N
O
Normalized Intensity
2.03
2.04
6.12
1.00
4.15
4.24
Chemical Shift (ppm)

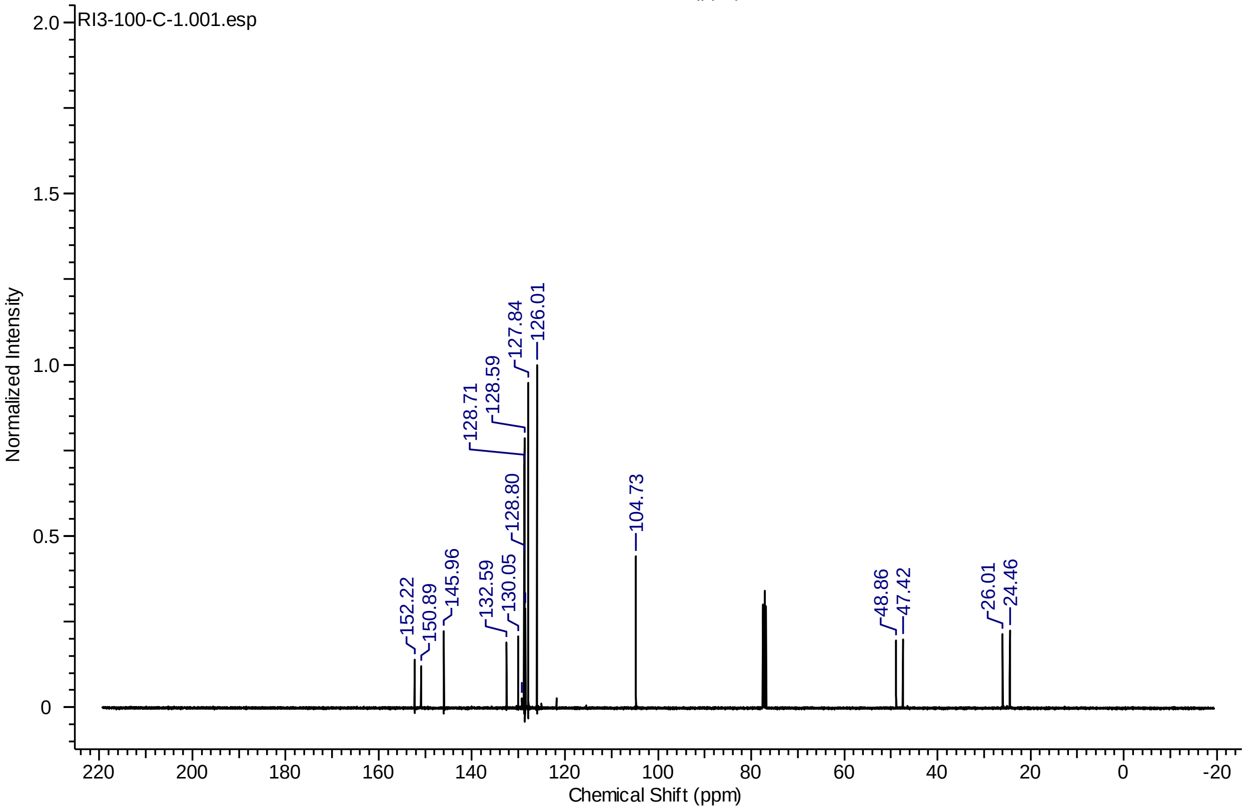
RI3-100-C-1.001.esp
Normalized Intensity
152.22
150.89
145.96
132.59
130.05
128.80
128.71
128.59
127.84
126.01
104.73
48.86
47.42
26.01
24.46
Chemical Shift (ppm)

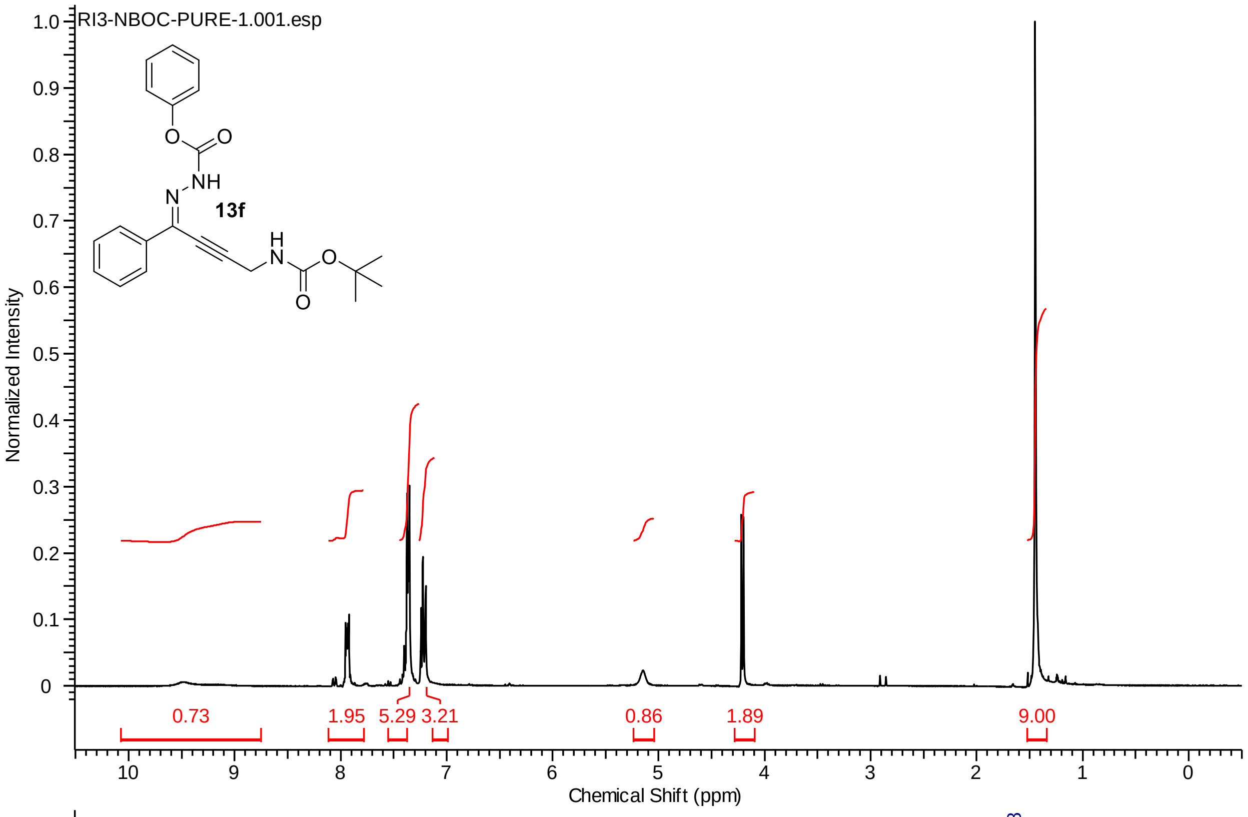

RI3-NBOC-PURE-1.001.esp
13f
Normalized Intensity
0.73
1.95
5.29
3.21
0.86
1.89
9.00
Chemical Shift (ppm)

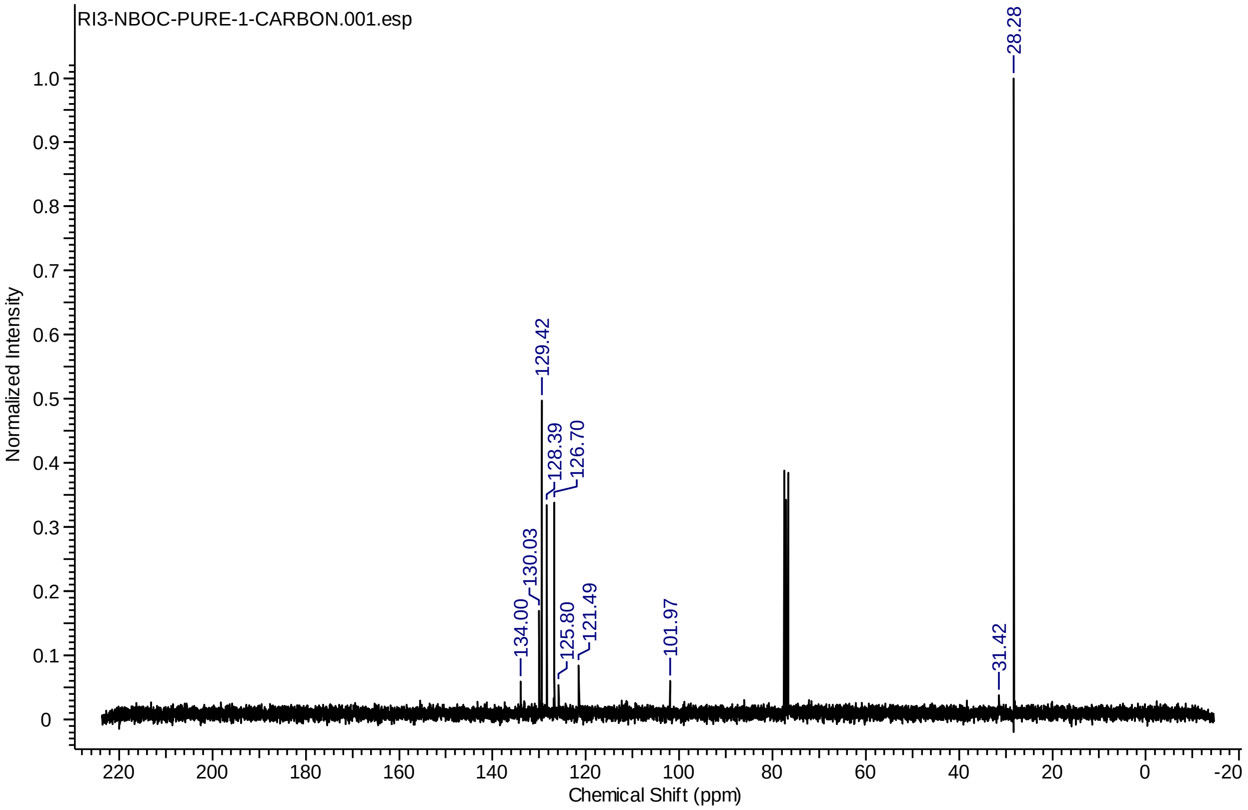

RI3-NBOC-PURE-1-CARBON.001.esp
Normalized Intensity
134.00
130.03
129.42
128.39
126.70
125.80
121.49
101.97
31.42
28.28
Chemical Shift (ppm)

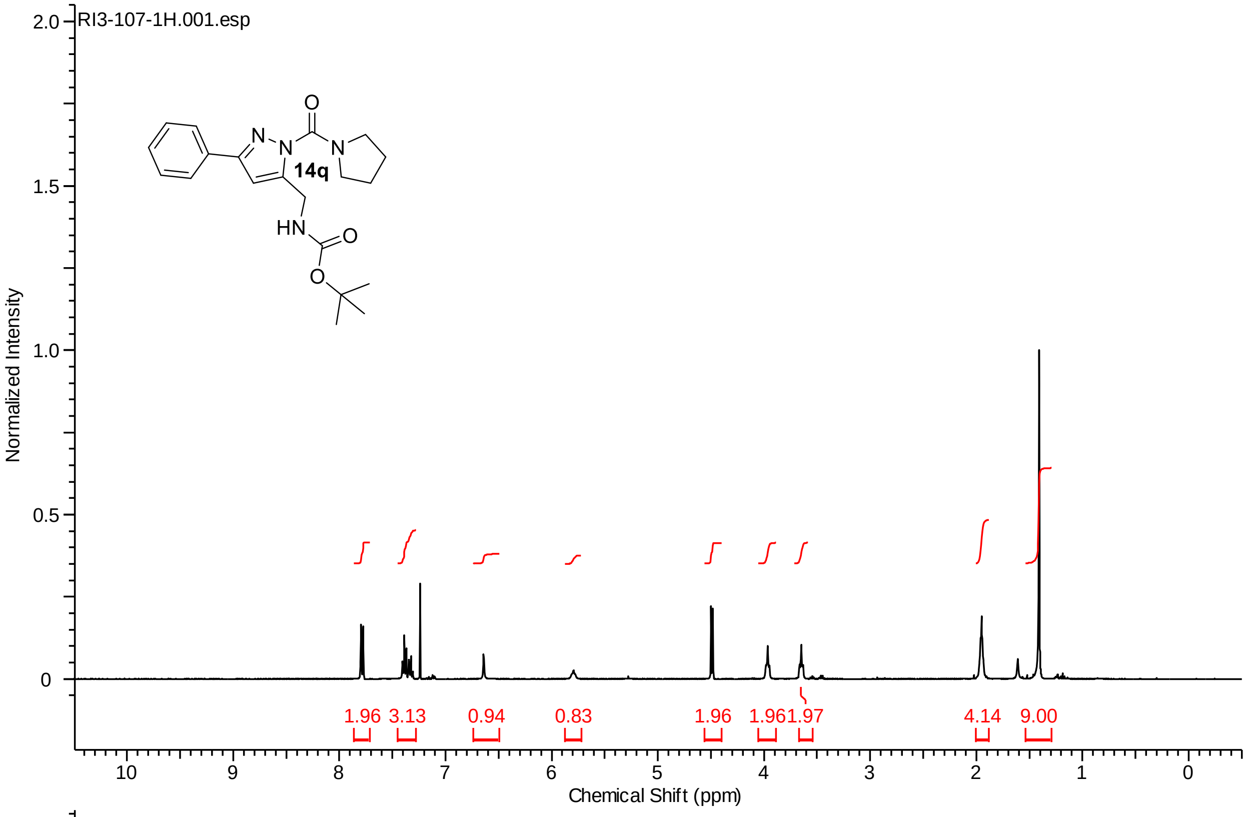
RI3-107-1H.001.esp
14q
N
N
N
O
HN
O
O
Normalized Intensity
1.96
3.13
0.94
0.83
1.96
1.96
1.97
4.14
9.00
Chemical Shift (ppm)

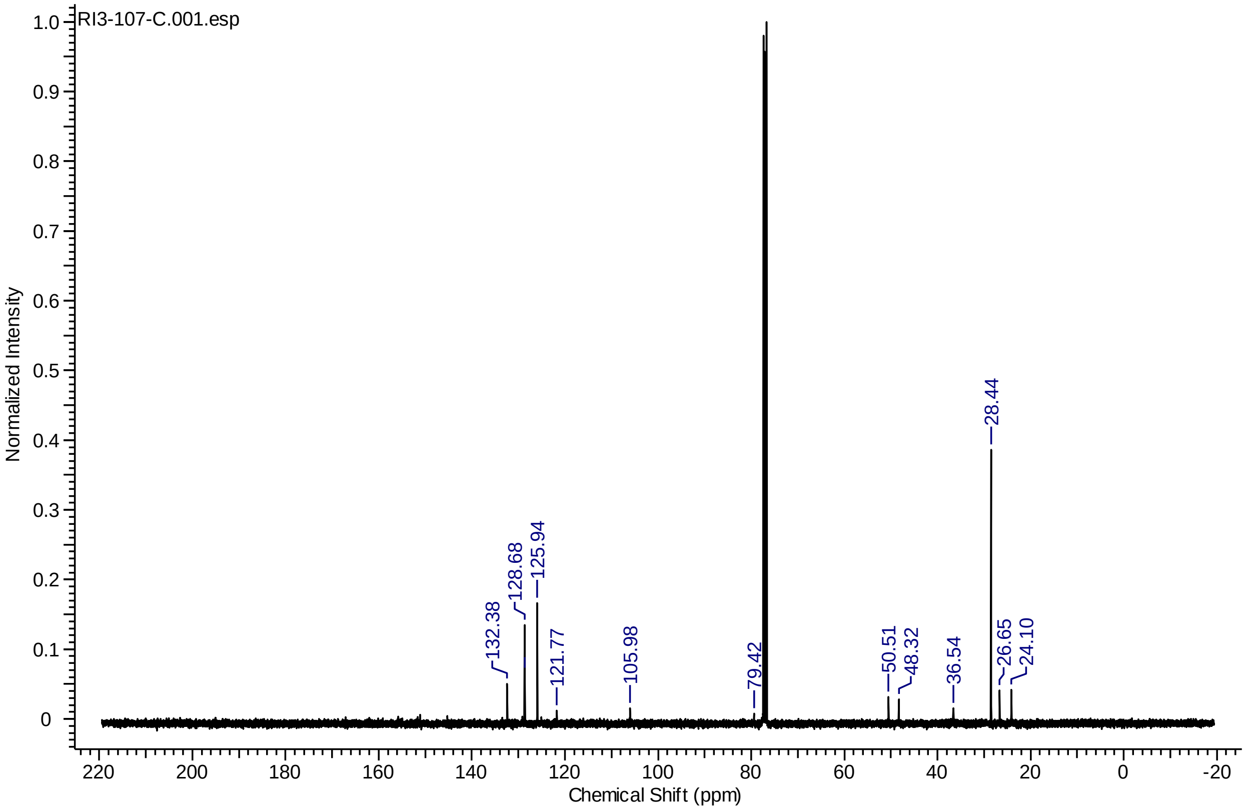
RI3-107-C.001.esp
Normalized Intensity
132.38
128.68
125.94
121.77
105.98
79.42
50.51
48.32
36.54
28.44
26.65
24.10
Chemical Shift (ppm)

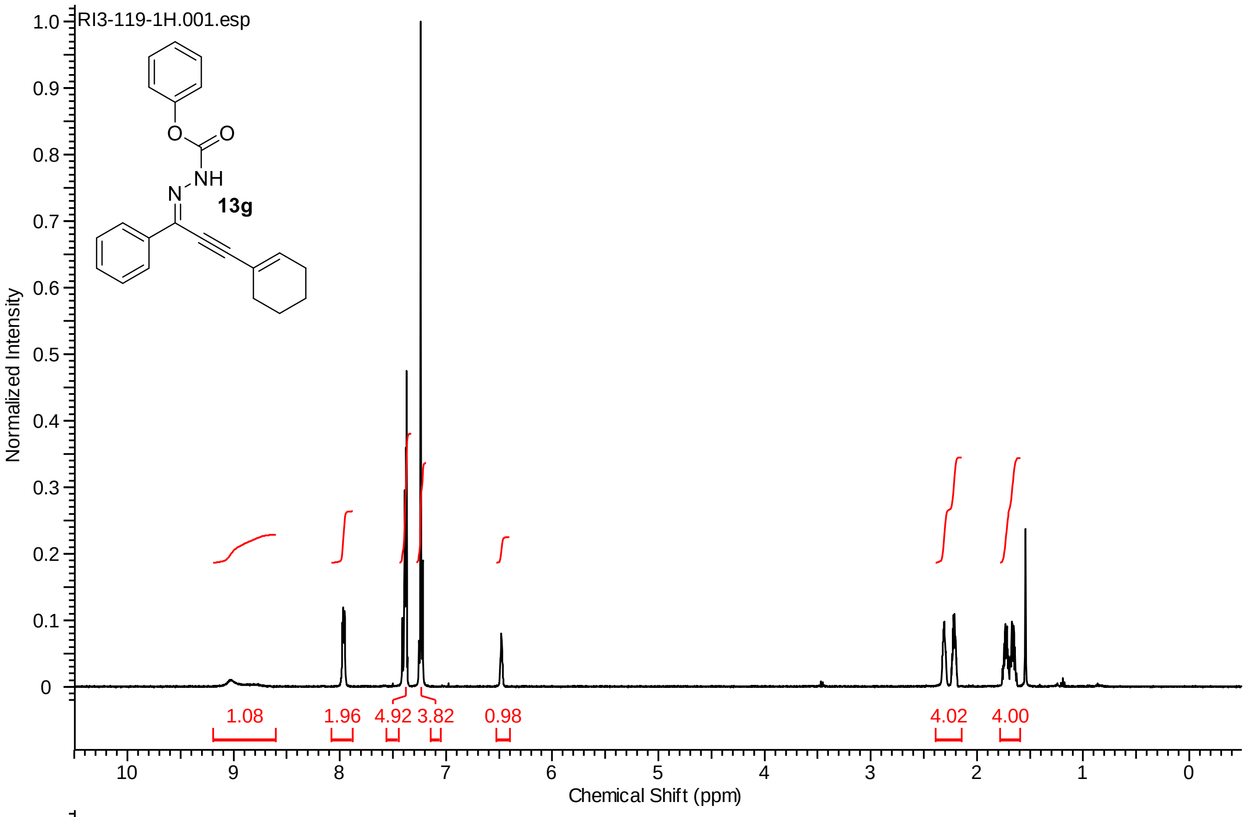
RI3-119-1H.001.esp
13g
Normalized Intensity
1.08
1.96
4.92
3.82
0.98
4.02
4.00
Chemical Shift (ppm)

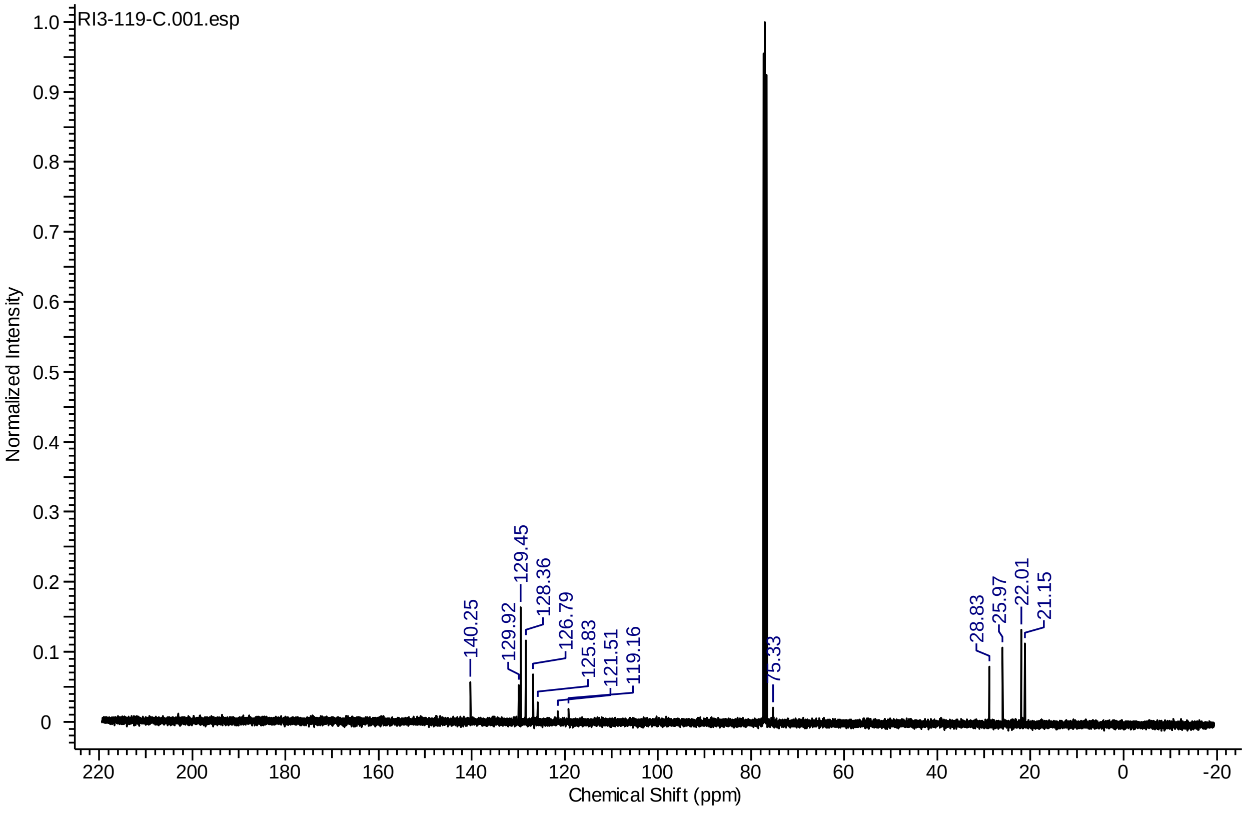
RI3-119-C.001.esp
Normalized Intensity
140.25
129.92
129.45
128.36
126.79
125.83
121.51
119.16
75.33
28.83
25.97
22.01
21.15
Chemical Shift (ppm)

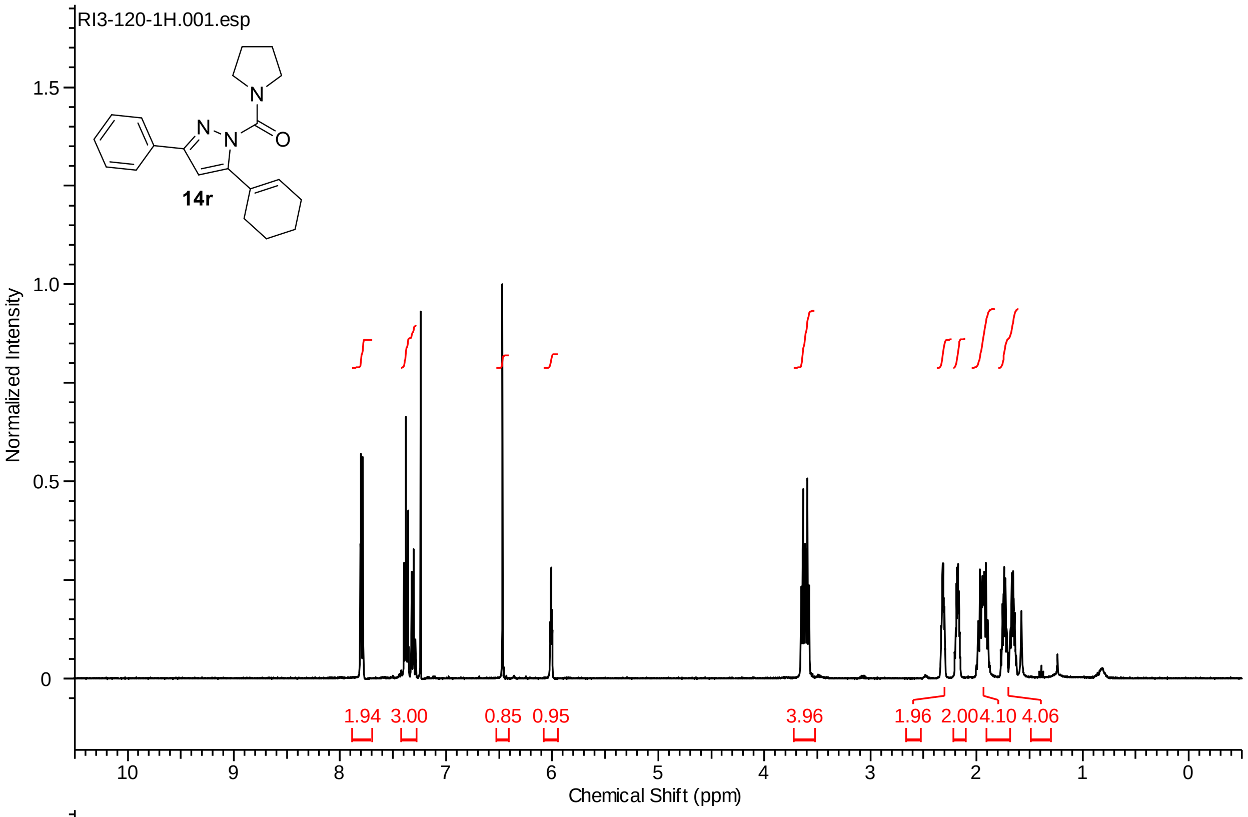
RI3-120-1H.001.esp
14r
N
N
N
O
1.94
3.00
0.85
0.95
3.96
1.96
2.00
4.10
4.06
Normalized Intensity
Chemical Shift (ppm)

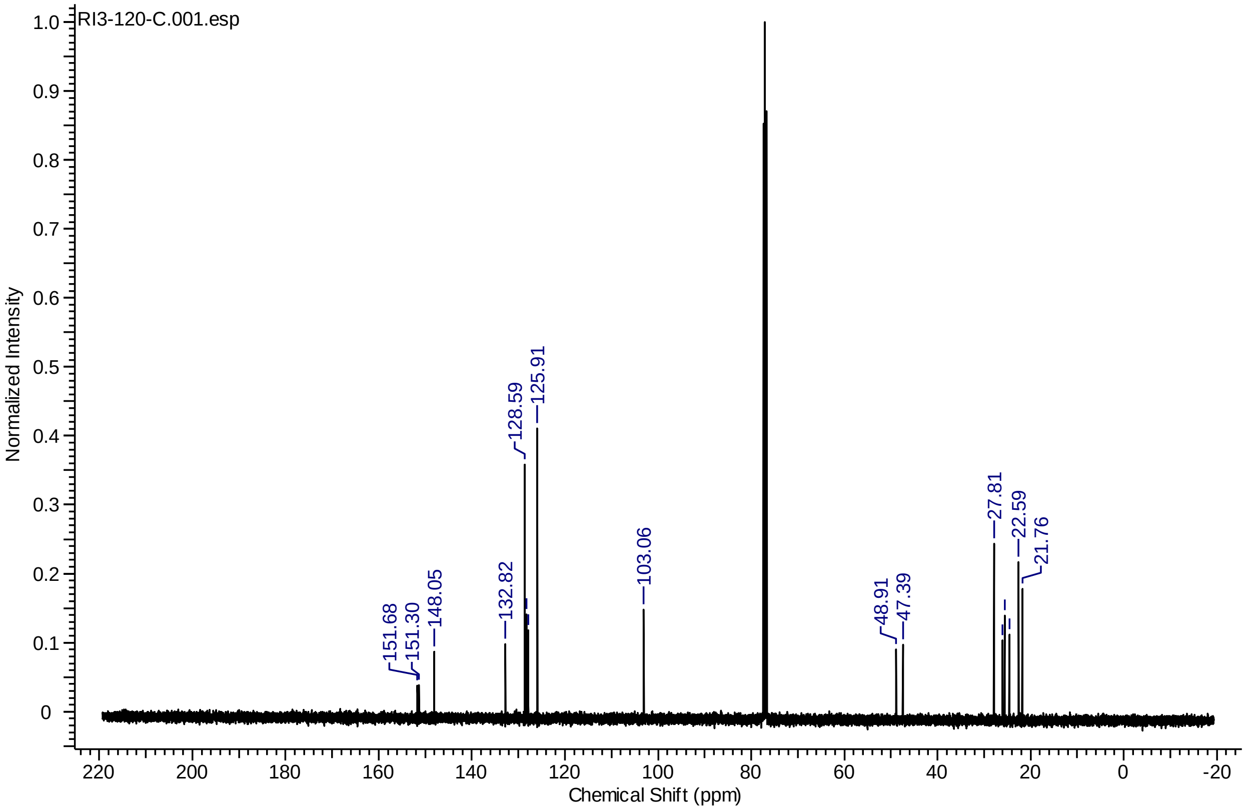
RI3-120-C.001.esp
151.68
151.30
148.05
132.82
128.59
125.91
103.06
48.91
47.39
27.81
22.59
21.76
Normalized Intensity
Chemical Shift (ppm)

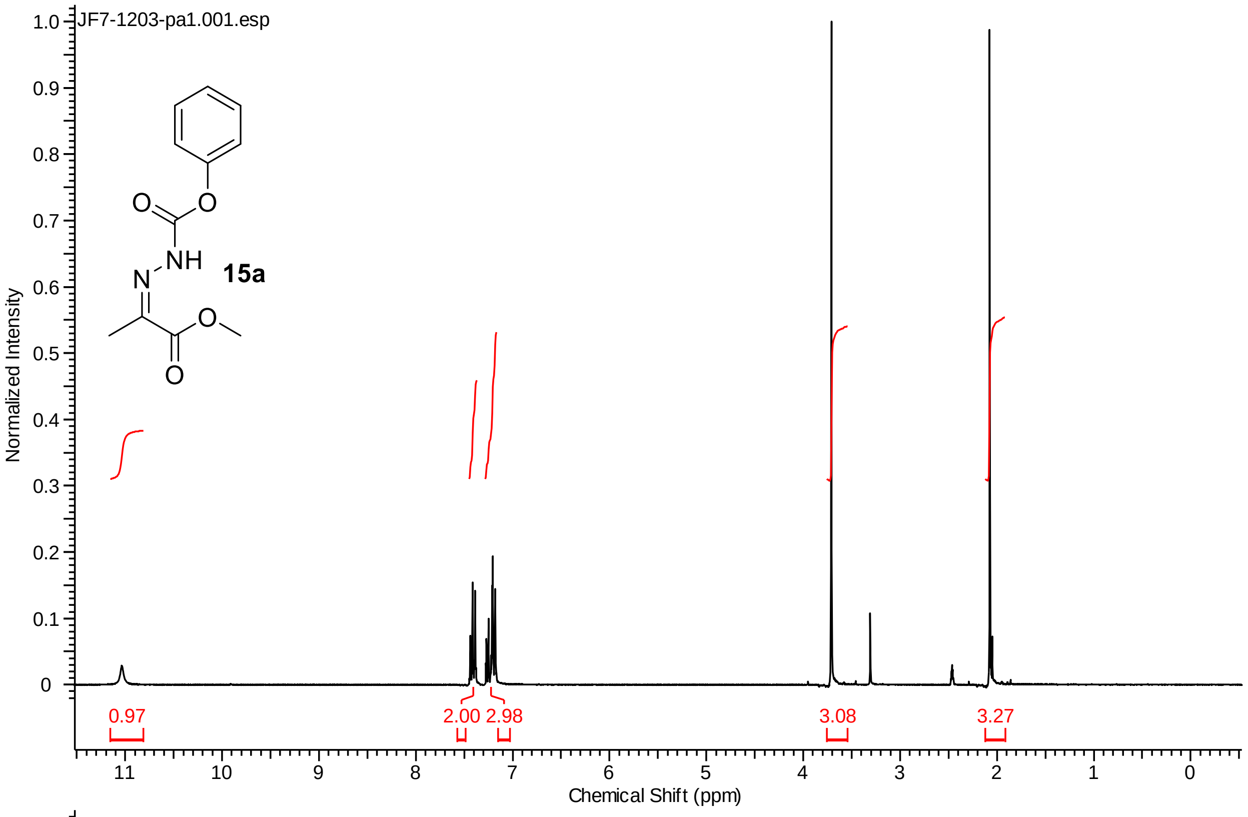
JF7-1203-pa1.001.esp
15a
Normalized Intensity
Chemical Shift (ppm)
0.97
2.00
2.98
3.08
3.27

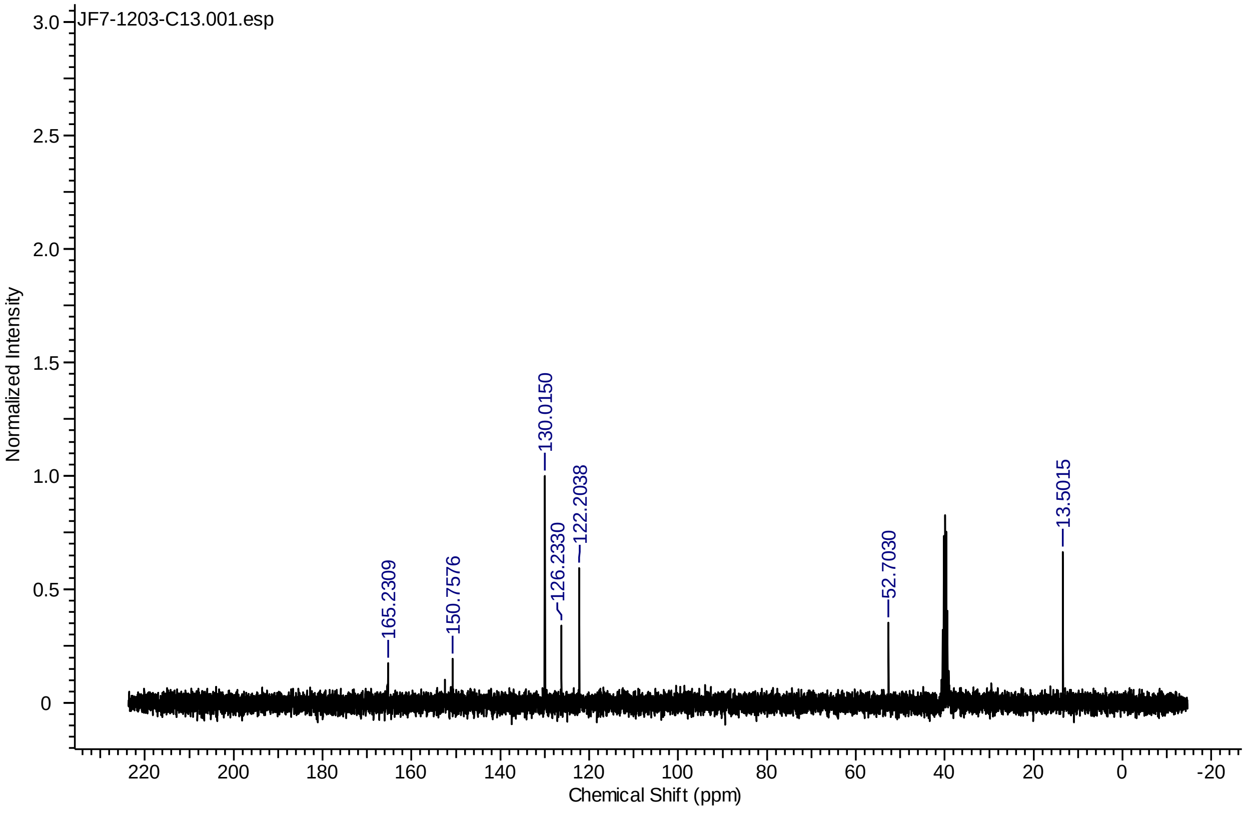
JF7-1203-C13.001.esp
Normalized Intensity
Chemical Shift (ppm)
165.2309
150.7576
130.0150
126.2330
122.2038
52.7030
13.5015

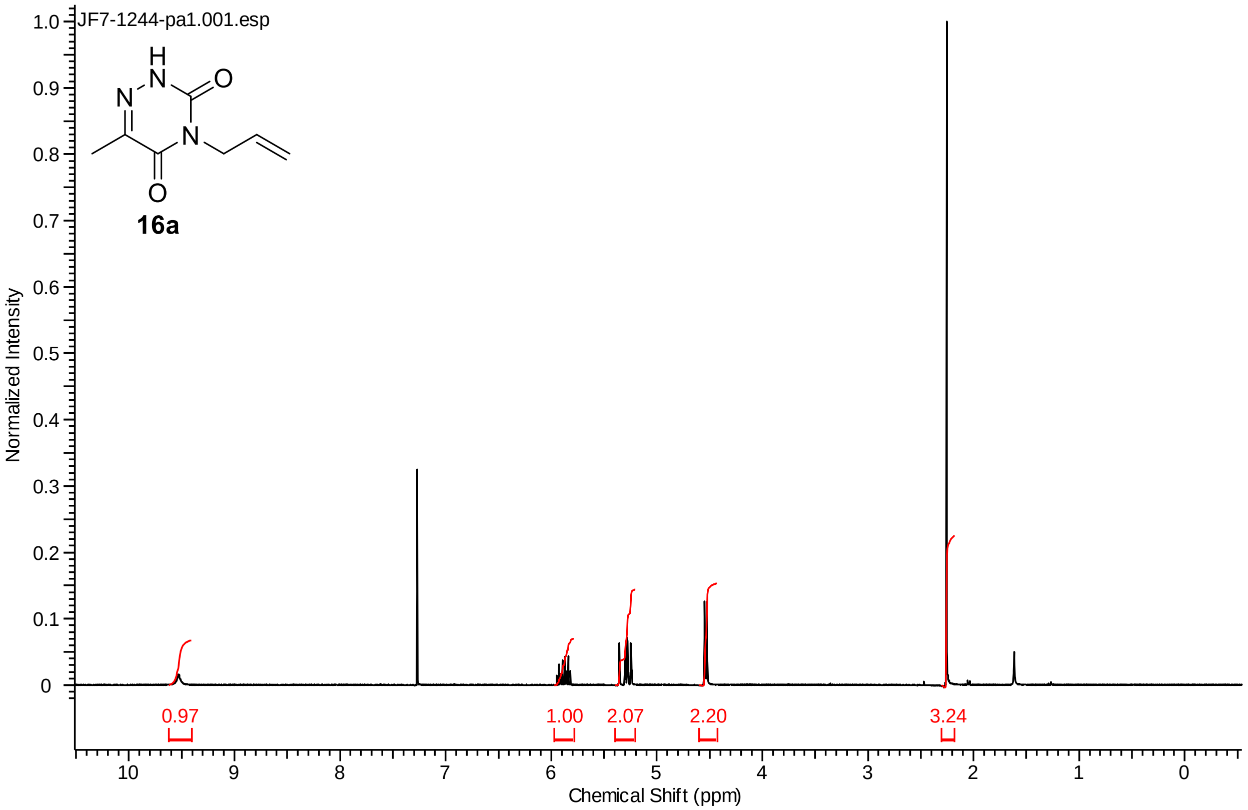
JF7-1244-pa1.001.esp
16a
Normalized Intensity
0.97
1.00
2.07
2.20
3.24
Chemical Shift (ppm)

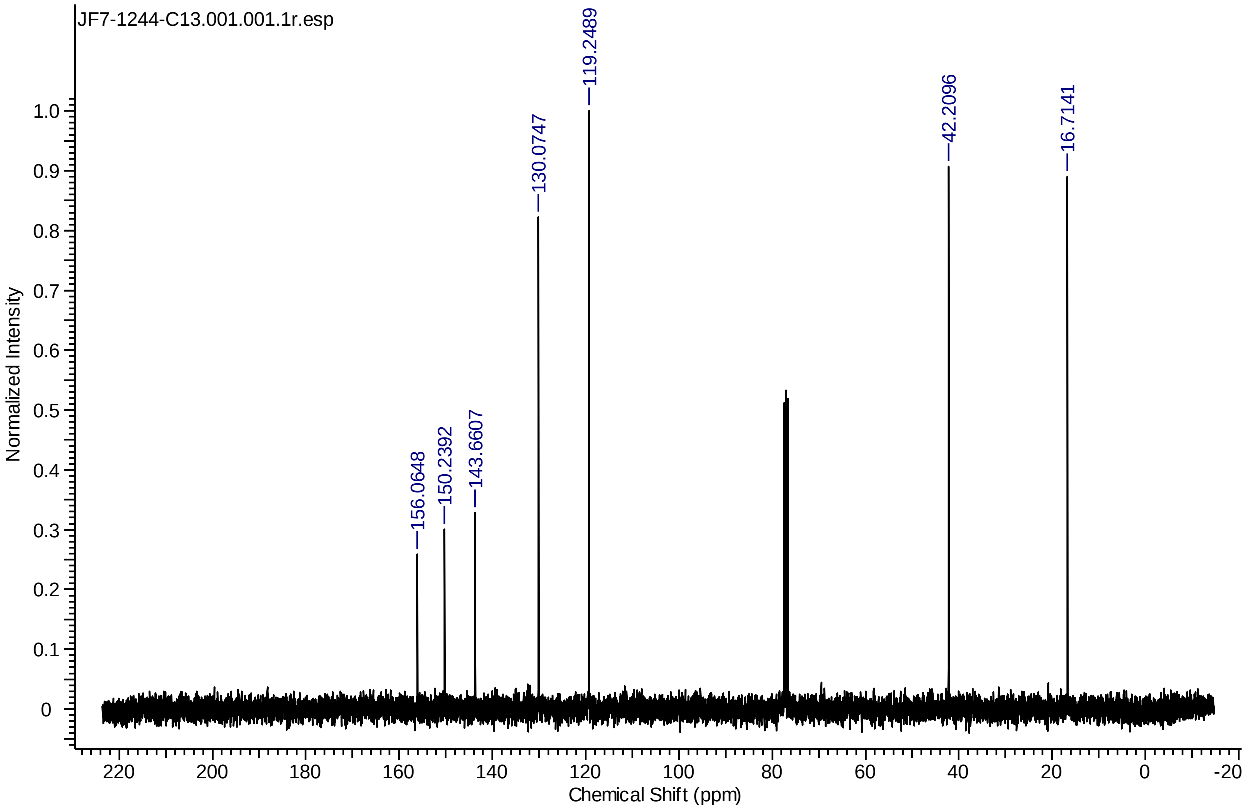
JF7-1244-C13.001.001.1r.esp
Normalized Intensity
156.0648
150.2392
143.6607
130.0747
119.2489
42.2096
16.7141
Chemical Shift (ppm)

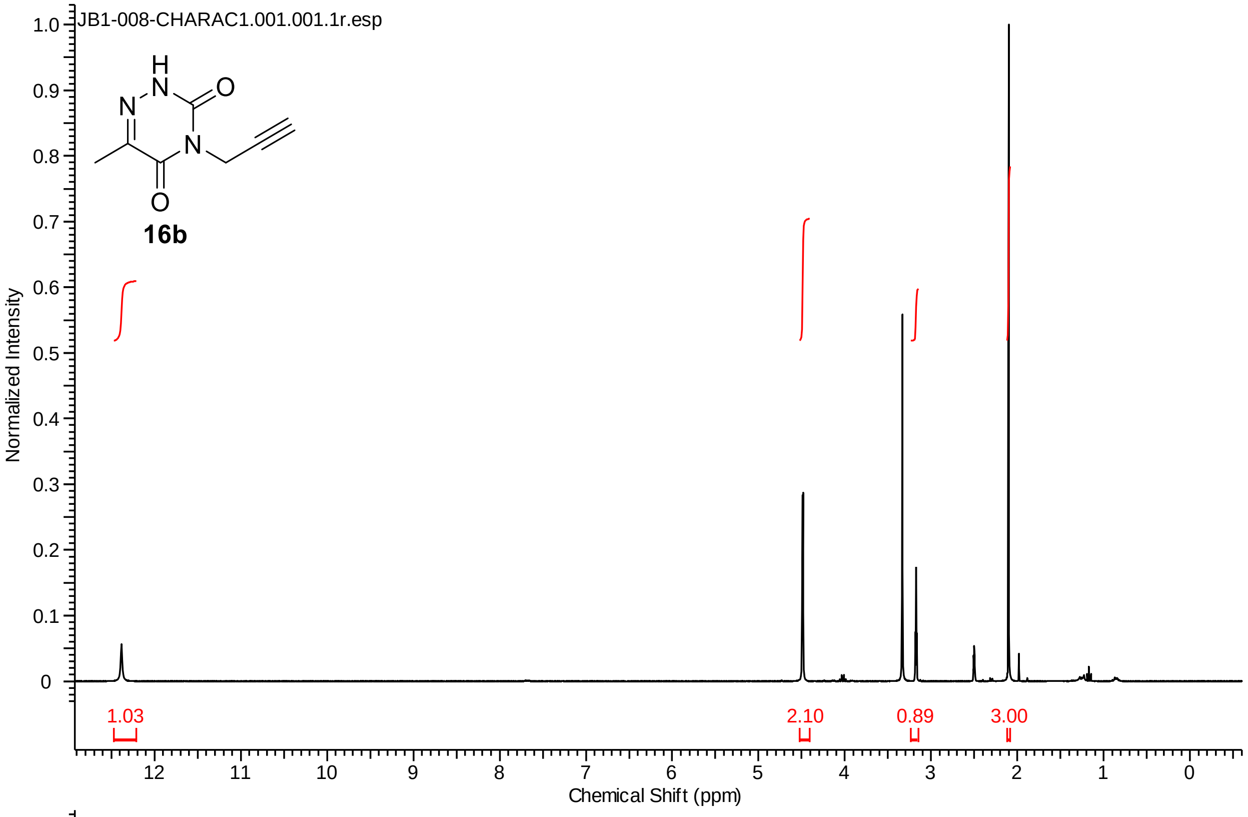
JB1-008-CHARAC1.001.001.1r.esp
16b
Normalized Intensity
Chemical Shift (ppm)
1.03
2.10
0.89
3.00

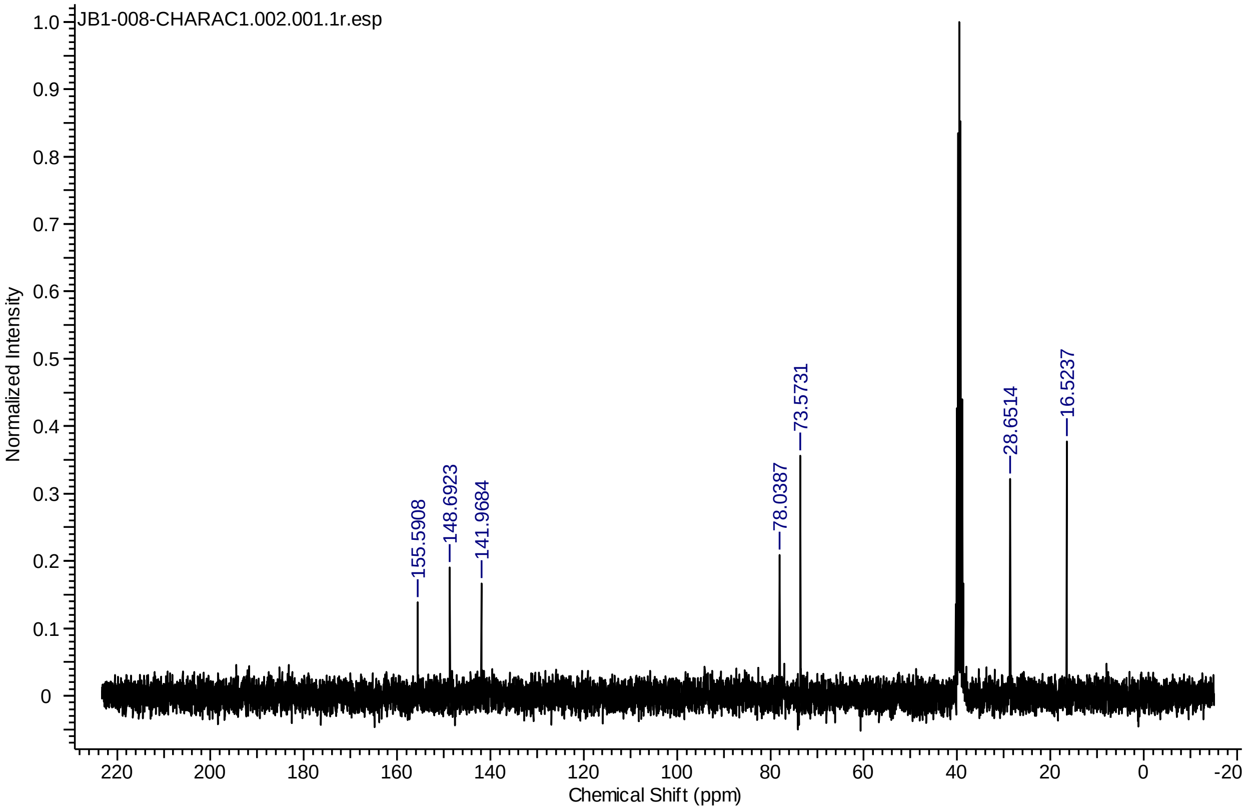
JB1-008-CHARAC1.002.001.1r.esp
Normalized Intensity
Chemical Shift (ppm)
155.5908
148.6923
141.9684
78.0387
73.5731
28.6514
16.5237

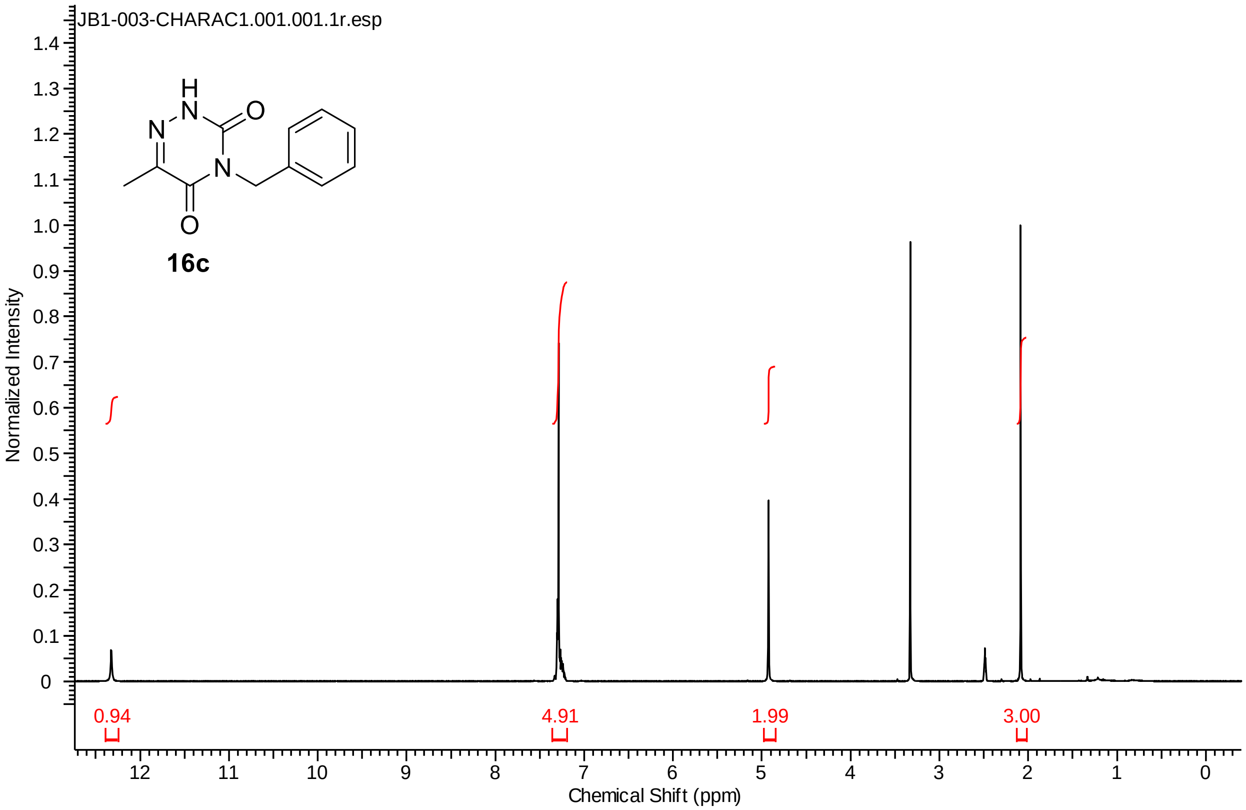
JB1-003-CHARAC1.001.001.1r.esp
16c
Normalized Intensity
0.94
4.91
1.99
3.00
Chemical Shift (ppm)

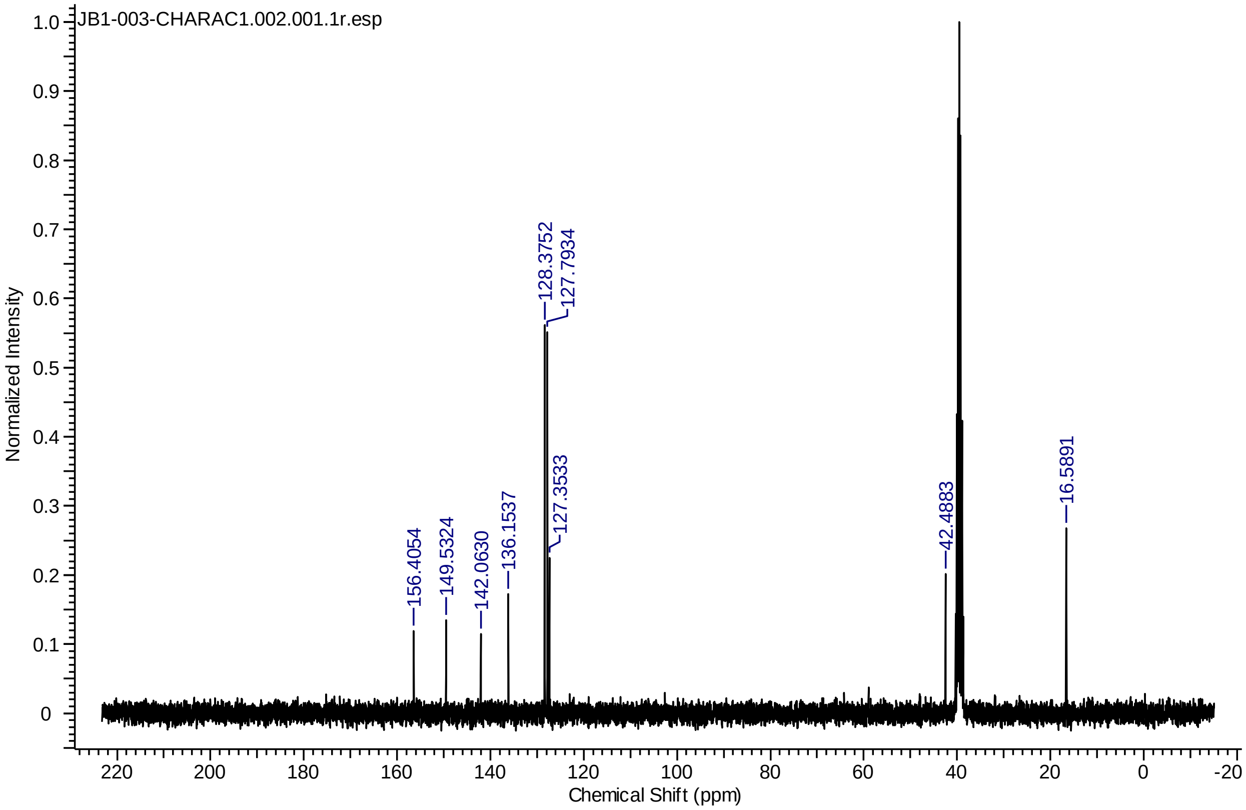
JB1-003-CHARAC1.002.001.1r.esp
Normalized Intensity
156.4054
149.5324
142.0630
136.1537
128.3752
127.7934
127.3533
42.4883
16.5891
Chemical Shift (ppm)

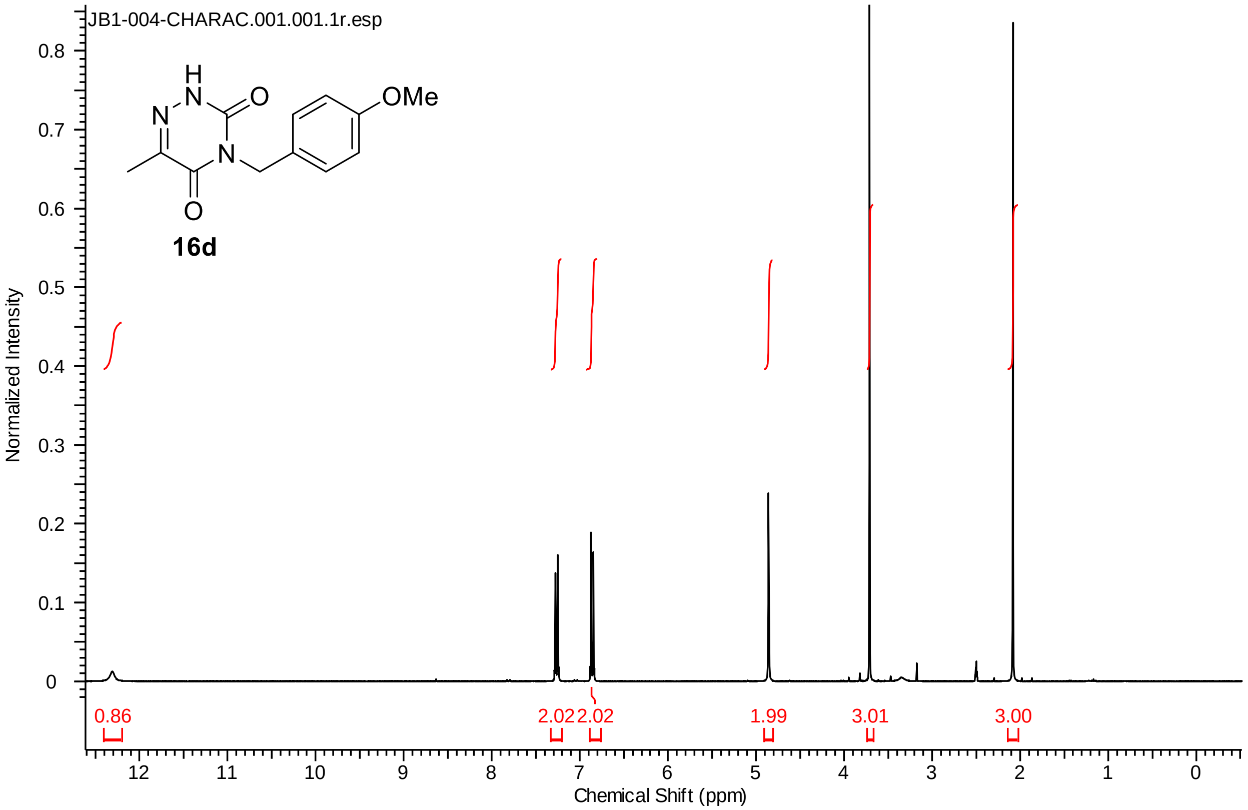
JB1-004-CHARAC.001.001.1r.esp
OMe
16d
0.86
2.02
2.02
1.99
3.01
3.00
Normalized Intensity
Chemical Shift (ppm)

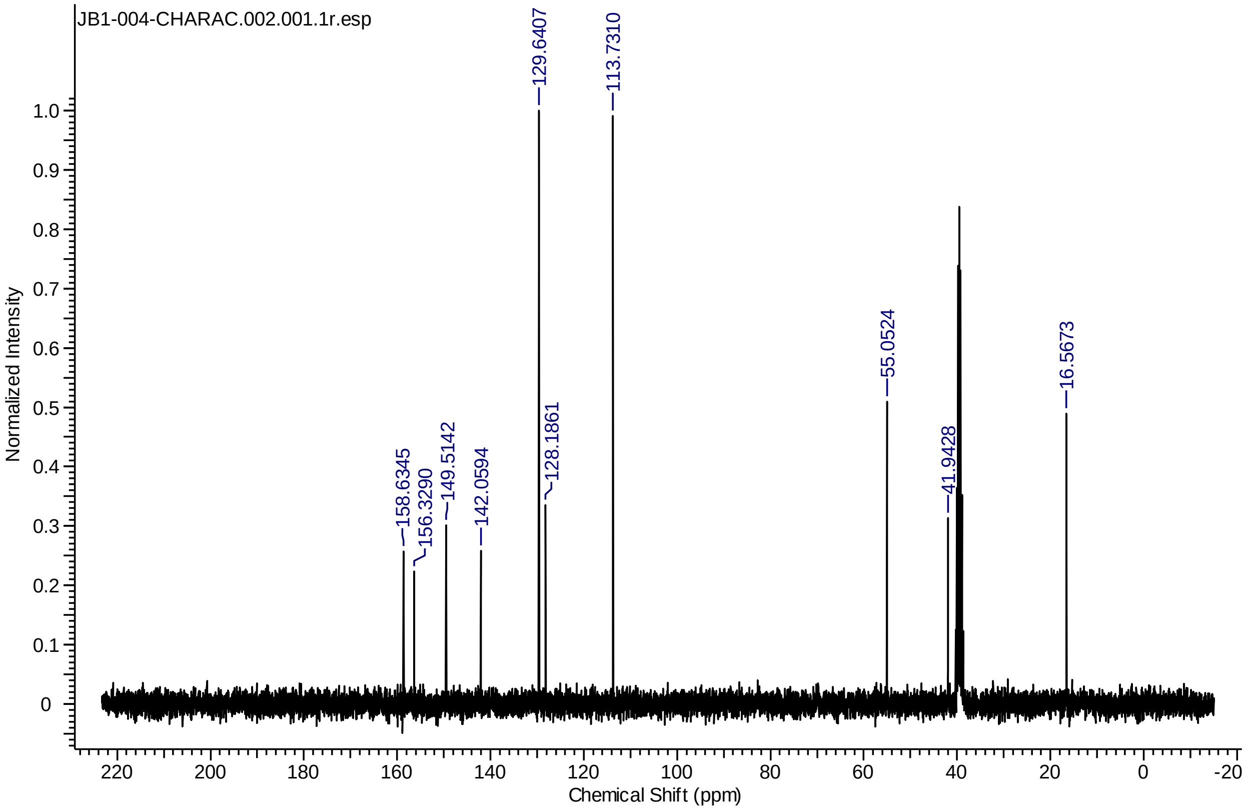
JB1-004-CHARAC.002.001.1r.esp
129.6407
113.7310
158.6345
156.3290
149.5142
142.0594
128.1861
55.0524
41.9428
16.5673
Normalized Intensity
Chemical Shift (ppm)

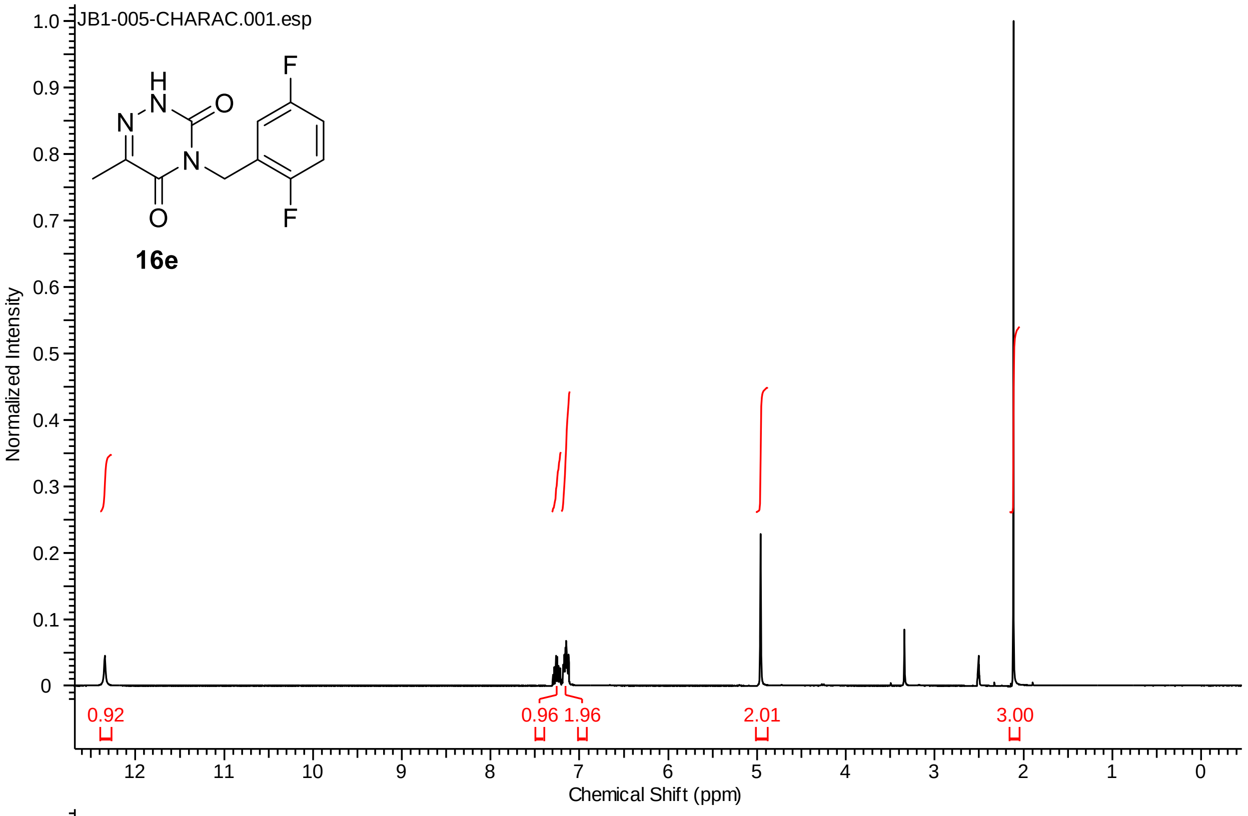
JB1-005-CHARAC.001.esp
16e
Normalized Intensity
0.92
0.96
1.96
2.01
3.00
Chemical Shift (ppm)

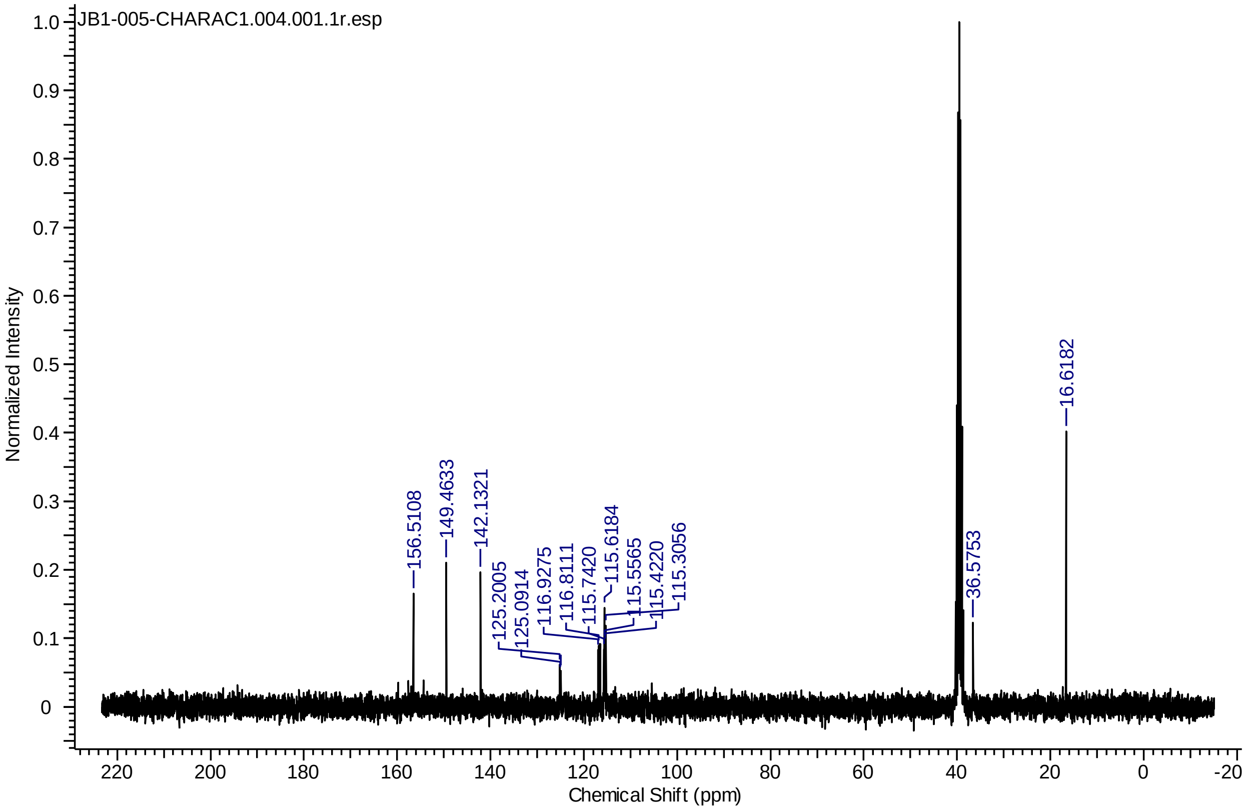
JB1-005-CHARAC1.004.001.1r.esp
Normalized Intensity
156.5108
149.4633
142.1321
125.2005
125.0914
116.9275
116.8111
115.7420
115.6184
115.5565
115.4220
115.3056
36.5753
16.6182
Chemical Shift (ppm)

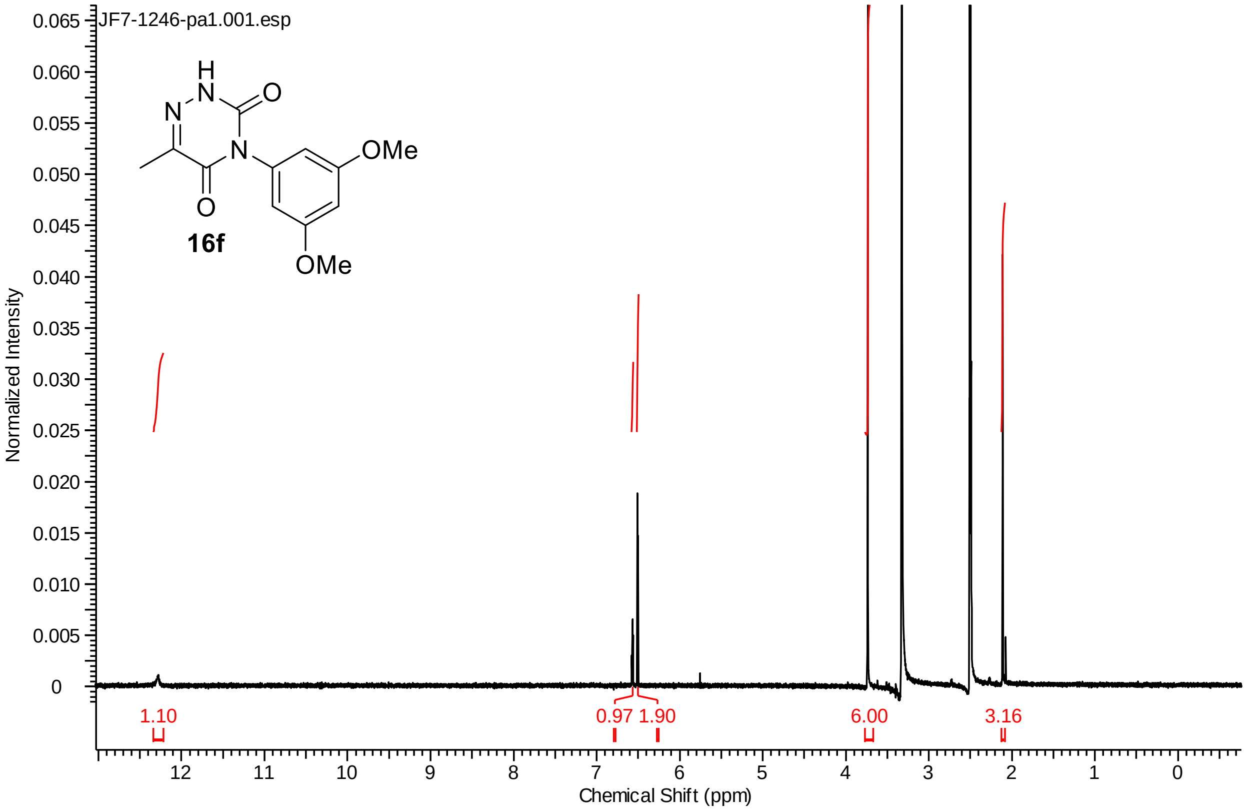
JF7-1246-pa1.001.esp
16f
OMe
OMe
1.10
0.97 1.90
6.00
3.16
Normalized Intensity
Chemical Shift (ppm)

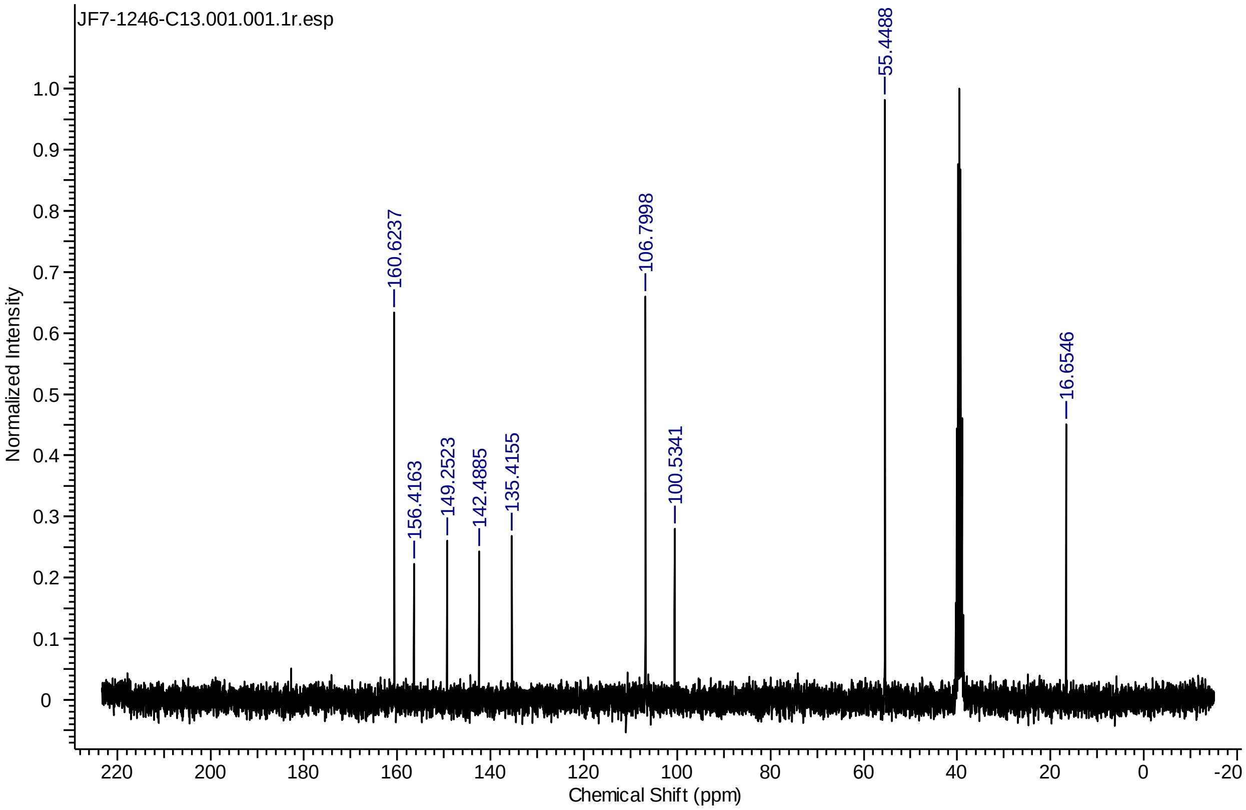
JF7-1246-C13.001.001.1r.esp
160.6237
156.4163
149.2523
142.4885
135.4155
106.7998
100.5341
55.4488
16.6546
Normalized Intensity
Chemical Shift (ppm)

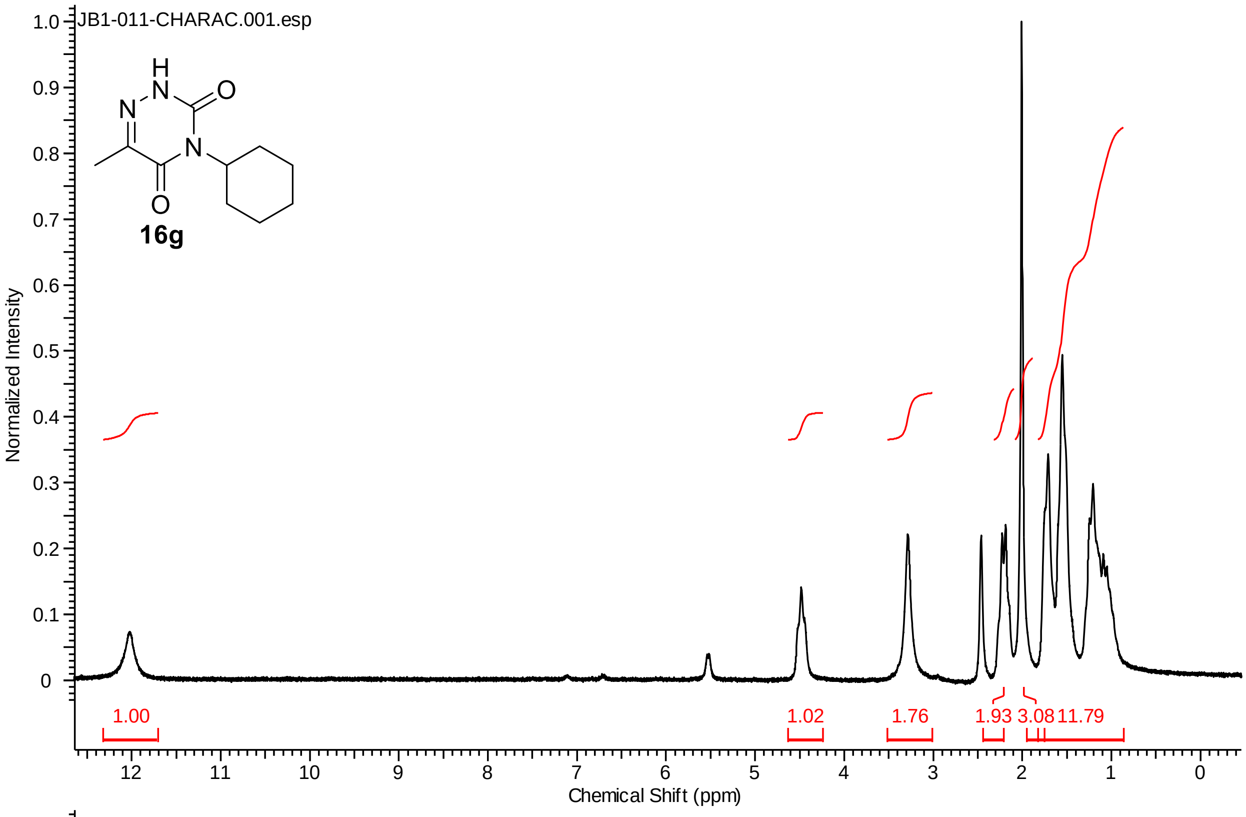
JB1-011-CHARAC.001.esp
16g
Normalized Intensity
1.00
1.02
1.76
1.93
3.08
11.79
Chemical Shift (ppm)

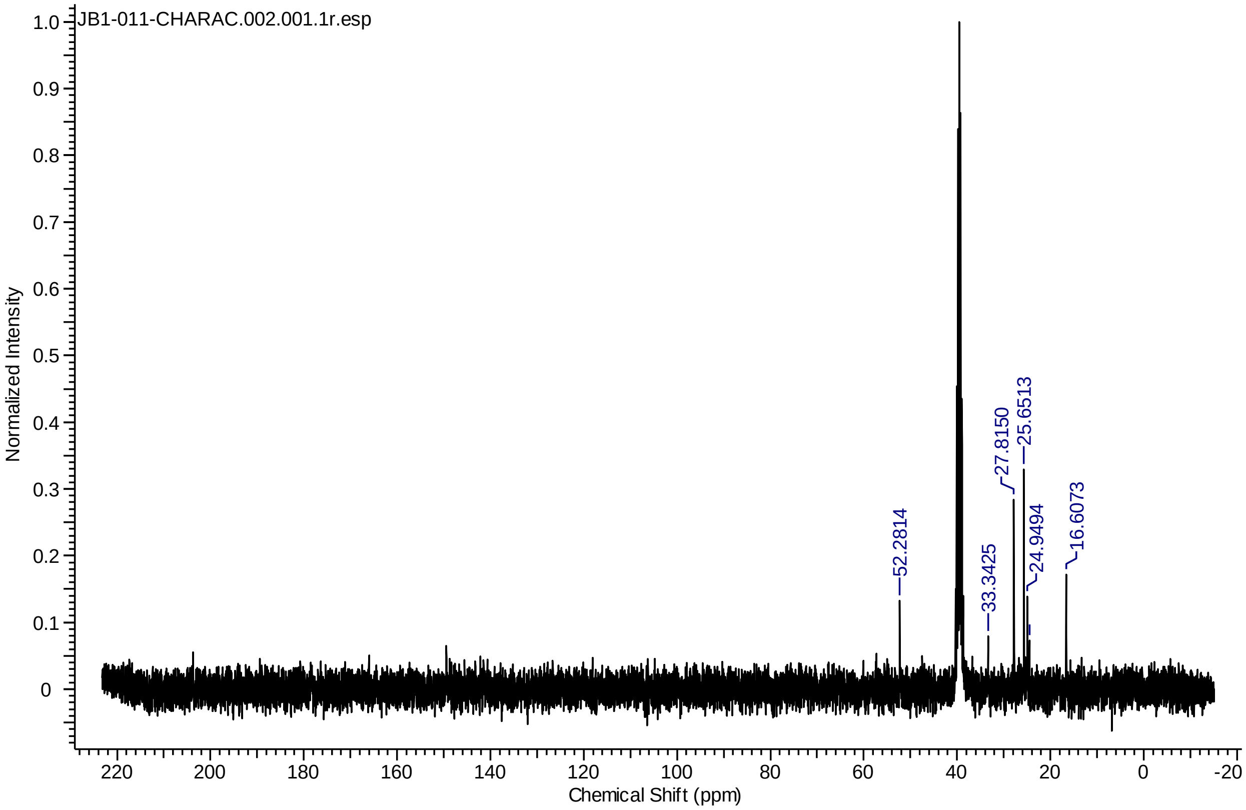
JB1-011-CHARAC.002.001.1r.esp
Normalized Intensity
52.2814
33.3425
27.8150
25.6513
24.9494
16.6073
Chemical Shift (ppm)

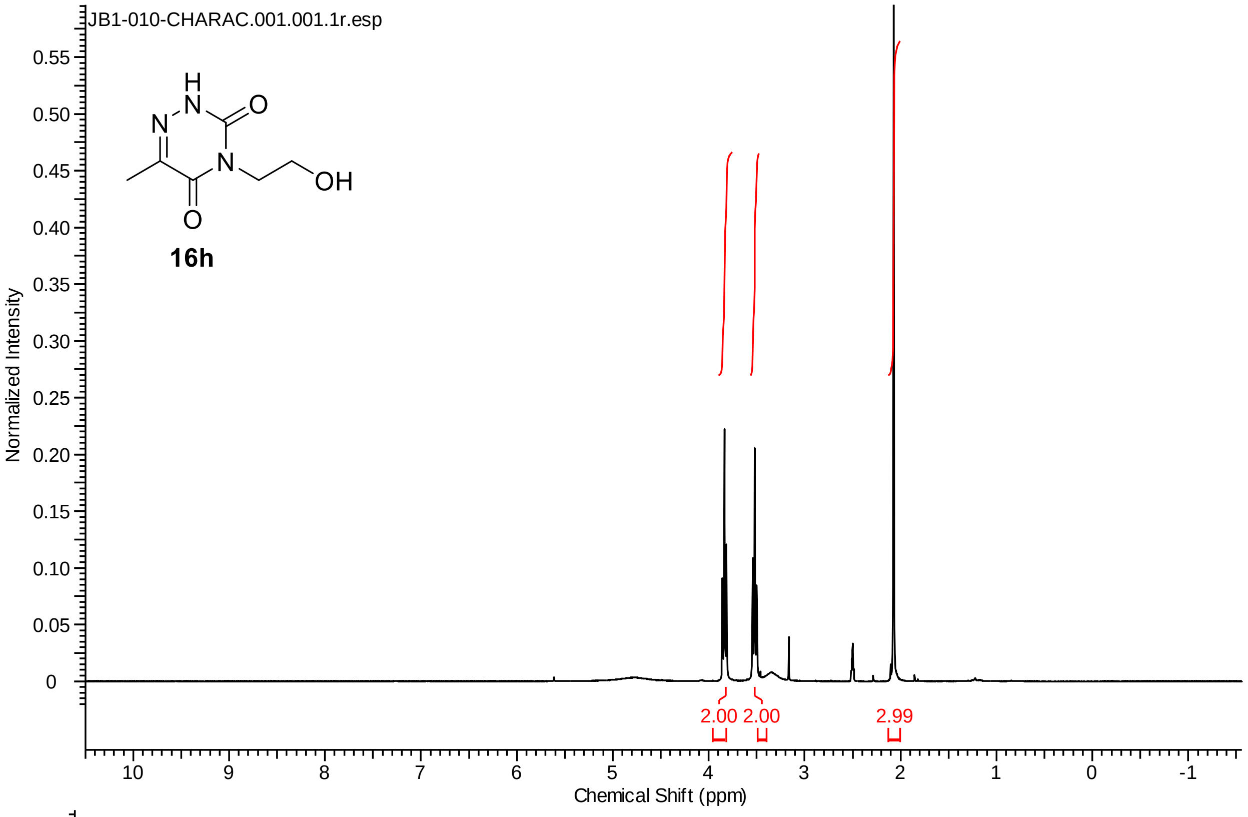
JB1-010-CHARAC.001.001.1r.esp
H
N
N
O
N
OH
O
16h
Normalized Intensity
2.00
2.00
2.99
Chemical Shift (ppm)

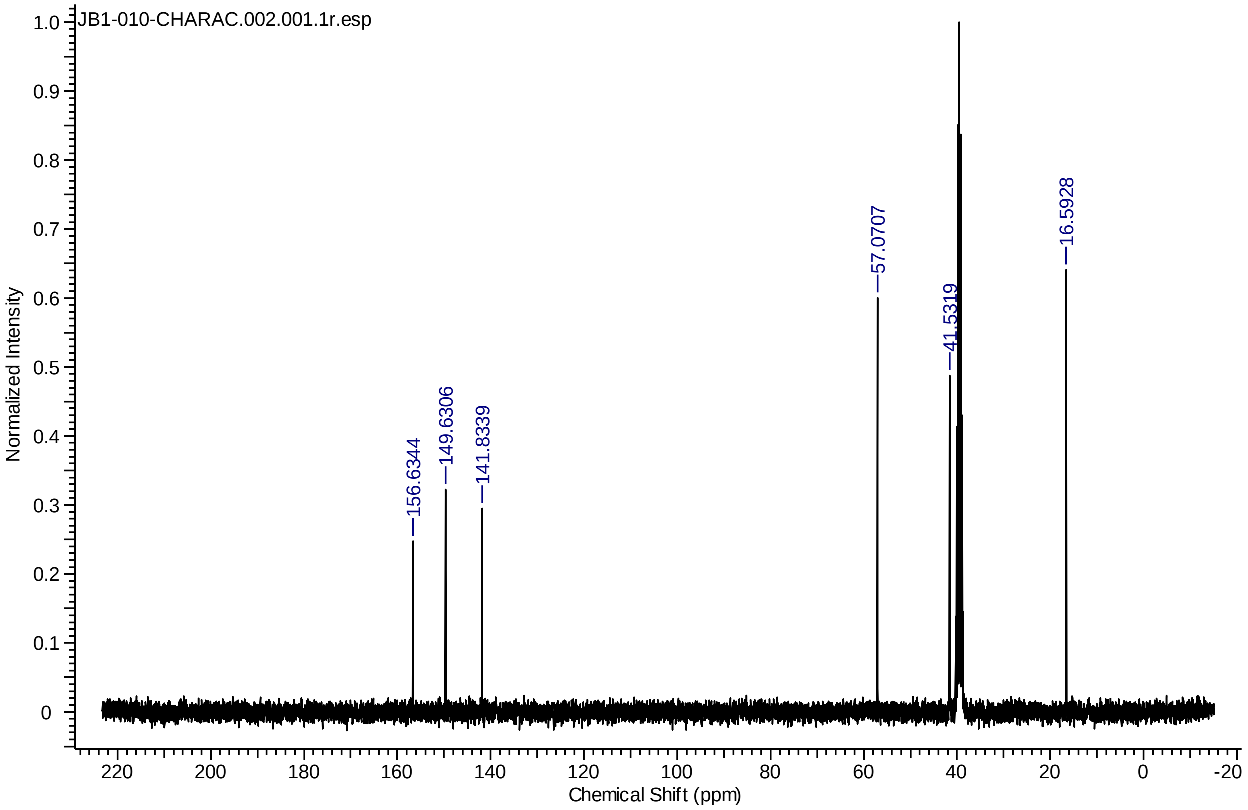
JB1-010-CHARAC.002.001.1r.esp
Normalized Intensity
156.6344
149.6306
141.8339
57.0707
41.5319
16.5928
Chemical Shift (ppm)

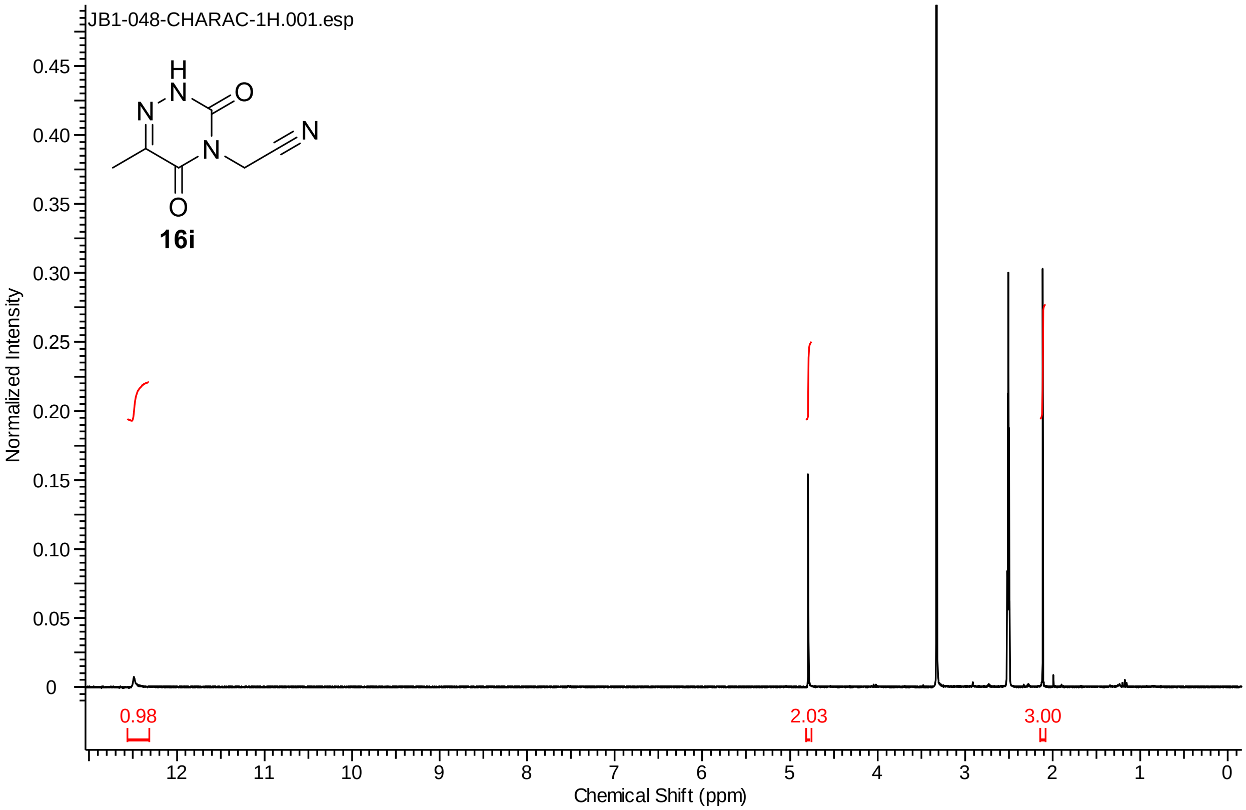
JB1-048-CHARAC-1H.001.esp
16i
Normalized Intensity
0.98
2.03
3.00
Chemical Shift (ppm)

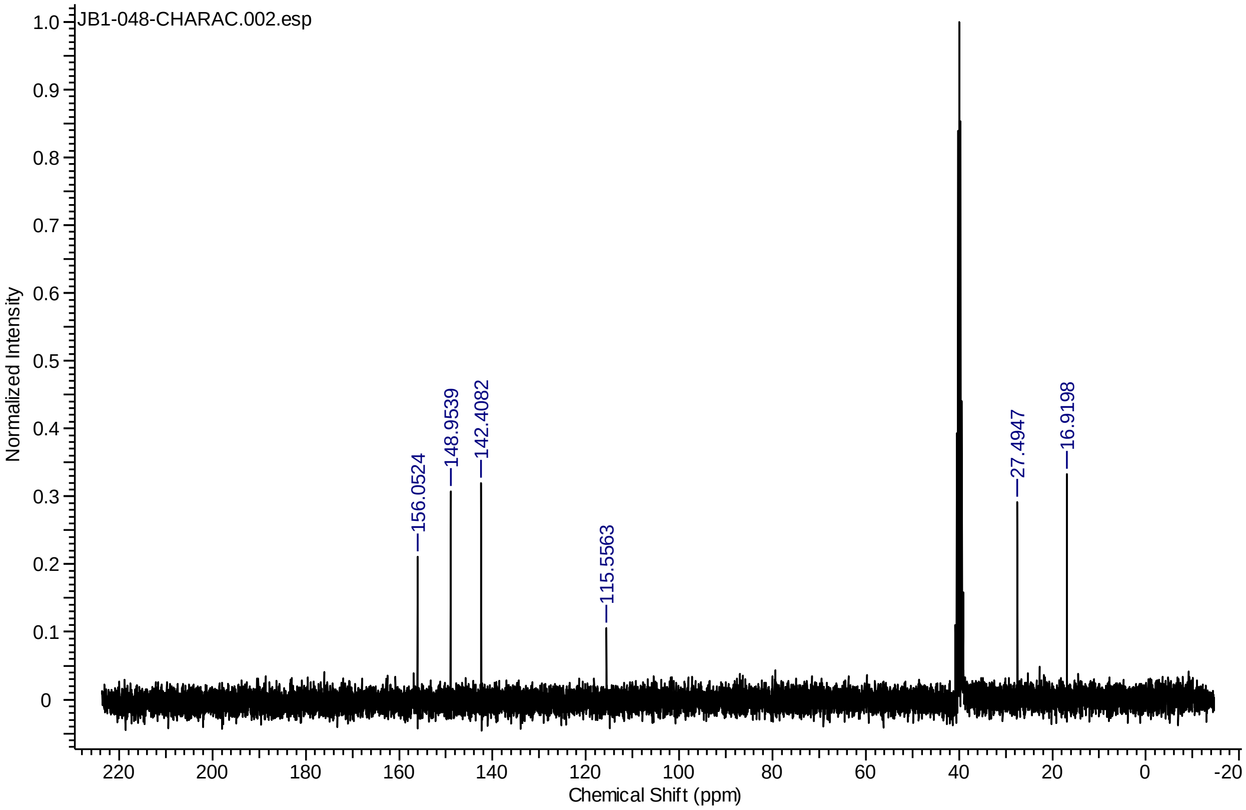
JB1-048-CHARAC.002.esp
Normalized Intensity
156.0524
148.9539
142.4082
115.5563
27.4947
16.9198
Chemical Shift (ppm)

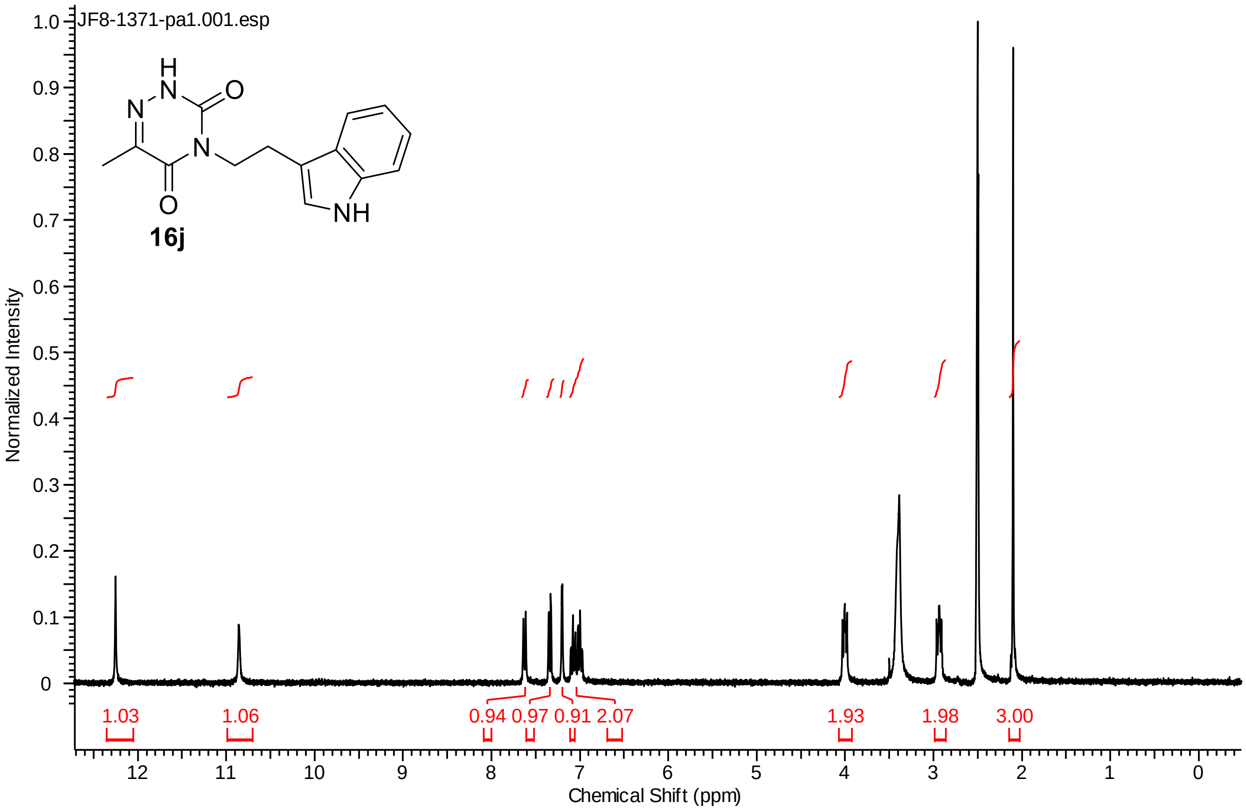
JF8-1371-pa1.001.esp
16j
Normalized Intensity
1.03
1.06
0.94
0.97
0.91
2.07
1.93
1.98
3.00
Chemical Shift (ppm)

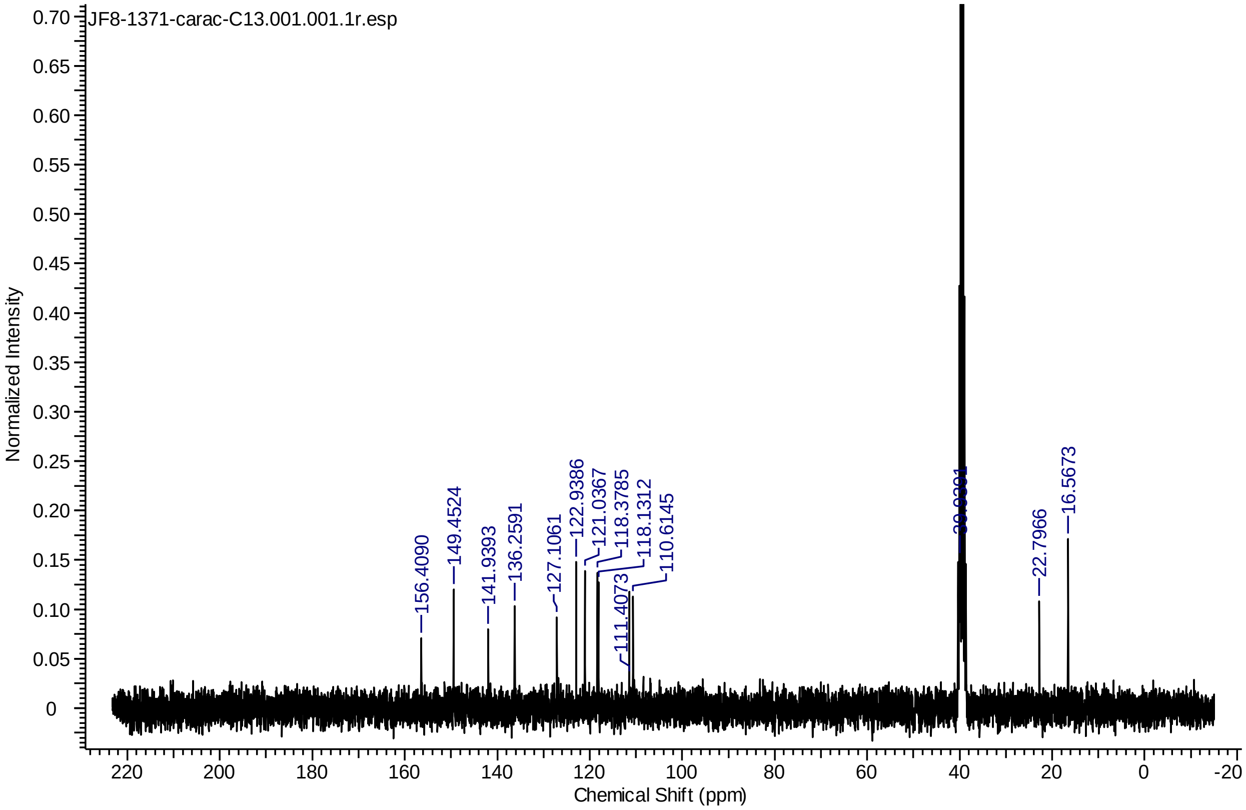
JF8-1371-carac-C13.001.001.1r.esp
Normalized Intensity
156.4090
149.4524
141.9393
136.2591
127.1061
122.9386
121.0367
118.3785
118.1312
111.4073
110.6145
22.7966
16.5673
Chemical Shift (ppm)

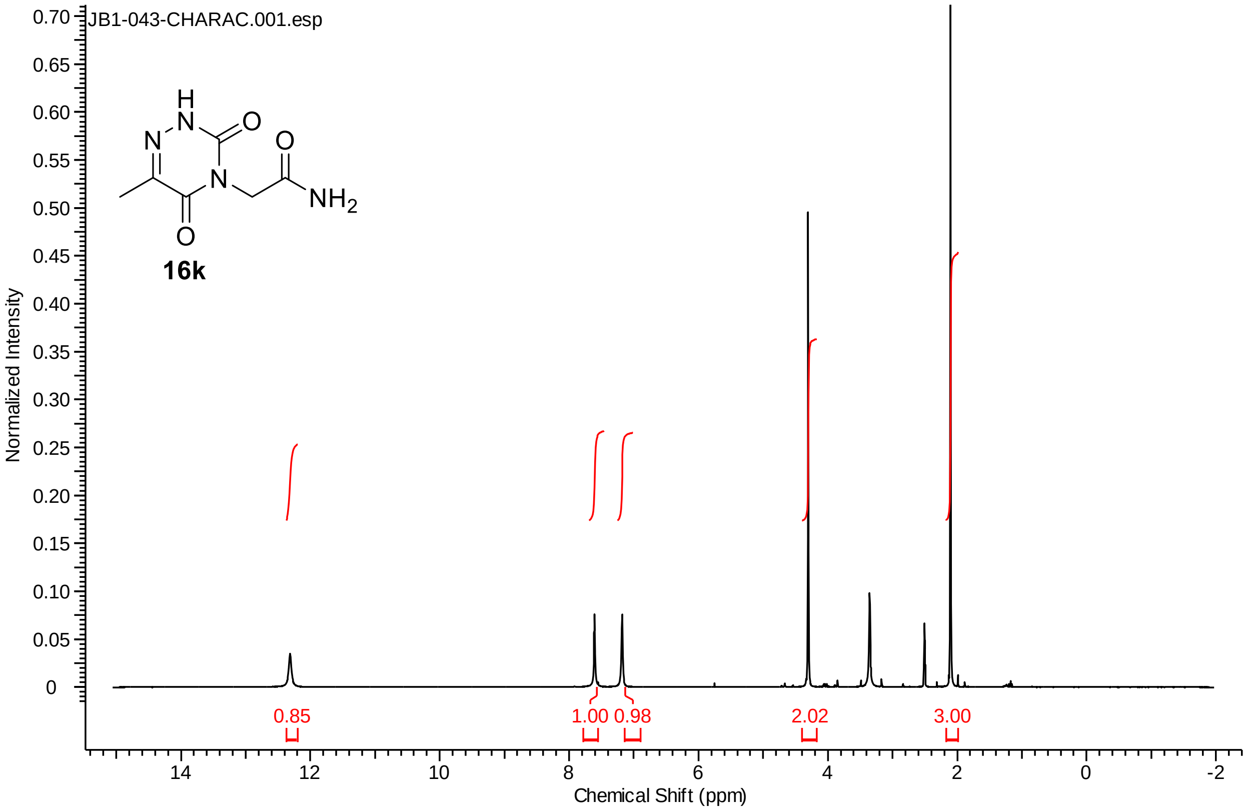

JB1-043-CHARAC.001.esp
16k
Normalized Intensity
Chemical Shift (ppm)
0.85
1.00
0.98
2.02
3.00

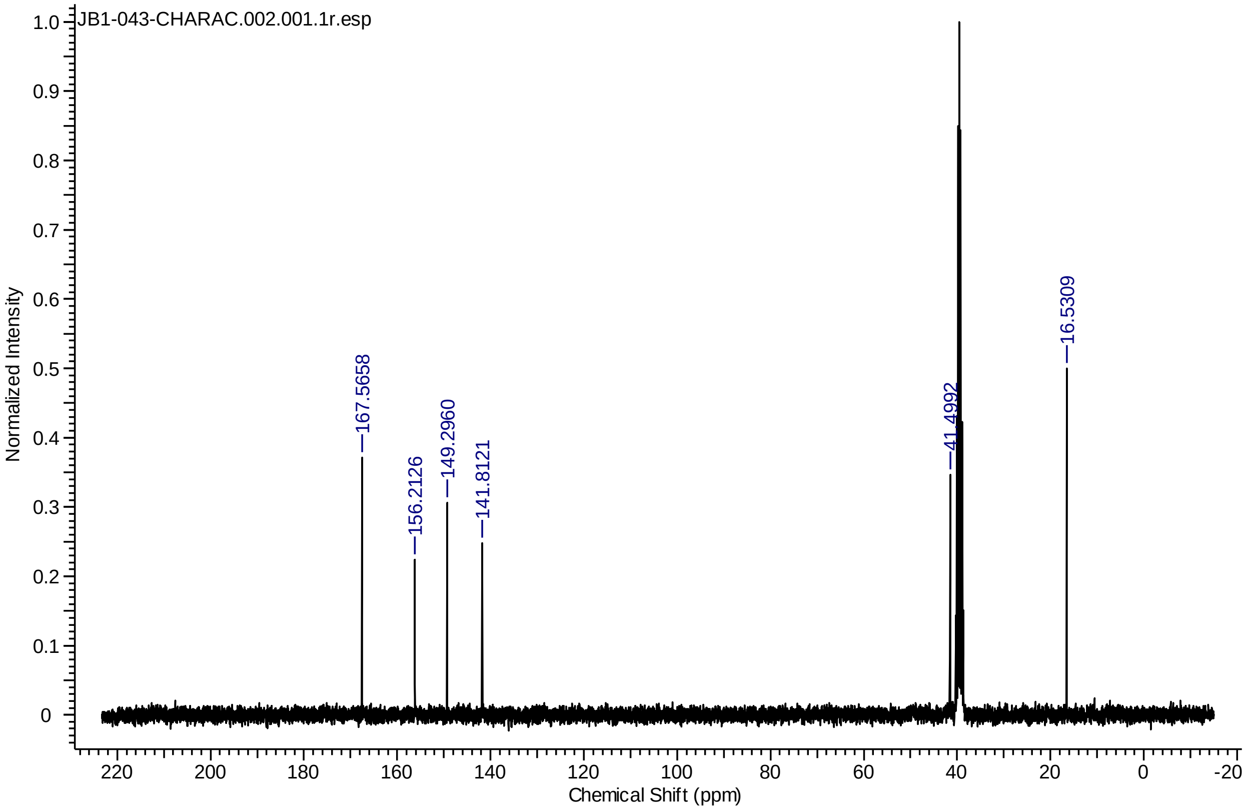

JB1-043-CHARAC.002.001.1r.esp
Normalized Intensity
Chemical Shift (ppm)
167.5658
156.2126
149.2960
141.8121
41.4992
16.5309

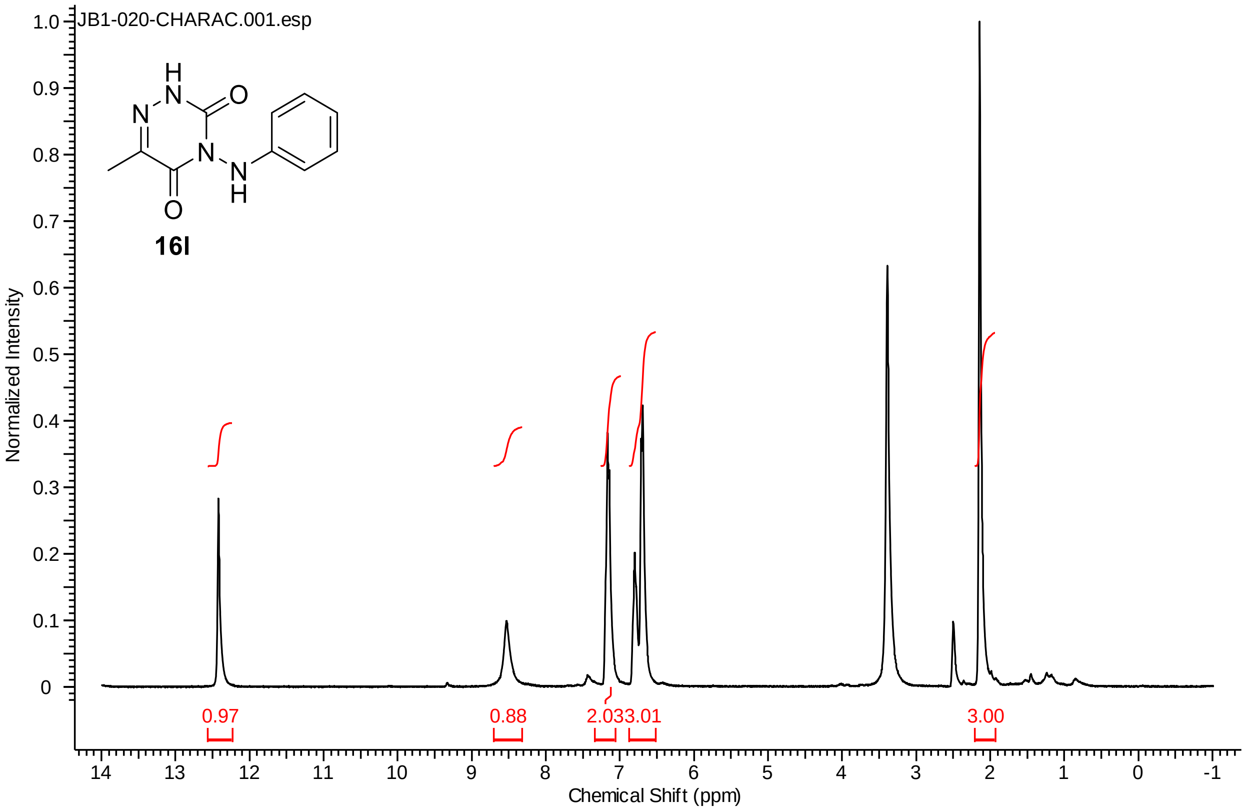
JB1-020-CHARAC.001.esp
16l
Normalized Intensity
0.97
0.88
2.03
3.01
3.00
Chemical Shift (ppm)

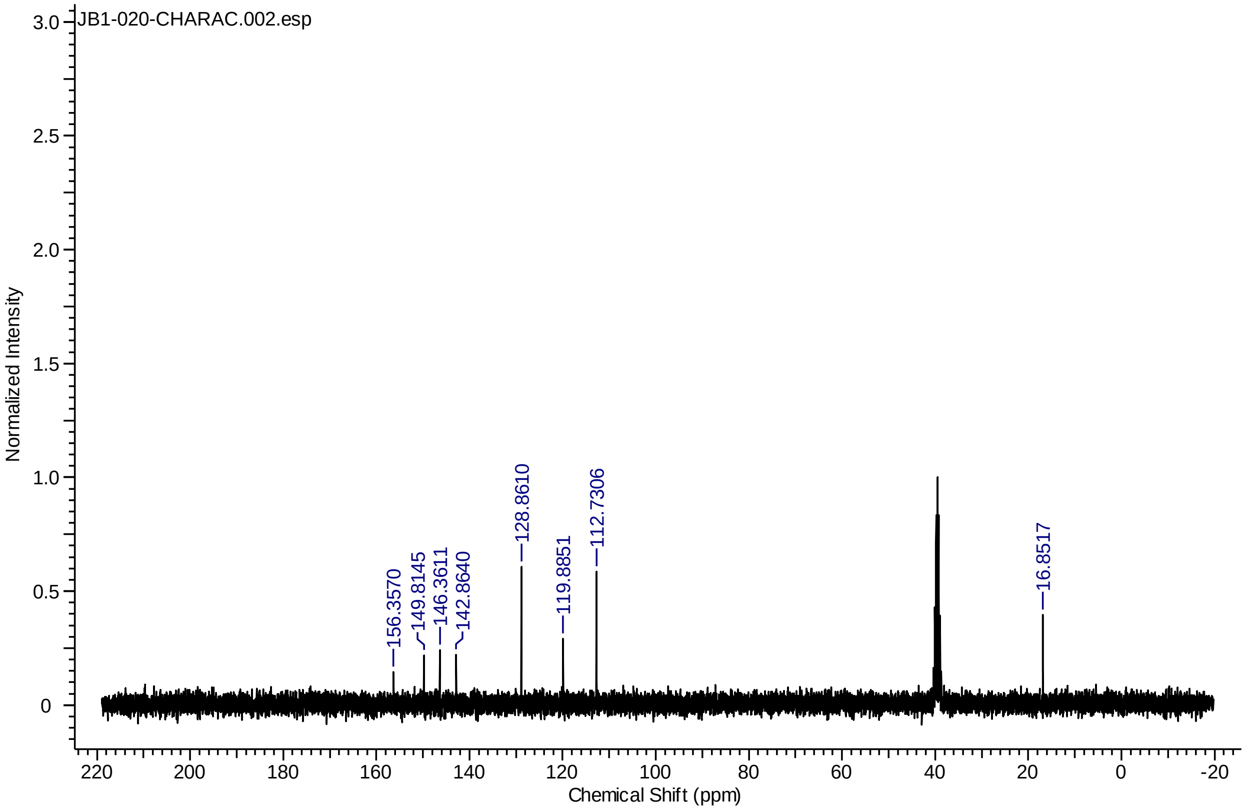
JB1-020-CHARAC.002.esp
Normalized Intensity
156.3570
149.8145
146.3611
142.8640
128.8610
119.8851
112.7306
16.8517
Chemical Shift (ppm)

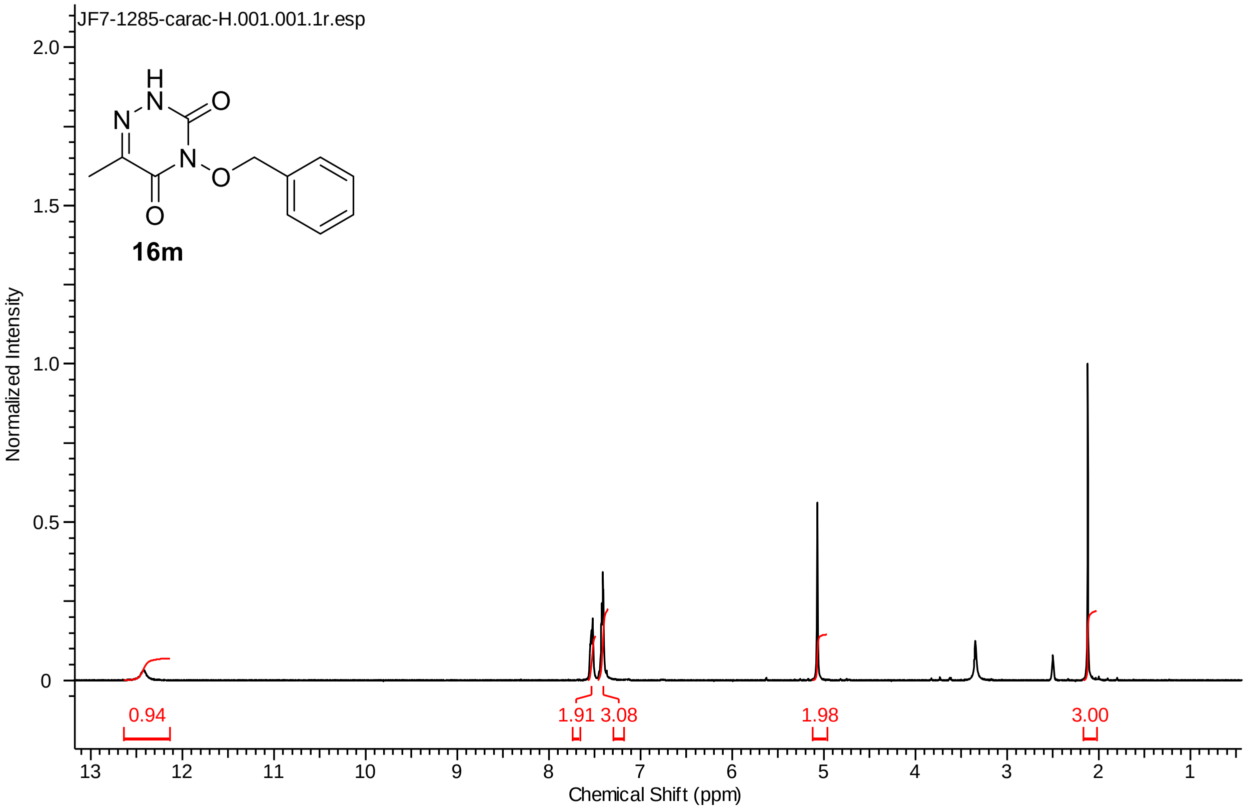
JF7-1285-carac-H.001.001.1r.esp
16m
0.94
1.91
3.08
1.98
3.00
Normalized Intensity
Chemical Shift (ppm)

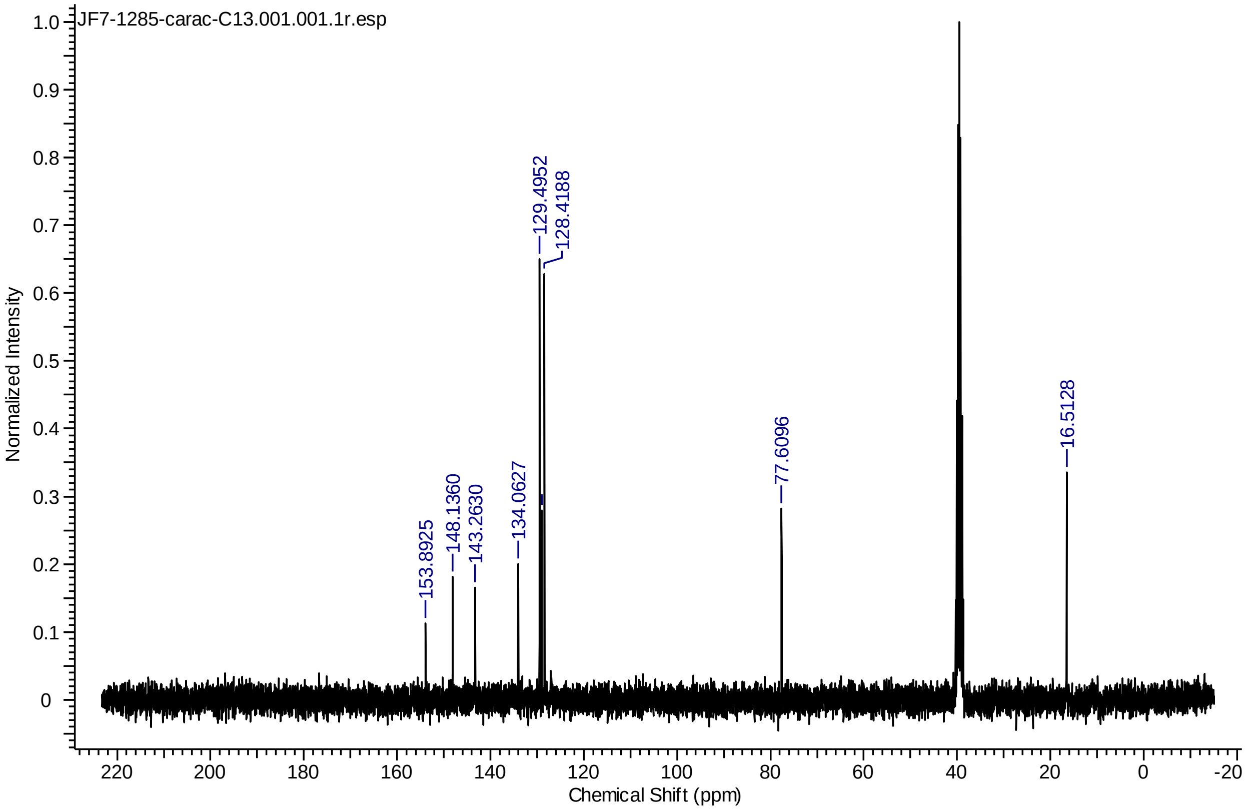
JF7-1285-carac-C13.001.001.1r.esp
153.8925
148.1360
143.2630
134.0627
129.4952
128.4188
77.6096
16.5128
Normalized Intensity
Chemical Shift (ppm)

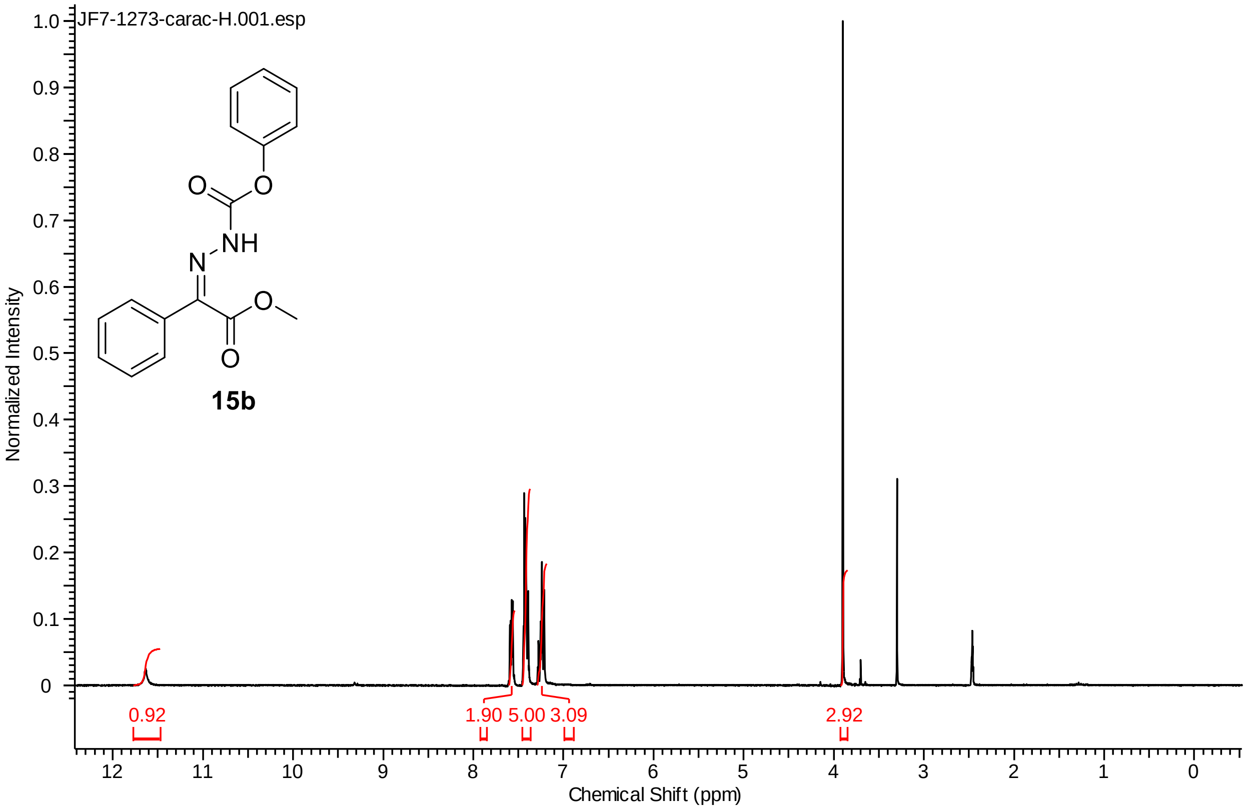
JF7-1273-carac-H.001.esp
O
O
NH
N
O
O
15b
Normalized Intensity
0.92
1.90
5.00
3.09
2.92
Chemical Shift (ppm)

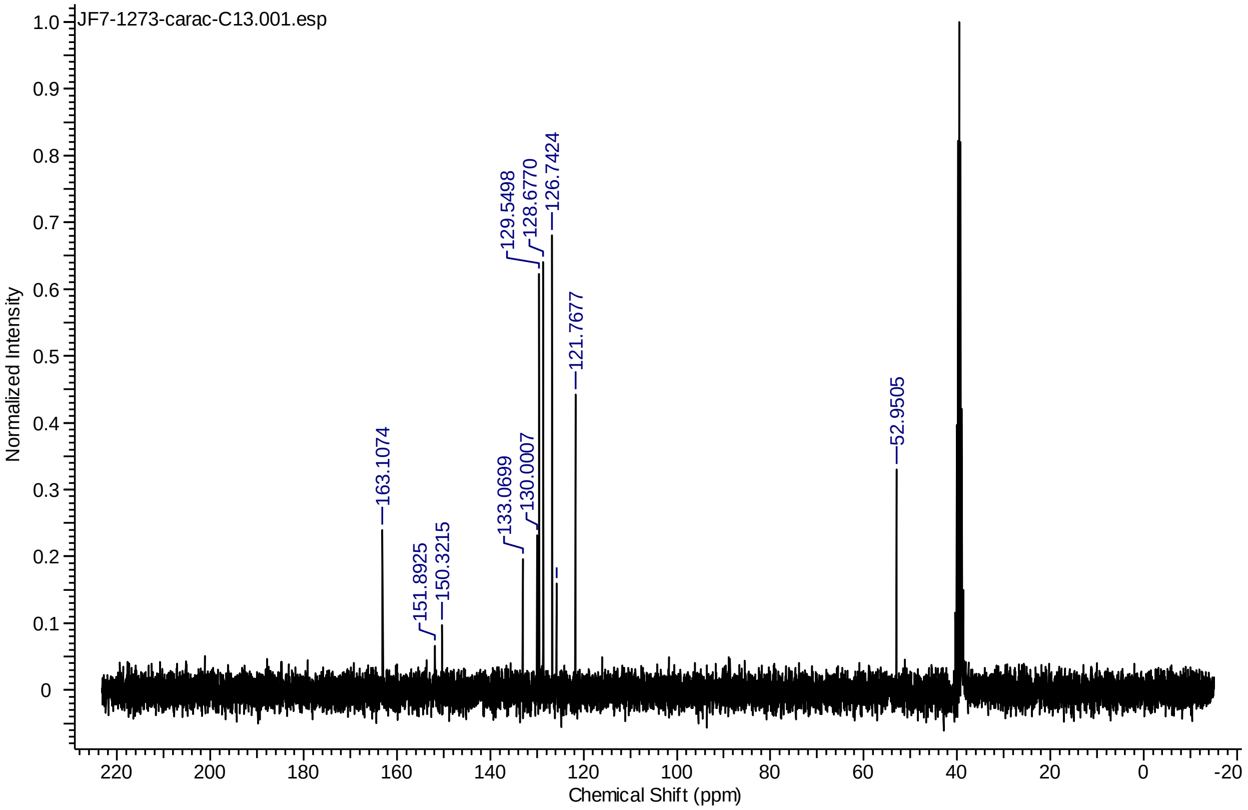
JF7-1273-carac-C13.001.esp
Normalized Intensity
163.1074
151.8925
150.3215
133.0699
130.0007
129.5498
128.6770
126.7424
121.7677
52.9505
Chemical Shift (ppm)

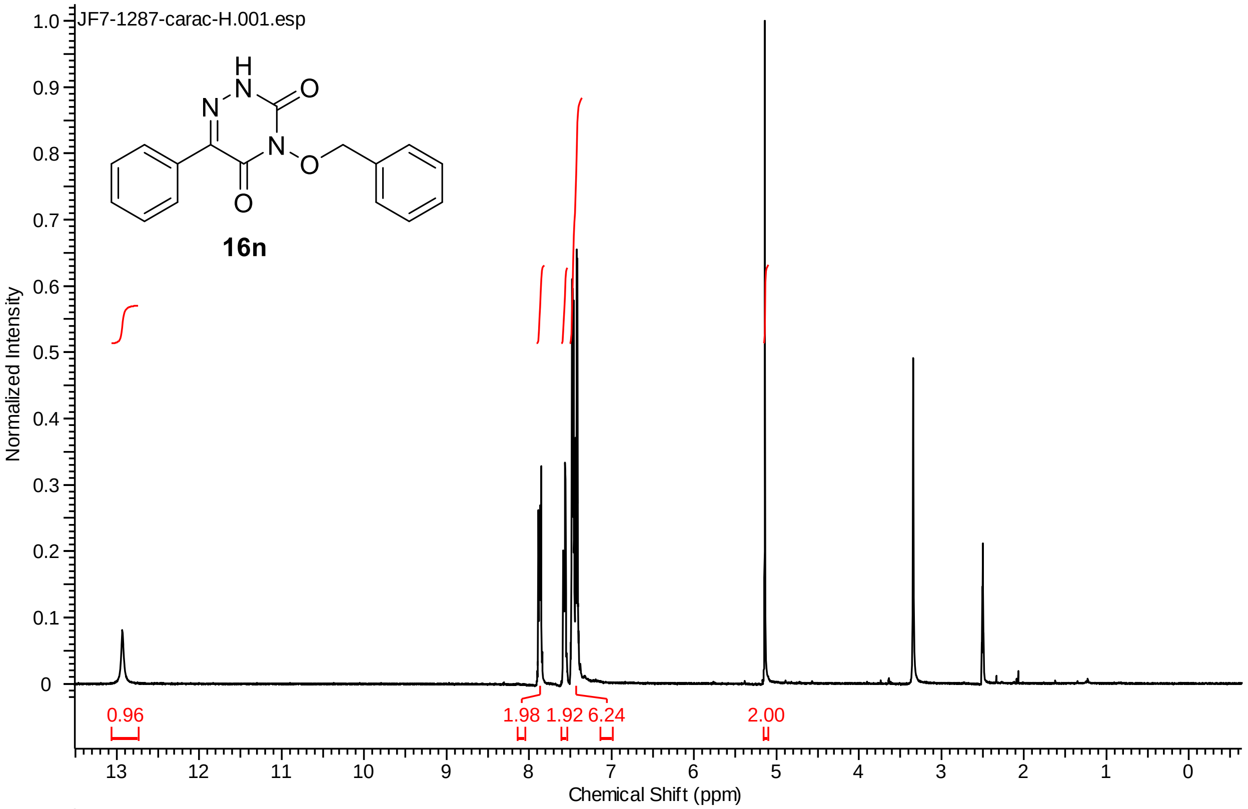
JF7-1287-carac-H.001.esp
16n
Normalized Intensity
0.96
1.98
1.92
6.24
2.00
Chemical Shift (ppm)

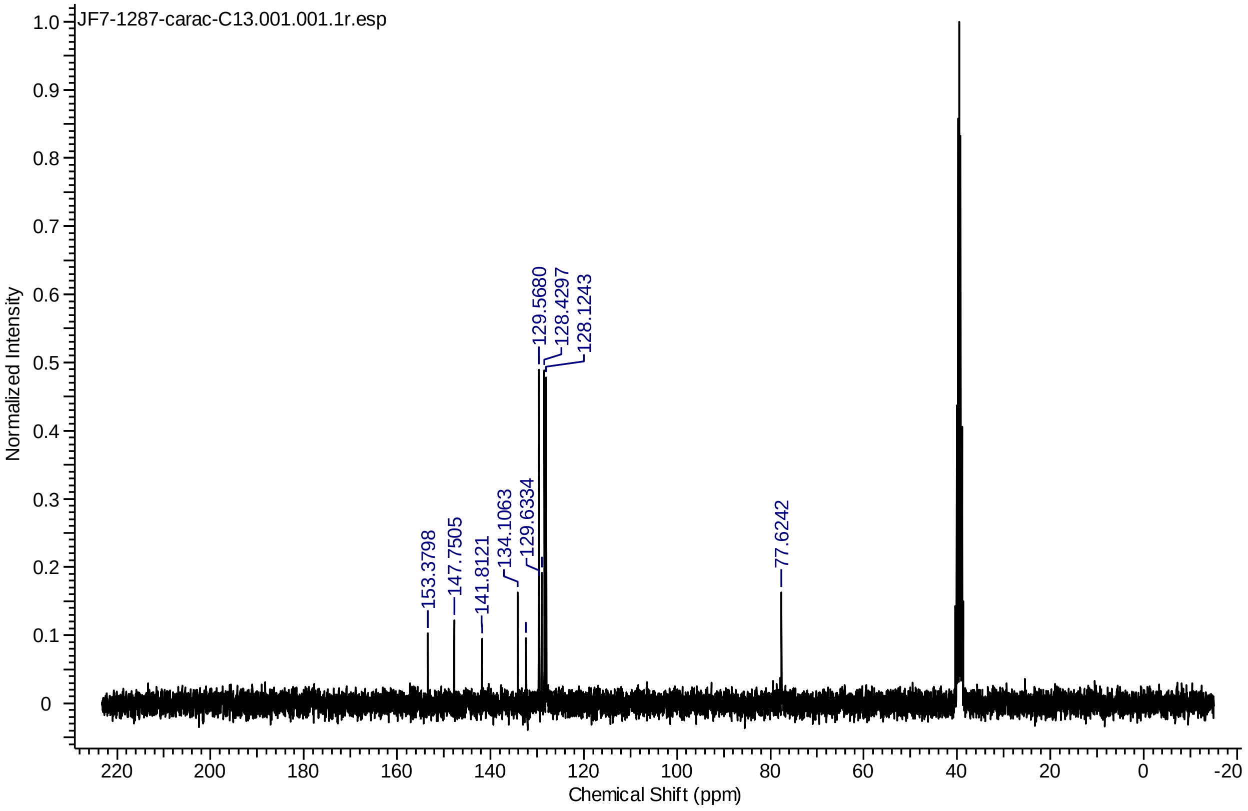
JF7-1287-carac-C13.001.001.1r.esp
Normalized Intensity
153.3798
147.7505
141.8121
134.1063
129.6334
129.5680
128.4297
128.1243
77.6242
Chemical Shift (ppm)

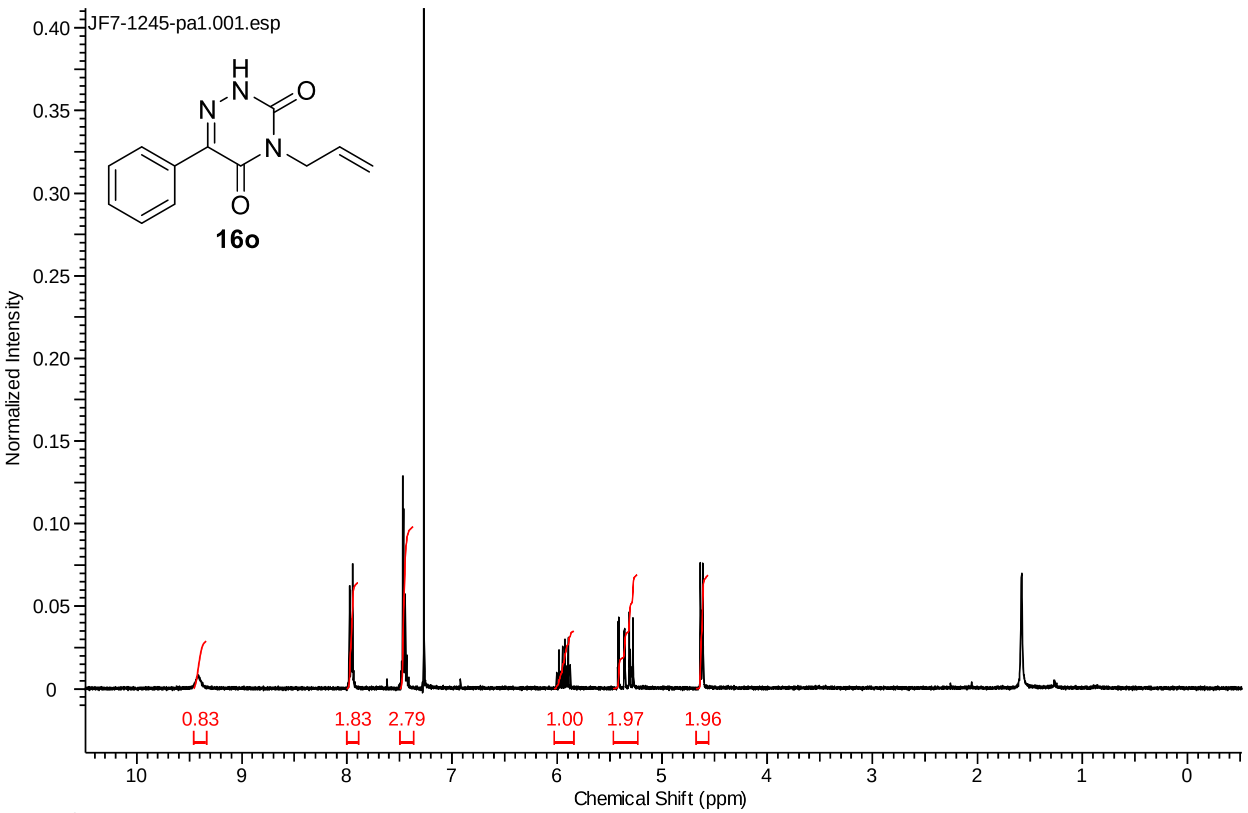
JF7-1245-pa1.001.esp
16o
Normalized Intensity
0.83
1.83
2.79
1.00
1.97
1.96
Chemical Shift (ppm)

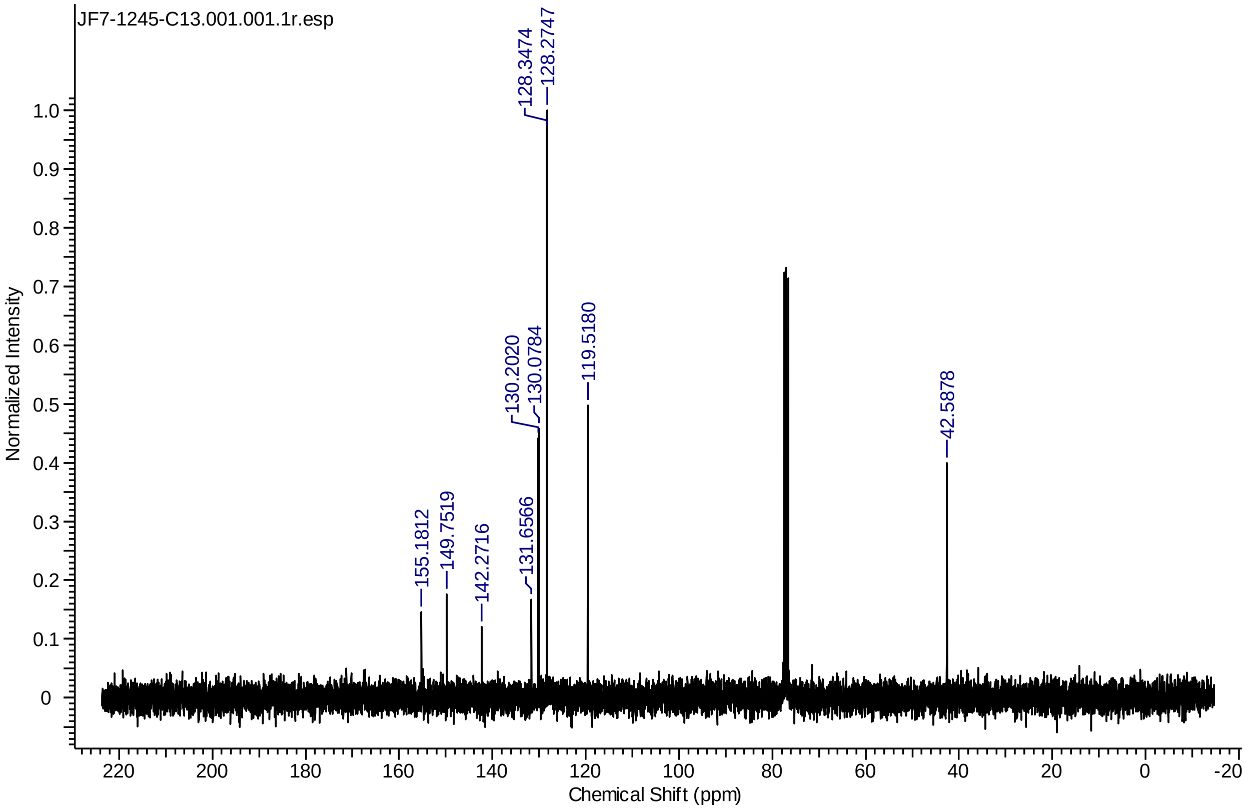
JF7-1245-C13.001.001.1r.esp
128.3474
128.2747
130.2020
130.0784
119.5180
42.5878
155.1812
149.7519
142.2716
131.6566
Normalized Intensity
Chemical Shift (ppm)

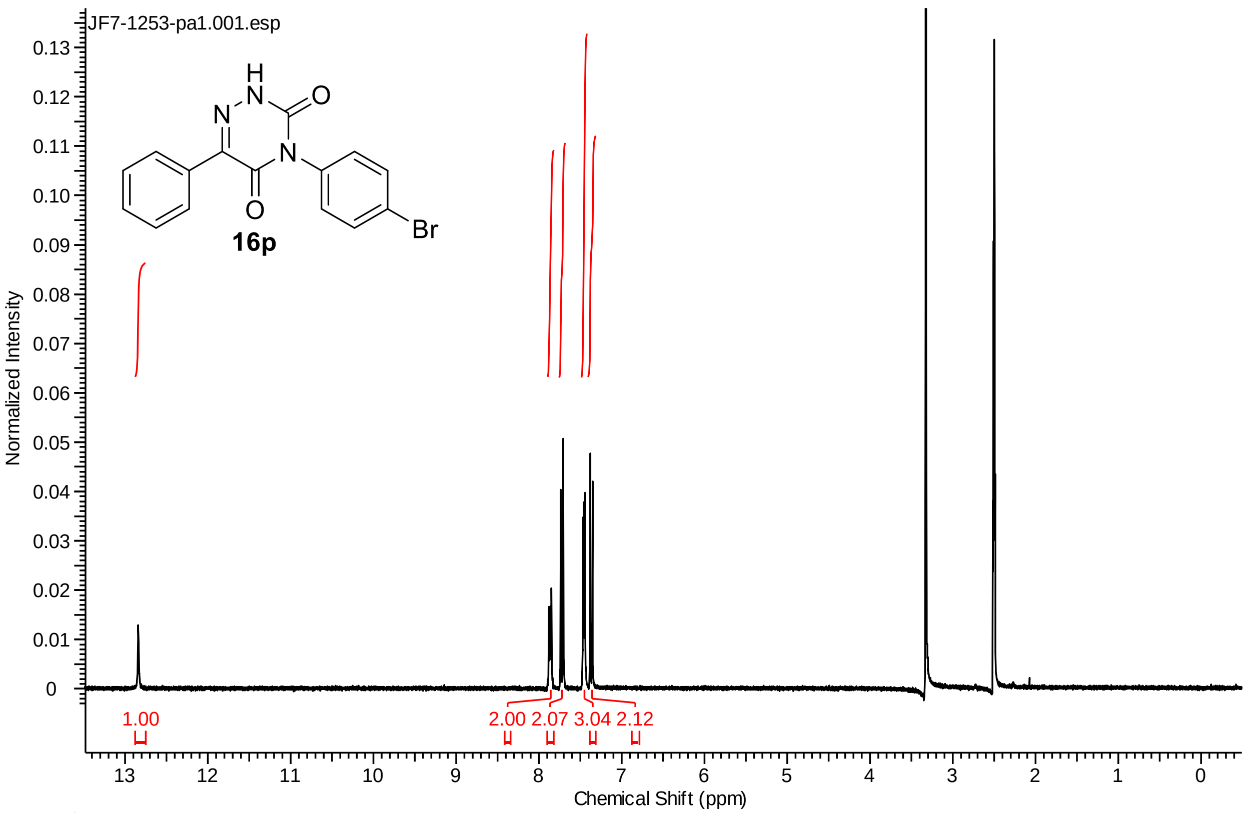
JF7-1253-pa1.001.esp
16p
Normalized Intensity
Chemical Shift (ppm)
1.00
2.00
2.07
3.04
2.12

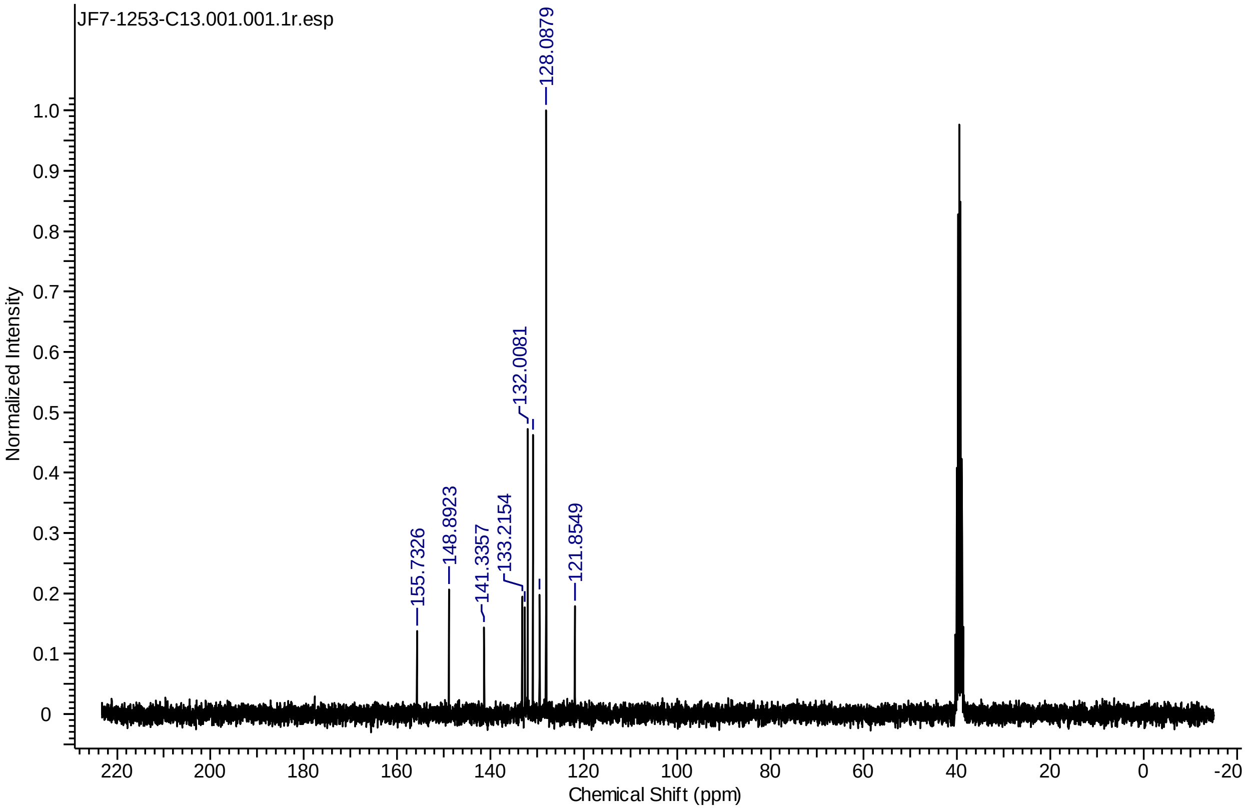
JF7-1253-C13.001.001.1r.esp
Normalized Intensity
Chemical Shift (ppm)
155.7326
148.8923
141.3357
133.2154
132.0081
128.0879
121.8549

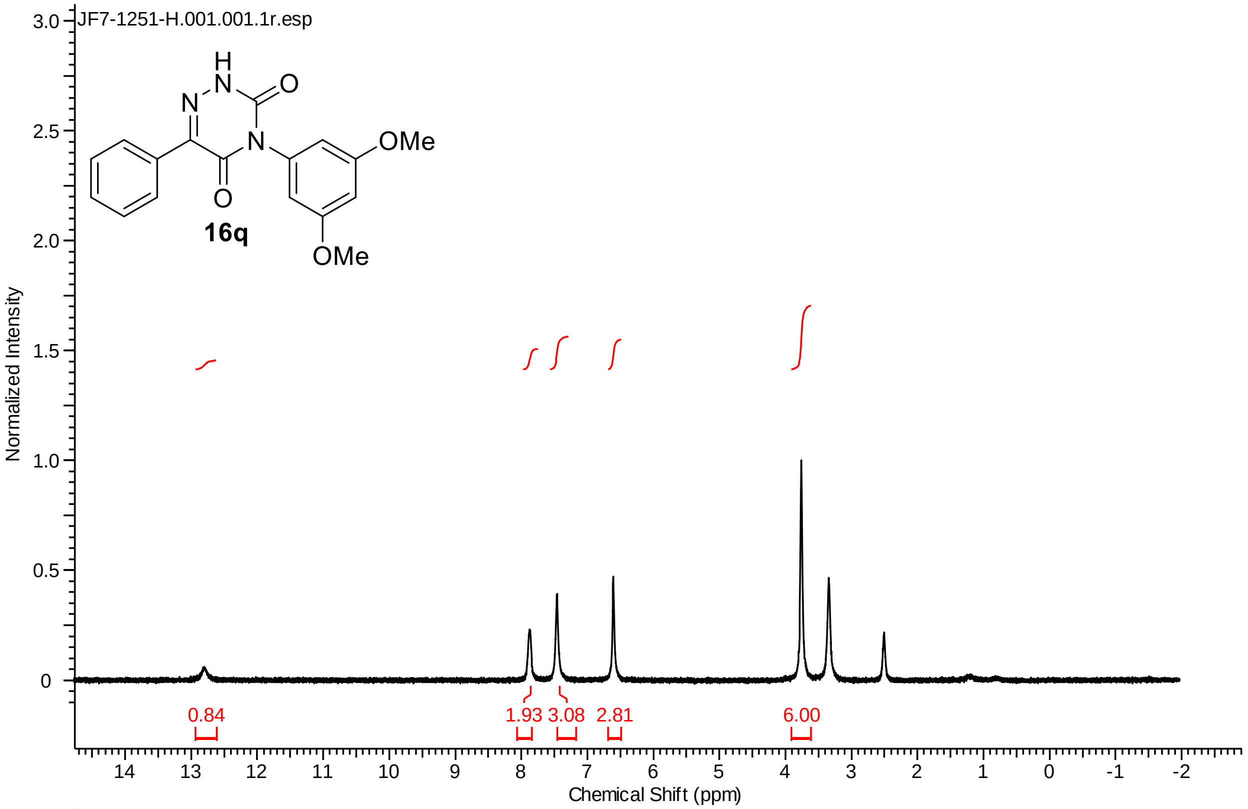
JF7-1251-H.001.001.1r.esp
H
N
N
O
N
OMe
O
16q
OMe
Normalized Intensity
0.84
1.93
3.08
2.81
6.00
Chemical Shift (ppm)

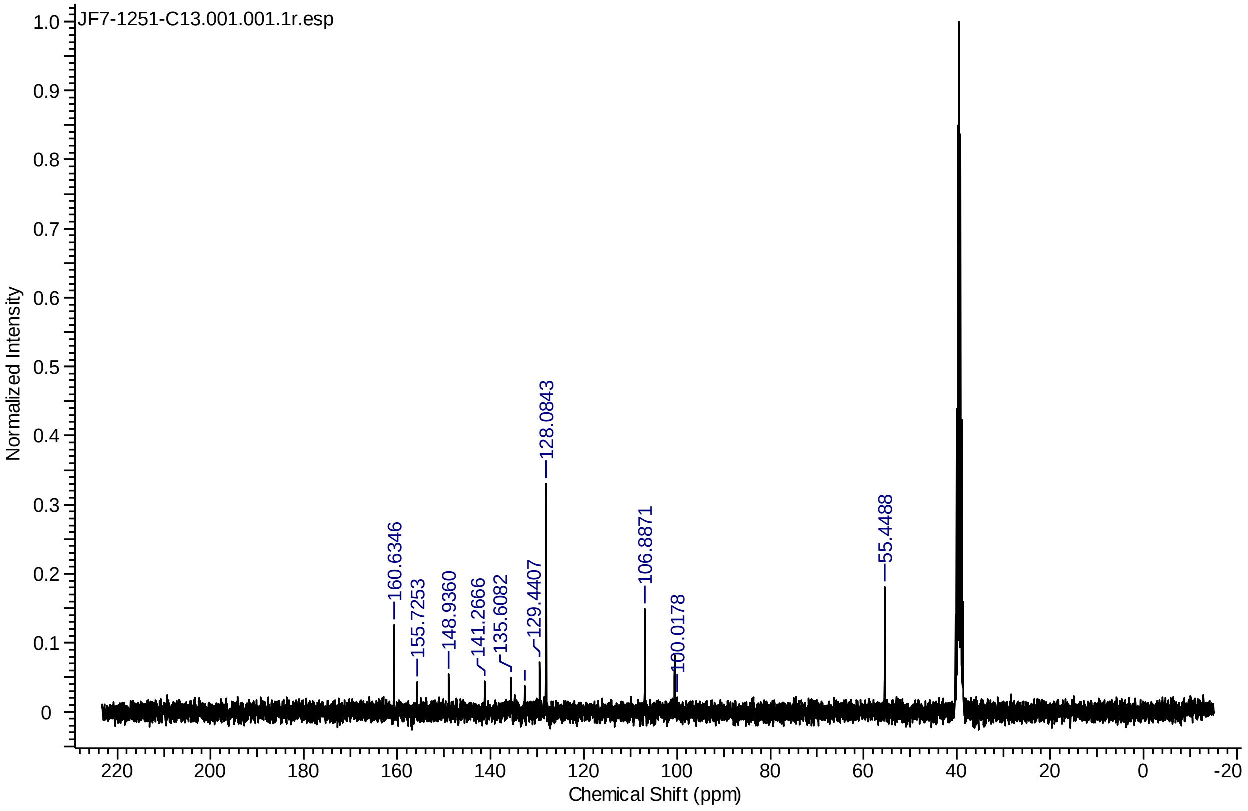
JF7-1251-C13.001.001.1r.esp
Normalized Intensity
160.6346
155.7253
148.9360
141.2666
135.6082
129.4407
128.0843
106.8871
100.0178
55.4488
Chemical Shift (ppm)

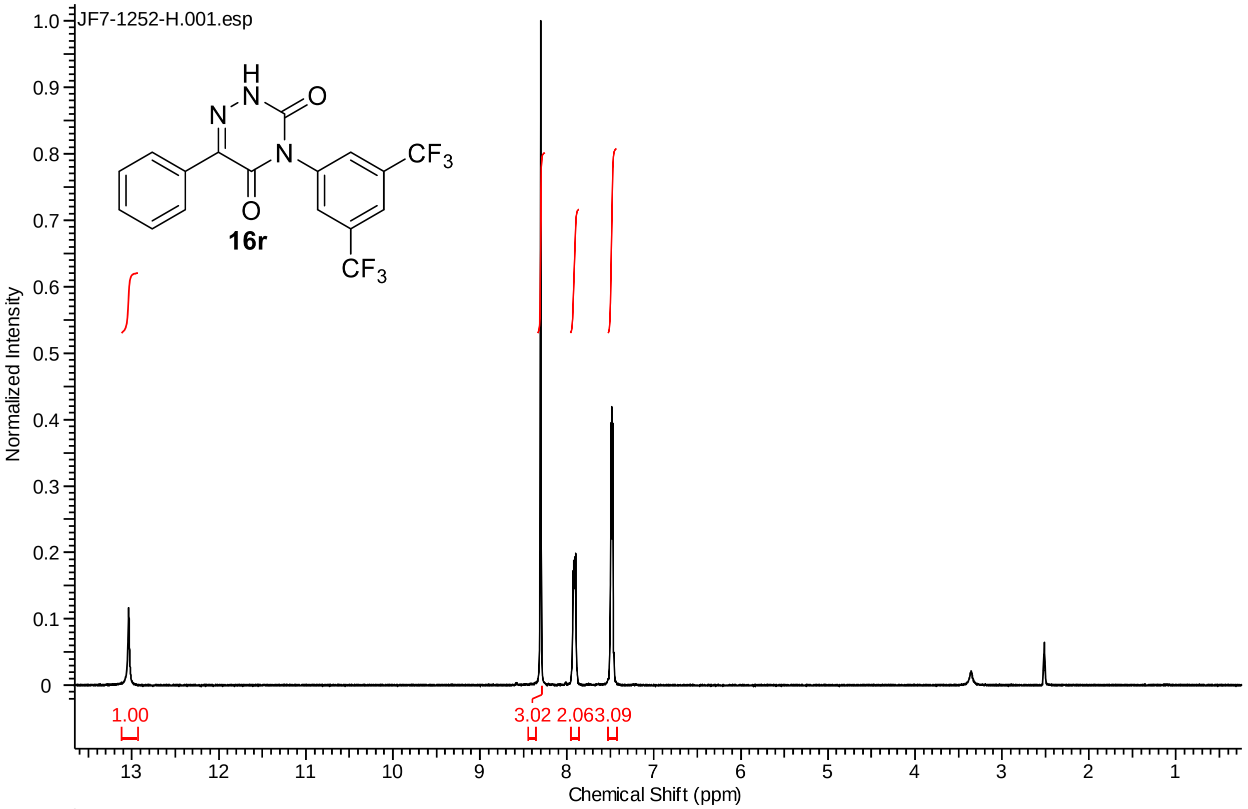
JF7-1252-H.001.esp
16r
Normalized Intensity
Chemical Shift (ppm)
1.00
3.02
2.06
3.09

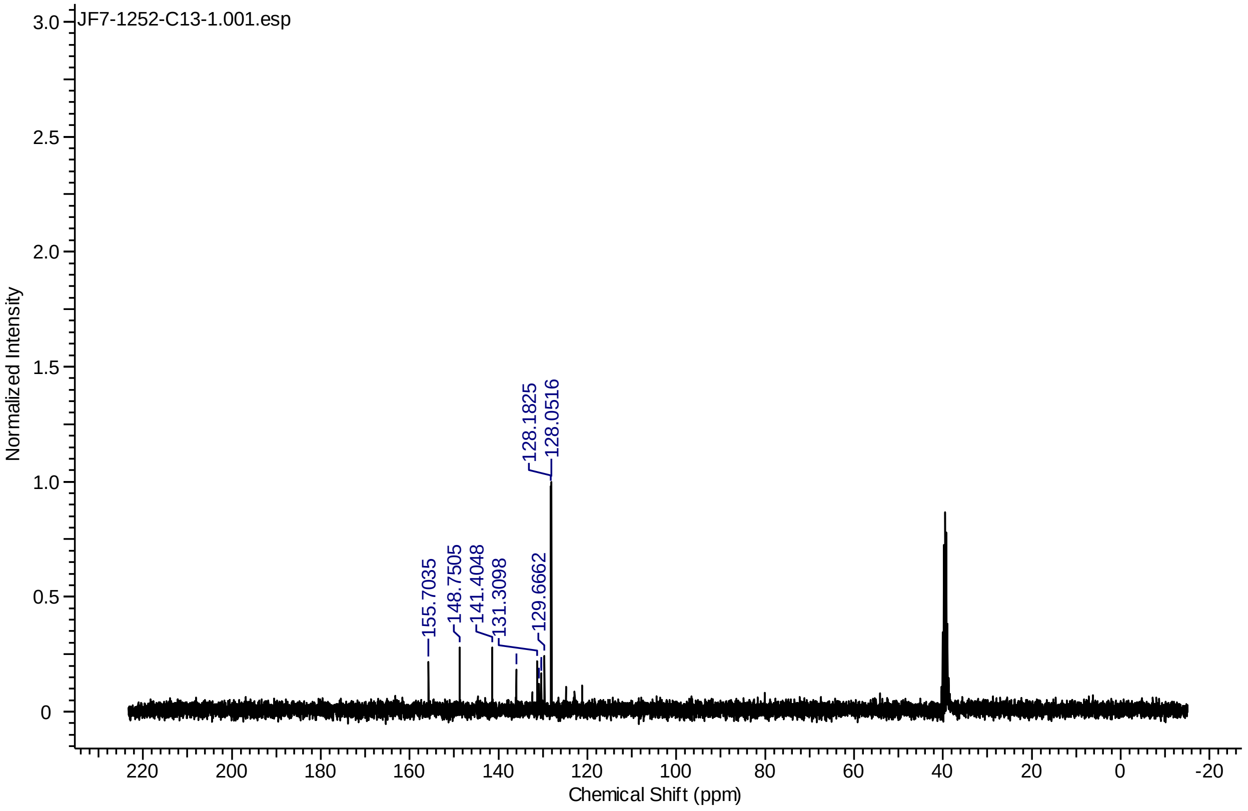
JF7-1252-C13-1.001.esp
Normalized Intensity
Chemical Shift (ppm)
155.7035
148.7505
141.4048
131.3098
129.6662
128.1825
128.0516

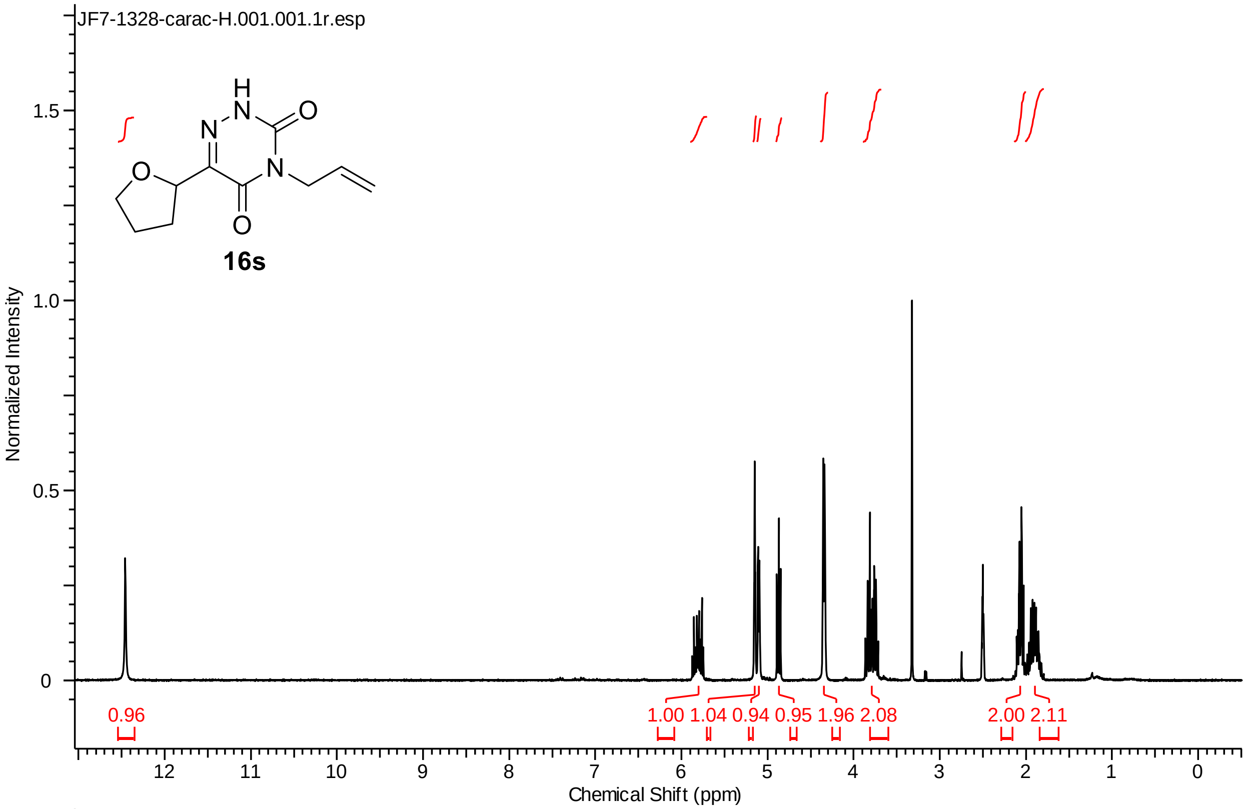
JF7-1328-carac-H.001.001.1r.esp
16s
Normalized Intensity
0.96
1.00 1.04 0.94 0.95 1.96 2.08
2.00 2.11
Chemical Shift (ppm)

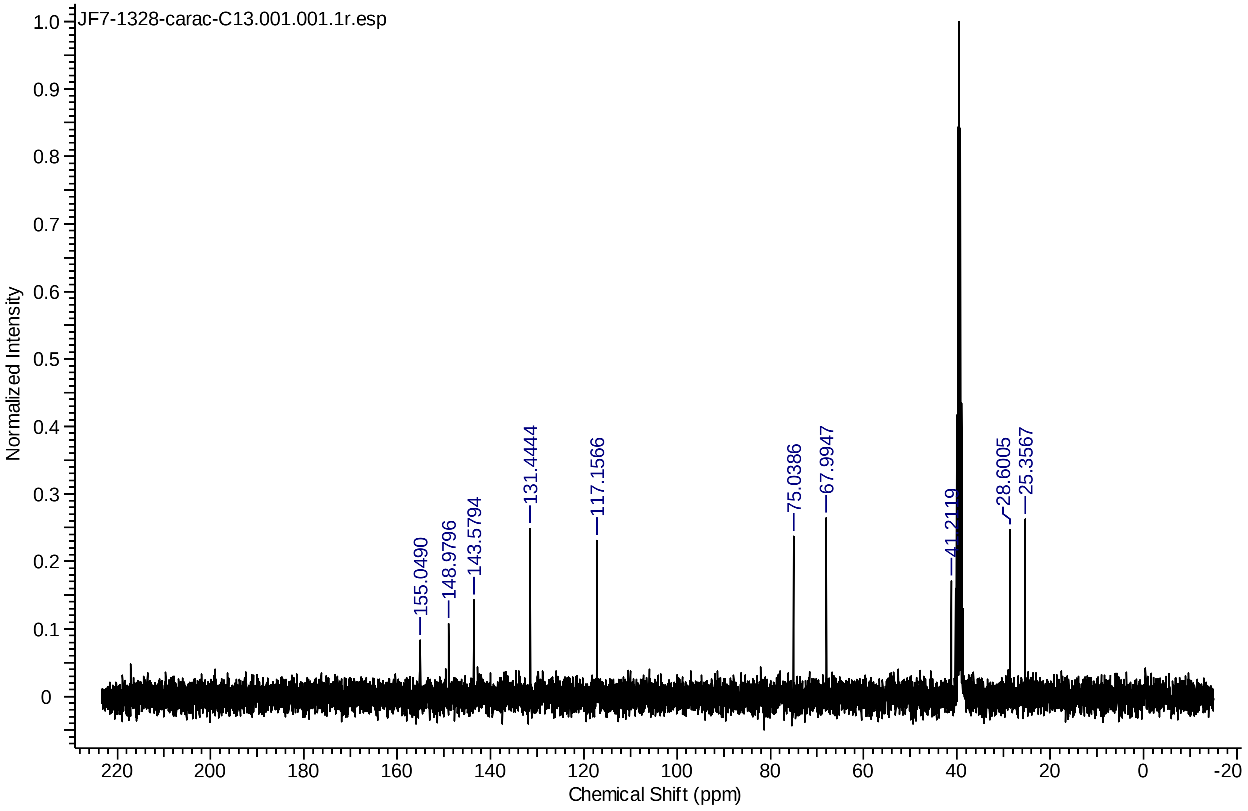
JF7-1328-carac-C13.001.001.1r.esp
Normalized Intensity
155.0490
148.9796
143.5794
131.4444
117.1566
75.0386
67.9947
41.2119
28.6005
25.3567
Chemical Shift (ppm)

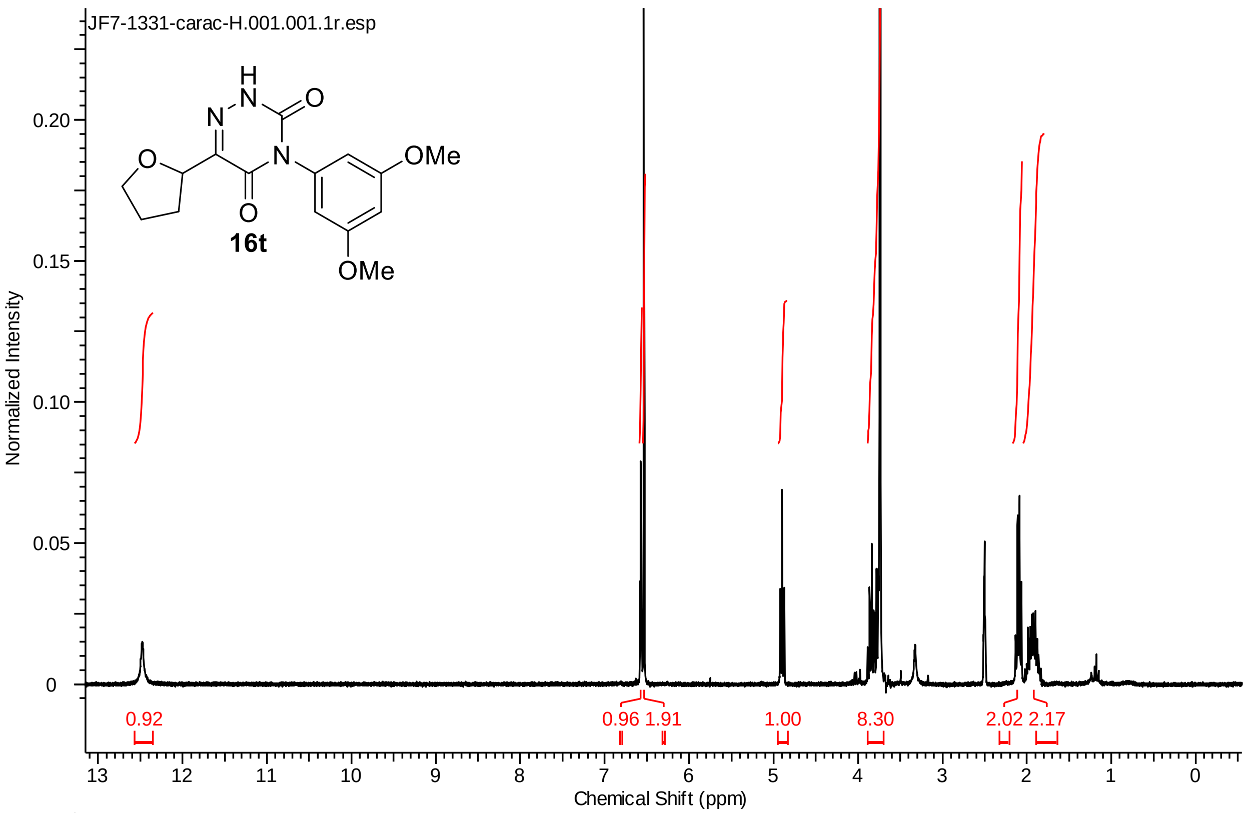
JF7-1331-carac-H.001.001.1r.esp
16t
OMe
OMe
Normalized Intensity
Chemical Shift (ppm)
0.92
0.96 1.91
1.00
8.30
2.02 2.17

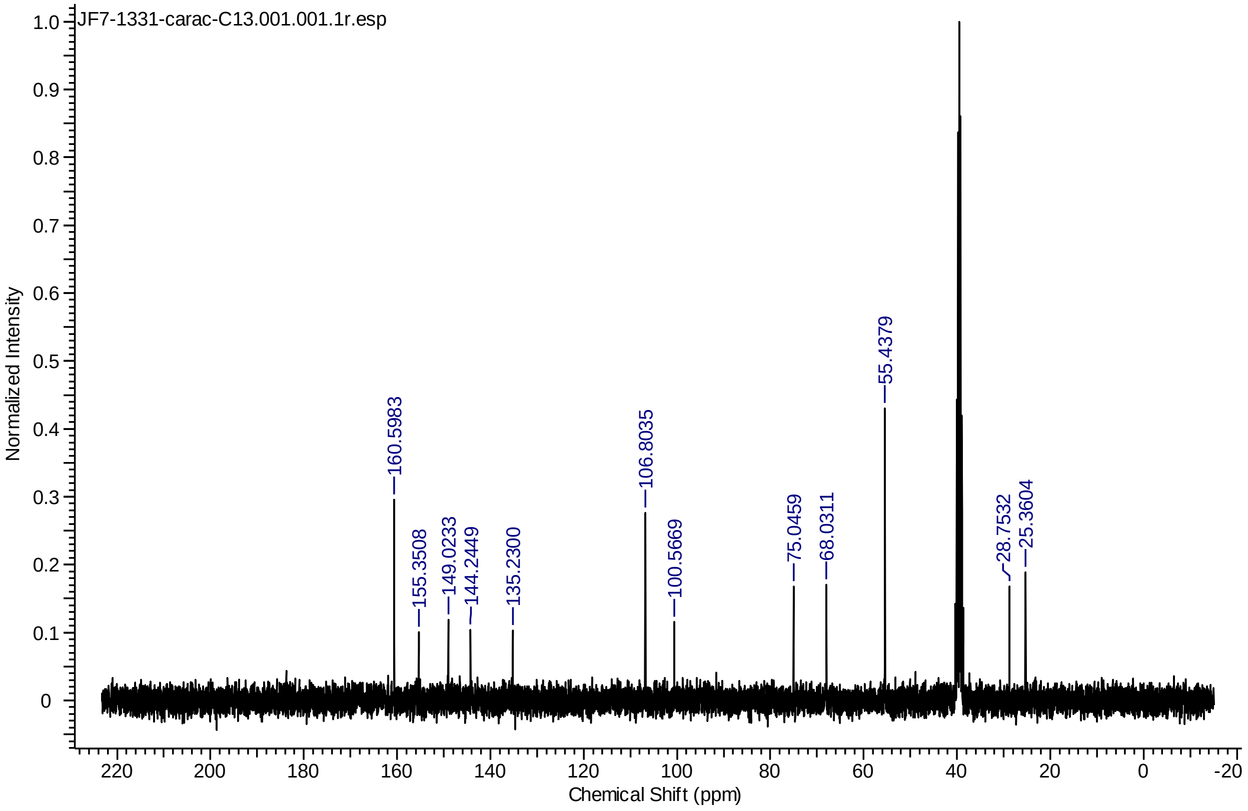
JF7-1331-carac-C13.001.001.1r.esp
Normalized Intensity
Chemical Shift (ppm)
160.5983
155.3508
149.0233
144.2449
135.2300
106.8035
100.5669
75.0459
68.0311
55.4379
28.7532
25.3604

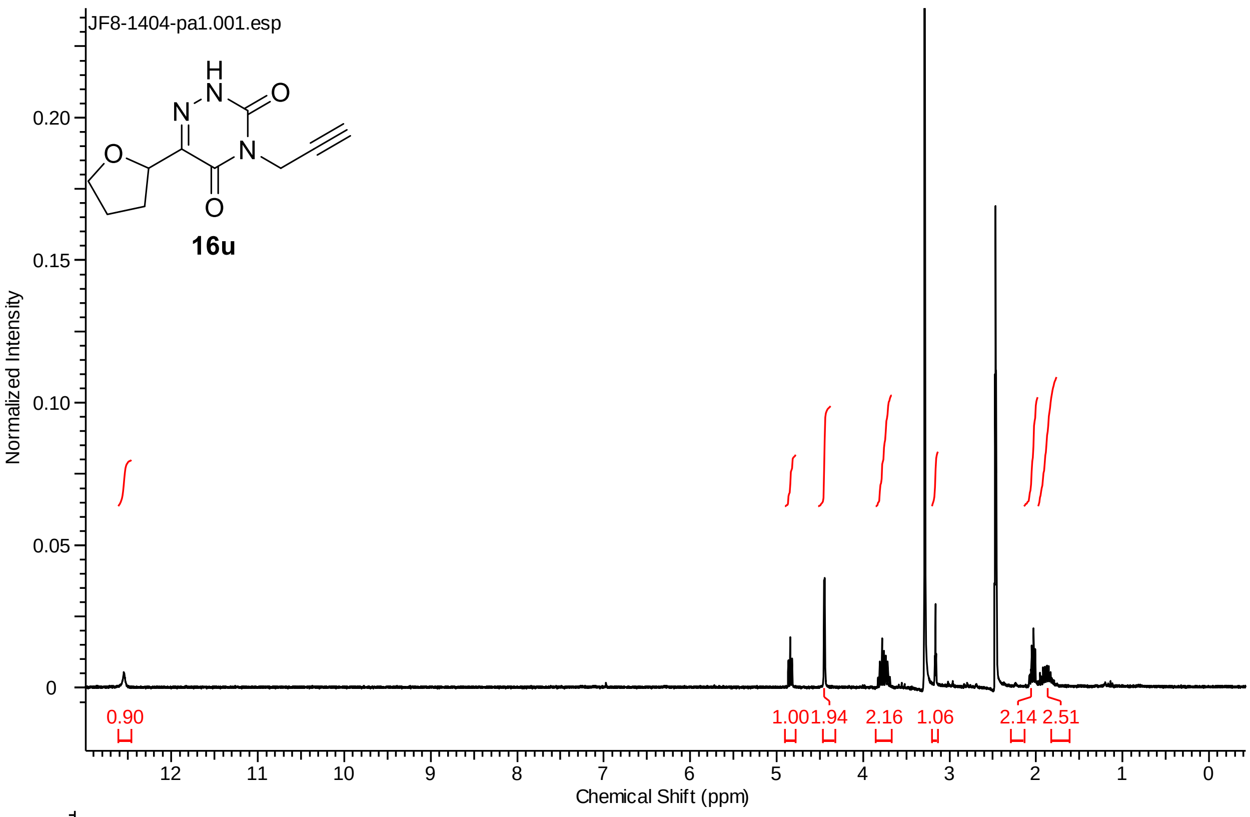
JF8-1404-pa1.001.esp
16u
0.90
1.00
1.94
2.16
1.06
2.14
2.51
Normalized Intensity
Chemical Shift (ppm)

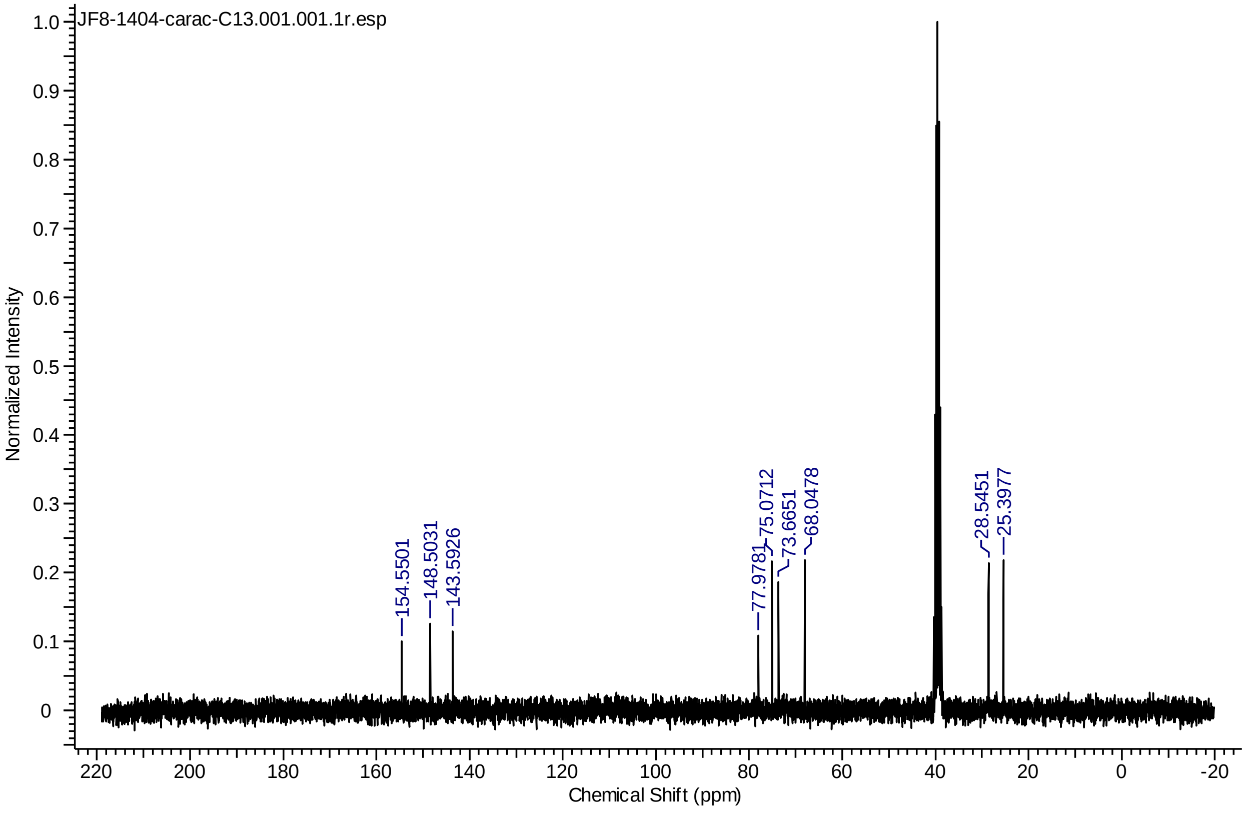
JF8-1404-carac-C13.001.001.1r.esp
154.5501
148.5031
143.5926
77.9781
75.0712
73.6651
68.0478
28.5451
25.3977
Normalized Intensity
Chemical Shift (ppm)

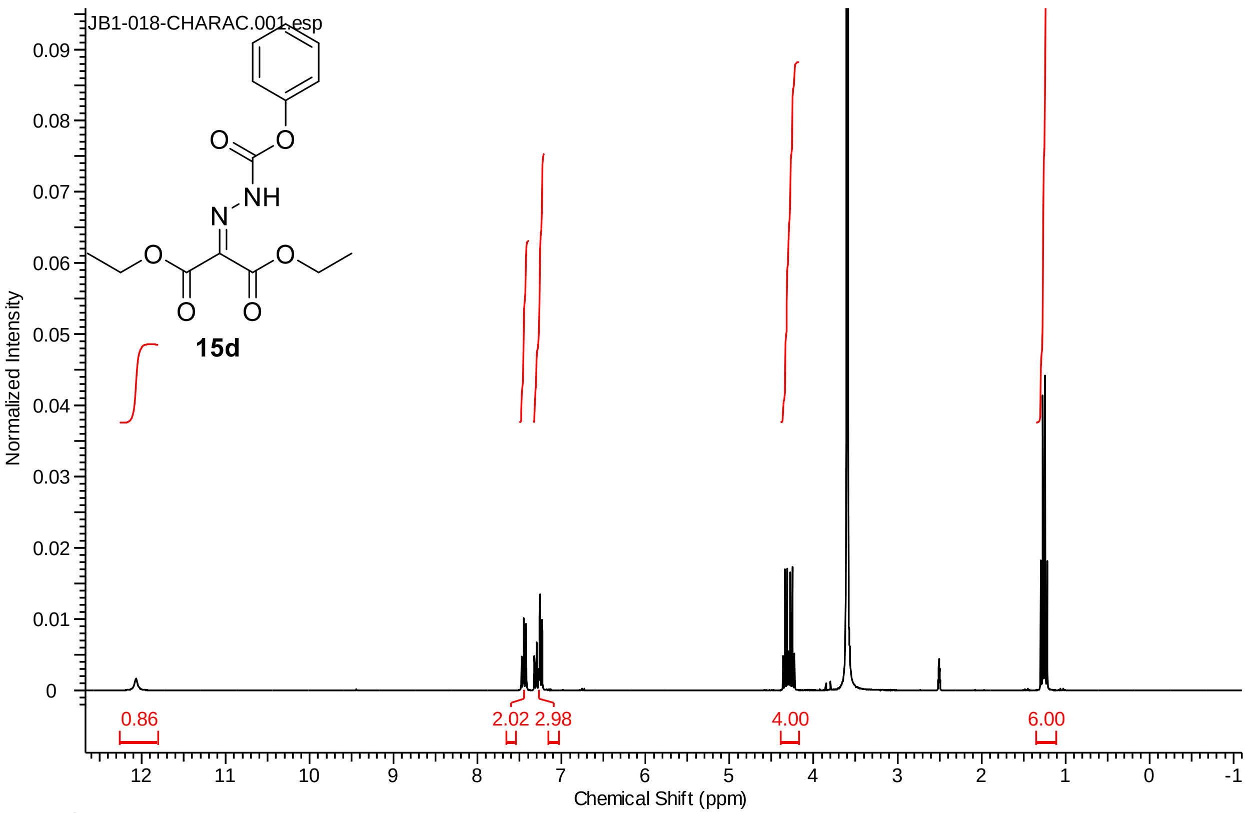
JB1-018-CHARAC.001.esp
15d
Normalized Intensity
0.86
2.02
2.98
4.00
6.00
Chemical Shift (ppm)

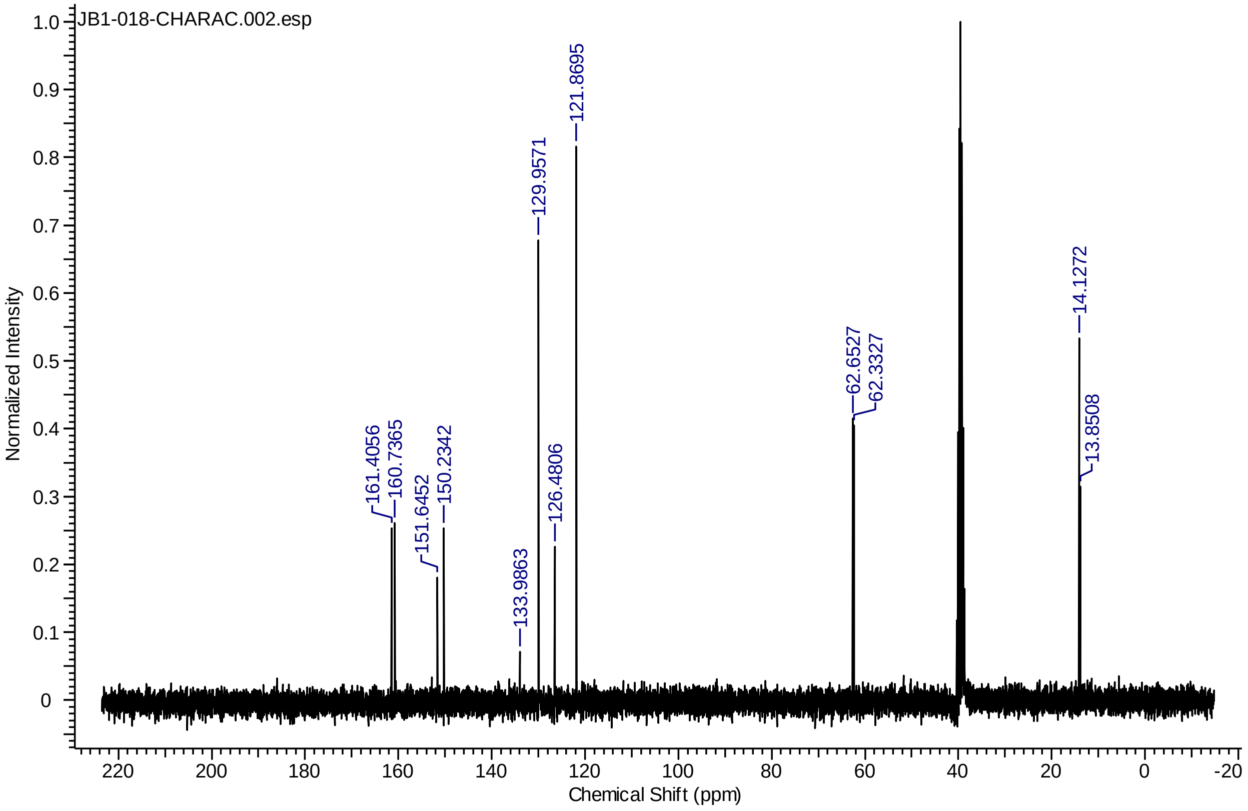
JB1-018-CHARAC.002.esp
Normalized Intensity
161.4056
160.7365
151.6452
150.2342
133.9863
129.9571
126.4806
121.8695
62.6527
62.3327
14.1272
13.8508
Chemical Shift (ppm)

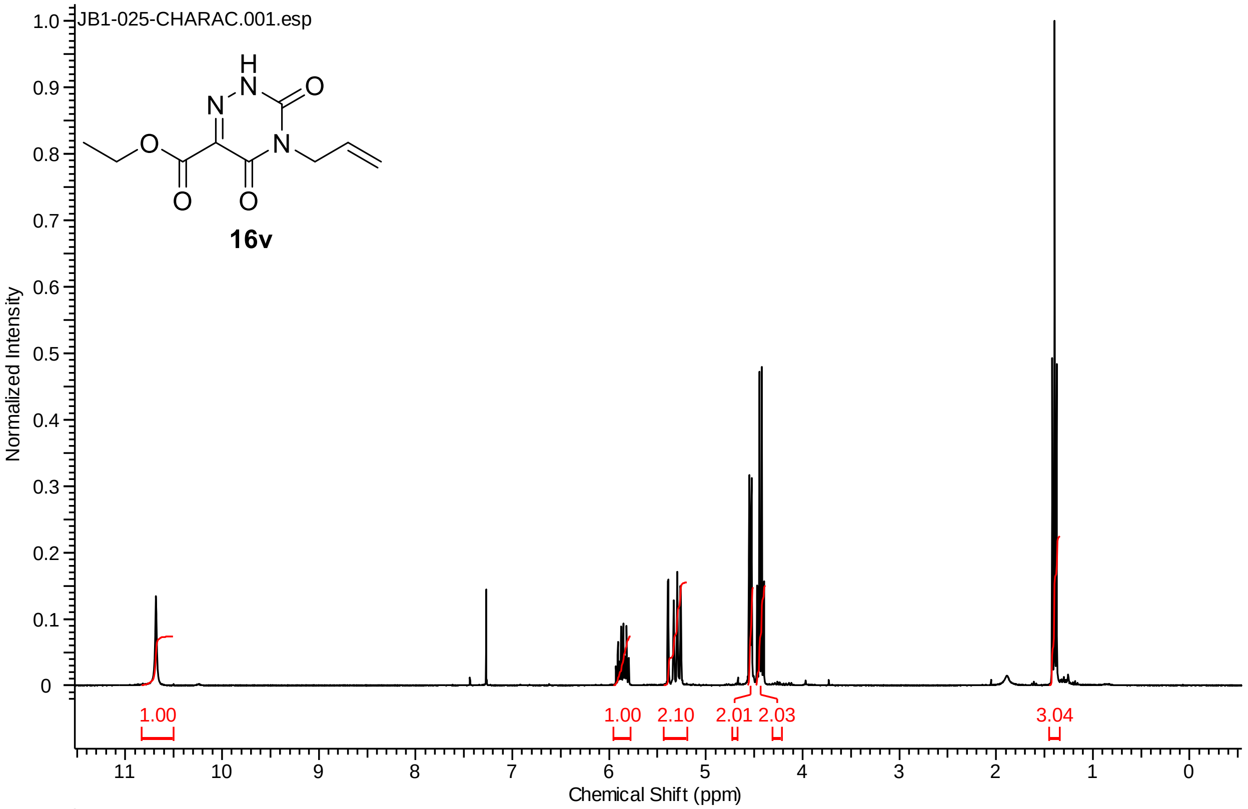
JB1-025-CHARAC.001.esp
16v
Normalized Intensity
1.00
1.00
2.10
2.01
2.03
3.04
Chemical Shift (ppm)

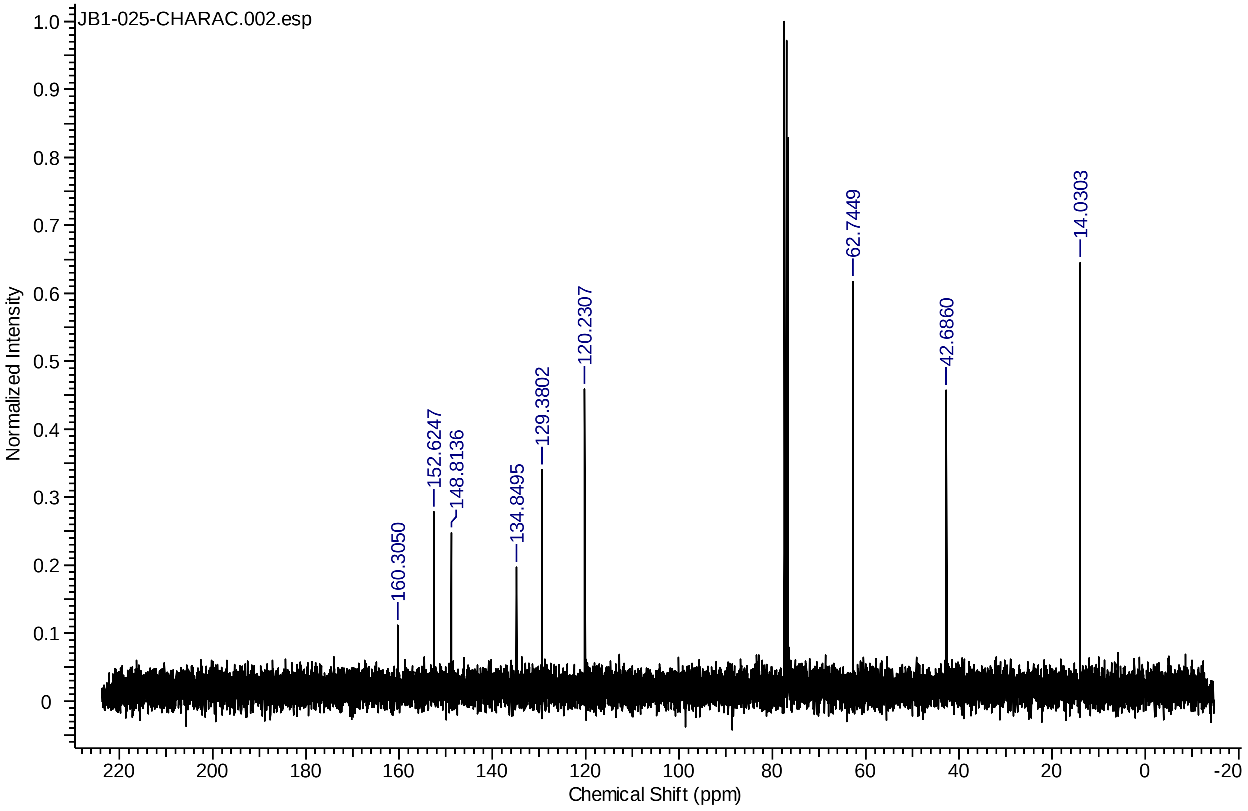
JB1-025-CHARAC.002.esp
Normalized Intensity
160.3050
152.6247
148.8136
134.8495
129.3802
120.2307
62.7449
42.6860
14.0303
Chemical Shift (ppm)

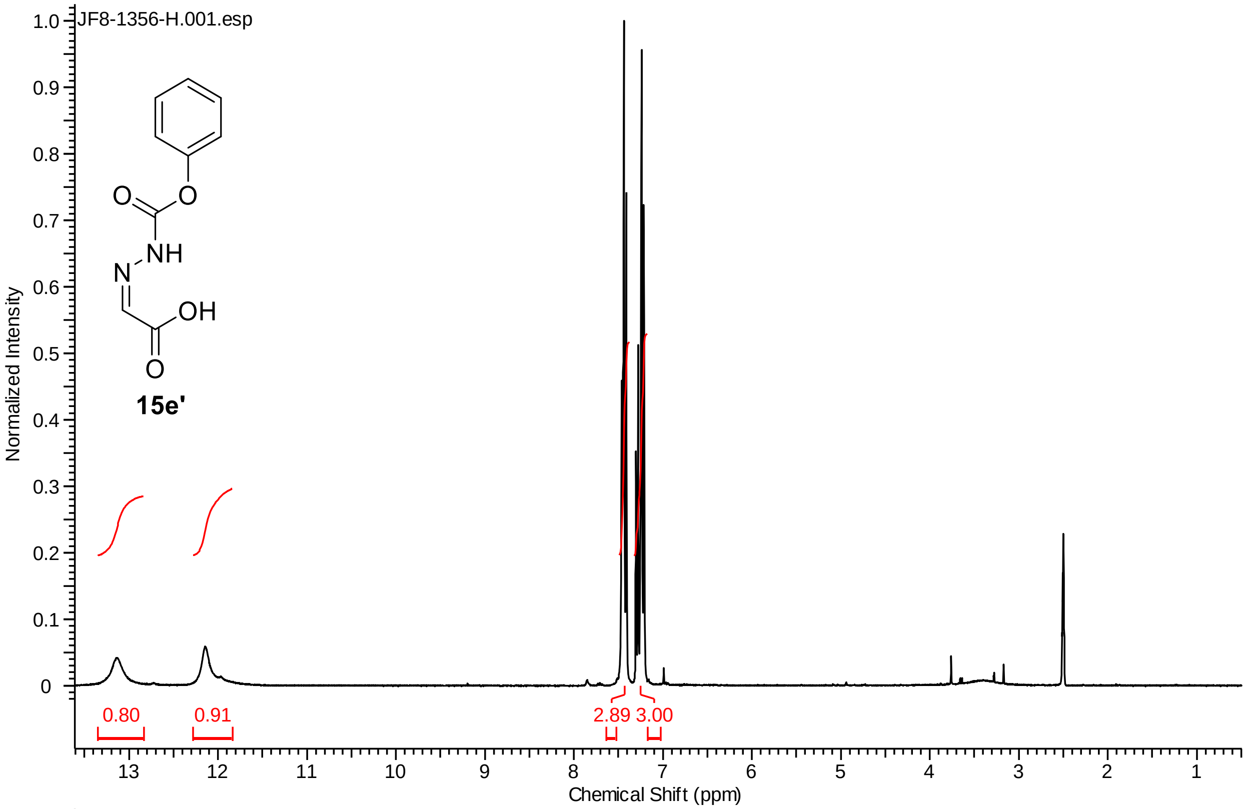
JF8-1356-H.001.esp
15e'
0.80
0.91
2.89
3.00
Normalized Intensity
Chemical Shift (ppm)

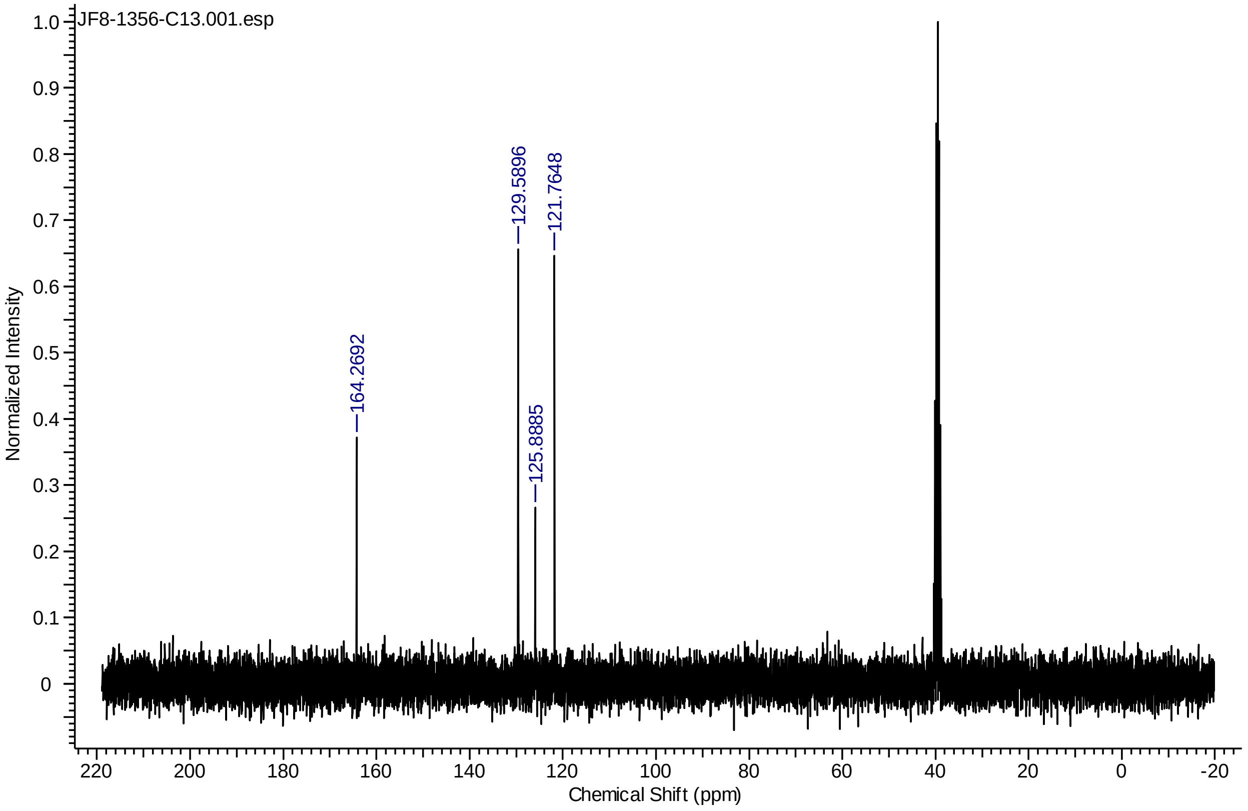
JF8-1356-C13.001.esp
164.2692
129.5896
125.8885
121.7648
Normalized Intensity
Chemical Shift (ppm)

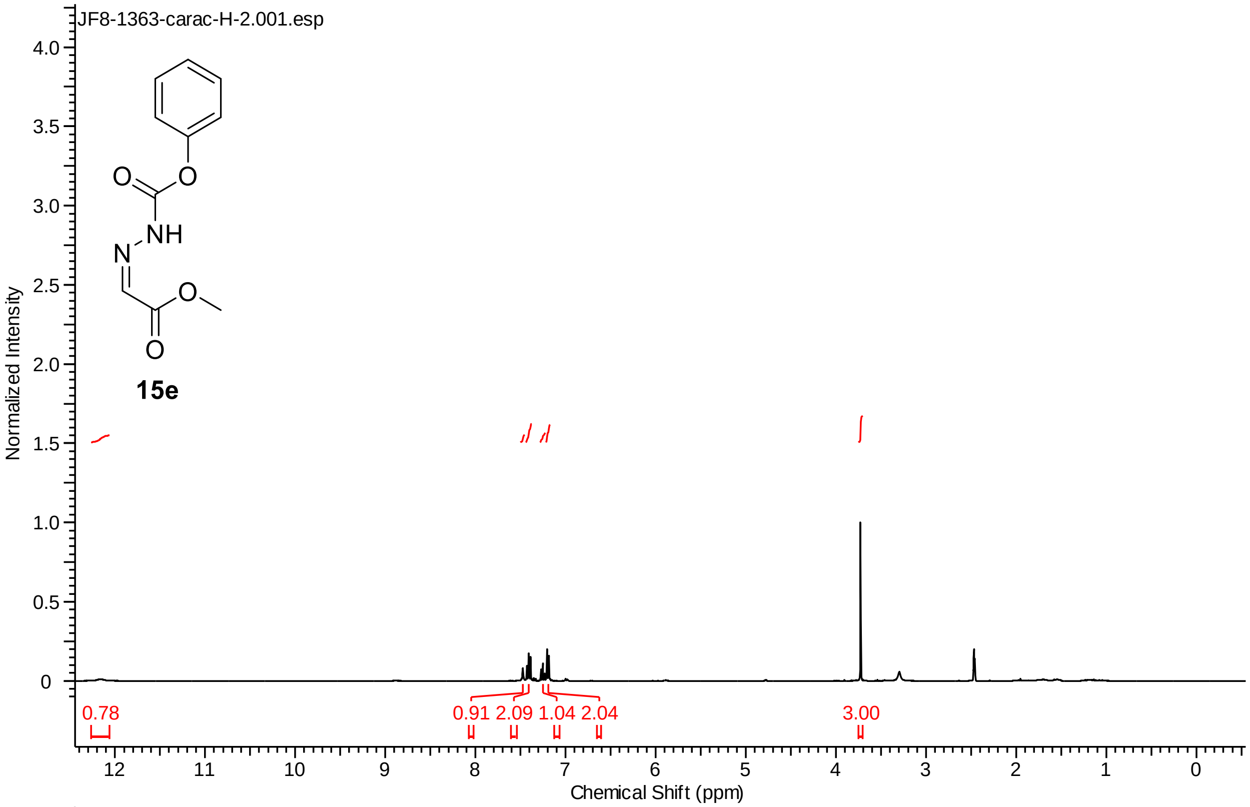
JF8-1363-carac-H-2.001.esp
15e
0.78
0.91 2.09 1.04 2.04
3.00
Normalized Intensity
Chemical Shift (ppm)

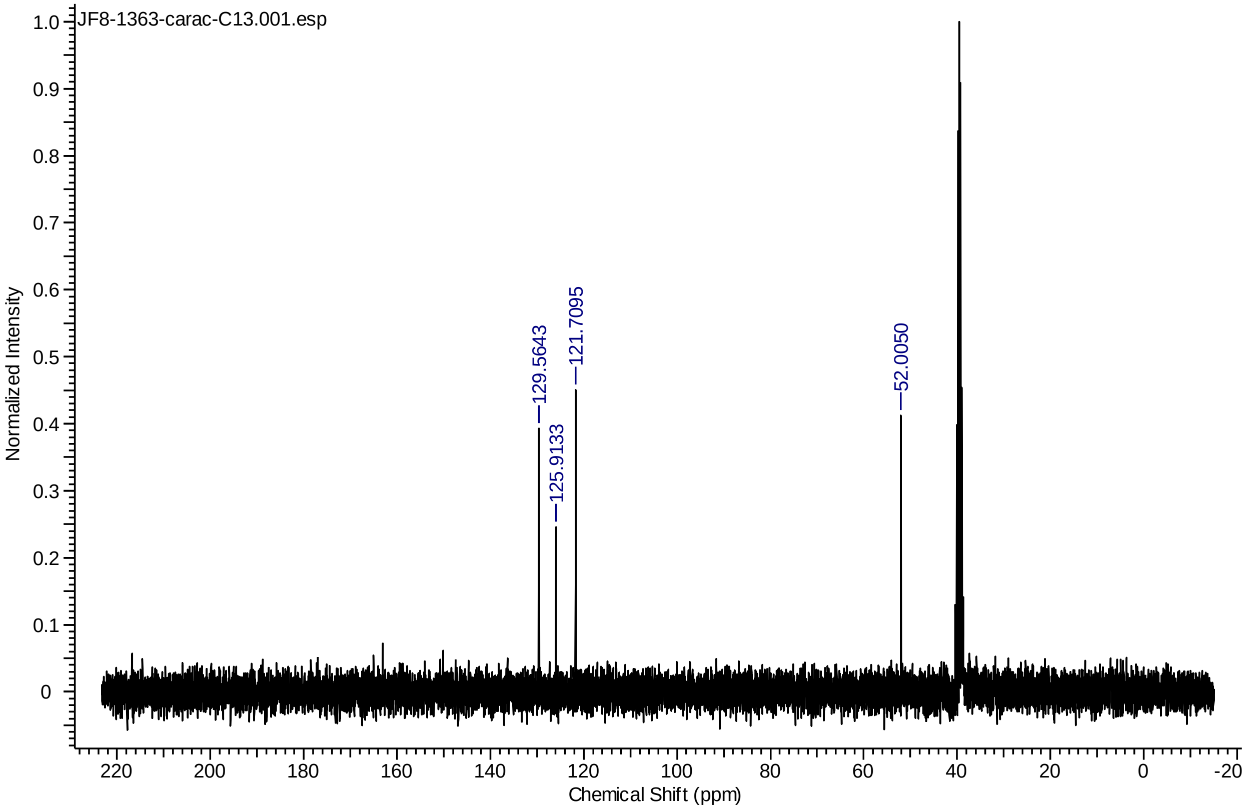
JF8-1363-carac-C13.001.esp
129.5643
125.9133
121.7095
52.0050
Normalized Intensity
Chemical Shift (ppm)

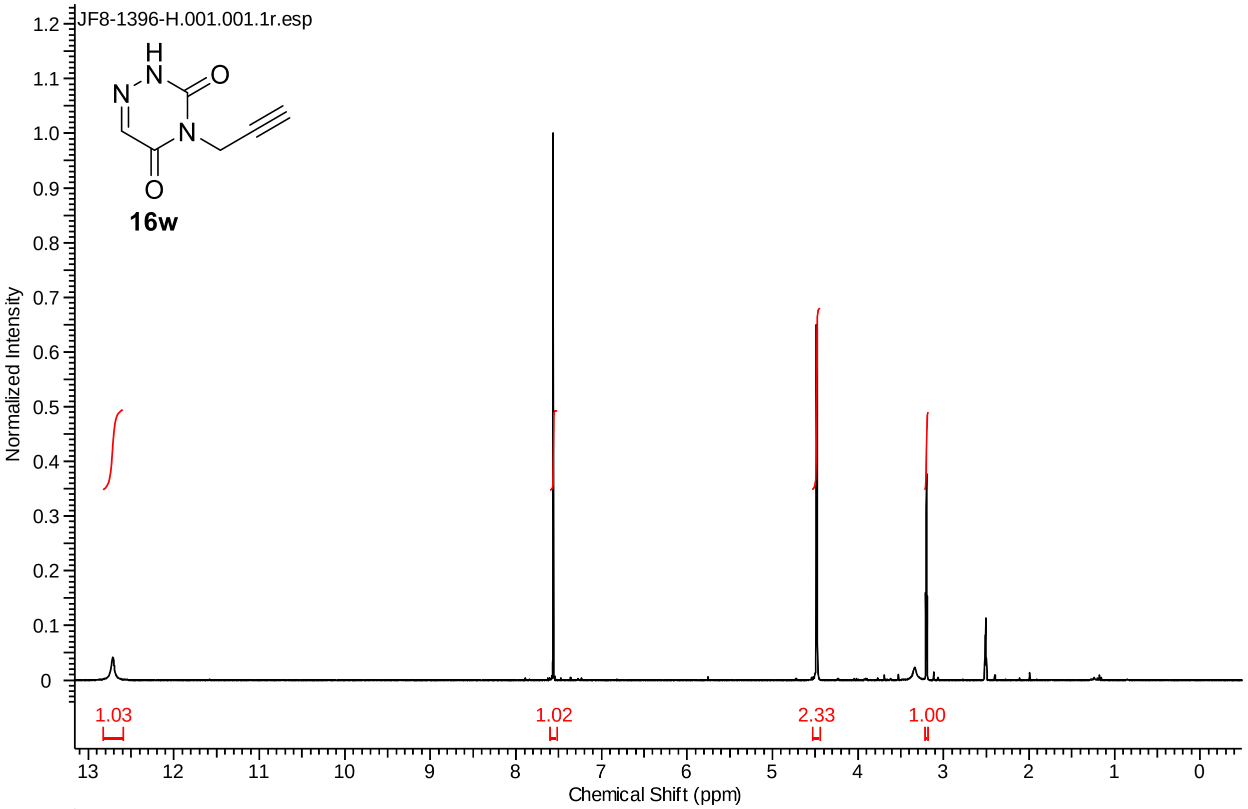
JF8-1396-H.001.001.1r.esp
16w
1.03
1.02
2.33
1.00
Normalized Intensity
Chemical Shift (ppm)

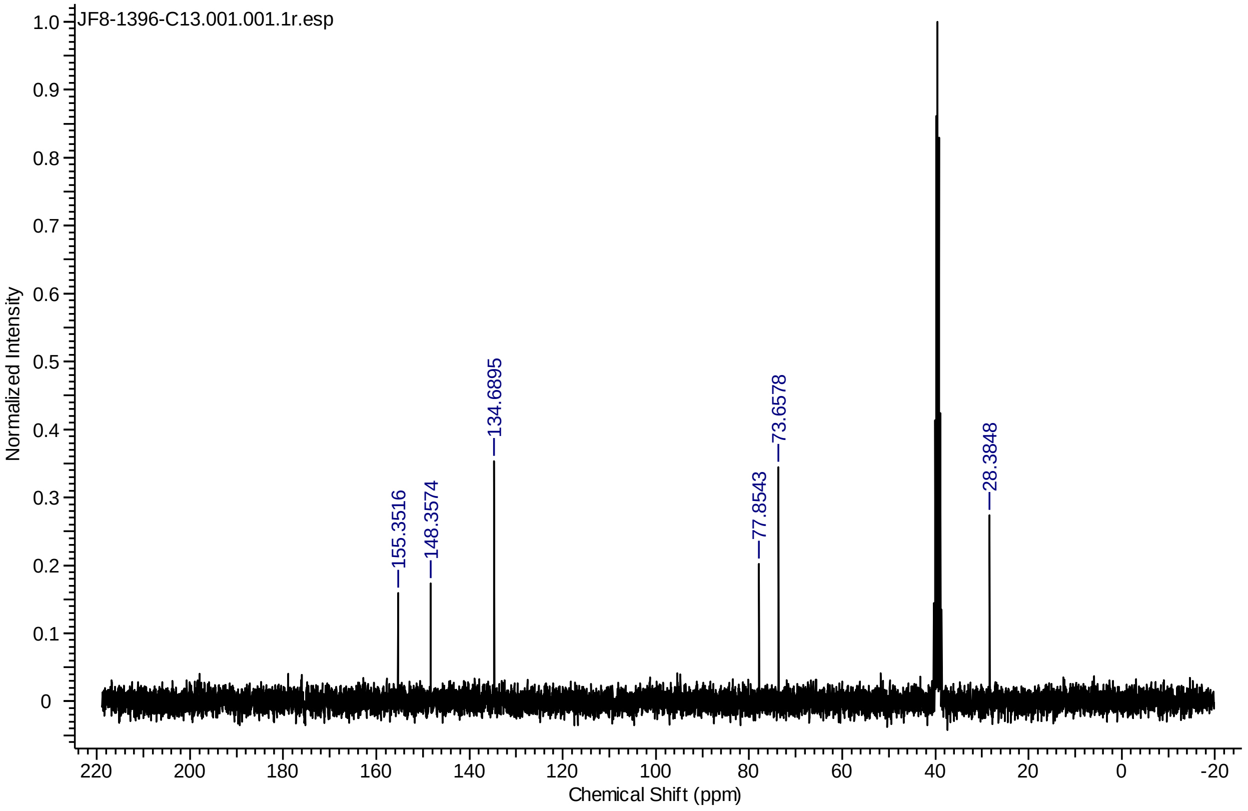
JF8-1396-C13.001.001.1r.esp
155.3516
148.3574
134.6895
77.8543
73.6578
28.3848
Normalized Intensity
Chemical Shift (ppm)

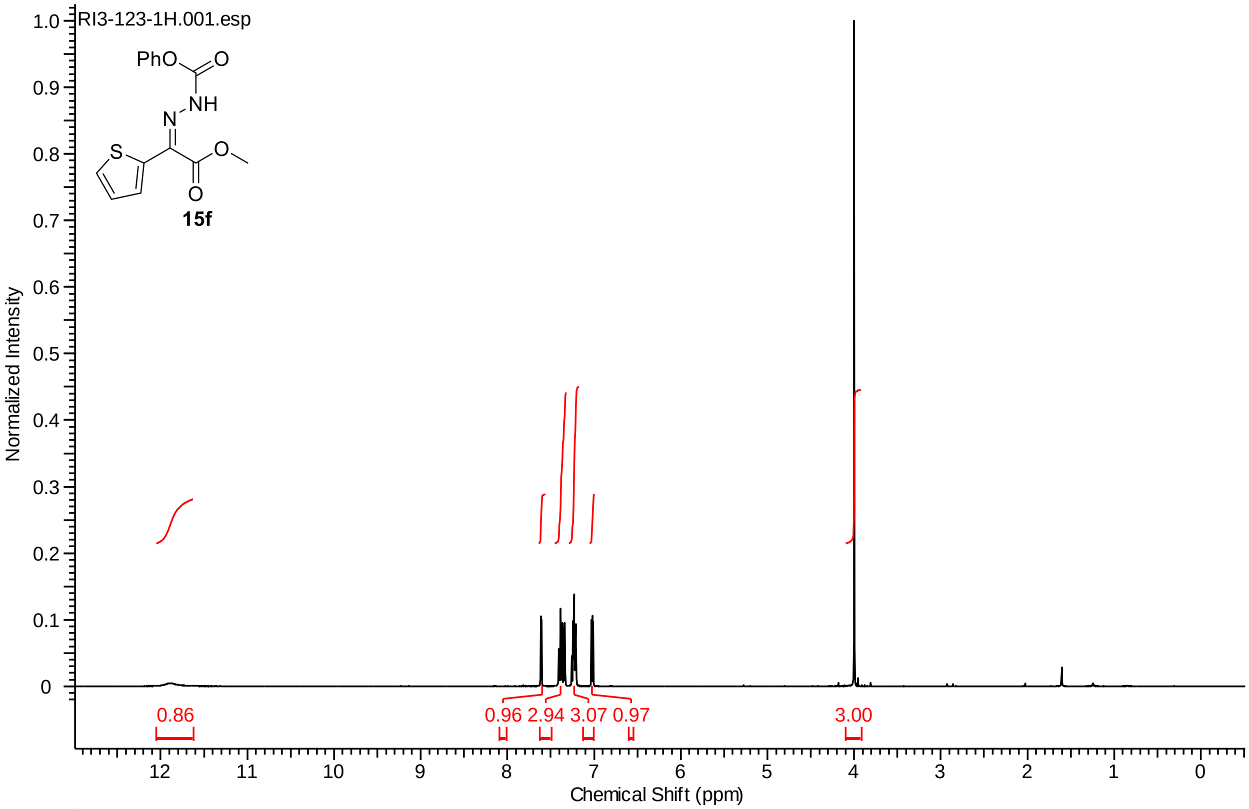
RI3-123-1H.001.esp
PhO
O
NH
N
S
O
O
15f
0.86
0.96
2.94
3.07
0.97
3.00
Normalized Intensity
Chemical Shift (ppm)

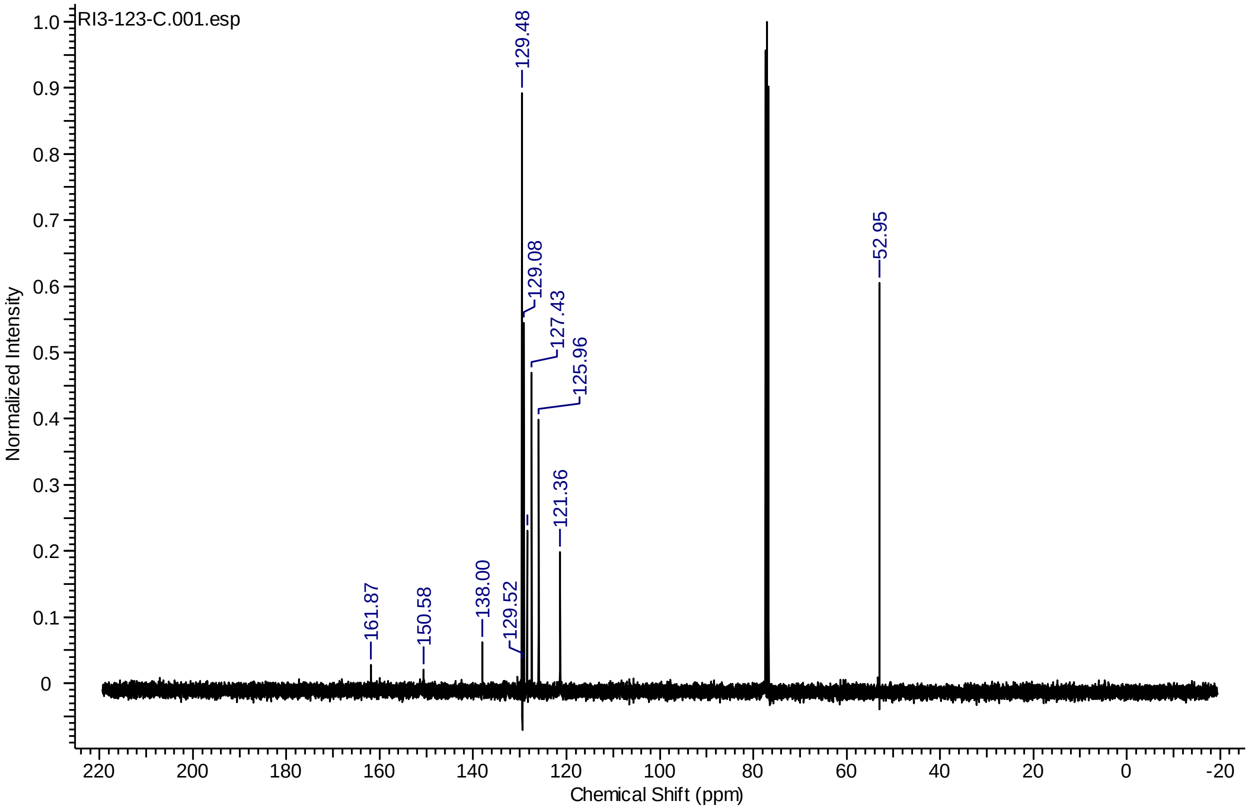
RI3-123-C.001.esp
161.87
150.58
138.00
129.52
129.48
129.08
127.43
125.96
121.36
52.95
Normalized Intensity
Chemical Shift (ppm)

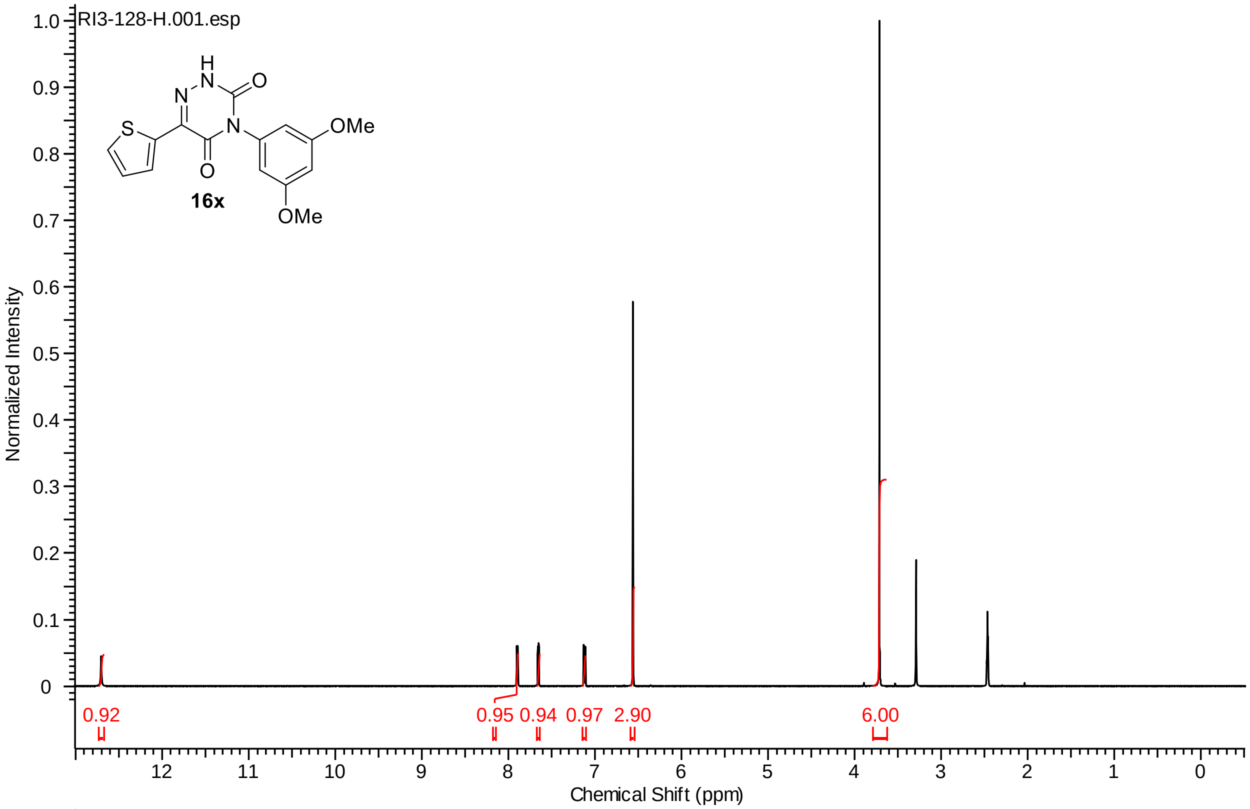
RI3-128-H.001.esp
16x
OMe
OMe
0.92
0.95
0.94
0.97
2.90
6.00
Chemical Shift (ppm)
Normalized Intensity

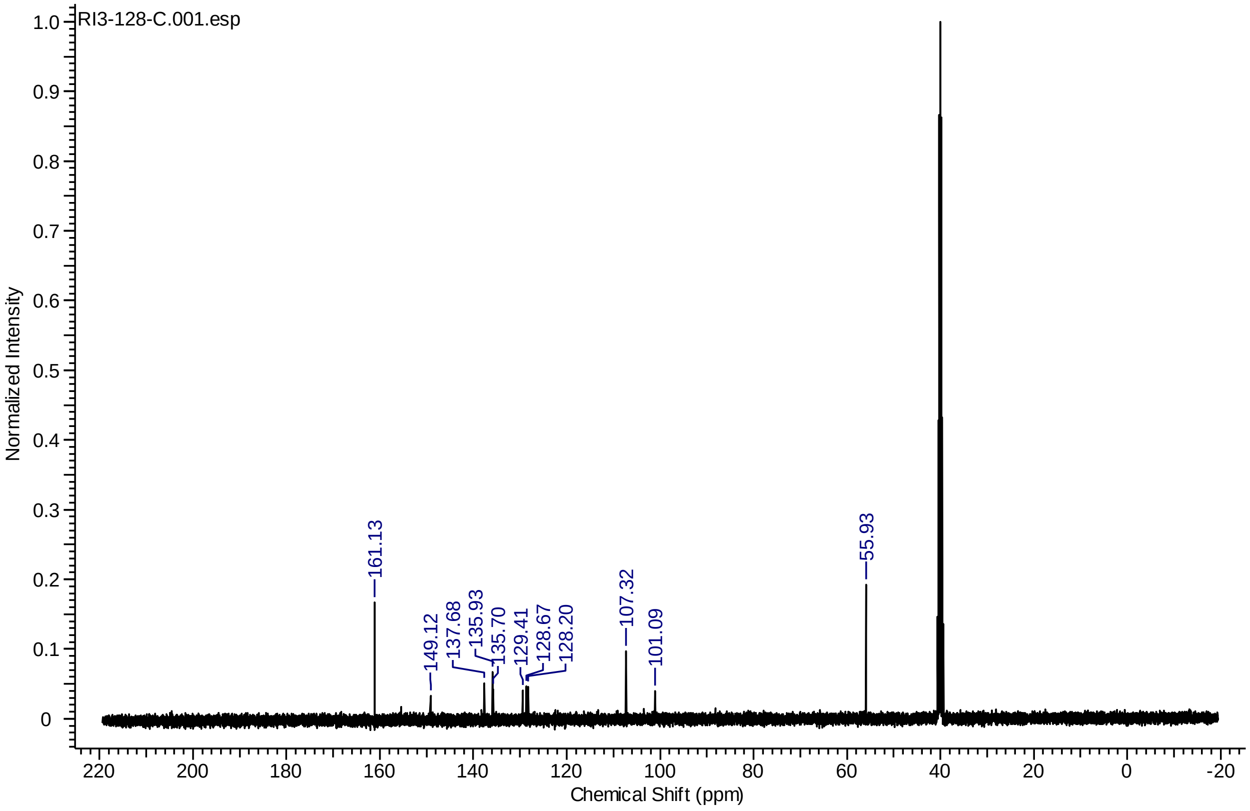
RI3-128-C.001.esp
161.13
149.12
137.68
135.93
135.70
129.41
128.67
128.20
107.32
101.09
55.93
Chemical Shift (ppm)
Normalized Intensity

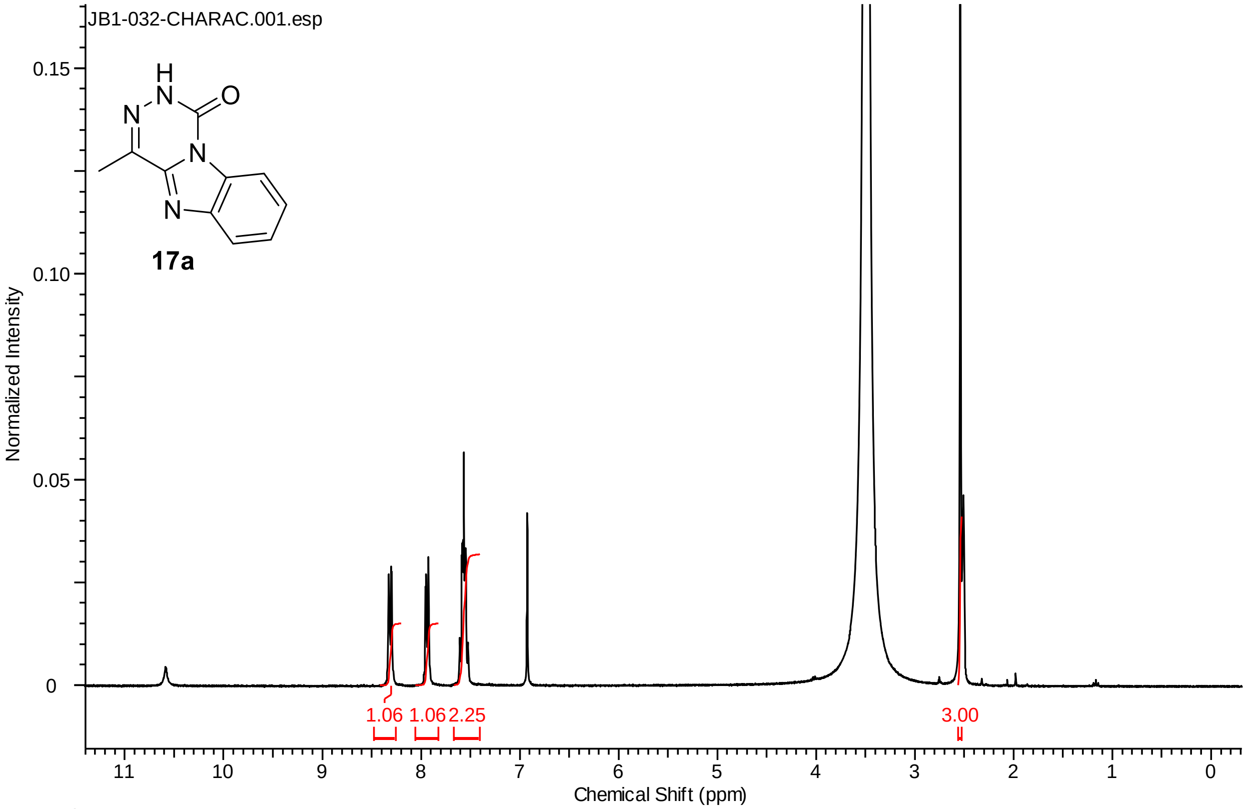
JB1-032-CHARAC.001.esp
17a
Normalized Intensity
1.06 1.06 2.25
3.00
Chemical Shift (ppm)

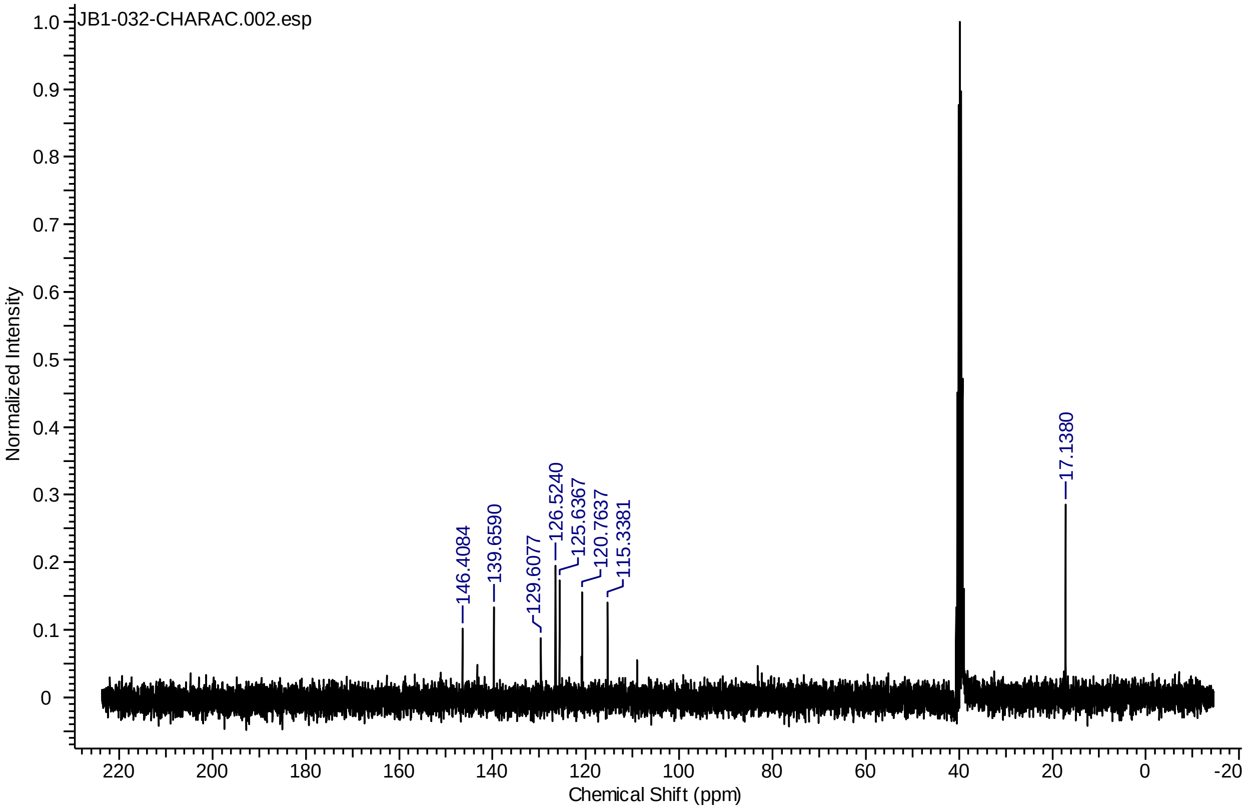
JB1-032-CHARAC.002.esp
Normalized Intensity
146.4084
139.6590
129.6077
126.5240
125.6367
120.7637
115.3381
17.1380
Chemical Shift (ppm)

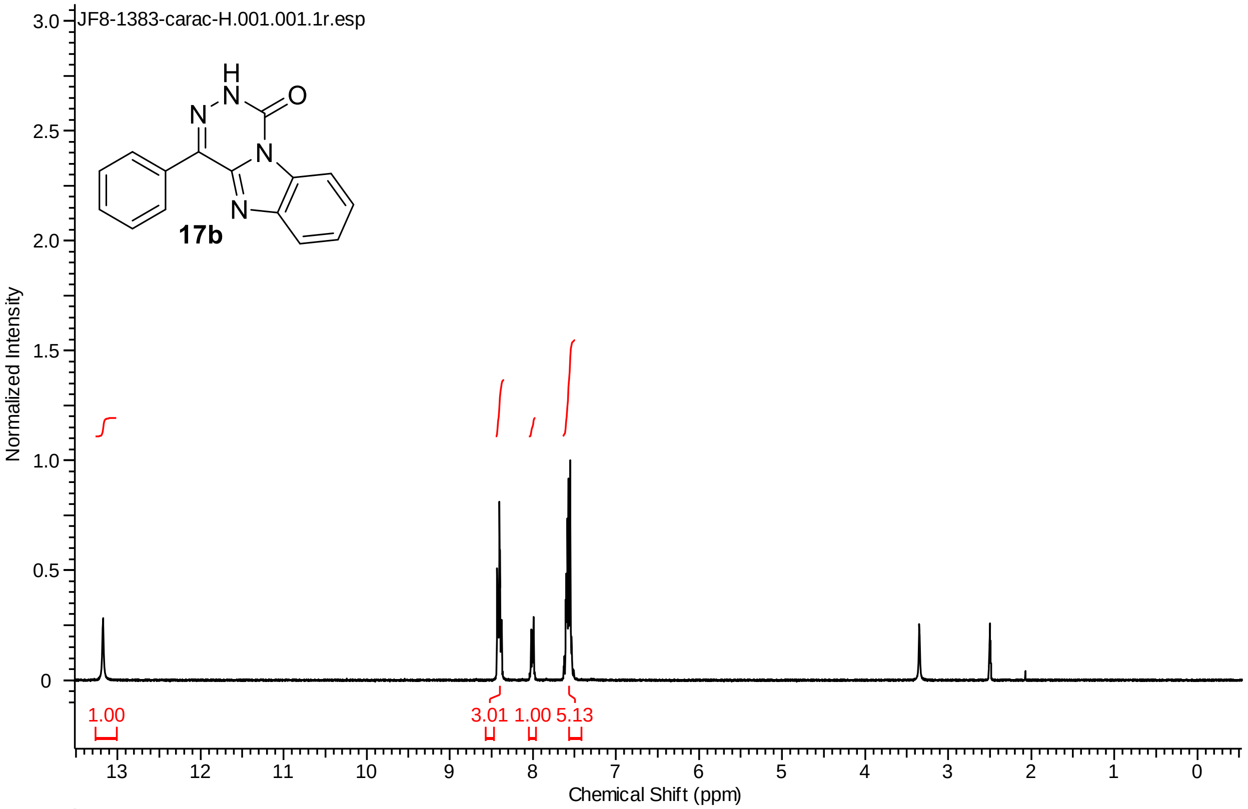
JF8-1383-carac-H.001.001.1r.esp
17b
Normalized Intensity
1.00
3.01
1.00
5.13
Chemical Shift (ppm)

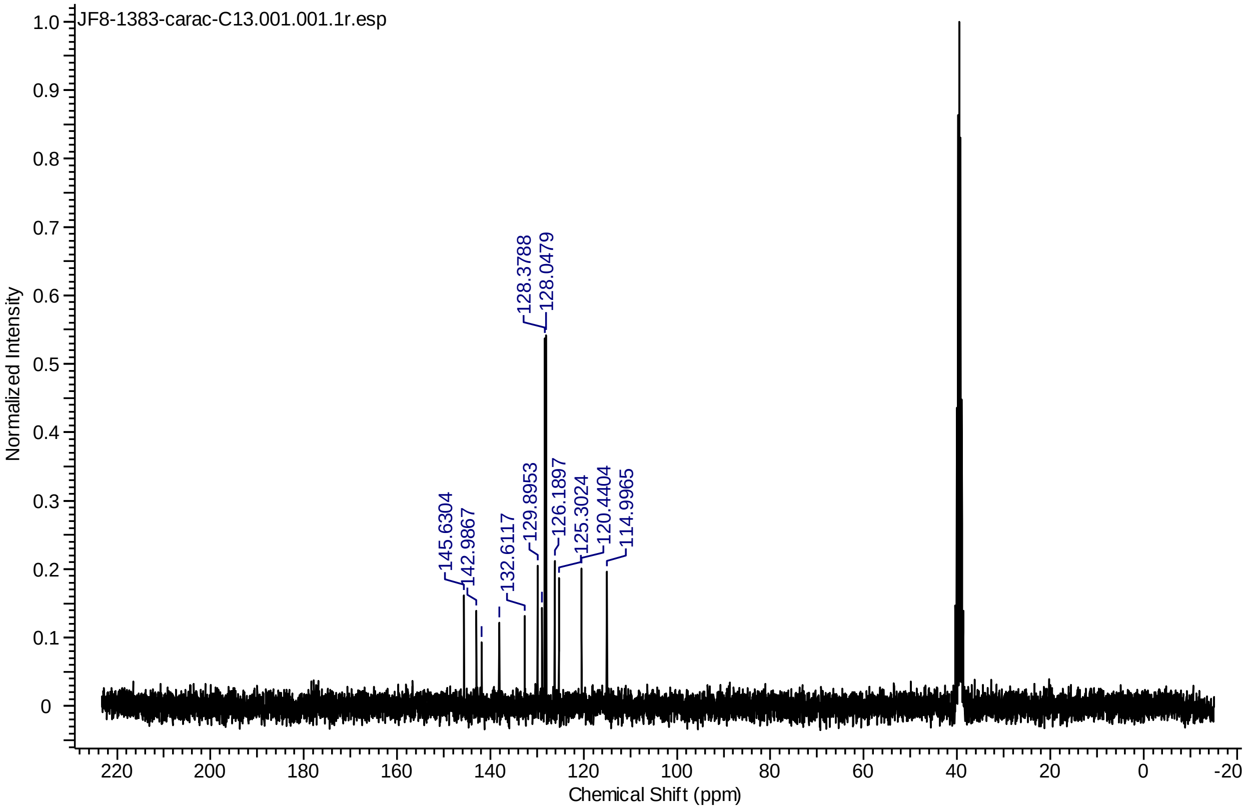
JF8-1383-carac-C13.001.001.1r.esp
Normalized Intensity
145.6304
142.9867
132.6117
129.8953
128.3788
128.0479
126.1897
125.3024
120.4404
114.9965
Chemical Shift (ppm)

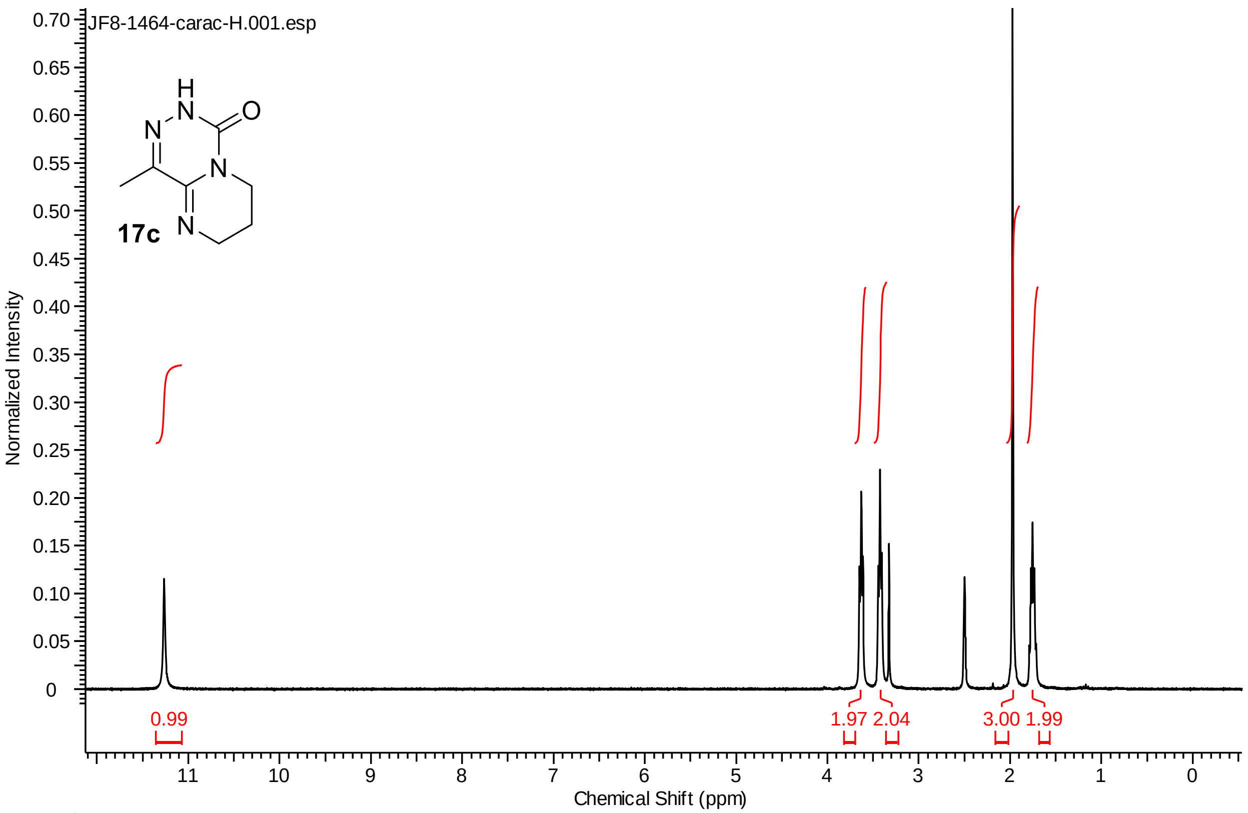
JF8-1464-carac-H.001.esp
17c
Normalized Intensity
0.99
1.97
2.04
3.00
1.99
Chemical Shift (ppm)

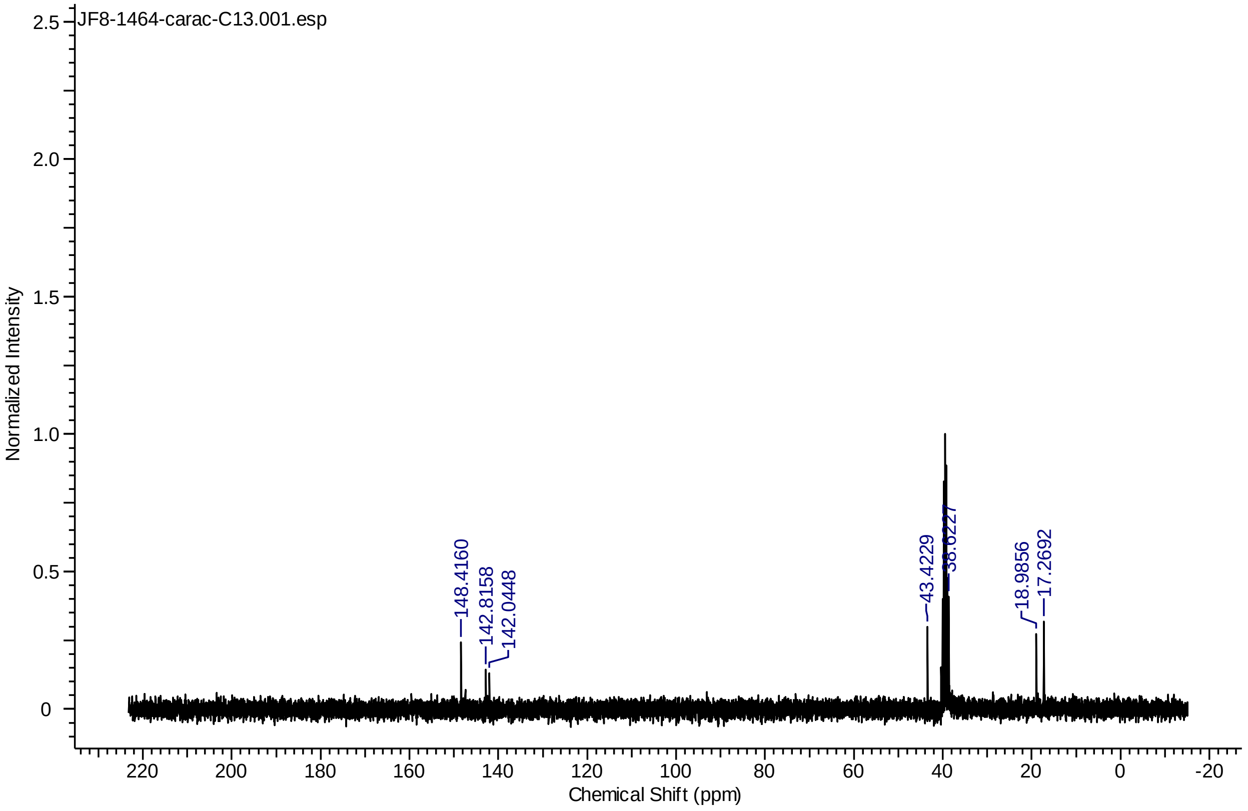
JF8-1464-carac-C13.001.esp
Normalized Intensity
148.4160
142.8158
142.0448
43.4229
18.9856
17.2692
Chemical Shift (ppm)

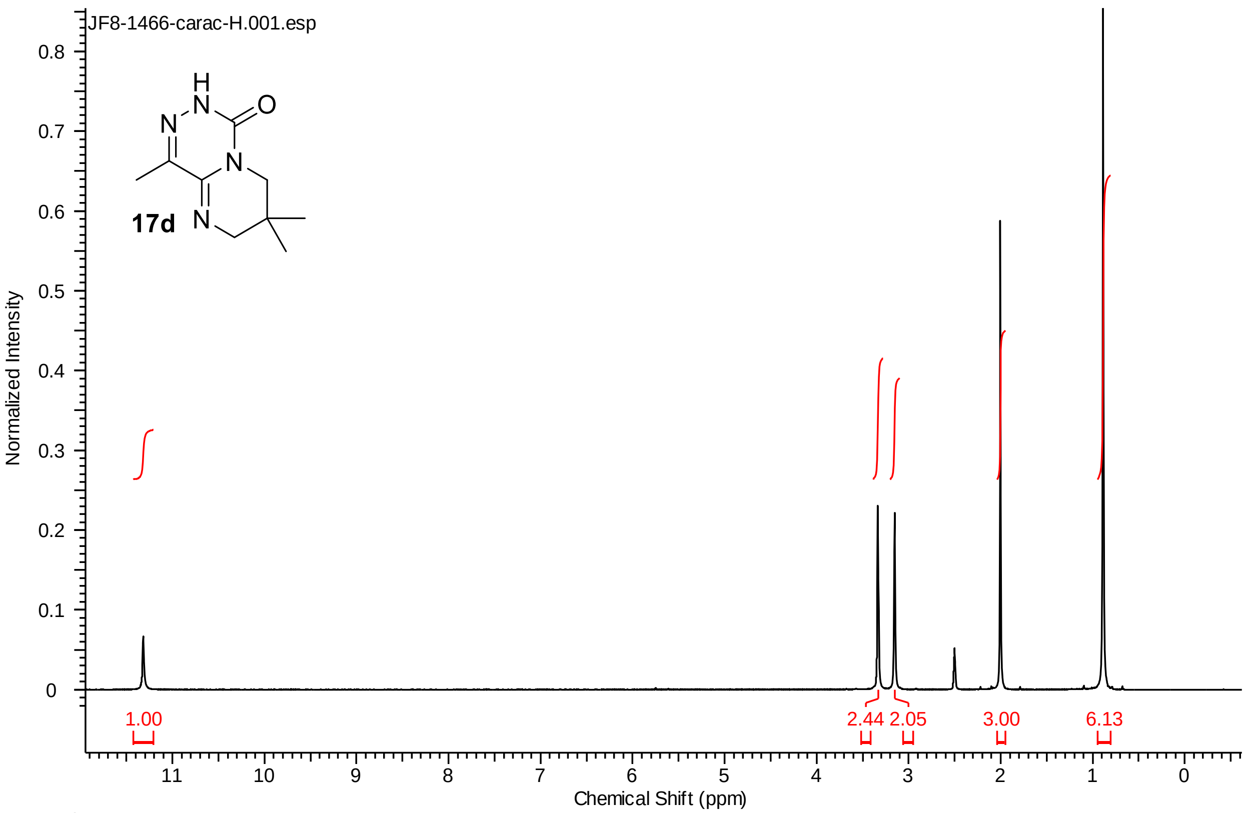
JF8-1466-carac-H.001.esp
17d
1.00
2.44
2.05
3.00
6.13
Normalized Intensity
Chemical Shift (ppm)

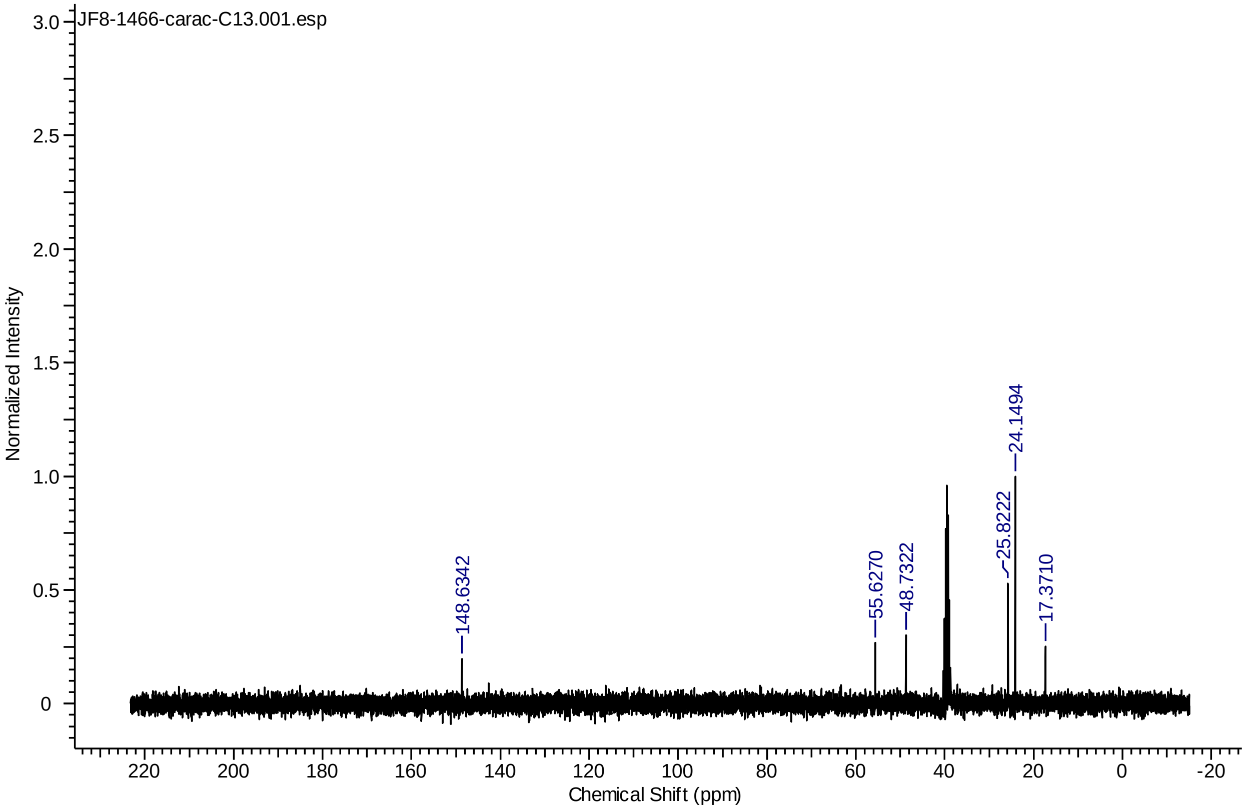
JF8-1466-carac-C13.001.esp
148.6342
55.6270
48.7322
25.8222
24.1494
17.3710
Normalized Intensity
Chemical Shift (ppm)

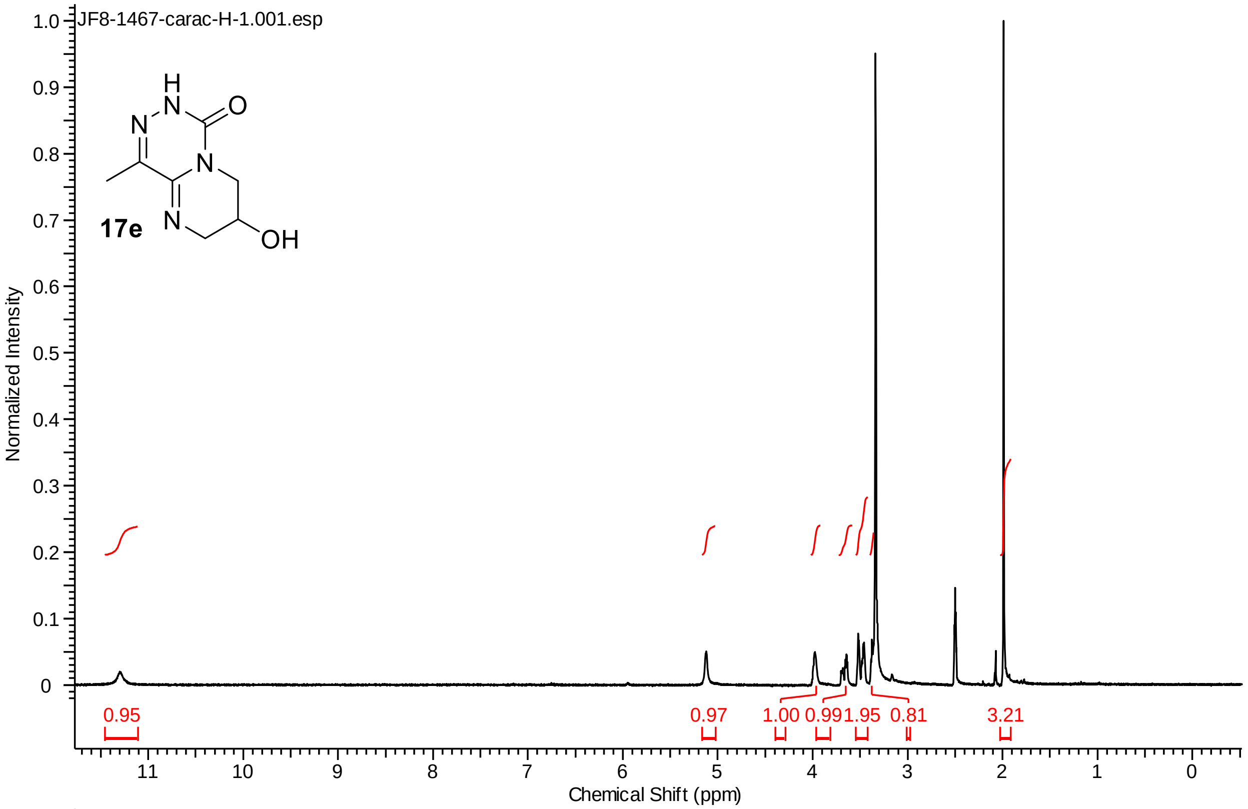
JF8-1467-carac-H-1.001.esp
17e
0.95
0.97
1.00
0.99
1.95
0.81
3.21
Normalized Intensity
Chemical Shift (ppm)

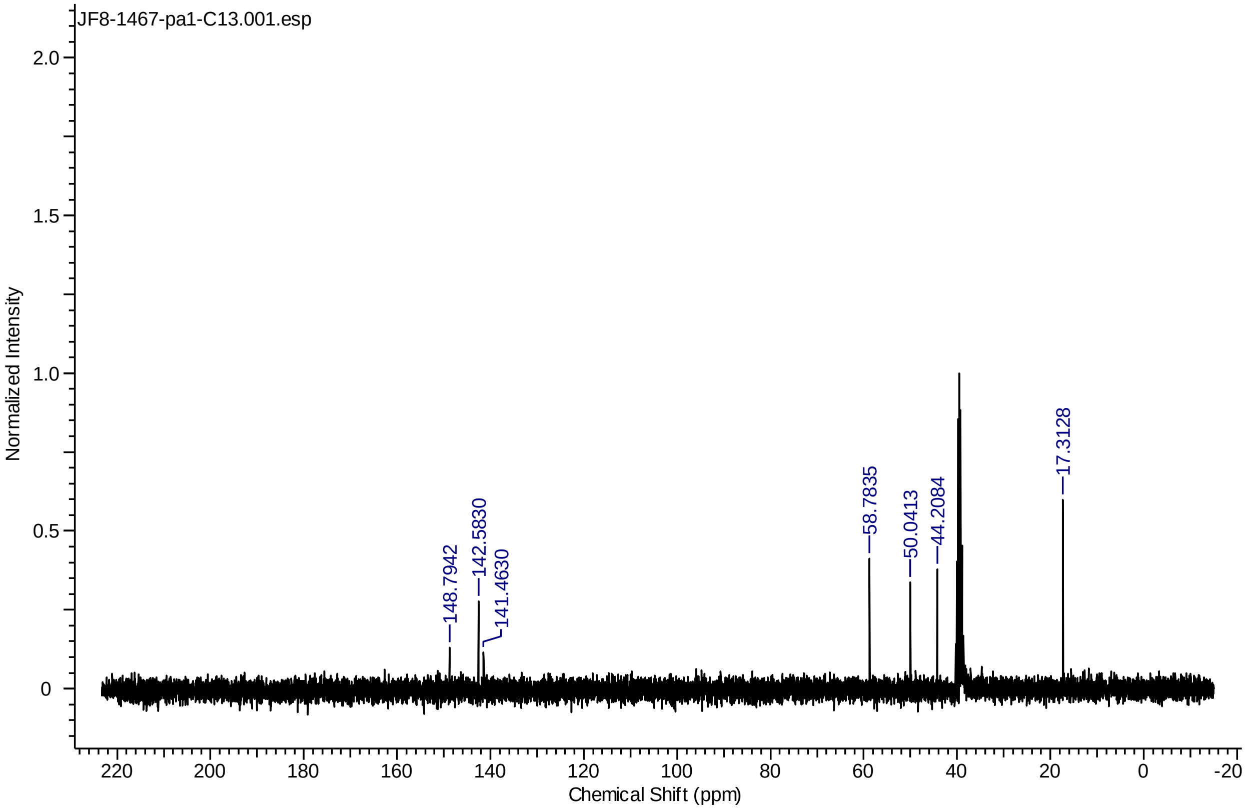
JF8-1467-pa1-C13.001.esp
148.7942
142.5830
141.4630
58.7835
50.0413
44.2084
17.3128
Normalized Intensity
Chemical Shift (ppm)
